# One‐Step Glycoengineering of NK Cells With High‐Affinity Siglec Ligands for Cancer Immunotherapy

**DOI:** 10.1002/advs.202522474

**Published:** 2026-03-02

**Authors:** Shuai Hu, Ben Huang, Lingyan Wang, Qiang Guo, Cuiping Jiang, Ruicheng Qi, Shuyao Wang, Lin‐Tai Da, Wenjie Peng

**Affiliations:** ^1^ Key Laboratory of Systems Biomedicine (Ministry of Education) Shanghai Center for Systems Biomedicine Shanghai Jiao Tong University Shanghai China

**Keywords:** chemoenzymatic glycoengineering, CMP‐sialic acid synthetase evolution, computational modelling, high affinity Siglec ligands, immunotherapeutic glycan remodeling

## Abstract

Siglecs, a family of sialic acid (Sia)‐binding immunomodulatory receptors selectively expressed on immune cells, are promising immunotherapeutic targets. While synthetic Sia derivatives can manipulate the Sia‐Siglec axis with high affinity, their therapeutic application has been hampered by the poor substrate tolerance of wild‐type CMP‐sialic acid synthase (CSS) for sterically demanding analogs. Here, we report a structure‐guided engineering strategy to evolve *Neisseria meningitidis* CMP‐Sia synthetase (NmCSS) for enhanced activity with bulky substrates. Coupling this optimized NmCSS variant with a sialyltransferase enabled a scalable “one‐pot two‐enzyme” (OPTE) synthesis of diverse sialoside analogs. Screening this library on glycan microarrays revealed novel high‐affinity ligands with selective Siglec binding profiles. Leveraging the OPTE system, we achieved single‐step glycoengineering of natural killer (NK)‐92MI cells with tailored Siglec‐2 or Siglec‐3 ligands, which exhibited potent cytotoxicity against B‐cell lymphoma (Siglec‐2^+^) and acute myeloid leukemia (Siglec‐3^+^) models. These engineered NK cells displayed significantly enhanced tumor killing capacity, mediated by enhanced granzyme release and cytokine production while maintaining excellent cell viability. This modular platform addresses critical limitations in enzymatic synthesis of modified sialosides and their efficient display on therapeutic cells. Our work establishes a versatile and practical platform for developing next‐generation immunotherapies that precisely target the Sia‐Siglec axis with improved specificity and functionality.

## Graphical abstract

This study develops an engineered NmCSS enzyme with improved activity for bulky group modified sialic acids, enabling efficient synthesis of structurally diverse sialoside analogs in a single step. Glycan microarray screening identified Siglec high‐affinity ligands, which were incorporated onto natural killer (NK) cells via a one‐pot enzymatic system. The glycoengineered NK cells showed enhanced tumor‐killing against B‐cell lymphoma and AML models through improved effector functions, establishing a versatile platform for Siglec‐targeted immunotherapy.

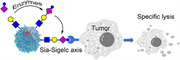

## 1 Introduction

The sialic acid (Sia)‐binding immunoglobulin‐like lectin (Siglec, Sig) family exhibits cell‐type specific expression patterns across immune cell subsets, serving as key regulators of immune cell activation thresholds [1]. These transmembrane receptors typically contain cytoplasmic immunoreceptor tyrosine‐based inhibitory motifs (ITIMs) that, upon engagement with cell‐surface sialoglycans (Siaα2,3‐/6‐/8‐Gal sequences), recruit Src‐homology 2 domain (SH2)‐containing phosphatase (SHP)‐1/2 to initiate inhibitory signaling cascades [2, 3]. This Sia‐Siglec axis represents an evolutionarily conserved “self‐recognition” system that maintains immune tolerance while preventing aberrant inflammatory responses. However, malignant cells have adopted this immunoregulatory mechanism through aberrant surface hypersialylation to engage inhibitory Siglecs (e.g., Sig‐7/9 on natural killer cells, Sig‐2 on B cells), creating an immunosuppressive tumor microenvironment [4, 5]. The unique combination of restricted expression, endocytic capacity, and checkpoint‐like functionality has positioned Siglecs as an attractive therapeutic target for cancer and immune disorders [6, 7, 8].

**FIGURE 1:**
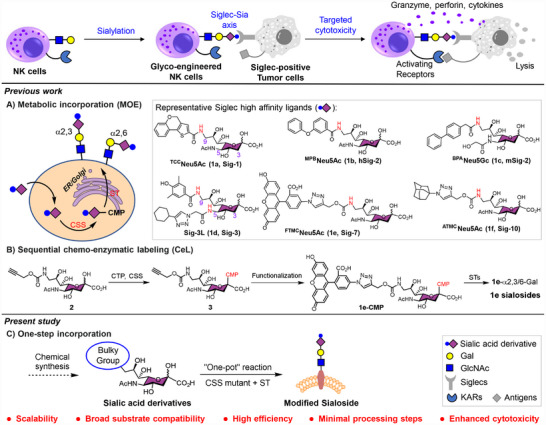
Generation of glyco‐engineered NK cells with enhanced anti‐tumor activity. Previous strategies: (A) Metabolic incorporation of Sia analogs through the endogenous pathway. Representative structures of reported Siglec ligands are shown. (B) Multi‐step chemo‐enzymatic synthesis involving transfer of CMP‐activated sialic acid analog (e.g., **1e‐CMP**) to surface acceptors by recombinant sialyltransferases (STs). Present strategy: (C) Streamlined one‐pot sialylation system featuring an engineered CMP‐sialic acid synthetase (CSS) coupled with sialyltransferases (STs) for single‐step glycan modification.

Beyond antibody‐based approaches, chemically‐modified sialoside ligands have been developed to enhance affinity and selectivity for Siglec recognition [9, 10, 11]. Several high‐affinity Sia analogs have been identified, including **1a** for Sig‐1 (Sialoadhesin, Sn, CD169), **1b** for human Sig‐2 (hCD22), **1c** for murine Sig‐2 (mCD22), **1d** for Sig‐3 (hCD33), **1e** for Sig‐7 and **1f** for Sig‐10/E (Figure 1A) [12, 13]. These synthetic ligands have enabled transformative applications across multiple therapeutic platforms. For example, **1b**‐, **1c**‐ and **1d**‐functionalized liposomes demonstrate precise targeting B‐cell acute lymphoblastic leukemia (B‐ALL) [14], suppress aberrant B‐cell activity [15], and modulate mast cell responses [16]; Glycopolymers bearing **1f** mediate microglia regulation through *cis*‐interactions with Sig‐E [17]; and **1c**‐ or **1d**‐modified proteins (e.g., toxins, anti‐IgD and anti‐IgE antibodies) were applied to selectively target murine B cells and mast cells [18, 19].

Glycoengineering approaches have revolutionized Siglec‐targeted therapies by enabling precise cell‐surface modification. Through metabolic oligosaccharide engineering (MOE) [20] and chemoenzymatic labeling (CeL) [21] strategies, high‐affinity ligands such as **1b** [22, 23] and **1e** [24] have been successfully incorporated onto natural killer (NK) cells, boosting their anti‐lymphoma efficacy to clinical‐stage development [25] (Figure 1A,B). Parallel developments in tumor cell glycoengineering demonstrate that **1a**‐modified cancer cells undergo enhanced macrophage‐mediated clearance via Sig‐1 engagement [26, 27].

Despite these advances, broader application of these strategies has been limited by challenges in synthesizing structurally diverse sialoside analogs, particularly those with bulky substitutions at C‐5 and/or C‐9 positions. A key example is the Siglec‐3 (Sig‐3)‐specific high‐affinity ligand **1d**‐sialoside, which bears modifications at both C‐5 and C‐9 but remains difficult to produce efficiently.

In vivo, sialylation proceeds through two key enzymatic steps: conversion of free sialic acid into its cytidine 5’‐monophosphate (CMP)‐activated intermediate (CMP‐Sia) by CMP‐Sia synthetases (CSSs), followed by transfer of the Sia residue to terminal galactose (Gal) acceptors by sialyltransferases (STs). Recombinant CSSs and STs have been widely employed in the enzymatic synthesis of natural sialosides as well as functional synthetic analogs [19, 28, 29, 30, 31, 32]. Previous studies have demonstrated that STs readily tolerate bulky substituents on sialic acids, efficiently transferring their CMP‐activated derivatives to glycoconjugate acceptors [19, 22, 23, 24, 33, 34, 35, 36]. In contrast, most wild‐type CSSs (wtCSSs) exhibit high activity only toward sialic acids bearing small chemical modifications (e.g., NH_2_, N_3_ or alkyne groups at the C‐5 or C‐9 position), but display markedly reduced efficiency with bulkier modifications. As a result, the preparation of sialoside analogs typically requires multi‐step workflows: enzymatic generation of CMP‐Neu5Ac bearing a small chemical handle, subsequent derivatizations (e.g., amidation or “click” chemistry) to introduce the bulky groups, and final transfer by STs to generate the desired sialosides (e.g., **1e**‐sialosides, Figure 1B) [22, 23, 24, 33, 34, 35]. In addition, CMP‐Sia derivatives are highly polar and labile under acidic or elevated temperature conditions, posing challenges for purification and often limiting material availability for downstream studies.

To expand the substrate scope of the CMP‐Sia synthetase from *N. meningitidis* (NmCSS), the most widely used CSS, several variants have been engineered to accommodate C5‐*N*‐acyl modifications such as benzyl and benzyloxycarbonyl groups [37, 38]. However, these mutants show minimal activity toward modifications at C‐9 position. Here, we report a one‐pot strategy for cell‐surface glycoengineering with high‐affinity Siglec ligands, in which CMP‐Sia derivatives are generated in situ by engineered NmCSS and directly transferred onto glycoconjugate acceptors by STs, thereby bypassing intermediate purification steps (Figure 1C). Through rational design, we broadened the substrate tolerance of NmCSS to include both C‐5‐ and C‐9‐modified Sia analogues. The catalytic activity of NmCSS mutants was systematically evaluated using a library of C‐5‐/C‐9‐substituted derivatives, which were efficiently transferred to LNnT (Galβ1–4GlcNAcβ1–3Galβ1–4Glc) acceptors by combination of α2,3‐/α2,6‐STs. As a dual‐purpose strategy, the resulting sialoside analogs were also immobilized on a glycan microarray to identify high‐affinity ligands for various Siglecs. Finally, we achieved single‐step engineering of NK‐92MI cells with high‐affinity ligands targeting Sig‐2 or Sig‐3, resulting in markedly enhanced cytotoxicity against Sig‐2^+^ or Sig‐3^+^ tumor cells.

## 2 Results and Discussion

### 2.1 Rationally Engineering NmCSS to Tolerate Bulky Group Modification at C‐9 of Sia

**FIGURE 2:**
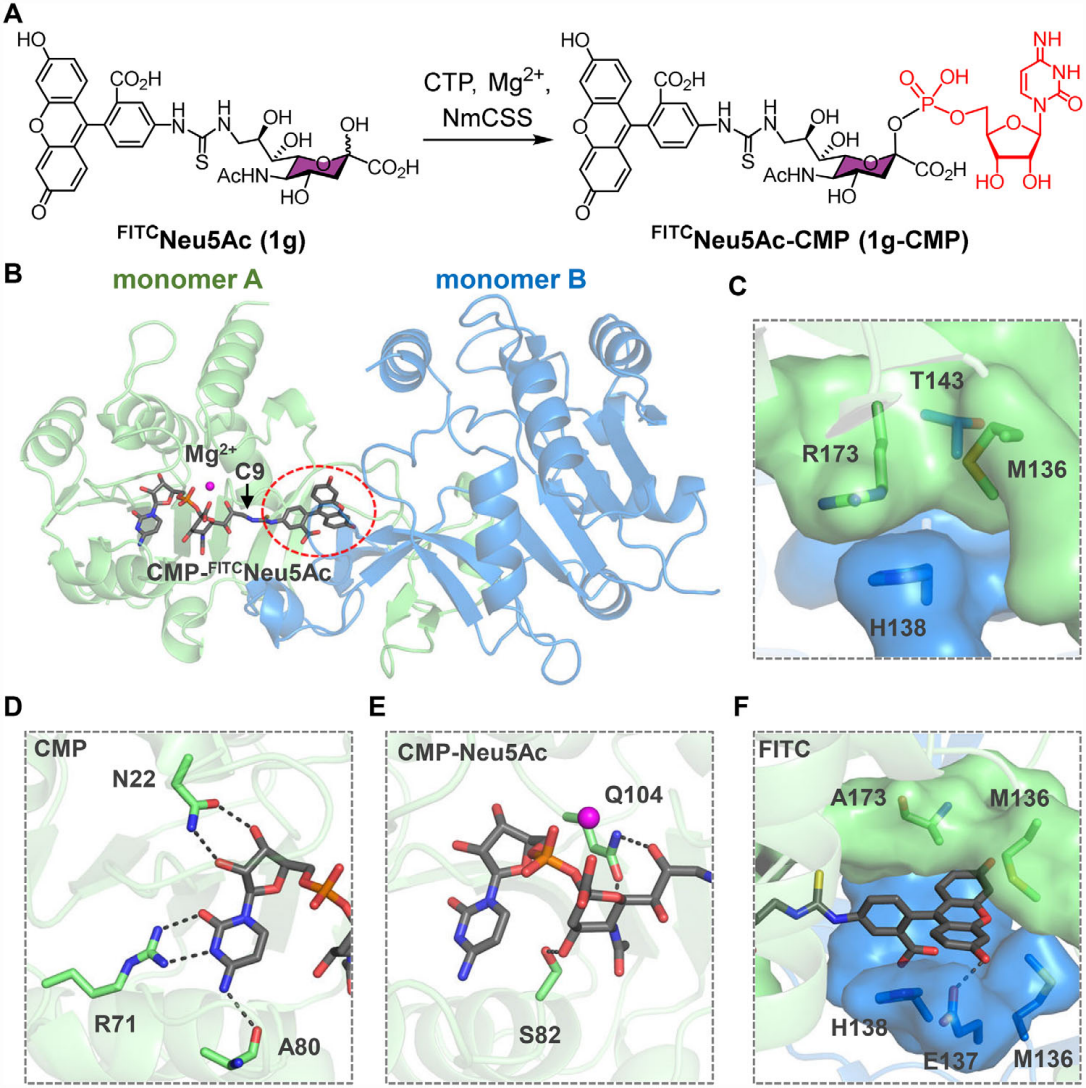
Engineering NmCSS for C‐9‐FITC‐modified Neu5Ac (**1** **g**) recognition. (A) Enzymatic conversion of ^FITC^Neu5Ac (**1** **g**) to ^FITC^Neu5Ac‐CMP (**1g‐CMP**). (B) Modeled **1g‐CMP**–NmCSS complex. The homodimer is represented as green and blue cartoons, with Mg^2+^ (magenta sphere) and **1g‐CMP** (gray sticks) shown in the active site. (C) Local structural environment surrounding the C‐9 substituent of Neu5Ac. (D–F) Interaction analysis between NmCSS R173A and distinct moieties of **1g‐CMP**: (D) CMP, (E) CMP‐Neu5Ac, and (F) FITC group. Hydrogen bonds are indicated by black dashed lines.

Fluorescein‐modified Neu5Ac (^FTMC^Neu5Ac, **1e**) has been identified as a high‐affinity ligand for Sig‐7 and widely applied to probe its biological roles in vitro and in vivo. However, wtNmCSS exhibits poor catalytic activity toward **1e**, likely due to steric hindrance from the bulky fluorescein substitution at the C‐9 position. To enhance NmCSS tolerance toward C‐9 modifications, we employed the more accessible ^FITC^Neu5Ac (**1** **g**) as a model substrate and constructed a **1g‐CMP**‐bound NmCSS complex for structural analyses (Figure 2). The resulting model suggested that the FITC group inserts into a pocket formed at the dimer interface, involving residues M136, T143 and R173 from monomer A, and E137 and H138 from monomer B. Pronounced steric clashes were observed between the FITC moiety and these residues, with R173 and H138 forming a stable cation–π interaction that prevents extension of FITC group along the binding channel (Figure 2C). These observations guided the selection of these five interface residues for subsequent site‐directed mutagenesis.

### 2.2 Catalytic Characterization of NmCSS Mutants

**FIGURE 3:**
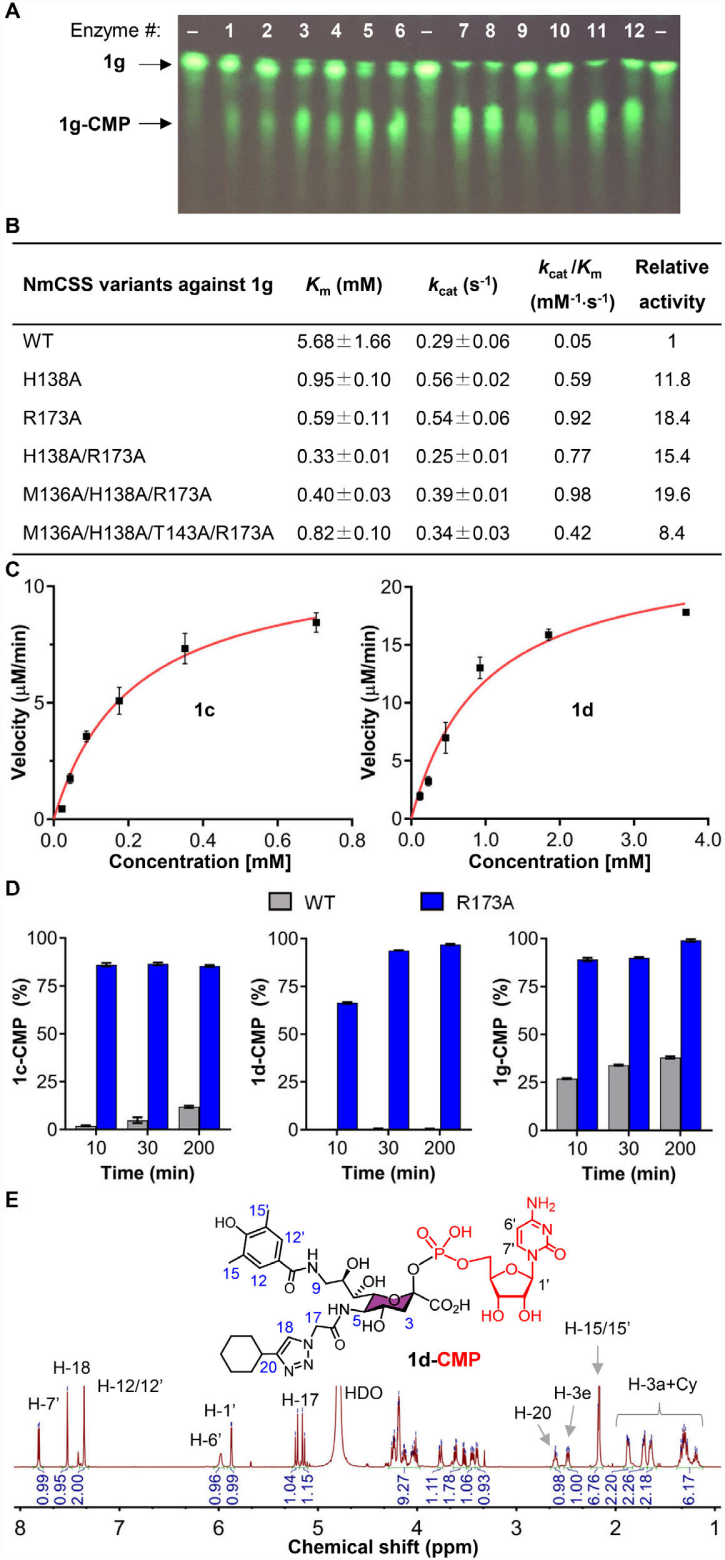
Catalytic activity of NmCSS variants. (A) Thin‐layer chromatography (TLC) analysis of NmCSS variant activity toward ^FITC^Neu5Ac (**1** **g**). Lanes #1–#12: wild type, M136A, H138A, T143A, R173A, H138A/R173A, M136A/H138A/R173A, M136A/H138A/T143A/R173A, M136G/E137G/H138G/H139G/R173A, M136G/E137G/H138‐/H139‐/R173A, E137R/H138G/R173A, E137R/H138G/H139‐/R173A. (B) Kinetic characterization of NmCSS variants using ^FITC^Neu5Ac (**1** **g**) as a substrate. (C) Kinetic parameter determination of the R173A mutant with ^BPA^Neu5Gc (**1c**) and CD33L (**1d**) as substrates. (D) Catalytic efficiency comparison between wild‐type (WT) NmCSS and the R173A mutant for **1c**, **1d** and **1** **g**. CMP‐activated products were quantified by RP‐HPLC at specified time points. Error bars represent the standard deviation from triplicate experiments. (E) Chemical structure and ^1^H NMR of enzymatically synthesized **1d**‐CMP via NmCSS R173A mutant. Cy represents the cyclohexyl group.

To assess the functional contribution of the identified interface residues to **1** **g** recognition, we performed site‐directed mutagenesis at these positions. A total of eleven NmCSS variants were generated and overexpressed in *E. Coli*, including four single‐point mutants and seven combinational mutants (Table S1). Their catalytic activities toward **1** **g** were evaluated by incubating each variant with **1** **g** and cytidine triphosphate (CTP), followed by thin‐layer chromatography (TLC) analysis (Figure 3A). As predicted by structural modeling, wtNmCSS displayed weak activity toward **1** **g** (lane 1). Among the single‐point variants (Met136, His138, Thr143 or Arg173; lanes 2–5), R173A exhibited the most pronounced enhancement (lane 5), indicating that R173 acts as a key gatekeeper residue (Figure 2C). Introduction of additional mutations to R173A, including H138A/R173A, M136A/H138A/R173A, M136A/H138A/T143A/R173A, E137R/H138G/173A and E137R/H138G/H139‐/173A (lanes 6–8, 11, and 12), led to further improvements in catalytic activity. In contrast, extensive modifications within the M136–H139 loop, in combination with the R173 substitution, abolished activity (lanes 9 and 10).

To further evaluate the catalytic performance of NmCSS variants, apparent kinetic parameters were measured using the malachite green colorimetric assay (Figure 3B; Figure S1A) [39]. The results demonstrated that the NmCSS mutants exhibited significantly improved binding affinity for **1** **g**, as reflected by reduced *K*_m_ values, whereas displaying minimum impacts on the enzyme turnover (*k*_cat_). Among them, single‐point mutants H138A and R173A showed 11.8‐ and 18.4‐fold higher catalytic efficiency (*k*_cat_/*K*_m_), respectively, relative to wild type. The triple mutant M136A/H138A/R173A was more active than the R173A mutant, with a 19.6‐fold higher relative activity compared to the wild type enzyme, consistent with TLC analysis (Figure 3A, lane 7).

As shown in Figure S1B, kinetic characterization revealed that most NmCSS variants maintained comparable catalytic efficiency (*k*_cat_/*K*_m_) toward unmodified Neu5Ac compared to wild‐type enzyme, except that H138A/R173A double mutant exhibited 12.4‐fold reduction in activity, while T143A single mutant demonstrated 4.9‐fold enhancement. Based on a comprehensive evaluation of both protein expression levels and catalytic performance, the R173A mutant was used for subsequent studies.

To gain mechanistic insights into **1** **g** recognition, we constructed a **1g‐CMP**‐NmCSS R173A complex and subjected it to energy minimization (Figure 2D,E). The resulting model showed that the CMP and Neu5Ac moieties established canonical interactions with key NmCSS residues (N22, R71, A80, S82, and Q104), consistent with the reported crystal structure [40]. Importantly, the FITC substituent could be well accommodated in a pocket formed at the dimer interface (Figure 2F).

We next assessed the catalytic activity of R173A mutant toward C‐9‐*N*‐biphenyl‐4‐acetamide (BPA) Neu5Gc [41] (**1c**, murine Sig‐2/mCD22 ligand) and Sig‐3L [42] (**1d**, human Sig‐3/hCD33 ligand), both poor substrates for wtNmCSS [19]. Strikingly, R173A showed a 46.3‐fold improvement in catalytic efficiency for **1c** relative to wild type (*k*_cat_/*K*_m_ = 3.7 vs. 0.08 mM^−1^s^−1^; Figure 3C; Figure S1C). Even more strikingly, while wtNmCSS activity toward **1d** was undetectable, R173A achieved a measurable *k*_cat_/*K*_m_ of 0.21 mM^−1^s^−1^ (Figure 3C; Figure S1D). Time‐course RP‐HPLC (reversed phase high‐performance liquid chromatography) assays further demonstrated that R173A efficiently generated **1c**‐CMP, **1d**‐CMP, and **1g**‐CMP, in stark contrast to wtNmCSS (Figure 3D). Structural characterization of the purified CMP‐sialosides by nuclear magnetic resonance (NMR) spectroscopy and mass spectrometry (MS) confirmed the presence of the benzamide and cyclohexyl (Cy) moieties in **1d**‐CMP (Figure 3E), biphenylacetyl group in **1c**‐CMP and FITC modification in **1g**‐CMP (see supplementary file). These results collectively demonstrate that the R173A mutation successfully overcomes the steric constraints that limit wtNmCSS activity toward bulky sialic acid analogs.

Although previous mutagenesis at positions F192/F193 demonstrated modest improvements in accommodating C‐5 modifications [38], our findings establish R173A as a robust NmCSS variant with dramatically expanded substrate promiscuity, capable of efficiently processing Sia analogs bearing bulky substitutions at both C‐5 and C‐9 positions. Molecular modeling of the **1d**‐**CMP** complexes with both wild‐type and R173A NmCSS revealed striking conformational changes induced by the mutation (Figure S2). Structural analysis demonstrated that replacing arginine with alanine at position 173 substantially increased the binding cavity length from 8.2 to 20 Å while moderately expanding the channel bottleneck from 2.4 to 2.7 Å (Figure S2F–H). The elongated cavity readily accommodates the C‐9‐substituted group oriented toward the deep pocket interior near the 173 residue, while the widened entry simultaneously permits accommodation of the C‐5 modification positioned at the cavity entrance (Figure S2B–E). These structural changes collectively explain the mutant enzyme's enhanced ability to process both C‐5‐ and C‐9‐modified sialic acid derivatives.

### 2.3 Synthesis of Sialoside Analogs via a “One‐Pot Two‐Enzyme” (OPTE) Approach

**FIGURE 4:**
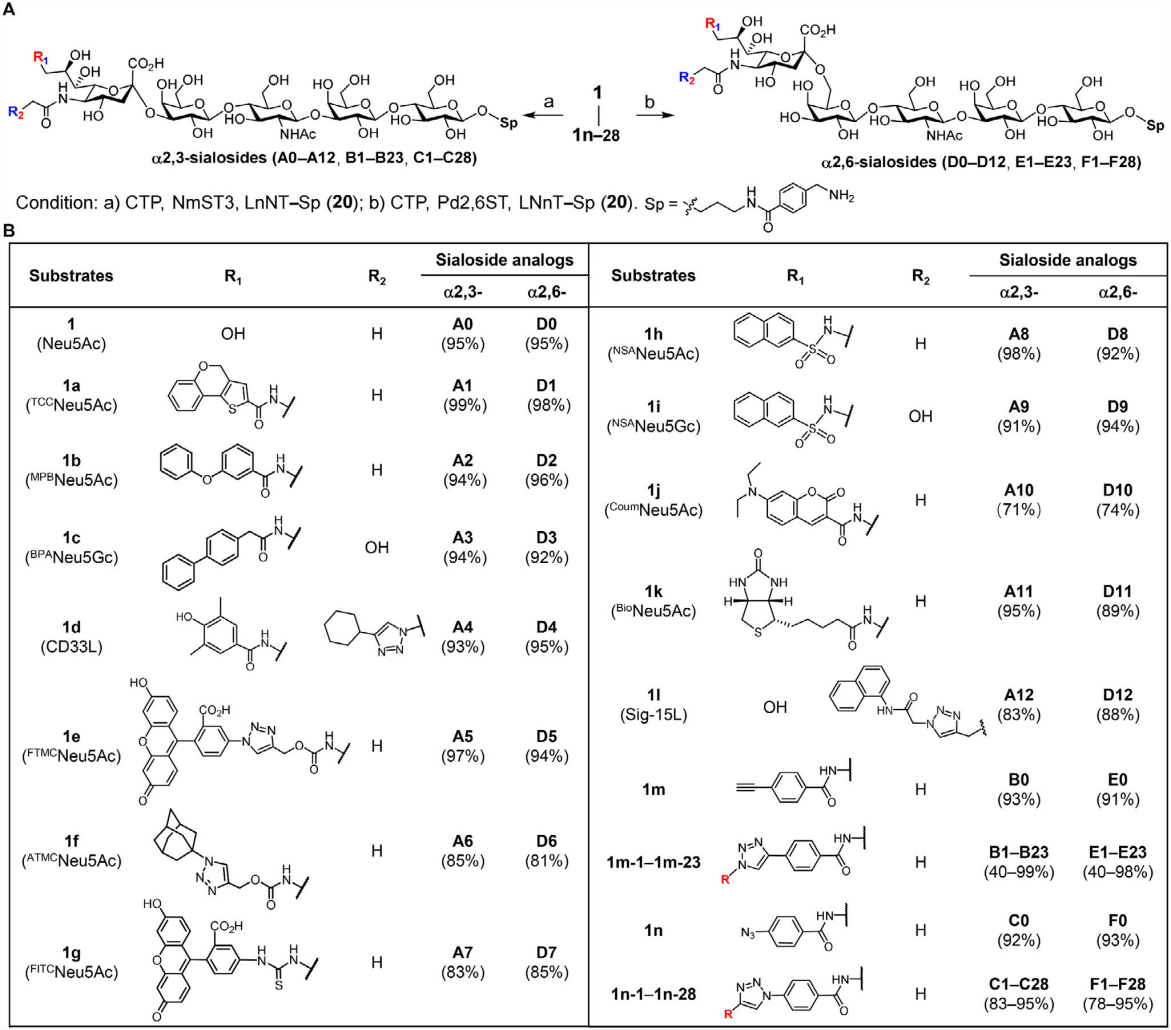
Chemoenzymatic synthesis of sialosides modified with large groups via a “one‐pot” enzymatic process. (A) Enzymatic transfer of sialic acid derivatives to LNnT in either α2,3‐ or α2,6‐linkages. (B) Modification of sialic acid at C‐5 or/and C‐9 positions via amide bond formation or click chemistry. Among them, compounds **1a**–**1i** and **1l** are previously established Siglec high‐affinity ligands. Compounds **1m‐1**–**1m‐23** and **1n‐1**–**1n‐28** (see Tables S2 and S3) were synthesized via click chemistry.

To investigate the substrate scope of the NmCSS R173A mutant, we chemically synthesized a panel of C‐5/C‐9‐amide‐substituted Neu5Ac/Neu5Gc derivatives (Figure 4; Scheme S1), including several high‐affinity Siglec ligands [12, 43, 44] (**1a–1i** and **1l** for Sig‐1, hSig‐2, mSig‐2, hSig‐3, Sig‐7, Sig‐10, Sig‐E, Sig‐F and Sig‐15), as well as coumarin (**1j**) and biotin (**1k**) conjugates. Notably, **1d** carries dual modifications at both C‐5 and C‐9, while **1l** features a single C‐5 substitution (synthetic route outlined in Schemes S2 and S3, respectively). Upon incubation with NmCSS R173A mutant in the presence of either the α2,3‐sialyltransferase NmST3 [45] or α2,6‐sialyltransferase Pd2,6ST [46] in one pot (Figure 4A), all derivatives (**1a**–**1l**) were successfully transferred to the enzymatically prepared tetrasaccharide LNnT (**20**, Scheme S4), affording the corresponding α2,3‐ or α2,6‐linked sialoside analogs (**A1**–**A12** and **D1**–**D12**, respectively; Figure 4B) in good to excellent yields. All products were purified by RP‐HPLC and structurally validated by NMR spectroscopic and MS analyses (see supplementary file).

Encouraged by these results, we further evaluated the tolerance of the NmCSS R173A mutant toward more diverse bulky substituents. To this end, 9‐*N*‐(4‐Ethynyl)benzoyl‐Neu5Ac (**1m**) and 9‐*N*‐(4‐azido‐)benzoyl‐Neu5Ac (**1n**) were chemically synthesized and subjected to copper‐catalyzed alkyne‐azide cycloaddition (CuAAC) with 23 azides and 28 alkynes, respectively (Scheme S5). The resulting conjugates (**1m‐1**–**1m‐23** and **1n‐1**–**1n‐28**) were subsequently transferred to LNnT, **20** using our OPTE system, generating a comprehensive library of the C‐9‐modified sialosides (**B1**–**B23** and **C1**–**C28** with α2,3‐linkages; **E1**–**E23** and **F1**–**F28** with α2,6‐linkages) in 40%–99% yields (Figure 4B; Tables S2 and S3).

Together, these findings demonstrate that the NmCSS R173A mutant readily accommodates bulky substitutions at the C‐5 and/or C‐9 positions of Neu5Ac/Neu5Gc, enabling a streamlined and scalable single‐step synthesis of structurally diverse sialoside analogs through our OPTE strategy–eliminating the need for intermediate purification while maintaining high product yields.

### 2.4 Analysis of Siglec Binding Specificity Using the Sialoside Analog Microarray

**FIGURE 5:**
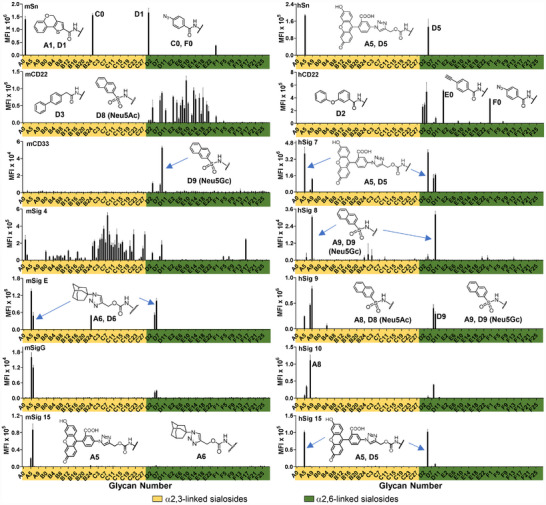
Glycan microarray analysis of recombinant murine (left panels) and human (right panels) Siglec binding specificity for sialoside analogs. Columns represent background subtracted average fluorescence intensities of the four median values out of six replicates with standard error bars. Note: compounds **A4** and **D4** were not included in the array.

Glycan microarray technology has been extensively employed to determine the specificity of glycan‐binding proteins [47, 48]. Thus, the synthetic sialoside analogs were immobilized on *N*‐hydroxysuccinimide (NHS)‐activated glass slides via the reducing end amine group and screened against 14 fluorescently labeled recombinant Siglec‐Fc chimeras, including seven human (hSig) and seven murine (mSig) Siglecs (Figure 5). The resulting microarray contained 132 compounds, comprising two natural sialosides and 130 synthetic analogs in either α2,3‐ or α2,6‐linkages (Figure 4B; Tables S2 and S3).

As expected, each Siglec showed strong binding to its previously reported high‐affinity ligands. For instance, mSig‐1 (mSn) bound to both α2,3‐ and α2,6‐linked C‐9‐*N*‐(4*H*‐thieno[3,2‐*c*]chromene‐2‐carbamoyl) (TCC)‐sialosides [49] (**A1** and **D1**), while hCD22 and mCD22 recognized α2,6‐linked C‐9‐*N*‐m‐phenoxybenzamide (MPB)‐sialoside [42] **D2** and BPA‐sialoside [41] **D3**, respectively. Interestingly, expanded binding repertoire was observed for mSig‐4 and mCD22, which uniquely interacted with multiple C‐series and E‐series compounds, respectively – a property not shared by other family members, suggesting these scaffolds may serve as selective ligands for further development. Notably, scaffolds **E0** and **F0** [50] were recognized by hCD22, but subsequent cycloaddition reactions abolished binding affinity. A similar trend was observed for mSn, which bound to **C0** and **F0** but not their cycloaddition products. This scaffold‐dependent recognition pattern provides critical insights for rational ligand design, with **E0** emerging as a uniquely specific hCD22 ligand within this screening platform.

Both α2,3‐ and α2,6‐linked FTMC‐sialosides (**A5** and **D5**), originally identified as high‐affinity ligands for hSig‐7 [51], also bound unexpectedly to hSn, hSig‐15, mSig‐E and mSig‐G. Similarly, the α2,3‐linked 2‐Naphthyl‐sulfonylamide (NSA)–Neu5Ac sialoside (**A8**), initially reported as a mSig‐F ligand [43], exhibited cross‐reactivity with hSig‐9 and ‐10 on our arrays. Because murine Siglecs preferentially bind Neu5Gc‐sialosides [41], we prepared ^NSA^Neu5Gc (**1i**) and enzymatically transferred it to LNnT, generating α2,3‐ and α2,6‐linked sialosides (**A9** and **D9**, respectively). Surprisingly, both **A9** and **D9** were recognized by hSig‐7, ‐8 and ‐9, with mCD22 and mCD33 also binding tightly to **D9**. These results suggested that FTMC‐ and NSA‐modified sialosides may serve as broad‐spectrum ligands capable of targeting multiple Siglecs.

Importantly, this is the first study in which all reported high‐affinity Siglec ligands (e.g., **A1**, **A6**, **A8**‐**A10**, **D1**‐**D3** and **D5**) have been immobilized on the same glycan microarray for side‐by‐side comparison. Our findings reveal that many of these analogs lack sufficient selectivity for probing Siglec biology, underscoring the need to identify more discriminating ligands for therapeutic and mechanistic studies. This challenge can be addressed with our OPTE synthesis strategy, enabling the construction of a large sialoside analog library for systematic analysis of their Siglec binding affinity and selectivity on the same microarray.

### 2.5 One‐Step Glycoengineering of CHO‐Lec2 Cells with High Affinity Siglec‐Ligands

Because of the intracellular nucleotide‐sugar interconversion pathway and the presence of multiple endogenous sialyltransferase isoforms, Sia analogs incorporated by MOE are often displayed on the cell surface in heterogeneous structures and various linkages (α2,3/6/8‐) [20]. This heterogeneity often leads to low labeling efficiency and potential off‐target effects. As an alternative, recombinant STs can directly transfer CMP‐activated Sia analogs onto cell surfaces, thereby generating structurally defined glycan epitopes. While the Wu [23, 24] and Boons [33, 34] laboratories have demonstrated the enzymatic transfer of Sias bearing bulky substituents to cell surfaces, their approaches required purification of the labile CMP‐Sia intermediates, limiting broader applications.

**FIGURE 6:**
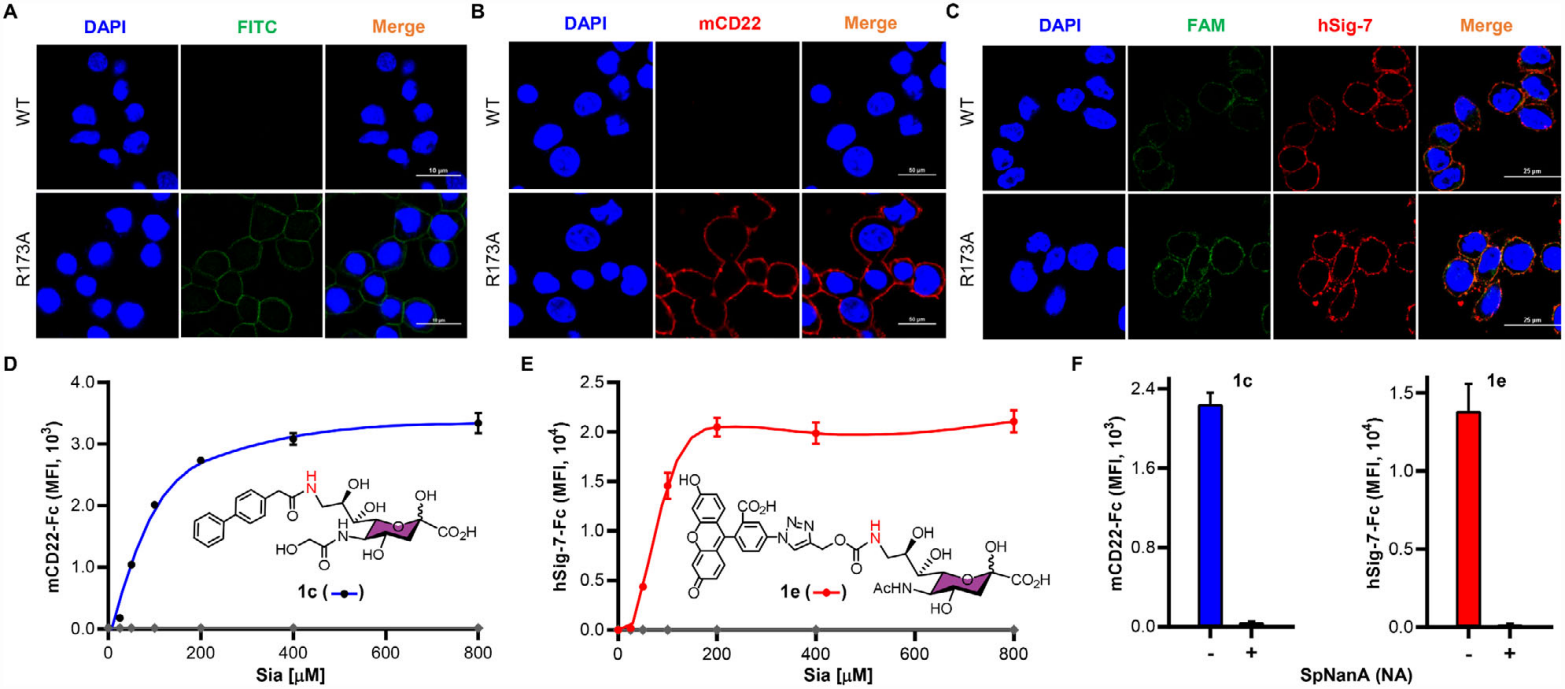
One‐step glycoengineering of CHO‐Lec2 cells with synthetic sialic acid derivatives. (A–C) Confocal microscopy analysis of CHO‐Lec2 cells engineered with sialic acid derivatives **1** **g**, **1c** (mCD22 ligand) and **1e** (hSig‐7 ligand), respectively, using either wtNmCSS or its R173A mutant in combination with the bacterial sialyltransferase Pd2,6ST. (D–E) Flow cytometry quantification of cell‐surface presentation of Neu5Ac (gray), **1c** (blue), and **1e** (red) across a concentration gradient (25–800 µM) using NmCSS R173A (0.5 mg/mL) and Pd2,6ST (20 µg/mL), detected by recombinant mCD22‐Fc or hSig‐7‐Fc chimeras, respectively. (F) Flow cytometry validation of surface‐displayed **1c** and **1e** (engineered at 400 µM) after treatment with (+) or without (‐) SpNanA sialidase (NA). Data represent mean fluorescence intensity (MFI) ± SD from three independent experiments.

To overcome this challenge, we employed our OPTE strategy, which combines the NmCSS R173A mutant with sialyltransferases, enabling direct incorporation of desired Siglec ligands onto cell surfaces in a single step. As a proof of concept, ^FITC^Neu5Ac (**1** **g**) was activated in situ with NmCSS R173A and then transferred by Pd2,6ST to Chinese Hamster Ovary (CHO)‐Lec2 cells, a model cell line that displays terminal galactose residues but lacks Sia modification due to an inactive CMP‐Sia transporter [52]. Confocal microscopy confirmed robust presentation of **1** **g** on the cell surface (Figure 6A). In contrast, cells treated with wtNmCSS and Pd2,6ST showed no detectable fluorescein signal, consistent with our in vitro enzyme activity assays (Figure 3).

We next examined the feasibility of installing ^BPA^Neu5Gc (**1c**, mCD22 ligand) and ^FTMC^Neu5Ac (**1e**, hSig‐7 ligand). Staining with mCD22‐Fc chimeras revealed that R173A, but not wtNmCSS, efficiently incorporated α2,6‐linked **1c** onto cell surfaces (Figure 6B). Although wtNmCSS exhibited some activity toward **1e**, R173A was more efficient, leading to stronger fluorescein (FAM) fluorescence and robust binding of hSig‐7‐Fc (Figure 6C). Flow cytometry further demonstrated concentration‐dependent incorporation of high‐affinity Siglec ligands, with fluorescence signals reaching saturation at 200–400 µM of Sia analogs (Figure 6D,E). In contrast, Neu5Ac‐engineered cells showed negligible Siglec‐Fc binding, consistent with the lower affinity of natural Sia. Importantly, treatment with the sialidase SpNanA from *S. pneumoniae* (NA) abolished Siglec‐Fc staining (Figure 6F), confirming that the observed interactions were dependent on the installed high‐affinity ligands.

Altogether, these results demonstrate that CHO‐Lec2 cells can be glycoengineered in a single step to display high affinity Siglec‐ligands.

### 2.6 Targeting hCD22^+^ Tumor Cells by hCD22‐Ligand–Engineered NK‐92MI Cells

**FIGURE 7:**
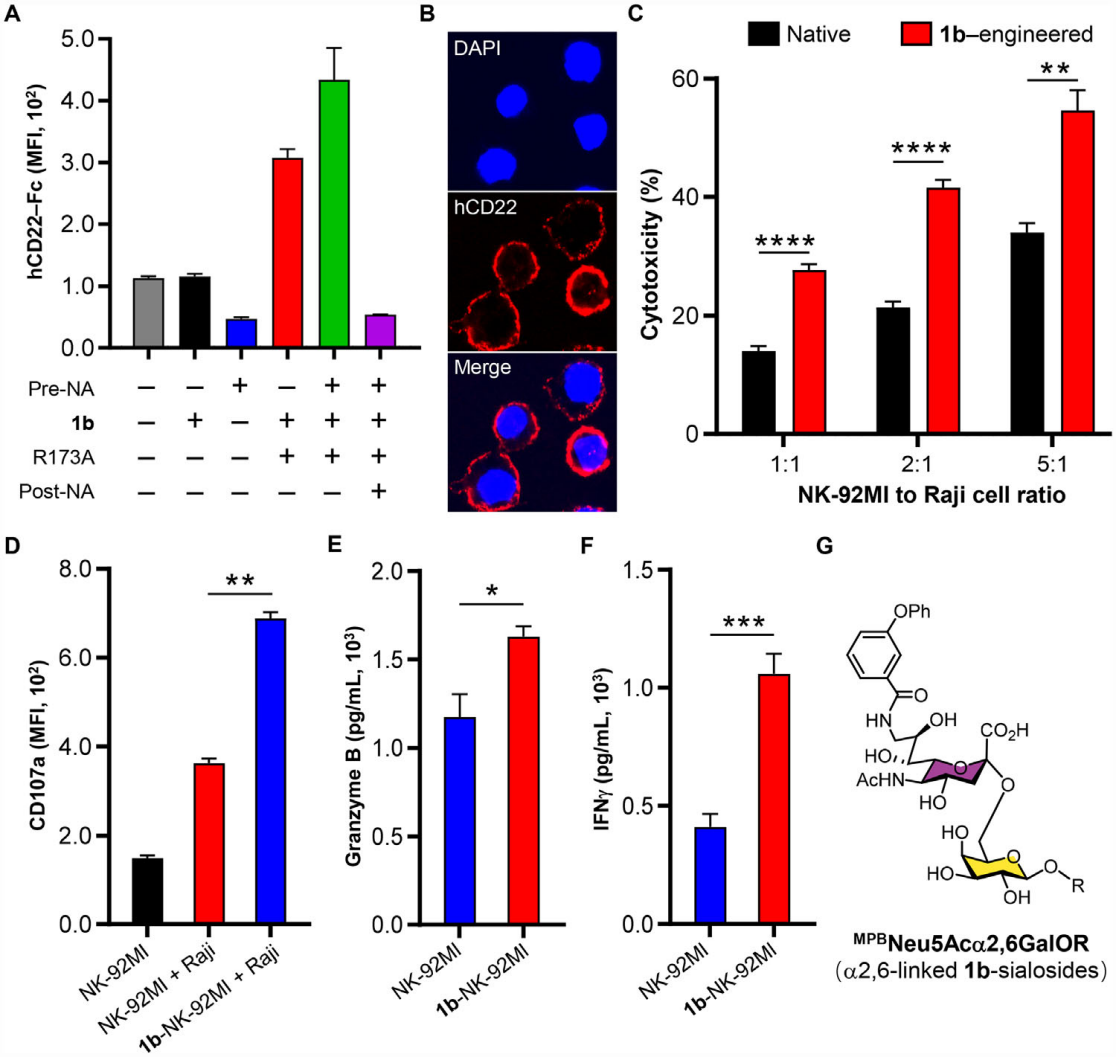
Enhanced cytotoxic activity of ^MPB^Neu5Ac (**1b**)‐engineered NK‐92MI cells against hCD22‐expressing Raji Cells. (A) Flow cytometric analysis of **1b**‐NK‐92MI cells, stained with hCD22‐Fc chimera. (B) Confocal microscopy analysis of **1b**‐NK‐92MI cells showing membrane‐localized **1b**‐sialosides (red) and DAPI‐stained nuclei (blue). (C) Cytotoxicity of **1b**‐NK‐92MI cells against Raji cells, measured by propidium iodide (PI) staining at indicated E/T ratios. (D–F) Activation profiling of naive vs. **1b**‐NK‐92MI cells upon Raji cell stimulation (E/T = 5). CD107a surface exposure, granzyme B release and IFNγ secretion were determined. Data represent mean fluorescence intensity (MFI) ± SD from three independent experiments. (G) Chemical structure of α2,6‐linked **1b**‐modified cell surface glycoconjugates, where R represents endogenous glycoproteins or glycolipids. The significance was analyzed with the two‐sided *t*‐test. (^*^) *p* ≤ 0.05; (^**^) *p* ≤ 0.01; (^***^) *p* ≤ 0.001.

To extend this one‐pot strategy toward immunotherapy, we glycoengineered human NK‐92MI cells, a widely used preclinical NK cell line [53]. Thus, ^MPB^Neu5Ac (**1b**, hCD22 ligand) was enzymatically activated in situ by NmCSS R173A and subsequently transferred to NK‐92MI cell surfaces via Pd2,6ST. Quantitative analysis demonstrated concentration‐dependent incorporation of **1b**, as evidenced by both flow cytometry and confocal microscopy (Figure 7A–B; Figure S3A). Pretreatment with SpNanA (NA) exposed additional galactose acceptors, enhancing **1b** incorporation and hCD22‐Fc binding, whereas post‐treatment with SpNanA cleaved the installed ligand and completely abolished binding (Figure 7A; Figure S3B), confirming the **1b**‐dependent binding.

Although hCD22 (hSig‐2) is expressed in nearly all B‐cell malignancies [54], no CD22‐targeted therapy has yet been approved. We therefore evaluated the therapeutic potential of **1b**‐NK‐92MI cells against hCD22^+^ Raji B lymphoma cells. Compared to unmodified counterparts, **1b**‐NK‐92MI cells exhibited significantly enhanced cytotoxicity against CD22+ Raji cells in a dose‐dependent manner, achieving 55% specific lysis at a 5:1 effector‐to‐target (E/T) ratio and representing a 62% improvement over naïve control cells (Figure 7C). This enhanced cytotoxicity was mechanistically linked to an approximately 2‐fold increase in CD107a expression (Figure 7D), along with significantly elevated secretion of granzyme B and interferon (IFN)‐γ (Figure 7E,F), consistent with robust NK cell activation.

**FIGURE 8:**
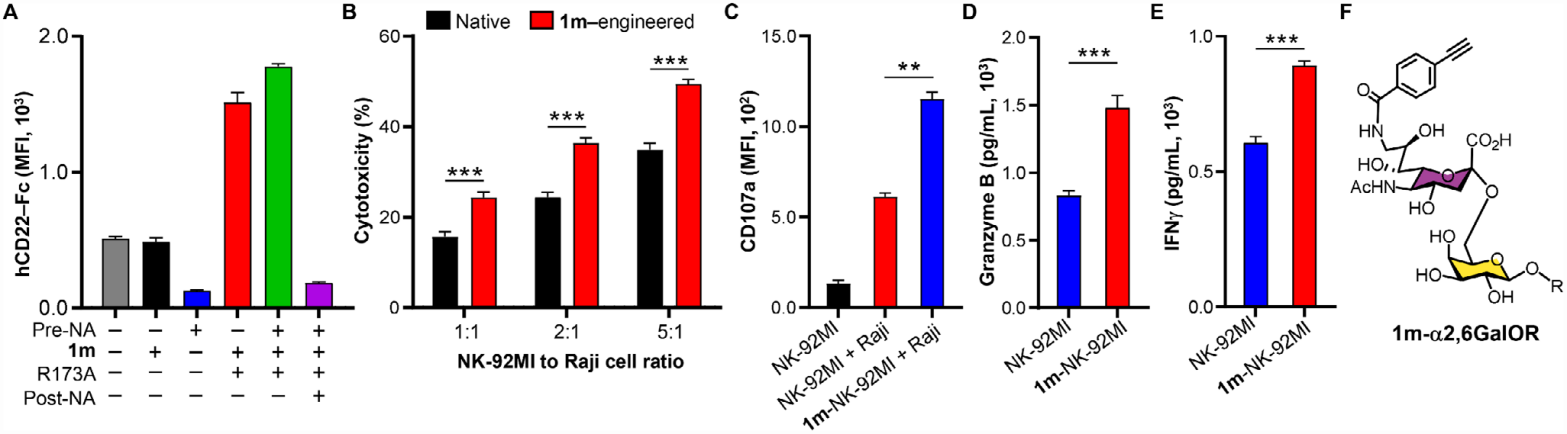
Targeting Raji Cells by **1m**‐engineered NK‐92MI cells. (A) Flow cytometric analysis of **1m**‐NK‐92MI cells, stained with hCD22‐Fc chimera. (B) Cytotoxicity of **1m**‐NK‐92MI cells against Raji cells, measured by propidium iodide (PI) staining at indicated E/T ratios. (C–E) Activation profiling of native vs. **1b**‐NK‐92MI cells upon Raji cell stimulation (E/T = 5). CD107a surface exposure, granzyme B release and IFNγ secretion was determined. Data represent mean fluorescence intensity (MFI) ± SD from three independent experiments. (F) Chemical structure of α2,6‐linked **1m**‐modified cell surface glycoconjugates, where R represents endogenous glycoproteins or glycolipids. The significance was analyzed with the two‐sided *t*‐test. (^*^) *p* ≤ 0.05; (^**^) *p* ≤ 0.01; (^***^) *p* ≤ 0.001.

Building upon these findings with the known **1b** ligand, we next evaluated our newly discovered **E0** (α2,6‐linked **1m**) ligand, which exhibited exceptional specificity for hCD22 without cross‐reactivity to other Siglec family members (Figure 5). Employing the same enzymatic engineering approach, we achieved efficient surface display of **1m** on NK‐92MI cells at 400 µM substrate concentration, with binding characteristics similarly enhanced by NA pretreatment and abolished by subsequent sialidase treatment (Figure 8A). In functional assays, **1m**‐engineered NK cells demonstrated superior cytotoxicity against Raji cells, reaching 50% specific lysis at a 5:1 E/T ratio (40% increase vs. controls; Figure 8B), accompanied by enhanced activation markers and effector molecule secretion (Figure 8C–E).

These results demonstrate that hCD22‐ligand–engineered NK‐92MI cells selectively recognize and kill hCD22^+^ tumor cells through enhanced degranulation and cytokine release. Importantly, because CD22 expression is independent of CD19, this strategy provides a valuable therapeutic alternative for CD19‐negative B‐cell malignancies that evade current CD19‐directed therapies [55].

The success of both engineering strategies highlights how precise glycan remodeling can convert naturally immunosuppressive Siglec interactions into potent tumor recognition mechanisms. Importantly, the combination of ligand engineering with prior desialylation created a synergistic enhancement of NK cell function, simultaneously amplifying activating signals through the 2B4 [56] and NKG2D [57] pathways while relieving inhibitory Siglec‐7/‐9 signaling. This multi‐modal approach not only improved target cell recognition through CD22 engagement but also promoted more stable immunological synapse formation, ultimately maximizing cytotoxic potential (Figure S3C). Accordingly, all subsequent glycoengineering experiments were performed using pre‐desialylated NK‐92MI cells as the cellular platform.

### 2.7 Targeting hCD33^+^ Tumor Cells by hCD33‐Ligand Engineered NK‐92MI Cells

CD33 (Sig‐3) is expressed in >90% of acute myeloid leukemia (AML) tumors [58], and serves as a major target for CD33‐directed therapies, including antibody–drug conjugates (e.g., commercialized gemtuzumab ozogamicin) and chimeric antigen receptor (CAR) T‐cell therapies [59]. Therefore, we sought to evaluate whether hCD33‐ligand (**1d**)‐engineered NK‐92MI cells could target CD33^+^ AML cells.

**FIGURE 9:**
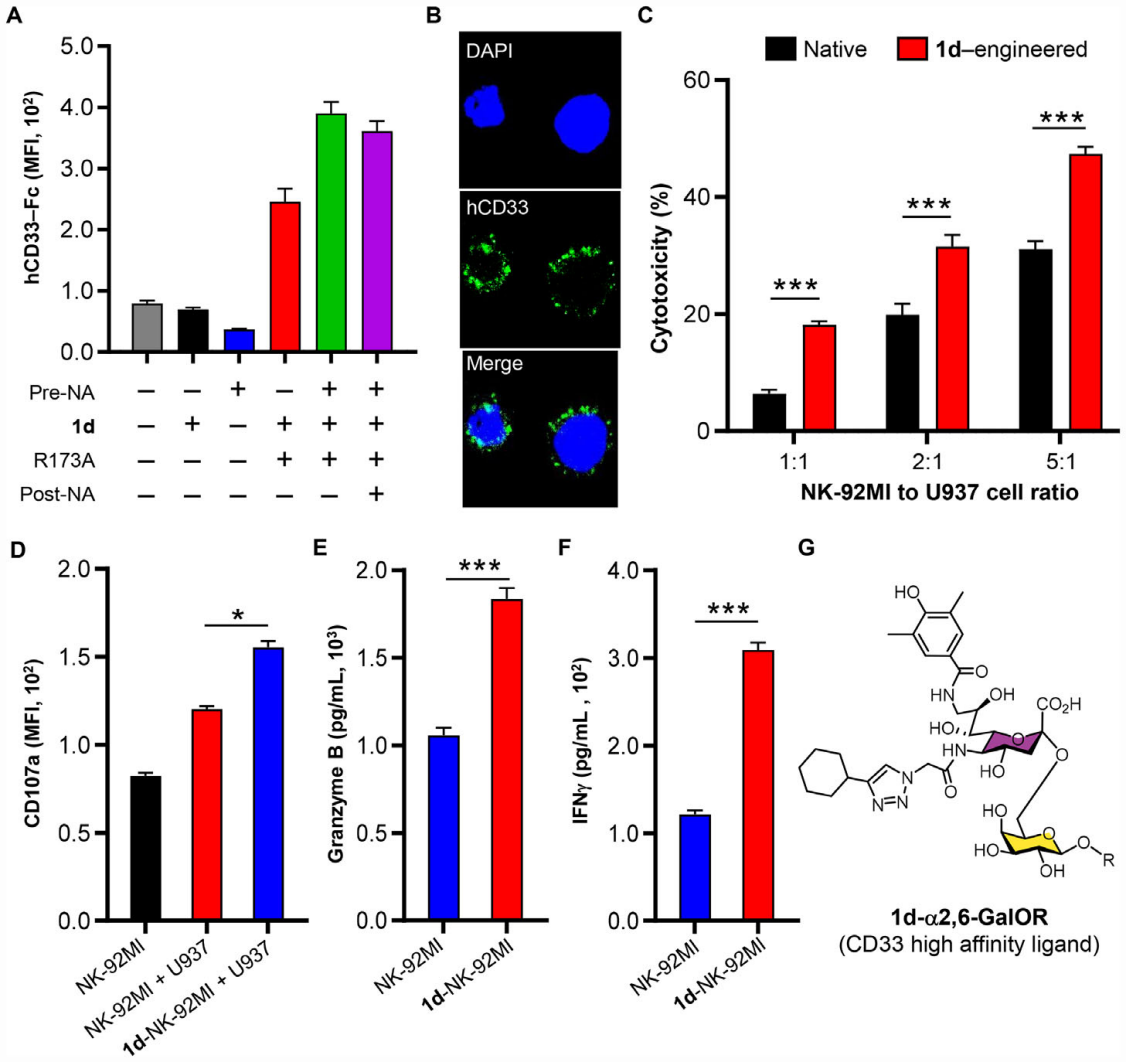
Enhanced cytotoxic activity of hCD33 ligand (**1d**)‐engineered NK‐92MI cells against hCD33^+^ U937 Cells. (A) Flow cytometric analysis of **1d**‐NK‐92MI cells remodeled via one‐pot enzymatic sialylation, stained with hCD33‐Fc chimera. (B) Confocal microscopy analysis of **1d**‐NK‐92MI cells showing membrane‐localized **1d**‐sialosides (green) and DAPI‐stained nuclei (blue). (C) Cytotoxicity of **1d**‐NK‐92MI cells against U937 cells, measured by PI staining at indicated E/T ratios. (D–F) Activation profiling of native vs. **1d**‐NK‐92MI cells upon U937 cell stimulation (E/T = 5). CD107a surface exposure, granzyme B and IFNγ secretion was determined. Data represent mean fluorescence intensity (MFI) ± SD from three independent experiments. (G) Chemical structure of α2,6‐linked **1d**‐modified cell surface glycoconjugates, where R represents endogenous glycoproteins or glycolipids.

Analog **1d** was activated in situ by NmCSS R173A, then directly transferred to NK‐92MI cells with Pd2,6ST in one step. Flow cytometry and confocal imaging showed successful installation of **1d**, with binding signals saturating at ∼400 µM (Figure 9A,B; Figure S4A). Similar to compound **1b**‐engineering scenario, pretreatment with SpNanA promoted **1d** incorporation and enhanced hCD33‐Fc binding. In contrast, post‐treatment failed to cleave the installed **1d**, suggesting that the bi‐substituted Neu5Ac analog is not a favorable substrate for SpNanA (Figure 9A; Figure S4B). In cytotoxicity assays, **1d**‐engineered NK‐92MI cells showed significantly higher killing of U937 AML cells at variable E/T ratios, with pre‐desialylated cells exhibiting the strongest effects (Figure 9C; Figure S4C). Stimulation also led to increased CD107a expression and elevated secretion of granzyme B and IFNγ (Figure 9D–F).

Importantly, compared with unmodified counterparts, both **1b**‐ and **1d**‐engineered NK‐92MI cells showed minimal cytotoxicity against Jurkat cells (hCD22^–^/hCD33^–^), confirming low non‐specific killing (Figures S3D and S4D). Furthermore, engineered NK‐92MI cells maintained normal viability and proliferation up to 800 µM ligand treatment and four days post‐incubation (Figure S5). These results confirmed that glycoengineered NK‐92MI cells preserved normal proliferative capacity and exceptional target discrimination, establishing the therapeutic precision of this approach.

## 3 Conclusion

Through computational modeling and rational mutagenesis, we have identified critical residues that control NmCSS's ability to accommodate sterically demanding modifications at C‐9 position of Neu5Ac. Our studies revealed that the R173A mutant demonstrated substantially improved catalytic efficiency toward C‐9‐modified analogs, with an 18.4‐fold increase for ^FITC^Neu5Ac and a 46.3‐fold increase for ^BPA^Neu5Gc (mCD22 ligand). Strikingly, unlike wild‐type NmCSS, the R173A variant could process challenging dual‐modified substrates like the CD33 ligand **1d**, which carries modifications at both C‐5 and C‐9 positions. Structural analyses indicated these functional improvements originate from the significant expansion of the binding pocket, facilitating accommodation of bulky modifications while maintaining catalytic integrity. However, while our current data strongly support the mutant's enhanced processing of C‐9 modifications, we acknowledge that more comprehensive evaluation using diverse C‐5 modified analogs would be valuable to fully characterize the enzyme's substrate promiscuity.

Using the established OPTE synthesis strategy, we rapidly generated a diverse sialoside analog library and screened it on a customized glycan microarray. This approach uncovered not only novel high‐affinity ligands for hCD22 (e.g., **1m**) and also new scaffolds for mCD22 and mSig‐4, providing a foundation for rational ligand design. Some previously reported ligands, such as FTMC‐ and NSA‐modified sialosides, showed cross‐reactivity to other siglecs, highlighting their potential as universal probes ─ a feature that could simplify future Siglec‐targeting applications.

To translate these findings into a functional system, we successfully implemented one‐step glycoengineering of living cells, including CHO‐Lec2 and NK‐92MI cells, with high‐affinity Siglec ligands using a streamlined “one‐pot two‐enzyme” approach. The engineered NK‐92MI cells established tighter contacts with tumor cells via the Siglec‐ligand axis, resulting in enhanced cytotoxicity against hCD22^+^ B‐cell lymphoma and hCD33^+^ AML cells. Mechanistically, this effect was driven by increased granzyme release and cytokine secretion, all while preserving cell viability and minimizing off‐target activity. Together, these results establish a powerful and adaptable platform for advancing Siglec‐focused immunotherapies, opening new avenues for exploiting the Sia–Siglec axis in cancer treatment.

## Author Contributions

S.H., B.H., and L.W. contributed equally to this work. W.P. and L.D. conceived and designed the study. S.H. and B.H. carried out the synthesis and biological experiments. L.W. and R.Q. performed the computational analysis. Q.G., C.J., and S.W. assisted in recombinant Siglec protein expression. W.P. wrote the manuscript, and all authors reviewed and edited the manuscript.

## Conflicts of Interest

The authors declare no conflicts of interest.

## Supporting information




**Supporting File**: advs74645‐sup‐0001‐SuppMat.pdf.

## Data Availability

The data that support the findings of this study are available in the supplementary material of this article.
