# Supplementary material for: One‐Step Glycoengineering of NK Cells With High‐Affinity Siglec Ligands for Cancer Immunotherapy
Source: Adv Sci (Weinh). 2026 Mar 2;13(26):e22474. doi: 10.1002/advs.202522474 (PMC13159100; doi:10.1002/advs.202522474)
Supplement: Supplementary file 1 — Supporting File: advs74645‐sup‐0001‐SuppMat.pdf. [file ADVS-13-e22474-s001.pdf]

Supporting Information

One-step Glycoengineering of NK Cells with High-Affinity Siglec Ligands for Cancer Immunotherapy

Shuai Hu[#], Ben Huang[#], Lingyan Wang[#], Qiang Guo, Cuiping Jiang, Ruicheng Qi, Shuyao Wang, Lin-Tai Da, Wenjie Peng[*]

Key Laboratory of Systems Biomedicine (Ministry of Education), Shanghai Center for Systems Biomedicine, Shanghai Jiao Tong University, Shanghai 200240, China

Table of Contents

1. Supporting figures, schemes and tables S2
2. General materials S12
3. General methods S12
4. Synthesis section S18
5. NMR and MS spectra S32
6. References S84

1. Supporting Figures and Schemes

A

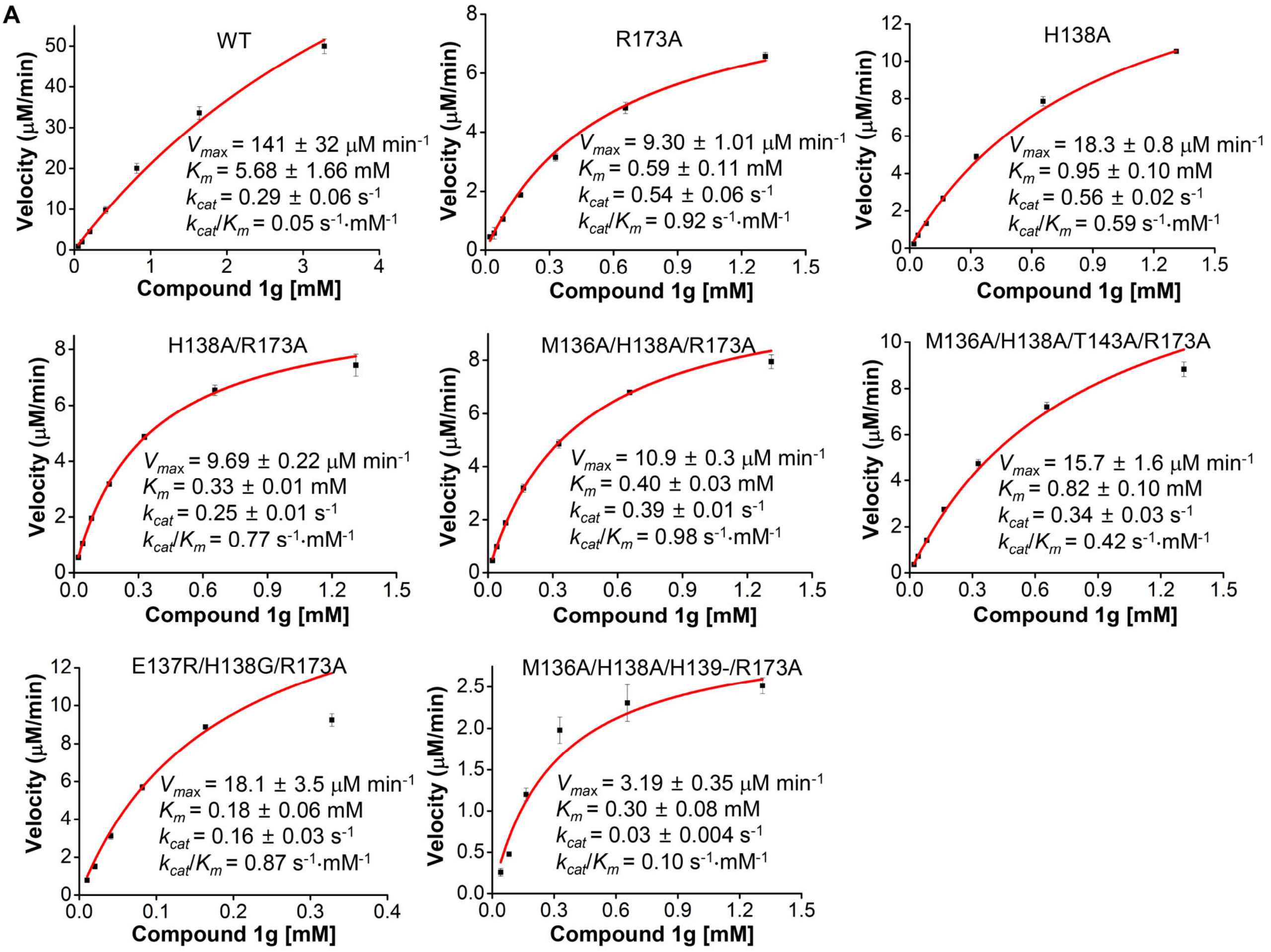

B

NmCSS variants	K_m (mM)	k_{cat} (s^{-1})	k_{cat}/K_m ($s^{-1}\cdot mM^{-1}$)	Improvement
WT	0.23 ± 0.02	11.7 ± 0.4	50.9	1
R173A	0.43 ± 0.06	16.3 ± 0.3	37.9	0.7
H138A	0.25 ± 0.03	12.2 ± 0.4	48.8	1
H138A/R173A	1.21 ± 0.15	5.0 ± 0.5	4.1	0.08
M136A	0.08 ± 0.04	9.3 ± 0.9	116	2.3
T143A	0.10 ± 0.01	24.7 ± 0.4	247	4.9
M136A/H138A/R173A	0.87 ± 0.05	19.2 ± 0.6	22.1	0.4
M136A/H138A/T143A/R173A	1.18 ± 0.11	22.9 ± 1.0	19.4	0.4

C

1c	K_m (mM)	k_{cat} (s^{-1})	k_{cat}/K_m ($s^{-1}\cdot mM^{-1}$)	Improvement
WT	2.76±0.35	0.22±0.02	0.08	1
R173A	0.21±0.04	0.77±0.05	3.7	46.3

D

1d	K_m (mM)	k_{cat} (s^{-1})	k_{cat}/K_m ($s^{-1}\cdot mM^{-1}$)	Improvement
WT	N.D.	N.D.	N.D.	1
R173A	0.97±0.14	0.20±0.02	0.21	N/A

Figure S1. Kinetic characterization of NmCSS variants using (A) ^{FITC}Neu5Ac (1g), (B) Neu5Ac, (C) ^{BPA}Neu5Gc (1c) and (D) CD33L (1d) as substrates. N.D., undetectable. N/A, not applicable.

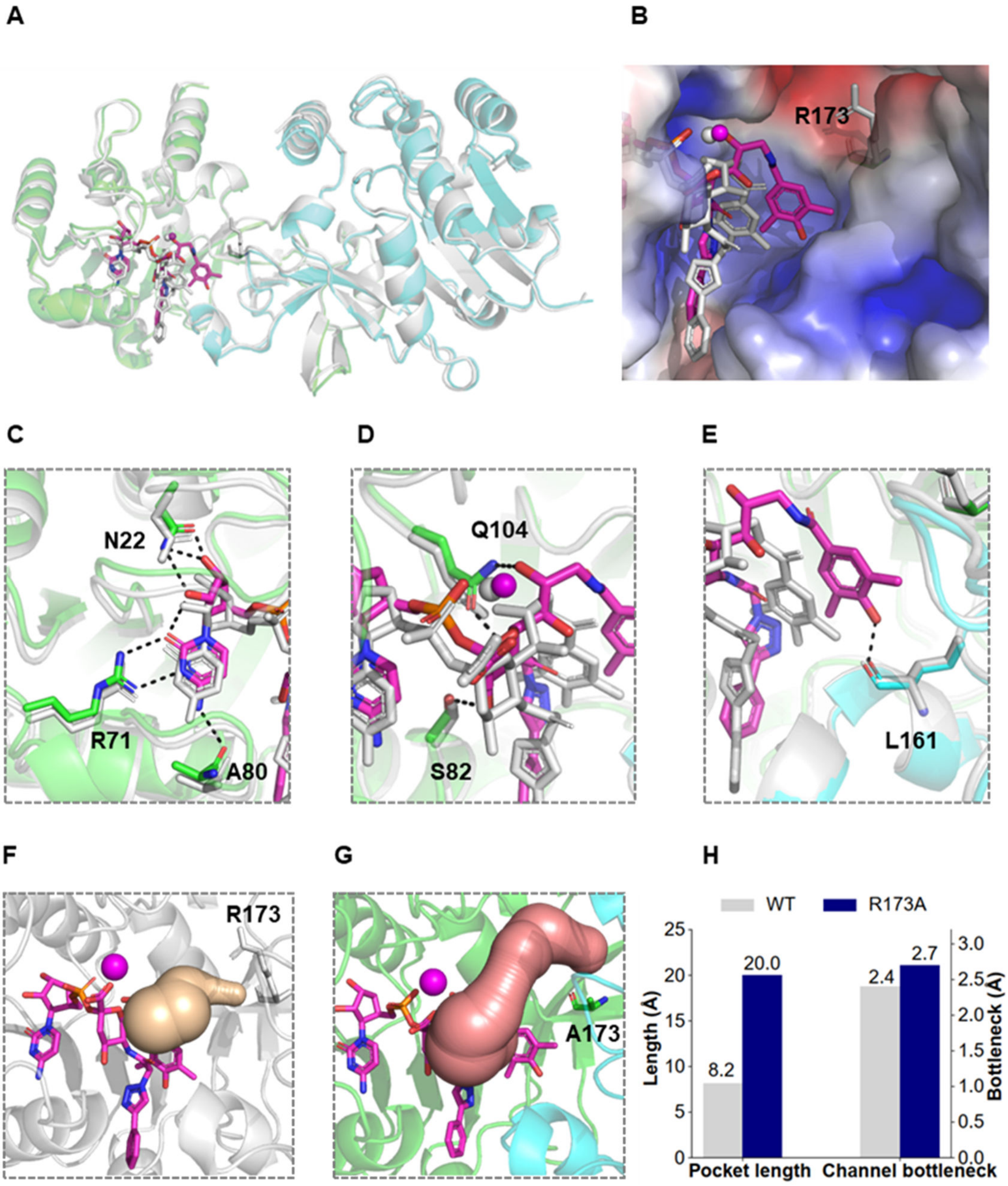

Figure S2. Structural insights into wild-type NmCSS and the R173A variant in complex with 1d-CMP. (A) Superposition of wild-type (gray) and R173A mutant (green for monomer A, light blue for monomer B) complexes. The Mg^{2+} ion is represented as a magenta sphere, while **1d**-CMP is isplayed as sticks (wild-type: gray; mutant: magenta). (B) Electrostatic potential analysis of the superimposed wild-type (gray) and R173A mutant NmCSS (red, positive; blue, negative). The mutation site (R173) is indicated. (C–E) Detailed interaction analysis of wild-type (gray) NmCSS and R173A mutant (green/light blue) complexed with distinct moieties of **1d**-CMP: (C) CMP, (D) CMP–Neu5Ac, and (E) the substituents at the C5 and C9 positions. Hydrogen bonds are shown as black dashed lines. (F–G) Characterization of binding-pocket cavity extending from R173/A173 to the **1d**-CMP in wild-type (gold, F) and mutant (pink, G) complexes using MOLE. (H) Quantitative measurement of the binding cavities in the wild-type (gray) and R173A mutant (dark blue) complexes.

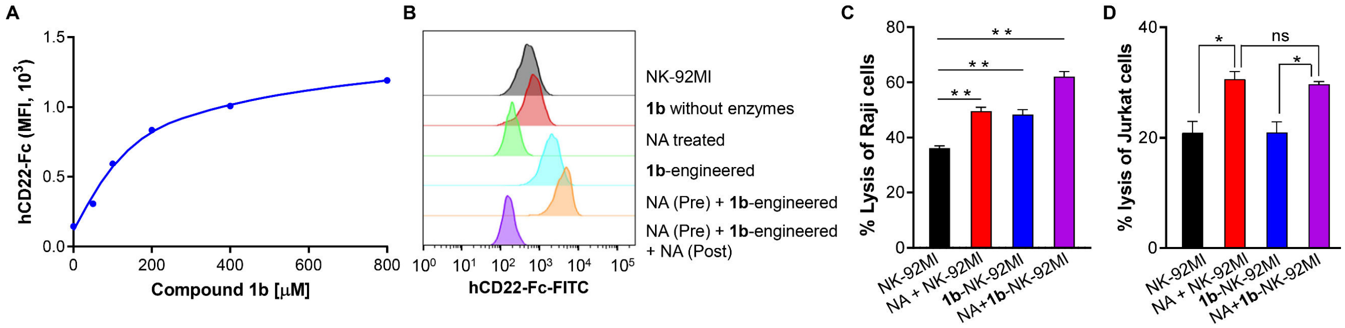

Figure S3. Glycoengineered NK-92MI cells displaying α2,6-linked ^{MPB}Neu5Ac (1b) exhibit enhanced hCD22-dependent cytotoxicity. (A) Concentration-dependent cell surface presentation of α2,6-linked **1b** on NK-92MI cells, quantified by flow cytometry using recombinant hCD22-Fc chimera (mean fluorescence intensity ± SEM, n=3). (B) Pre-desialylation with sialidase SpNanA (NA) enhanced hCD22-Fc binding, which completely abolished by following post-desialylation. (C) Pre-desialylated **1b**-NK-92MI cells showed enhanced cytotoxicity against hCD22-expresion Raji Cells (E/T = 5, 400 μM of **1b**). (D) Both native and **1b**-engineered NK-92MI cells w/o pre-desialylation exhibited comparable cytotoxicity against hCD22-negative Jurkat cells. The significance was analyzed with the two-sided t-test. (ns) not significant; (*) $p \leq 0.05$; (**) $p \leq 0.01$; (***) $p \leq 0.001$.

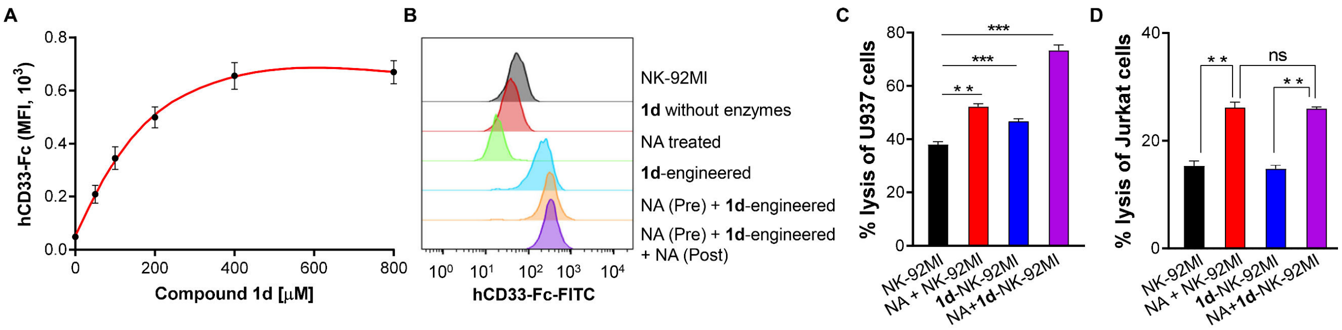

Figure S4. Glycoengineered NK-92MI cells displaying α2,6-linked CD33L (1d) exhibit enhanced hCD33-dependent cytotoxicity. (A) Concentration-dependent cell surface presentation of α2,6-linked **1d** on NK-92MI cells, quantified by flow cytometry using recombinant hCD33-Fc chimera (mean fluorescence intensity ± SEM, n=3). (B) Pre-desialylation with sialidase SpNanA (NA) enhanced hCD33-Fc binding, which completely abolished by following post-treatment with NA. (C) Sialidase pre-treated **1d**-NK-92MI cells showed enhanced cytotoxicity against hCD33-expression U937 Cells (E/T = 5, 400 μM of **1d**). (D) Both native and **1d**-engineered NK-92MI cells w/o pre-desialylation exhibited comparable cytotoxicity against CD33-negative Jurkat cells. The significance was analyzed with the two-sided t-test. (ns) not significant; (*) p ≤ 0.05; (**) p ≤ 0.01; (***) p ≤ 0.001.

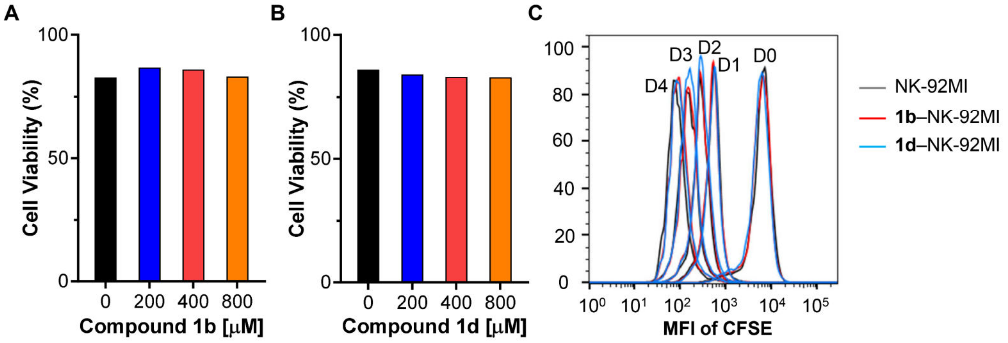

Figure S5. Viability and proliferation profiles of 1b- and 1d-engineered NK-92MI cells. (A-B) Cell viability analysis by flow cytometry using propidium iodide (PI) staining under various concentrations of **1b** and **1d** (mean ± SD, n = 3 independent experiments). (C) Proliferation kinetics of native versus glycoengineered (**1b** and **1d**) NK-92MI cells, assessed by CFSE assay over 96 hours.

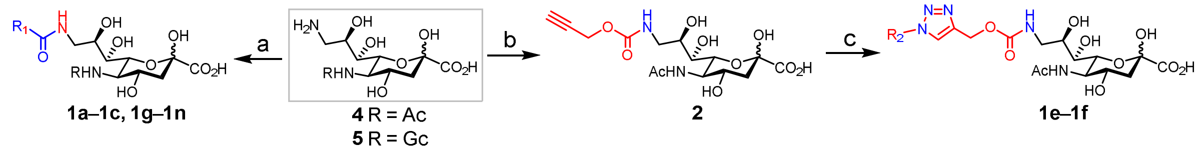

Scheme S1 Chemical synthesis of C9-amide-functionalized sialic acid derivatives (see Figure 4). Reaction conditions: (a) R_1CO-NHS, $NaHCO_3$, H_2O, 0°C–RT, 80–95%; (b) propargyl chloroformate, $NaHCO_3$, H_2O, 0°C–RT; (c) R_2N_3, $CuSO_4$, Na ascorbate, THPTA (cat.), DMF–H_2O, RT, 72% for **1e** and 85% for **1f**.

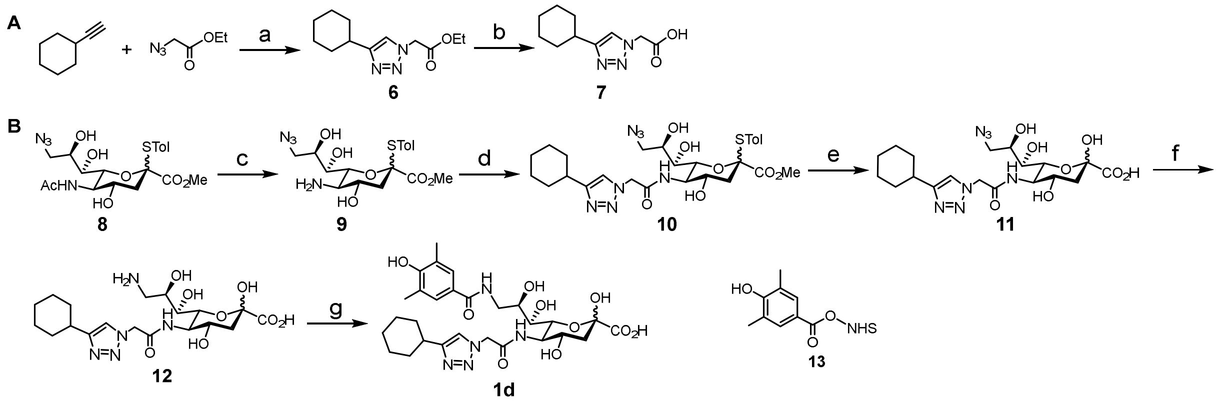

Scheme S2. Chemical synthesis of disubstituted sialic acid 1d. Reaction conditions: (a) $CuSO_4$, Na ascorbate, THPTA (cat.), t-BuOH/H_2O/THF = 1/2/6, RT, 95%; (b) NaOH, THF/H_2O = 5, 88%; (c) MsOH, MeOH, 60°C, 95%; d) **7** (1.2 eq.), HATU (1.5 eq.), DIEA, DMF, 77%; (e) i) LiOH, MeOH-H_2O (3:1); ii) NBS, acetone–H_2O (3:1), 83% for two steps; f) PMe_3, H_2O, 93%; g) **13** (3.0 eq.), $NaHCO_3$, H_2O, 88%.

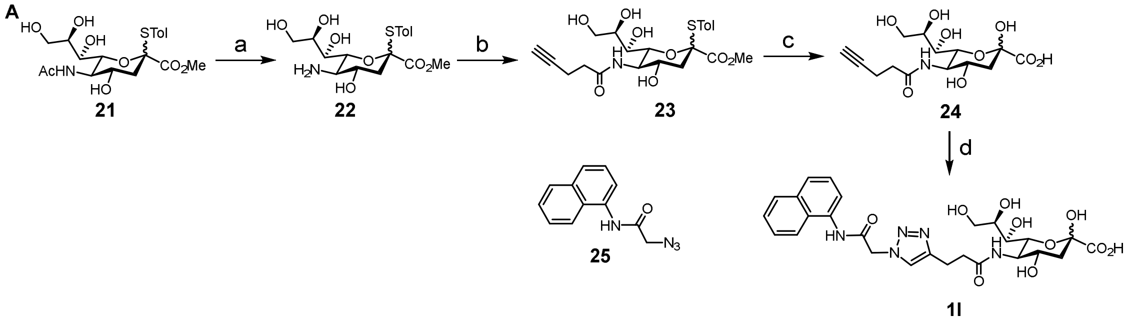

Scheme S3. Chemical synthesis of C-5-substituted sialic acid 1l. Reaction conditions: (a) MsOH, MeOH, 60°C; d) 4-pentynoic acid (1.2 eq.), HATU (1.2 eq.), DIEA, DMF, 87% for two steps; (c) i) LiOH, THF-H_2O (5:1); ii) NBS, acetone–H_2O (6:1), 81% for two steps; (d) **25** (1.3 eq.), Na ascorbate (1.3 eq.), $CuSO_4$ (cat.), THPTA (cat.), DMF–H_2O (1:1, v/v), RT, 88%.

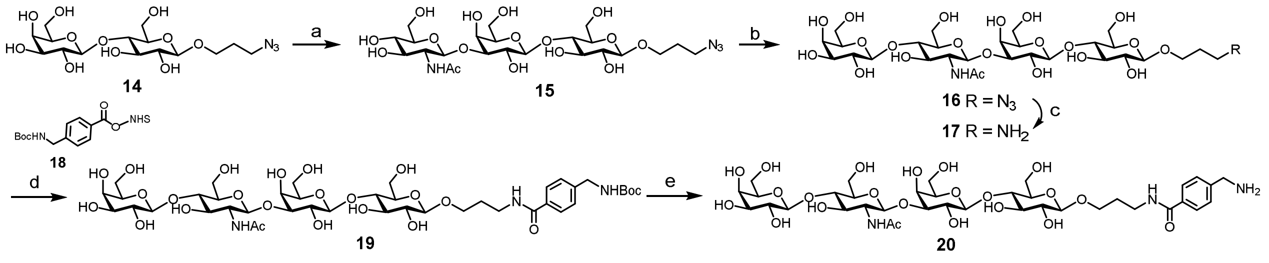

Scheme S4. Chemoenzymatic synthesis of linker-functionalized LNnT tetrasaccharide 20. Reaction conditions: (a) UDP-GlcNAc, HpLgtA, 37°C, 92%; (b) UDP-Glc, NmLgtB–GalE,

37°C, 90%; (c) PMe_3, H_2O, RT, 98%; (d) **18**, $NaHCO_3$, H_2O, 0°C–RT, 80%; (e) TFA, H_2O, RT, 92%.

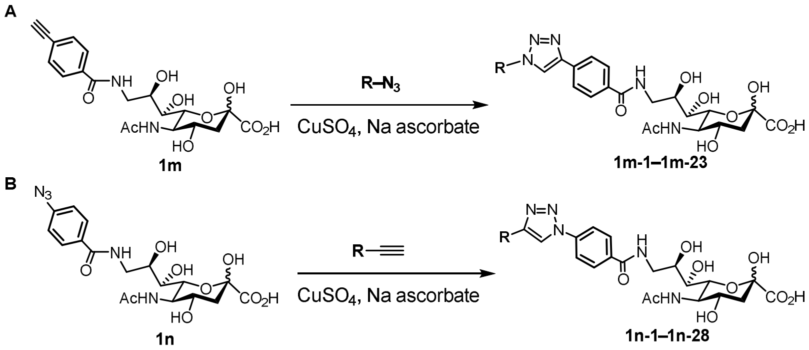

Scheme S5. Synthesis of C9-modified sialic acid derivatives via copper-catalyzed azide–alkyne cycloaddition (CuAAC). Azide and alkyne substrates used in this study are provided in Tables S2 and S3. Reaction conditions: azide or alkyne, $CuSO_4$, Na ascorbate, THPTA (cat.), DMF–H_2O, RT, 70–90%.

Table S1. Sequence of primers used for mutagenesis

NmCSS Variant	Primers	Sequences (5'-3')
WT	Forward	CTTAGGAGGTCATATGGAAAAACAAAATATTGCGGTTATAC
	Reverse	ACGCGTCGACTCATCAATGGTGATGATGGTGGTGGTTGCT TTCCTTGTGATTAAGAATGTTTTC
M136A	Forward	CAgcaGAGCATCATCCACTAAAAACCCTGCTT
	Reverse	TGGATGATGCTCtgcTGGGCATGCAGAGACAACG
H138A	Forward	CCCAATGGAGgctCATCCACTAAAAACCCTG
	Reverse	GTGGATGagcCTCCATTGGGCATGCAGAGAC
T143A	Forward	CCACTAAAAgcaCTGCTTCAAATCAATAATGGCG
	Reverse	AGCAGtgcTTTTAGTGGATGAGCCTCCATTGG
R173A	Forward	GCATTTgcgCCTAATGGTGCAATTTACATTAATG
	Reverse	GCACCATTAGGcgcAAATGCCTGAGGTAATTG
M136A/H138A/T143A	Forward	cagaggctcatccactaaaagcaCTGCTTCAAATCAATAATGGCG
	Reverse	ttagtggatgagcctctgcTGGGCATGCAGAGACAACG
M136G/E137G/H138G/H139G	Forward	AggtggtggtggtCCACTAAAAACCCTGCTTCAAATC
	Reverse	AGTGGaccaccaccaccTGGGCATGCAGAGACAACG
M136G/E137G/H138-/H139-	Forward	CTGCATGCCCAggtggTCCACTAAAAACCCTGCTTCA
	Reverse	TTTTTAGTGGAccaccTGGGCATGCAGAGACAACGG
E137R/H138G	Forward	AATGaggggtCATCCACTAAAAACCCTGCTTCA
	Reverse	AGTGGATGacccctCATTGGGCATGCAGAGACAA

Table S2 Chemical structures of sialosides B1-B23 and E1-E23.[a]

Substrate	R	Sialosides (Yield)		Substrate	R	Sialosides (Yield)	
		α2-3 linked	α2-6 linked			α2-3 linked	α2-6 linked
**1m-1**	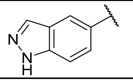	**B1** (84%)	**E1** (81%)	**1m-2**	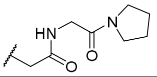	**B2** (94%)	**E2** (92%)
**1m-3**	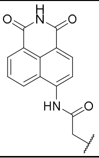	**B3** (40%)	**E3** (40%)	**1m-4**	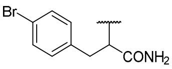	**B4** (90%)	**E4** (90%)
**1m-5**	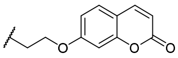	**B5** (71%)	**E5** (74%)	**1m-6**	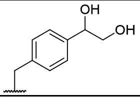	**B6** (83%)	**E6** (85%)
**1m-7**	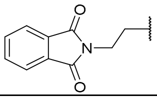	**B7** (97%)	**E7** (93%)	**1m-8**	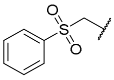	**B8** (99%)	**E8** (98%)
**1m-9**	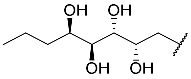	**B9** (95%)	**E9** (95%)	**1m-10**	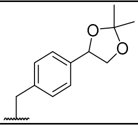	**B10** (79%)	**E10** (79%)
**1m-11**	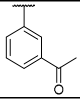	**B11** (90%)	**E11** (90%)	**1m-12**	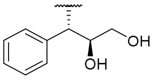	**B12** (90%)	**E12** (90%)
**1m-13**	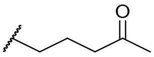	**B13** (90%)	**E13** (90%)	**1m-14**	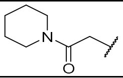	**B14** (80%)	**E14** (78%)
**1m-15**	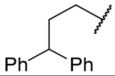	**B15** (84%)	**E15** (88%)	**1m-16**	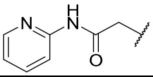	**B16** (79%)	**E16** (76%)
**1m-17**	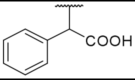	**B17** (87%)	**E17** (90%)	**1m-18**	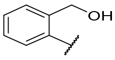	**B18** (96%)	**E18** (95%)
**1m-19**	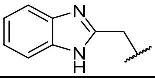	**B19** (93%)	**E19** (93%)	**1m-20**	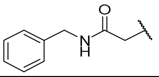	**B20** (93%)	**E20** (93%)
**1m-21**	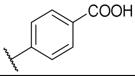	**B21** (90%)	**E21** (90%)	**1m-22**	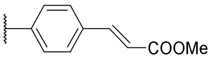	**B22** (95%)	**E22** (95%)
**1m-23**	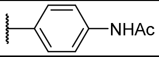	**B23** (90%)	**E23** (90%)				

[a] All sialosides were purified by HPLC.

Table S3 Chemical structures of sialosides C1-C28 and F1-F28.[a]

Substrate	R	**Sialosides** (Yield)		Substrate	R	**Sialosides** (Yield)	
		α2-3 linked	α2-6 linked			α2-3 linked	α2-6 linked
**1n-1**	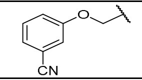	**C1** (90%)	**F1** (90%)	**1n-2**	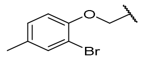	**C2** (86%)	**F2** (81%)
**1n-3**	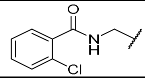	**C3** (90%)	**F3** (90%)	**1n-4**	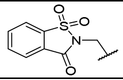	**C4** (90%)	**F4** (90%)
**1n-5**	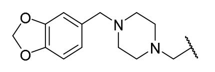	**C5** (90%)	**F5** (90%)	**1n-6**	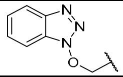	**C6** (90%)	**F6** (90%)
**1n-7**	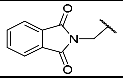	**C7** (83%)	**F7** (82%)	**1n-8**	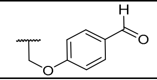	**C8** (90%)	**F8** (90%)
**1n-9**	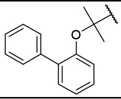	**C9** (90%)	**F9** (85%)	**1n-10**	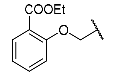	**C10** (90%)	**F10** (90%)
**1n-11**	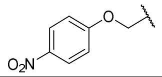	**C11** (90%)	**F11** (90%)	**1n-12**	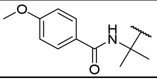	**C12** (90%)	**F12** (90%)
**1n-13**	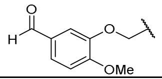	**C13** (90%)	**F13** (90%)	**1n-14**	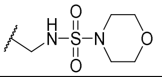	**C14** (90%)	**F14** (90%)
**1n-15**	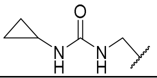	**C15** (90%)	**F15** (90%)	**1n-16**	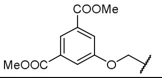	**C16** (90%)	**F16** (95%)
**1n-17**	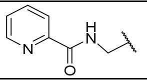	**C17** (88%)	**F17** (78%)	**1n-18**	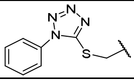	**C18** (90%)	**F18** (90%)
**1n-19**	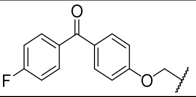	**C19** (95%)	**F19** (87%)	**1n-20**	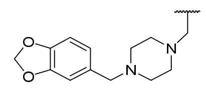	**C20** (90%)	**F20** (90%)
**1n-21**	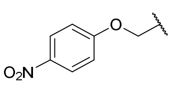	**C21** (90%)	**F21** (90%)	**1n-22**	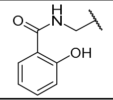	**C22** (90%)	**F22** (90%)
**1n-23**	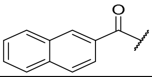	**C23** (95%)	**F23** (84%)	**1n-24**	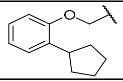	**C24** (90%)	**F24** (90%)
**1n-25**	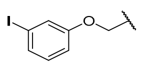	**C25** (90%)	**F25** (90%)	**1n-26**	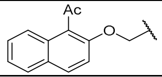	**C26** (90%)	**F26** (90%)
**1n-27**	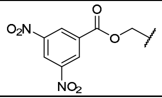	**C27** (95%)	**F27** (95%)	**1n-28**	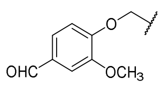	**C28** (90%)	**F28** (90%)

[a] All sialosides were purified by HPLC.

Addtional sialoside analogs on the array:

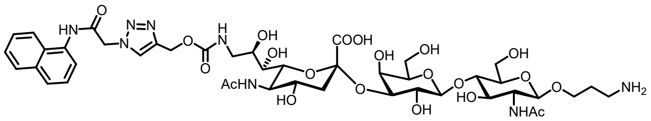

B24

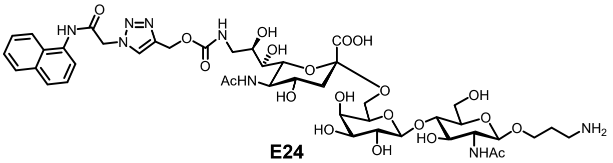

E24

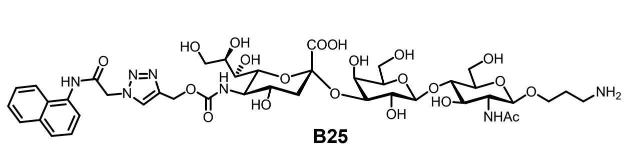

B25

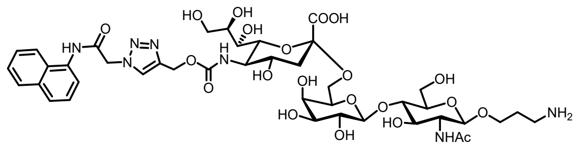

E25

2. Materials

All chemical compounds were purchased from commercialized suppliers and used without further purification unless otherwise specified. Thin layer chromatography (TLC) was performed on silica gel plates 60 F_{254} (Jiangyou, Yantai, China) and visualized under UV light and/or by treatment with 10% sulfuric acid in ethanol after heating. Silica gel 60 (300-400 mesh, Titansci, shanghai, China) was used for flash silica gel column chromatography. Gel filtration chromatography was performed using a column (100 cm × 1.0 cm) packed with BioGel P-2 Fine resins (Bio-Rad, Hercules, CA) or Sephadex G-25 super fine resins (Cytiva, MA). Goat anti-Human IgG Fc (DyLight® 650) was obtained from Abcam. Alexa Fluor 647-conjugated AffiniPure goat anti-rabbit IgG (H+L) was purchased from Jackson ImmunoResearch. Rabbit anti-human IgG Fc was purchased from Sigma-Aldrich. Mouse anti-Strep IgG was purchased from Solarbio Science & Technology (Beijing, China). CoraLite488-conjugated goat anti-mouse IgG (H+L) and Anti-Human CD107a / LAMP1 were purchased from Proteintech (Wuhan, China). Cell culture media and fetal bovine serum (FBS) were purchased from Gibco (ThermoFisher, USA).

Human and murine Siglec-Fc chimeras were expressed from Chinese hamster ovary (CHO) cell lines as previously described.[1] *Helicobacter pylori* β1,3-N-acetylglucosaminyltransferase (HpLgtA)[2], *Neisseria meningitides* β1,4-galactosyltransferase/GalE fusion enzyme (NmLgtB-GalE)[3], *Photobacterium damselae* α2,6-sialyltransferase (Pd2,6ST)[4], *Neisseria meningitides* α2,3-sialyltransferase 3 (NmST3)[5], *Streptococcus pneumoniae* R6 neuraminidase A (SpNanA, NA)[6], and *Escherichia Coli* pyrophosphatase (PPA)[7] were expressed and purified as reported in the literature.

Tris(3-hydroxypropyltriazolylmethyl)amine (THPTA) was synthesized according to literature.[8]

3. General Methods

NMR spectra were recorded on Bruker Avance III (400M Hz) or Bruker AVANCE NEO (700 MHz) instrument at 25ºC. NMR data were processed with Mnova software. MALDI-TOF data were recorded on an Applied Biosystems DE MALDI-TOF (ThermoFisher) using dihydroxybenzoic acid as the matrix. ESI-MS data were recorded on a Waters Acquity UPLC with SQD2 mass spectrometer. Flow cytometry data were acquired on a LSR Fortessa flow cytometer (BD Biosciences) and analyzed using the Flowjo software.

3.1 Computation modeling:

The CMP-^{FITC}Neu5Ac and Mg^{2+} bound NmCSS complex (termed as CMP-^{FITC}Neu5Ac–NmCSS afterwards) was modeled based on a crystal structure of NmCSS (PDB ID: 6CKK)[9] where NmCSS exists as a homodimer and binds with one CTP molecule (Figure 2B). The CTP was then replaced with the CMP-Neu5Ac structure derived from another crystal structure of CSS (PDB ID: 1QWJ).[10] Finally, the CMP-^{FITC}Neu5Ac–NmCSS model was obtained by manually attaching one FITC group at the C9 position of CMP-Neu5Ac.

To analyze the molecular mechanism of CMP-^{FITC}Neu5Ac recognition by the R173A mutant of NmCSS, we substituted R173 with Alanine based on the above wild type structure. The final model was then subject to energy minimization using steepest decent and conjugate gradient methods. In specific, NmCSS was described by ff14SB.[11] To derive the AMBER parameters of

CMP-^{FITC}Neu5Ac, the electrostatic potential energy was calculated with HF/6-31G* basis set using Gaussian 09 package,[12] then the restrained electrostatic potential (RESP) charges were determined using the antechamber program.[13] Other parameters of CMP-^{FITC}Neu5Ac were adopted from the GAFF force field.[14] The CMP-^{FITC}Neu5Ac-NmCSS^{R173A} complex was immersed into a cubic box filled with TIP3P water, and 11 Na^+ ions were added to neutralize the whole system. Finally, energy minimization was performed using the Amber-18 package.[15]

The **1d**-CMP–NmCSS complex was modeled using the AlphaFold3-based Seedfold platform with default parameters.[16] In brief, protein sequences were provided in FASTA format, **1d**-CMP was encoded as a SMILES string, and Mg^{2+} coordination was specified using the appropriate Chemical Components Dictionary (CCD) parameters. The top-ranked model was selected based on the interface predicted Template Modeling (ipTM) scores. The R173A variant was generated by in silico mutagenesis of the wild-type structure. The **1d**-CMP–NmCSS^{R173A} complex was refined using the same protocol.

The electrostatic potential calculations (Figure S2B) were performed using the APBS Electrostatics plugin[17] implemented in PyMOL (https://pymol.org/). Quantitative geometric analyses of the binding pocket (length and channel bottleneck) were conducted using MOLEonline[18]. All molecular graphics were rendered using PyMOL.

3.2 Construction of NmCSS Mutants:

The mutagenic PCR was performed using the native NmCSS gene in the pCWori vector as the template, with primers listed in Table S1. Following digestion, the PCR product was transformed into *E. coli* DH5α competent cells. Selected clones were subjected to minipreps and DNA sequencing to confirm the mutation. Plasmids harboring the target genes were subsequently transformed into *E. coli* BL21(DE3) competent cells for protein expression. The transformed bacteria were cultured in LB rich medium supplemented with ampicillin (100 μg/mL). The target protein was then overexpressed and purified according to the previously described procedure.[19]

3.3 Thin-layer chromatography analysis of NmCSS variant activity toward ^{FITC}Neu5Ac (1g).

Reaction mixtures containing 2 mM **1g**, 3 mM CTP, and an appropriate amount of each NmCSS mutant were incubated in 100 mM Tris·HCl buffer (20 μL, containing 20 mM $MgCl_2$, 0.2 mg/mL BSA, pH 8.5) at 37°C for 10 min. Product (**1g-CMP**) formation was analyzed by TLC (iPrOH/NH_4OH/H_2O, 5/2/1, v/v/v) and visualized under UV light at 365 nm.

3.4 Measurement kinetic parameters for NmCSS variants via malachite green assay[20]:

All Sia derivatives were quantified using the Periodate-Resorcinol Assay.[21-22]

Preparation of Malachite Green Reagents:

- Reagent A: 1 mL H_2SO_4 (conc.), 5 mL H_2O, and 7.3 mg malachite green powder;
- Reagent B: 2 mL Reagent A, 0.5 mL ammonium molybdate (7.5% in H_2O), 40 uL Tween-20 (11% v/v).

Pi standard Curves:

To generate a phosphate standard curve, 20 μL of reaction buffer containing diluted K_2HPO_4 (1.25–40 μM) was added to 180 μL cold H_2O, followed by 50 μL Reagent B. The mixture was gently mixed and incubated at RT for 15 min. Absorbance was measured at 620 nm using a multiwell plate reader (Biotek Cytation 5).

Enzymatic reactions:

Reactions were carried out in 30 μL of Tris·HCl buffer (100 mM, pH 8.5) containing 20 mM $MgCl_2$, 2.5% DMSO, 5 mM CTP, 0.2 mg/mL BSA, appropriate amount of each NmCSS variant (typically 0.005–0.05 mg/mL), 0.1 mg/mL PPA and serial dilutions of Sia derivatives, in a 96-well plate. A blank control lacking the Sia derivative was included.

Reactions were initiated by addition of NmCSS, followed by shaking at 37°C for 10 min. The reaction was stoppeded by placing the plate on ice. Subsequently, 20 μL of the reaction mixture was transferred to 180 μL cold H_2O, followed by addition of 50 μL Reagent B. The mixture was gently mixed, incubated at RT for 15 min, and absorbance was recorded at 620 nm (Biotek Cytation 5).

All assays were performed in triplicate. Kinetic parameters were determined by curve fitting of Michaelis–Menten plots using Origin 8 software.

3.5 General Procedure for copper-catalyzed azide-alkyne cycloaddition (CuAAC):

The corresponding sialoside, sodium ascorbate (1.0 equiv.), and Tris(3-hydroxypropyltriazolylmethyl) amine (THPTA, 0.4 equiv.) were dissolved in H_2O. To this solution, the appropriate alkyne or azide (1.5 equiv.) in DMF was added, followed by $CuSO_4$ (0.2 equiv.) to initiate the reaction. The mixture was shaken at room temperature. Upon completion, the reaction was diluted with water and lyophilized to remove organic solvent. The resulting residue was purified by RP-HPLC.

3.6 General Procedure for "one-pot two-enzyme" approach to synthesize sialoside analogs:

A mixture of LNnT (**20**, 10 mM), sialic acid analog (1.5 equiv.), CTP (2.0 equiv.), and NmCSS variant in Tris·HCl (100 mM, pH 8.5) containing 20 mM $MgCl_2$ was incubated at 37°C. The reaction was monitored by TLC until completion, after which either Pd2,6ST or ST3 was added, and the reaction was further incubated at 37°C for 8 h. The reaction mixture was then centrifuged, the supernatant was filtered through a 0.22 μm filter before purification by RP-HPLC.

3.7 RP-HPLC analysis:

Reaction yields were determined by HPLC on a Shimadzu LC-20AD system equipped with a GL Sciences reverse-phase (RP) C-18 column (250 × 2.1 mm) and a detector monitoring absorbance at 220 and 254 nm. The flow rate was maintained at 1.0 mL/min for all analyses, with the following specific gradient conditions:

1) *For sialosides:* Solvent A = 95% H_2O with 0.1% TFA; Solvent B = 95% CH_3CN with 0.1% TFA.

   Gradient: 0-2 min, 0% B; 2-20 min, 0-100% B; 20-25 min, 100% B.

2) *For CMP-Sia:* Solvent A = 95% H_2O with 10 mM KH_2PO_4, pH 8.0; Solvent B = 90% CH_3CN with 10% solvent A.

Gradient: 0-2 min, 0% B; 2-22 min, 0-15% B; 22-24 min, 15-100% B; 24-30 min, 100% B.

3) *For CMP-^{FITC}Neu5Ac:* Solvent A and B as above for CMP-sialic acids.

   Gradient: 0-2 min, 0% B; 2-20 min, 0-25% B; 20-25 min, 25-100% B; 25-30 min, 100% B.

4) *For CMP-^{BPA}Neu5Gc*: Solvent A and B as above for CMP-sialic acids.

   Gradient: 0-2 min, 0% B; 2-5 min, 0-40% B; 5-15 min, 40-55% B; 15-18 min, 55-100% B; 18-23 min, 100% B.

5) *For CMP-CD33L:* Solvent A and B as above for CMP-sialic acids.

   Gradient: 0-2 min, 0% B; 2-5 min, 0-40% B; 5-15 min, 40-65% B; 15-18 min, 65-100% B; 18-23 min, 100% B.

3.8 Glycan microarray study:

This protocol complies with MIRAGE Glycan Array Guideline v 1.0.[23] Glycans were printed onto *N*-hydroxy succinimide activated glass slides (Slide H, Schott/Nexterion) at 100 μM in printing buffer (150 mM phosphate, pH 8.5 containing 0.005% Tween-20) in six replicates as previously described[24] using a Smart Arrayer48 microarray printing system (CaptitalBio™). Each Siglec (10 μg/mL) was pre-complexed with Dylight 650-labeled anti-human IgG-Fc (5 μg/mL) for 30 min and then applied to the array surface for 1 hour incubation. Slides were subsequently washed by successive rinses with PBS-T (300 mM phosphate, 0.005% Tween-20), PBS and deionized H_2O. Fluorescence signals (Alexa Fluor 647 channel) were acquired using a Genepix 4000A microarray scanner (Molecular Devices). Data processing was performed using a custom Microsoft Excel Macro developed by Dr. Lin Liu (available at http://zenodo.org/record/5146251). For each glycan, the highest and the lowest signal were removed and the remaining four replicates were used to calculate the average intensity along with the standard deviation (SD).

3.9 Cell culture:

CHO-Lec2 cells were maintained in αMEM medium supplemented with 10% (vol/vol) heat-inactivated FBS, 100 U/mL penicillin G and 100 mg/mL streptomycin. NK-92MI cells were cultured in αMEM containing 12.5% FBS, 12.5% horse serum, 200 μM inositol, 20 μM folic acid, 50 μM 2-mercaptoethanol, 100 U/mL penicillin G, and 100 mg/mL streptomycin. U937, Raji and Jurkat cells were cultured in RPMI 1640 medium supplemented with 10% heat-inactivated FBS, 100 U/mL penicillin G, and 100 mg/mL streptomycin.

3.10 General Procedure for one single step incorporation of Siglec ligand onto live cells via "One-pot two-enzyme" approach:

CMP-Sia derivatives were prepared *in situ* by incubating sialic acid analogs (10 mM) with CTP (20 mM) and NmCSS R173A (0.5 mg/mL) in Tris·HCl (100 mM, pH 8.5) containing 20 mM $MgCl_2$ was incubated at 37°C. The reaction was monitored by TLC until completion.

For glycoengineering of live cells: cells (0.5 x 10^6) were resuspended in 100 μL reaction buffer (HBSS supplemented with 3 mM HEPES and 20 mM $MgCl_2$, pH 8.0), containing CMP-Sia derivative (25-800 μM) and Pd2,6ST (20 μg/mL) at 25°C for 30 min. Engineered cells were then washed three times with PBS, resuspended in PBS and stained with Siglec-Fc chimeras

(10 μg/mL) precomplexed with APC-conjugated anti-human Fc antibody (5 μg/mL) on ice for 30 min. After washing, resuspended in FACS buffer, and analyzed by flow cytometry.

Sialidase treatment was conducted in 100 μL HBSS buffer (3 mM, pH 6.5) containing 0.1 mg/mL SpNanA at 37°C for 20 min, either before or after the incorporation of the Sia analogs.

3.11 Confocal imaging:

Cells were cultured on 13 mm round coverslips in a 24 well plate. After glycoengineered with 300 μL of reaction buffer containing CMP-Sia derivative (100 μM), the cells were washed three times with pre-warmed PBS and fixed at RT with 4% paraformaldehyde (PFA) for 15 min. Fixed cells were blocked with 3% BSA in PBS for 1 h at RT, followed by incubation with pre-complexed Siglec-Fc (10 μg/mL) and rabbit anti-human IgG Fc (5 μg/mL) at 4 °C overnight.

After three washes with PBS, cells were incubated with AF647-conjugated goat anti-rabbit IgG antibody at 4 °C for 1 h. Subsequently, cells were washed with PBS, permeabilized with 0.25% Triton X-100 in PBS for 15 min at RT, and counterstained with DAPI. Confocal images were acquired using a Nikon A1Si confocal laser scanning microscope.

3.12 Viability and proliferation of glycoengineered NK-92MI cells:

Cell viability assay: native or engineered cells (0.5 x 10^6 cells) were resuspended in 1 mL of PBS containing 10 μg/mL propidium iodide (PI) and incubated on ice for 5 min. Samples were analyzed by FACS, and cell viability was determined by the percentage of PI-negative cells.

Cell proliferation assay: native or engineered cells (0.5 x 10^6 cells per sample) were labeled with 1 μM carboxyfluorescein succinimidyl ester (CFSE) at 37°C for 15 min. The cells were washed three times with PBS, and resuspended in complete culture medium supplemented with FBS. CFSE fluorescence was analyzed by flow cytometry on days 0, 1, 2, 3, and 4 to monitor cell proliferation.

3.13 Assessment of the NK-92MI cytotoxicity against cancer cells:

CFSE-labeled tumor cells (1 x 10^4 cells per well) were co-cultured with native or glycoengineered NK-92MI cells at different effector-to-target (E/T) ratios (1:1, 2:1, and 5:1) in 200 μL RPMI-1640 medium at 37°C for 3 h. Then, cells were stained with 10 μg/mL PI on ice for 5 min. Flow cytometry was used to quantify cytotoxicity, defined as the percentage of CFSE and PI double positive cells. The cytotoxicity rate was calculated using the following formula:

Cytotoxicity (%) = (Target cell death% - Spontaneous death%)/(1 - spontaneous death%) x 100.

3.14 IFN-γ and Granzyme B release assay:

Cancer cells (1 x 10^4 cells per well) were co-cultured with NK-92MI cells at an effector-to-target (E/T) ratio of 5:1 in 200 μL RPMI-1640 medium at 37°C for 3 h. After centrifugation, the supernatants were collected and analyzed using Human IFN-gamma ELISA Kit (Multi Sciences Biotech, Hangzhou, China) and Human Granzyme B ELISA Kit (Multi Sciences Biotech, Hangzhou, China), according to the manufacturer's instructions.

3.15 CD107a expression of NK-92 MI cells:

Glyco-engineered NK-92 MI cells were labeled with 1 μM CFSE and co- cultured with cancer cells at E/T = 5 at 37°C for 3 h. The cells were centrifuged and washed by PBS, followed with

staining of Anti-CD107a antibody (10 μg/mL) and Alexa Fluor 647 AffiniPure Goat Anti-Mouse IgG (H+L) (5 μg/mL) on ice for 30 min in the dark. Then, the cells were washed and resuspended in FACS buffer for flow cytometry analysis.

4. Synthesis section

Compound **1a**[25], **1b**[26], **2**[27], $^{9\text{-}NH2}$Neu5Ac (**4**)[28], $^{9\text{-}NH2}$Neu5Gc (**5**)[29], and LNnT (**16**)[30] were synthesized as previously reported.

Compound 1c:

BPA-NHS (50 mg, 0.16 mmol) in 1 mL THF was added to the mixture of compound **5** (35.5 mg, 0.11 mmol) and $NaHCO_3$ (92 mg, 1.09 mmol) in 1 mL water. The reaction was stirred at room temperature until the starting material was consumed. THF was evaporated and the resulting residue was purified by RP-HPLC to afford **1c** (48 mg, 85%) as a syrup.

^{1}H NMR (400 MHz, D_2O): δ 7.73 – 7.63 (m, 5H), 7.51 (t, *J* = 8.6 Hz, 2H), 7.46 -7.35 (m, 2H), 4.17 – 4.05 (m, 4H), 3.99 (t, *J* = 10.2 Hz, 1H), 3.84 – 3.74 (m, 1H), 3.66 (s, 2H), 3.61 (dd, *J* = 14.1, 3.1 Hz, 1H), 3.47 – 3.41 (m, 1H), 3.28 (dd, *J* = 14.2, 7.6 Hz, 1H), 2.30 (dd, *J* = 13.0, 4.9 Hz, 1H), 1.87 (t, *J* = 12.3 Hz, 1H); ^{13}C NMR (151 MHz, D_2O) : δ 175.5, 175.1, 175.04, 140.1, 139.4, 134.4, 129.73, 129.69, 129.1, 127.7, 127.3, 126.9, 126.8, 110.4, 95.9, 75.4, 70.0, 68.8, 66.7, 61.0, 51.9, 49.5, 42.9, 42.0, 39.2; ESI-MS: m/z Calc'd for $C_{25}H_{30}N_2O_{10}$: 518.2; found:519.3 $[M + H]^+$.

Compound 1c-CMP:

Compound **1c** (3.0 mg, 5.79 μM) and CTP (4.5 mg, 1.5 equiv.) were dissolved in 579 μL Tris·HCl (100 mM, pH 8.5) containing $MgCl_2$ (20 mM) and NmCSS R173A (0.1 mg/mL). The mixture was incubated at 37°C, which progress was monitored by TLC analysis. After completion, the reaction was centrifuged, and the supernatant was filtered through a 0.22 μm filter and purified by RP-HPLC, affording the product **1c-CMP** (4.1 mg, 86%).

^{1}H NMR (700 MHz, CD_3OD): δ 7.89 (d, *J* = 7.5 Hz, 1H), 7.75 – 7.71 (m, 4H), 7.52 (t, *J* = 7.7 Hz, 2H), 7.47 (d, *J* = 8.0 Hz, 2H), 7.42 (t, *J* = 7.5 Hz, 1H), 5.99 (d, *J* = 7.6 Hz, 1H), 5.83 (d, *J* = 4.3 Hz, 1H), 4.32 (d, *J* = 10.7 Hz, 1H), 4.26 (t, *J* = 5.2 Hz, 1H), 4.25 – 4.19 (m, 1H), 4.17 (s, 2H), 4.13 – 4.09 (m, 2H), 4.07 (t, *J* = 10.4 Hz, 1H), 4.05 – 3.97 (m, 2H), 3.84 – 3.79 (m, 1H), 3.75 – 3.66 (m, 3H), 3.37 (d, *J* = 9.6 Hz, 1H), 3.17 (dd, *J* = 13.9, 9.5 Hz, 1H), 2.54 (dd, *J* = 13.3, 4.8 Hz, 1H), 1.72 – 1.65 (m, 1H); ESI-MS: m/z Calc'd for $C_{34}H_{42}N_5O_{17}P$: 823.2; found:822.6 $[M - H]^-$.

Compound 1d:

To a solution of compound **12** (60 mg, 0.13 mmol) in 1 mL water, $NaHCO_3$ (5 equiv.) was added, followed by addition of compound **13** (3 equiv.). The reaction was stirred at room temperature until the reaction was complete. The reaction was loaded to C18 column and eluted with H_2O-ACN. After concentration and lyophilization, the product **1d** was obtained as a solid (53 mg, 88%).

^{1}H NMR (700 MHz, D_2O): δ 7.49 (s, 1H), 7.41 (s, 2H), 5.21 (d, *J* = 16.5 Hz, 1H), 5.11 (d, *J* = 16.5 Hz, 1H), 4.17 – 4.06 (m, 2H), 3.98 (t, *J* = 10.3 Hz, 1H), 3.92 – 3.84 (m, 1H), 3.63 (dd, *J* = 14.4, 3.4 Hz, 1H), 3.58 (dd, *J* = 14.4, 5.7 Hz, 1H), 3.47 (dd, *J* = 9.1, 1.1 Hz, 1H), 2.61 – 2.51 (m, 1H), 2.30 (dd, *J* = 13.0, 5.0 Hz, 1H), 2.20 (s, 6H), 1.91 – 1.79 (m, 3H), 1.75 – 1.68 (m, 2H), 1.67 – 1.61 (m, 1H), 1.38 – 1.07 (m, 5H); ^{13}C NMR (176 MHz, D_2O): δ 173.6, 171.1, 168.0, 155.6, 153.5, 127.7, 125.0, 123.2, 95.4, 70.0, 68.9, 68.8, 66.4, 52.5, 52.2, 42.9, 38.8, 34.1, 32.1, 32.0, 25.4, 25.3, 15.7; ESI-MS: m/z Calc'd $C_{28}H_{39}N_5O_{10}$ for 605.3, found: 606.4 $[M + H]^+$.

Compound 1d-CMP:

Compound **1d** (3.0 mg, 4.95 μM) and CTP (3.9 mg, 1.5 equiv.) were dissolved in 495 μL Tris·HCl (100 mM, pH 8.5) containing $MgCl_2$ (20 mM) and NmCSS R173A (0.5 mg/mL). The mixture was incubated at 37°C, which progress was monitored by TLC analysis. After completion, the reaction was centrifuged, and the supernatant was filtered through a 0.22 μm filter and purified by RP-HPLC, affording the product **1d-CMP** (4.2 mg, 93%).

^{1}H NMR (700 MHz, D_2O): δ 7.81 (d, J = 7.6 Hz, 1H), 7.53 (s, 1H), 7.36 (s, 2H), 6.02 – 5.96 (brs, 1H), 5.87 (d, J = 4.4 Hz, 1H), 5.22 (d, J = 16.6 Hz, 1H), 5.15 (d, J = 16.6 Hz, 1H), 4.29 – 3.98 (m, 7H), 3.83 – 3.73 (m, 1H), 3.67 – 3.50 (m, 2H), 3.44 (dd, J = 14.0, 7.6 Hz, 1H), 3.40 (d, J = 9.3 Hz, 1H), 2.65 – 2.56 (m, 1H), 2.48 (dd, J = 13.3, 4.8 Hz, 1H), 2.17 (s, 6H), 1.91 – 1.84 (m, 2H), 1.76 – 1.69 (m, 2H), 1.67 – 1.61 (m, 2H), 1.37 – 1.15 (m, 5H); ^{13}C NMR (176 MHz, D_2O): δ 165.8, 141.1, 127.6, 125.0, 122.8, 89.0, 82.8, 74.4, 71.5, 70.0, 69.4, 68.5, 66.5, 65.0, 62.4, 52.1, 51.9, 42.3, 41.0, 34.3, 32.3, 32.2, 25.5, 25.4, 15.8; ESI-MS: m/z Calc'd for $C_{37}H_{51}N_8O_{17}P$: 910.3; found: 911.4 $[M + H]^+$.

Compound 1e:

A solution of K_2CO_3 (64 mg, 0.46 mmol) in 800 μL water was added to a mixture of 5-aminofluorescein (64 mg, 0.18 mmol), imidazole-1-sulfonyl azide hydrochloride (46 mg, 0.22 mmol) and $CuSO_4{\cdot}5H_2O$ (0.5 mg, 0.002 mmol) in DMF (200 μL), stirred at room temperature overnight. When the starting material was consumed, the mixture was lyophilized to affored the 5-azidofluorescein, which was directly subjected to the CuAAC reaction with compound **2** (23.3 mg, 0.060 mmol) without further purification. After completion, compound **1e** (72%) was yielded by purification with RP-HPLC.

^{1}H NMR (700 MHz, CD_3OD): δ 8.75 (s, 1H), 8.52 (s, 1H), 8.31 (dd, J = 8.2, 2.0 Hz, 1H), 7.47 (d, J = 8.3 Hz, 1H), 6.98 – 6.75 (m, 5H), 6.66 (d, J = 9.0 Hz, 2H), 5.28 (s, 2H), 4.07 – 3.97 (m, 2H), 3.83 (t, J = 10.3 Hz, 1H), 3.77 – 3.71 (m, 1H), 3.60 (dd, J = 14.0, 3.4 Hz, 1H), 3.40 (d, J = 9.2 Hz, 1H), 3.20 (dd, J = 14.2, 7.3 Hz, 1H), 2.21 (dd, J = 12.9, 5.0 Hz, 1H), 2.00 (s, 3H), 1.82 (t, J = 12.1 Hz, 1H); ^{13}C NMR (176 MHz, CD_3OD): δ 170.0, 159.0, 159.0, 154.2, 131.0, 130.6, 130.3, 130.1, 128.6, 127.5, 127.3, 127.2, 124.3, 124.3, 117.5, 113.8, 110.8, 103.6, 96.6, 82.1, 72.1, 71.7, 70.6, 67.8, 54.2, 49.5, 47.94, 45.68, 41.00, 37.43, 22.68; ESI-MS: m/z Calc'd for $C_{35}H_{33}N_5O_{15}$: 763.2; found: 764.3 $[M + H]^+$.

Compound 1f:

Adamentyl azide, prepared as described previously[31] was mixed with compound **2** (16.7 mg, 0.043 mmol), followed by the general procedure of CuAAC to afford compound **1f** (85%) by RP-HPLC separation.

^{1}H NMR (400 MHz, D_2O): δ 8.12 (s, 1H), 5.16 (s, 2H), 4.05 (dd, J = 10.6, 4.7 Hz, 2H), 3.91 (t, J = 10.2 Hz, 1H), 3.80 – 3.68 (m, 1H), 3.59 – 3.44 (m, 2H), 3.15 (dd, J = 14.3, 7.5 Hz, 1H), 2.30 (dd, J = 13.1, 4.9 Hz, 1H), 2.25 – 2.11 (m, 9H), 2.02 (s, 3H), 1.86 (t, J = 12.2 Hz, 1H), 1.79 – 1.65 (m, 6H); ^{13}C NMR (101 MHz, D_2O): δ 174.8, 173.1, 158.0, 122.1, 119.2, 95.2, 70.4, 69.4, 68.9, 66.6, 61.1, 52.1, 42.1, 38.8, 35.1, 29.2, 22.1; ESI-MS: m/z Calc'd for $C_{25}H_{37}N_5O_{10}$: 567.3; found: 568.4 $[M + H]^+$.

Compound 1g:

FITC (19.4mg, 0.05 mmol) in 350 μL MeOH was added to the mixture of compound **4** (23.6 mg, 0.077 mmol) and $NaHCO_3$ (43 mg, 0.51 mmol) in water (0.7 mL), stirred in dark at room temperature until the starting material was consumed. MeOH was evaporated and the resulting residue was subjected to RP-HPLC purification to afford **1g** (29.2 mg, 84%) as an amorphous yellow solid.

^1H NMR (400 MHz, CD_3OD): δ 8.17 (d, *J* = 1.9 Hz, 1H), 7.79 (dd, *J* = 8.3, 2.1 Hz, 1H), 7.15 (d, *J* = 8.2 Hz, 1H), 6.71 – 6.59 (m, 4H), 6.50 – 6.47 (m, 2H), 4.12 – 3.81 (m, 5H), 3.78 – 3.65 (m, 1H), 3.51 (d, *J* = 12.4 Hz, 1H), 2.22 (dd, *J* = 12.8, 4.8 Hz, 1H), 2.01 (s, 3H), 1.85 (t, *J* = 12.1 Hz, 1H); ESI-MS: m/z Calc'd for $C_{32}H_{31}N_3O_{13}S$: 697.2; found: 698.3 $[M + H]^+$.

Compound 1g-CMP:

Compound **1g** (3.0 mg, 4.3 μM) and CTP (3.4 mg, 1.5 equiv.) were dissolved in 430 μL Tris·HCl (100 mM, pH 8.5) containing $MgCl_2$ (20 mM) and NmCSS R173A (0.1 mg/mL). The mixture was incubated at 37°C, which progress was monitored by TLC analysis. After completion, the reaction was centrifuged, and the supernatant was filtered through a 0.22 μm filter and purified by RP-HPLC, affording the product **1g-CMP** (3.3 mg, 76%).

^1H NMR (700 MHz, D_2O): δ 7.71 (d, *J* = 7.6 Hz, 1H), 7.63 (s, 2H), 7.36 (d, *J* = 8.0 Hz, 1H), 7.23 (d, *J* = 9.3 Hz, 1H), 7.20 (d, *J* = 9.2 Hz, 1H), 6.68 – 6.53 (m, 5H), 5.81 (d, *J* = 7.6 Hz, 1H), 5.68 (d, *J* = 4.4 Hz, 1H), 4.28 – 3.88 (m, 10H), 3.56 (t, *J* = 11.6 Hz, 1H), 3.41 (d, *J* = 9.4 Hz, 1H), 2.45 (dd, *J* = 13.6, 4.7 Hz, 1H), 2.03 (s, 3H), 1.62 (t, *J* = 12.1 Hz, 1H); ESI-MS: m/z Calc'd for $C_{41}H_{43}N_6O_{20}PS$: 1002.2; found:1001.6 $[M - H]^-$.

Compound 1h:

2-Naphthalenesulfonyl chloride (37 mg, 0.16 mmol) in 1.2 mL MeOH was added to the mixture of compound **4** (21 mg, 0.068 mmol) and $NaHCO_3$ (57 mg, 0.66 mmol) in 0.8 mL water. The reaction was stirred at room temperature until the starting material was consumed. MeOH was evaporated and the resulting residue was subjected to RP-HPLC purification to afford compound **1h** (30 mg, 88%).

^1H NMR (400 MHz, D_2O): δ 8.47 – 8.44 (m, 1H), 8.12 – 8.05 (m, 2H), 8.00 (d, *J* = 8.0 Hz, 1H), 7.87 – 7.78 (m, 1H), 7.75 – 7.66 (m, 2H), 4.04 – 3.81 (m, 3H), 3.71 – 3.62 (m, 1H), 3.43 (dd, *J* = 9.0, 1.2 Hz, 1H), 3.31 (dd, *J* = 13.8, 2.9 Hz, 1H), 3.02 – 2.95 (m, 1H), 2.23 (dd, *J* = 13.0, 4.9 Hz, 1H), 2.02 (s, 3H), 1.81 (t, *J* = 12.1 Hz, 1H); ^{13}C NMR (176 MHz, CD_3OD): δ 175.1, 171.7, 138.6, 136.2, 133.6, 130.5, 130.47, 130.2, 129.8, 129.13, 129.11, 129.0, 128.6, 123.52, 123.50, 96.6, 72.0, 71.9, 71.5, 71.4, 70.28, 67.8, 54.3, 54.2, 53.2, 47.8, 41.0, 40.7, 22.7, 22.26; ESI-MS: m/z Calc'd for $C_{21}H_{26}N_2O_{10}S$: 498.1; found: 499.4 $[M + H]^+$.

Compound 1j:

7-diethylaminocoumarin-3-carboxylic acid NHS ester[32] (28 mg, 0.078 mmol) in 1.8 mL THF was added to the mixture of compound **4** (21.5 mg, 0.07 mmol) and $NaHCO_3$ (60 mg, 0.71 mmol) in 0.6 mL water. The reaction was stirred at room temperature until the starting material was consumed. THF was evaporated and the resulting residue was subjected to RP-HPLC purification to afford **1j** (32 mg, 83%).

^1H NMR (400 MHz, CD_3OD): δ 8.61 (s, 1H), 7.53 (d, *J* = 9.0 Hz, 1H), 6.80 (dd, *J* = 9.0, 2.4 Hz, 1H), 6.54 (s, 1H), 4.08 – 3.81 (m, 6H), 3.53 –3.52 (m, 1H), 3.52 (dd, *J* = 14.0, 7.0 Hz, 4H),

2.20 (dd, J = 12.0, 4.0 Hz, 1H), 2.00 (s, 3H), 1.90 (t, J = 12.0 Hz, 1H), 1.23 (t, J = 7.0 Hz, 6H); ^{13}C NMR (101 MHz, CD_3OD): δ 159.7, 155.1, 149.9, 133.3, 112.2, 110.1, 97.8, 72.6, 68.9, 54.9, 49.0, 46.6, 42.0, 23.4, 13.4; ESI-MS: m/z Calc'd for $C_{25}H_{33}N_3O_{11}$: 551.2; found:552.4 [M + H]$^+$.

Compound 1k:

Biotin-N-hydroxysuccimide ester (10.6 mg, 0.031 mmol) in 150 μL DMSO was added to the mixture of compound **4** (11.5 mg, 0.037 mmol) and $NaHCO_3$ (26 mg, 0.31 mmol) in 300 μL H_2O. The reaction was stirred at room temperature until the starting material was consumed. Reaction mixture was lyophilized and re-dissolved in water, subjected to RP-HPLC purification to afford product **1k** (14.7 mg, 89%).

^{1}H NMR (400 MHz, D_2O): δ 4.62 (dd, J = 8.0, 4.8 Hz, 1H), 4.44 (dd, J = 7.9, 4.5 Hz, 1H), 4.06 – 3.87 (m, 2H), 3.84 – 3.76 (m, 1H), 3.65 – 3.54 (m, 1H), 3.43 (dd, J = 9.0, 1.2 Hz, 1H), 3.39 – 3.32 (m, 1H), 3.27 (dd, J = 14.2, 7.7 Hz, 1H), 3.01 (dd, J = 13.1, 4.9 Hz, 1H), 2.80 (d, J = 13.0 Hz, 1H), 2.73 (s, 1H), 2.30 (t, J = 7.3 Hz, 2H), 2.19 (d, J = 4.8 Hz, 1H), 2.06 (s, 3H), 1.81 – 1.56 (m, 5H), 1.49 – 1.38 (m, 2H); ^{13}C NMR (101 MHz, D_2O): δ 177.3, 176.6, 174.6, 165.4, 70.1, 69.8, 68.9, 67.2, 62.0, 60.2, 55.3, 52.3, 42.7, 39.7, 35.4, 27.8, 27.6, 25.1, 22.1; ESI-MS: m/z Calc'd for $C_{21}H_{34}N_4O_{10}S$: 534.2; found: 535.3 [M + H]$^+$.

Compound 1l:

Compound **24** (10 mg, 0.03 mmol), **25** (9.8 mg, 0.04 mmol), THPTA (4.7 mg, 0.01 mmol) and sodium ascorbate (3 mg, 0.03 mmol) were dissolved in the mixture of 300 μL DMF and 300 μL H_2O. Then, $CuSO_4{\cdot}5H_2O$ (1.5 mg, 6 μmol) was added to the reaction and stirred at room temperature until the starting material was consumed. DMF was evaporated and the resulting residue was subjected to RP-HPLC purification to afford **1l** (14 mg, 88%).

^{1}H NMR (400 MHz, Deuterium Oxide): δ 8.01 – 7.94 (m, 1H), 7.95 – 7.88 (m, 3H), 7.59 (m, , 2H), 7.56 – 7.49 (m, 2H), 5.56 (s, 2H), 4.01 – 3.95 (m, 1H), 3.95 – 3.92 (m, 1H), 3.90 – 3.82 (m, 1H), 3.76 (dd, J = 11.8, 2.7 Hz, 1H), 3.72 – 3.63 (m, 1H), 3.53 (dd, J = 11.8, 6.6 Hz, 1H), 3.35 (dd, J = 9.2, 1.1 Hz, 1H), 3.09 – 3.00 (m, 2H), 2.77 – 2.61 (m, 2H), 2.18 (dd, J = 13.0, 4.8 Hz, 1H), 1.75 (dd, J = 13.0, 11.3 Hz, 1H); ESI-MS: m/z Calc'd C26H31N5O10 for 573.21, found: 572.41 [M – H]$^-$.

Compound 1m:

4-Ethynylbenzoic acid NHS ester (33.5 mg, 0.14 mmol) in 0.8 mL THF was added to the mixture of compound **4** (28.3 mg, 0.09 mmol) and $NaHCO_3$ (62 mg, 0.74 mmol) in 0.2 mL H_2O. The reaction was stirred at room temperature until the starting material was consumed. THF was evaporated and the resulting residue was subjected to RP-HPLC purification to afford **1m** (32.9 mg, 82%).

^{1}H NMR (400 MHz, D_2O): δ 7.72 (d, J = 8.3 Hz, 2H), 7.62 (d, J = 8.1 Hz, 2H), 4.10 – 4.01 (m, 2H), 3.98 – 3.90 (m, 2H), 3.77 (dd, J = 14.2, 3.2 Hz, 1H), 3.65 (s, 1H), 3.55 – 3.47 (m, 2H), 2.28 (dd, J = 13.0, 4.8 Hz, 1H), 2.01 (s, 3H), 1.87 (t, J = 20.3 Hz, 1H); ^{13}C NMR (176 MHz, D_2O): δ 174.8, 173.2, 170.6, 133.8, 132.3, 127.2, 125.1, 95.3, 82.7, 80.4, 70.3, 69.5, 68.8, 66.6, 52.1, 43.3, 38.8, 22.0; ESI-MS: m/z Calc'd for $C_{20}H_{24}N_2O_9$: 436.2; found: 437.3 [M + H]$^+$.

Compound 1n:

4-Azidobenzoic acid NHS ester (18 mg, 0.09 mmol) in 1.2 mL THF was added to the mixture of compound **4** (22.7 mg, 0.07 mmol) and $NaHCO_3$ (62 mg, 0.74 mmol) in 0.8 mL H_2O. The reaction was stirred at room temperature until the starting material was consumed. THF was evaporated and the resulting residue was subjected to RP-HPLC purification to afford **1n** (26.7 mg, 80%).

^1H NMR (700 MHz, D_2O): δ 7.77 (d, J = 8.6 Hz, 2H), 7.18 (d, J = 8.6 Hz, 2H), 4.09 – 4.07 (m, 2H), 3.97 – 3.91 (m, 2H), 3.77 (dd, J = 14.1, 3.2 Hz, 1H), 3.54 – 3.50 (m, 2H), 2.31 (dd, J = 13.0, 4.9 Hz, 1H), 1.91 (s, 3H), 1.87 (t, J = 12.8 Hz, 1H); ^{13}C NMR (176 MHz, D_2O): δ 174.8, 173.5, 170.4, 143.7, 129.8, 128.9, 119.1, 95.4, 70.3, 69.5, 68.9, 66.6, 52.1, 43.3, 38.9, 22.0; ESI-MS: m/z Calc'd for $C_{18}H_{23}N_5O_9$: 453.2; found:454.3 $[M + H]^+$.

Compound 6

Cyclohexylacetylene (240 μL, 1.84 mmol), ethyl azidoacetate (212 μL, 1.84 mmol) and THPTA (320 mg, 0.74 mmol) were dissolved in 3 mL THF, followed by addition of sodium ascorbate (182 mg, 1.84 mmol) in the mixture of 330 μL *t*-BuOH and 660 μL H_2O. Then, $CuSO_4 \cdot 5H_2O$ (92 mg, 0.37 mmol) was added to the reaction and stirred at room temperature until the starting material was consumed. The reaction was extracted with 150 mL DCM. After concentration, the residue was purified by flash column chromatography on silica gel (PE/EA = 4) to afford compound **6** (416 mg, 95%).

^1H NMR (400 MHz, $CDCl_3$): δ 7.37 (s, 1H), 5.10 (s, 2H), 4.24 (dd, J = 14.0, 7.0 Hz, 2H), 2.88 – 2.70 (m, 1H), 2.13 – 1.99 (m, 2H), 1.85 – 1.68 (m, 3H), 1.49 – 1.19 (m, 8H); ESI-MS: m/z Calc'd $C_{12}H_{19}N_3O_2$ for 237.2, found: 238.2 $[M + H]^+$.

Compound 7

NaOH (211 mg, 5.28 mmol) in 600 μL H_2O was added to the reaction of compound **6** (416 mg, 1.76 mmol) in 3 mL THF. The reaction was stirred at room temperature until the starting material was consumed. Then, the mixture was neutralized by addition of ion-exchange resin Amberlite IR-120 (H^+). After filtration, the solution was concentrated to afford compound **7** (415 mg, 88%).

^1H NMR (400 MHz, $CDCl_3$): δ 7.54 (s, 1H), 5.15 (s, 2H), 2.84 – 2.73 (m, 1H), 2.09 – 1.98 (m, 2H), 1.84 – 1.64 (m, 3H), 1.43 – 1.21 (m, 5H); ESI-MS: m/z Calc'd $C_{10}H_{15}N_3O_2$ for 209.2, found: 208.2 $[M - H]^-$.

Compound 9

Compound **8**[29] (500 mg, 1.10 mmol) was dissolved in the mixture of 4 mL MeOH and 1 mL MsOH, stirred at 60°C until the starting material was consumed. The mixture was neutralized by $NaHCO_3$ powder. After filtration, the reaction was concentrated and the residue was purified by flash column chromatography on silica gel (DCM/MeOH = 10) to afford compound **9** (400 mg, 95%).

^1H NMR (400 MHz, CD_3OD): δ 7.44 (d, J = 8.1 Hz, 1H), 7.19 (d, J = 7.9 Hz, 1H), 4.44 (dd, J = 10.0, 1.3 Hz, 1H), 3.96 – 3.86 (m, 2H), 3.82 (dd, J = 8.4, 1.4 Hz, 1H), 3.67 – 3.60 (m, 1H), 3.59 (s, 3H), 3.44 (dd, J = 12.9, 6.1 Hz, 1H), 2.83 (t, J = 9.9 Hz, 1H), 2.62 (dd, J = 13.6, 4.6 Hz, 1H), 2.35 (s, 3H), 1.96 – 1.86 (m, 1H); ESI-MS: m/z Calc'd $C_{17}H_{24}N_4O_6S$ for 412.1, found: 413.2 $[M + H]^+$.

Compound 10

At 0°C, compound **9** (200 mg, 0.49 mmol) was dissolved in 1 mL DMF (anhydr.), followed by addition of compound **7** (120 mg, 0.59 mmol), HATU (280 mg, 0.74 mmol), and DIEA (170 μL, 0.98 mmol) in 1 mL DMF (anhydr.). The mixture was warmed to room temperature and stirred for 2 h and then, concentrated. The resulting residue was purified by flash column chromatography on silica gel (DCM/MeOH = 20) to afford compound **10** (227 mg, 77%).

^{1}H NMR (400 MHz, CD_3OD): δ 7.77 (s, 1H), 7.43 (d, *J* = 8.1 Hz, 2H), 7.19 (d, *J* = 7.9 Hz, 2H), 5.19 (s, 2H), 4.56 (dd, *J* = 10.5, 1.1 Hz, 1H), 4.22 – 4.13 (m, 1H), 3.94 (t, *J* = 10.2 Hz, 1H), 3.90 – 3.84 (m, 1H), 3.64 – 3.52 (m, 5H), 3.41 (dd, *J* = 12.8, 6.3 Hz, 1H), 2.83 – 2.73 (m, 1H), 2.69 (dd, *J* = 13.7, 4.7 Hz, 1H), 2.35 (s, 3H), 2.10 – 1.93 (m, 3H), 1.89 – 1.71 (m, 3H), 1.53 – 1.25 (m, 5H); ESI-MS: m/z Calc'd $C_{27}H_{37}N_7O_7S$ for 603.3, found: 604.4 $[M + H]^+$.

Compound 11

Compound **10** (100 mg, 0.17 mmol) and LiOH (20 mg, 0.85 mmol) were dissolved in 600 μL MeOH and 200 μL H_2O, stirred at room temperature for 2 h. The mixture was neutralized by addition of ion-exchange resin Amberlite IR-120 (H^+). After filtration, the solution was concentrated to afford acid **11a** (97 mg, 98%).

Compound **11a** and NBS (86 mg, 0.48 mmol) were dissolved in 600 μL acetone and 200 μL H_2O. After stirred at room temperature for 0.5 h, the mixture was neutralized by addition of $NaHCO_3$ powder. After filtration, the solution was concentrated and the residue was purified by G-15 beads to afford compound **11** (68 mg, 85%).

^{1}H NMR (600 MHz, D_2O): δ 7.96 (s, 1H), 5.32 (s, 2H), 4.19 – 4.09 (m, 2H), 3.97 (t, *J* = 10.3 Hz, 1H), 3.91 – 3.86 (m, 1H), 3.61 – 3.44 (m, 3H) , 2.85 – 2.77 (m, 1H), 2.31 (dd, *J* = 13.1, 4.9 Hz, 1H), 2.03 – 1.95 (m, 2H), 1.87 (t, *J* = 12.0 Hz, 1H), 1.80 – 1.64 (m, 3H), 1.48 – 1.21 (m, 5H); ^{13}C NMR (125 MHz, D_2O): δ 173.2, 167.9, 152.8, 124.4, 95.3, 69.9, 68.9, 68.6, 66.5, 53.8, 52.7, 52.4, 38.8, 34.0, 32.0, 25.4, 25.3; ESI-MS: m/z Calc'd $C_{19}H_{29}N_7O_8$ for 483.2, found: 484.3 $[M + H]^+$.

Compound 12

To a solution of compound **11** (68 mg, 0.14 mmol) in 1 mL H_2O, PMe_3 (1 M in THF, 700 μL, 0.70 mmol) was added. After stirred at room temperature for 2 h, the reaction was concentrated and the resulting residue was purified by G-15 beads to afford compound **12** (60 mg, 93%).

^{1}H NMR (700 MHz, D_2O): δ 7.87 (s, 1H), 5.27 (s, 2H), 4.16 – 4.05 (m, 2H), 3.99 – 3.88 (m, 2H), 3.53 (dd, *J* = 8.6, 1.2 Hz, 1H), 3.36 (dd, *J* = 13.1, 3.2 Hz, 1H), 2.96 (dd, *J* = 13.1, 9.5 Hz, 1H), 2.81 – 2.74 (m, 1H), 2.30 (dd, *J* = 13.2, 5.0 Hz, 1H), 2.00 – 1.93 (m, 2H), 1.86 (t, *J* = 12.3 Hz, 1H), 1.78 – 1.65 (m, 3H), 1.47 – 1.19 (m, 5H); ^{13}C NMR (176 MHz, D_2O): δ 176.3, 168.5, 154.2, 123.4, 96.3, 70.4, 69.8, 66.9, 66.8, 52.4, 52.0, 42.6, 39.3, 34.4, 32.3, 25.5, 25.4; ESI-MS: m/z Calc'd $C_{19}H_{31}N_5O_8$ for 457.2, found: 458.3 $[M + H]^+$.

Compound 19:

N-tert-Butoxycarbonyl-4-aminomethylbenzoic acid (400 mg, 1.59 mmol) in 3 mL THF was treated with *N*-hydroxy succinimide (220 mg, 1.91 mmol) and DCC (400 mg, 1.94 mmol) in 1 mL DCM. The reaction was stirred at room temperature overnight. White precipitate was filtrated

and the filtrate was evaporated under vacuum to provide NHS-activated product **18**. The crude **18** was used without further purification.

To a solution of compound **16**[30] (150mg, 0.19 mmol) in 2 mL water, PMe_3 (1 M in THF, 0.3 mL) was added. The mixture was stirred at room temperature for 1 h and subjected to purify by G-15 to afford desired product **17** (146 mg, 100%). Then, compound **18** (2.0 equiv.) in 1 mL THF was added to a mixture of compound **17** and $NaHCO_3$ in 0.9 mL water, stirred at room temperature overnight. THF was evaporated and the resulting residue was subjected to a C18 Sep Pak column and eluted with 40% MeOH to afford white solid **19** (152 mg, 80%).

^{1}H NMR (400 MHz, D_2O): δ 7.74 (d, J = 8.1 Hz, 2H), 7.42 (d, J = 8.0 Hz, 2H), 4.71 (d, J = 8.2 Hz, 1H), 4.48 (d, J = 8.1 Hz, 1H), 4.46 (d, J = 7.8 Hz, 1H), 4.43 (d, J = 7.8 Hz, 1H), 4.31 (s, 2H), 4.16 (d, J = 3.2 Hz, 1H), 4.06 – 3.92 (m, 4H), 3.89 – 3.47 (m, 23H), 3.32 (t, J = 8.4 Hz, 1H) , 2.04 (s, 3H), 2.01 – 1.91 (m, 2H), 1.44 (s, 9H); ^{13}C NMR (176 MHz, D_2O): δ 175.0, 170.6, 158.3, 127.3, 127.0, 103.0, 102.9, 102.7, 102.0, 82.0, 78.4, 78.2, 75.3, 74.9, 74.8, 74.5, 74.4, 74.3, 72.8, 72.5, 72.2, 71.0, 69.9, 68.5, 68.3, 67.9, 61.0, 60.9, 60.1, 59.9, 55.2, 43.4, 36.91 28.5, 27.6, 22.2; ESI-MS: m/z Calc'd for $C_{42}H_{67}N_3O_{24}$: 997.4; found: 998.5 $[M + H]^+$.

Compound 20:

TFA (0.2 mL) was added to a solution of compound **19** (152 mg, 0.15 mmol) in 2 mL water. The mixture was stirred at room temperature, followed by loading to a C18 Sep Pak column, eluted with 5% MeOH to afford white solid **20** (125 mg, 92%).

^{1}H NMR (400 MHz, D_2O): δ 7.82 (d, J = 8.3 Hz, 2H), 7.57 (d, J = 8.2 Hz, 2H), 4.71 (d, J = 8.2 Hz, 1H), 4.55 – 4.47 (m, 2H), 4.44 (d, J = 7.8 Hz, 1H), 4.26 (s, 2H), 4.17 (d, J = 3.2 Hz, 1H), 4.05 – 3.94 (m, 4H), 3.80 – 3.68 (m, 14H), 3.68 – 3.50 (m, 9H), 3.31 (t, J = 6.4 Hz,1H), 2.04 (s, 3H), 2.01 – 1.94 (m, 2H); ^{13}C NMR (176 MHz, D_2O): δ 174.9, 170.3, 162.9, 136.3, 129.0, 127.8, 102.9, 102.85, 102.7, 102.1, 82.0, 78.4, 78.2, 75.3, 74.9, 74.8, 74.5, 74.4 72.8, 72.5, 72.2, 71.0, 69.9, 68.5, 68.3, 68.0, 61.0, 60.95, 60.1, 55.2, 42.6, 37.0, 28.4, 22.2; ESI-MS: m/z Calc'd for $C_{37}H_{59}N_3O_{22}$: 897.4; found: 898. 5 $[M + H]^+$.

Compound 23

Compound **21**[29] (2.33 mmol) was dissolved in the mixture of 10 mL MeOH and 0.5 mL MsOH, stirred at 60°C until the starting material was consumed. The mixture was neutralized by $NaHCO_3$ powder. After filtration, the reaction was concentrated and the residue was dissolved in 4 mL DMF (anhydr.), followed by addition of 4-pentynoic acid (274 mg, 2.8 mmol), HATU (1.06 g, 2.8 mmol), and DIEA (770 μL, 4.66 mmol) in 1 mL DMF (anhydr.). The mixture was warmed to room temperature and stirred for 2 h and then, concentrated. The resulting residue was purified by flash column chromatography on silica gel (DCM/MeOH = 30) to afford compound **23** (916 mg, 87% for two steps).

^{1}H NMR (400 MHz, Methanol-d4): δ 7.49 (d, J = 7.8 Hz, 2H), 7.19 (d, J = 7.7 Hz, 2H), 4.53 (d, J = 10.4 Hz, 1H), 4.19 – 4.08 (m, 1H), 3.93 (t, J = 10.2 Hz, 1H), 3.88 - 3.77 (m, 2H), 3.72 – 3.64 (m, 2H), 3.57 (s, 3H), 3.37 (s, 1H), 2.69 (dd, J = 13.6, 4.6 Hz, 1H), 2.53 (s, 3H), 2.42 – 2.30 (m, 4H), 1.97 (t, J = 12.1Hz, 1H); ^{13}C NMR (101 MHz, Deuterium Oxide): δ 175.6, 142.4, 139.3, 136.2, 134.4, 129.4, 125.3, 83.6, 70.4, 70.2, 69.7, 69.2, 68.3, 63.2 34.6, 20.5, 14.9, 14.3; ESI-MS: m/z calc. for C22H29NO8S: 467.16; found 485.23 $[M+NH_4]^+$.

Compound 24

Compound **23** (600 mg, 1.28 mmol) and LiOH (92 mg, 3.84 mmol) were dissolved in 25 mL THF and 5 mL H_2O, stirred at room temperature for 1 h. The mixture was neutralized by addition of ion-exchange resin Amberlite IR-120 (H^+). After filtration, the solution was concentrated. The residue was dissolved in 3.6 mL acetone and 0.6 mL H_2O, followed by addition of NBS (273 mg, 1.54 mmol). After stirred at room temperature for 1 h, the mixture was neutralized by addition of $NaHCO_3$ powder. After filtration, the solution was concentrated and the residue was purified by G-15 beads to afford compound **23** (359 mg, 81% for two steps).

^{1}H NMR (400 MHz, Deuterium Oxide): δ 4.04 – 3.86 (m, 3H), 3.79 (d, J = 11.7 Hz, 2H), 3.71 (t, J = 7.4 Hz, 1H), 3.63 – 3.50 (m, 1H), 2.46 (d, J = 6.8 Hz, 4H), 2.36 (d, J = 8.7 Hz, 1H), 2.17 (dd, J = 12.9, 4.7 Hz, 1H), 1.77 (t, J = 12.1 Hz, 1H); ^{13}C NMR (101 MHz, Deuterium Oxide): δ 96.4, 83.6, 70.4, 70.4, 70.2, 68.6, 67.1, 63.3, 52.3, 39.4, 34.7, 14.6; ESI-MS: m/z calc. for C14H21NO9: 347.12; found 348.10 $[M+H]^+$.

Compound A1:

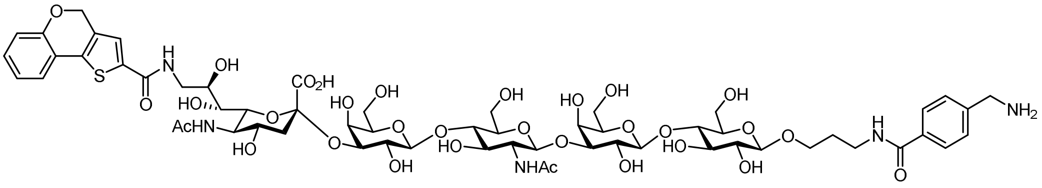

Prepared using General Procedure for "one-pot two-enzyme" approach.

^{1}H NMR (700 MHz, D_2O): δ 7.80 (d, J = 8.2 Hz, 2H), 7.55 (d, J = 8.2 Hz, 2H), 7.39 – 7.33 (m, 2H), 7.27 (t, J = 7.6 Hz, 1H), 7.04 (t, J = 7.5 Hz, 1H), 6.96 (d, J = 8.1 Hz, 1H), 5.22 – 5.17 (m, 2H), 4.76 (d, J = 8.2 Hz, 1H), 4.59 (d, J = 8.2 Hz, 1H), 4.49 (d, J = 8.0 Hz, 1H), 4.46 (d, J = 7.8 Hz, 1H), 4.37 – 4.31 (m, 1H), 4.25 (s, 2H), 4.11 (dd, J = 9.8, 3.2 Hz, 1H), 4.06 – 4.00 (m, 3H), 3.99 – 3.86 (m, 3H), 3.82 – 3.49 (m, 26H), 3.48 – 3.41 (m, 2H), 3.31 (t, J = 8.0 Hz, 1H), 2.78 (dd, J = 12.5, 4.6 Hz, 1H), 2.08 – 1.93 (m, 8H), 1.83 (t, J = 12.2 Hz, 1H); ^{13}C NMR (176 MHz, D_2O): δ 175.0, 174.8, 170.2, 163.9, 151.7, 136.3, 136.0, 134.5, 132.2, 129.0, 127.8, 126.5, 123.8, 122.8 116.7, 102.9, 102.7, 102.4, 102.0, 99.6, 81.9, 78.5, 77.7, 75.2, 74.8, 74.7, 74.5, 74.4, 72.8, 72.0, 70.3, 70.0, 69.9, 69.32, 68.29, 68.0, 65.7, 61.0, 60.1, 59.8, 55.1, 51.7, 43.0, 42.6, 39.8, 37.0, 28.4, 22.2, 22.0; ESI-MS: m/z Calc'd for $C_{60}H_{83}N_5O_{31}S$: 1401.5; found:1402.8 $[M + H]^+$.

Compound A3:

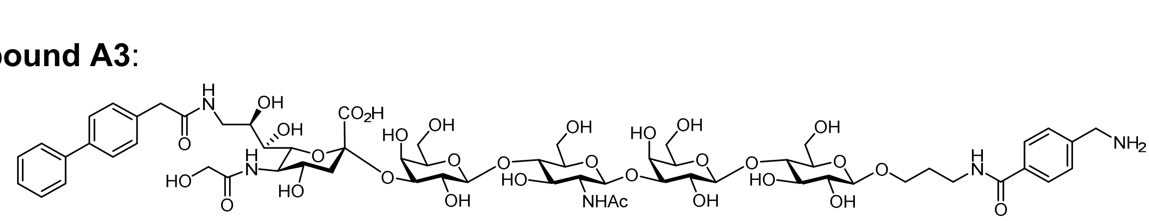

Prepared using General Procedure for "one-pot two-enzyme" approach.

^{1}H NMR (700 MHz, D_2O): δ 7.83 – 7.79 (m, 2H), 7.77 – 7.72 (m, 4H), 7.58 – 7.53 (m, 4H), 7.49 – 7.44 (m, 3H), 4.63 (d, J = 8.4 Hz, 1H), 4.50 (d, J = 8.1 Hz, 1H), 4.39 (d, J = 7.8 Hz, 1H), 4.32 (d, J = 7.8 Hz, 1H), 4.22 (s, 2H), 4.11 – 4.10 (m, 2H), 4.08 – 4.01 (m, 2H), 3.98 – 3.91 (m, 4H), 3.84 – 3.46 (m, 30H), 3.31 (t, J = 8.1 Hz, 1H), 2.79 (dd, J = 12.5, 4.6 Hz, 1H), 2.04 (s, 3H), 1.98 – 1,96 (m, 2H), 1.80 (t, J = 12.2 Hz, 1H); ^{13}C NMR (176 MHz, D_2O): δ 175.7, 174.8, 174.6, 173.5, 170.3, 163.1, 139.9, 139.2, 136.3, 134.7, 134.5, 129.7, 129.3, 129.0, 127.8, 127.2, 126.8, 102.9, 102.7, 102.5, 102.1, 99.6, 81.9, 78.5, 78.0, 75.6, 75.0, 74.9, 74.8, 74.5, 74.4, 72.8, 72.6,

71.9, 70.0, 69.9, 69.3, 68.3, 68.0, 67.9, 67.2, 61.0, 60.7, 60.1, 59.7, 55.1, 51.3, 42.6, 42.5, 42.0, 39.8, 37.0, 28.4, 22.2; ESI-MS: m/z calc. for $C_{62}H_{87}N_5O_{31}$: 1397.5; found:1398.7 $[M + H]^+$.

Compound A4:

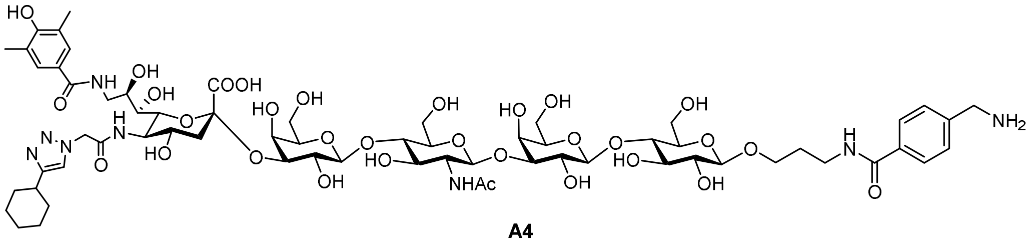

A4

Prepared using General Procedure for "one-pot two-enzyme" approach.

^{1}H NMR (700 MHz, D_2O): δ 7.82 (d, *J* = 8.3 Hz, 2H), 7.60 – 7.52 (m, 3H), 7.48 (s, 2H), 5.22 (d, *J* = 16.6 Hz, 1H), 5.13 (d, *J* = 16.6 Hz, 1H), 4.67 (d, *J* = 8.1 Hz, 1H), 4.50 (d, *J* = 7.9 Hz, 2H), 4.44 (d, *J* = 7.8 Hz, 1H), 4.26 (s, 2H), 4.16 – 3.90 (m, 6H), 3.89 – 3.46 (m, 28H), 3.32 (t, *J* = 8.4 Hz, 1H), 2.78 (dd, *J* = 12.3, 4.6 Hz, 1H), 2.67 – 2.55 (m, 1H), 2.25 (s, 6H), 2.04 (s, 3H), 2.01 – 1.94 (m, 2H), 1.93 – 1.63 (m, 6H), 1.43 – 1.14 (m, 5H); ESI-MS: m/z Calc'd for $C_{65}H_{96}N_8O_{31}$: 1484.6; found:1483.8 $[M - H]^-$.

Compound A6:

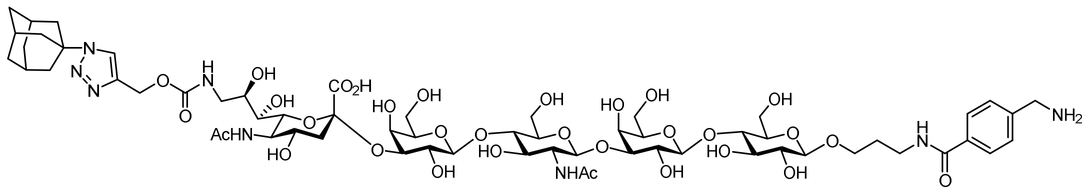

A6

Prepared using General Procedure for "one-pot two-enzyme" approach.

^{1}H NMR (700 MHz, D_2O): δ 7.81 (d, *J* = 8.0 Hz, 2H), 7.56 (d, *J* = 8.0 Hz, 2H), 4.70 (d, *J* = 8.4 Hz, 1H), 5.21 (brs, 1H), 4.70 (d, *J* = 8.4 Hz, 1H), 4.54 (d, *J* = 7.8 Hz, 1H), 4.50 (d, *J* = 8.0 Hz, 1H), 4.43 (d, *J* = 7.8 Hz, 1H), 4.26 (s, 2H), 4.15 (d, *J* = 3.3 Hz, 1H), 4.09 (d, *J* = 9.9 Hz, 1H), 4.04 – 4.01 (m, 1H), 3.98 – 3.48 (m, 32H), 3.31 (t, *J* = 8.6 Hz, 1H), 3.23 – 3.18 (m, 1H), 2.79 – 2.72 (m, 1H), 2.28 – 2.18 (m, 9H), 2.03 (s, 3H), 2.02 (s, 3H), 1.98 – 1.96 (m, 2H), 1.85 – 1.75 (m, 7H); ^{13}C NMR (176 MHz, D_2O): δ 170.3, 136.3, 134.5, 127.8, 102.9, 102.8, 102.5, 102.1, 82.0, 78.4, 77.9, 75.6, 75.2, 74.9, 74.8, 74.6, 74.4, 72.8, 72.1, 70.0, 69.3, 68.3, 68.2, 68.0, 67.5, 61.0, 60.1, 59.8, 55.2, 51.7, 42.2, 35.2, 29.2, 22.2, 22.0; ESI-MS: m/z calc. for $C_{62}H_{94}N_8O_{31}$: 1446.6; found:1447.9 $[M + H]^+$.

Compound A7:

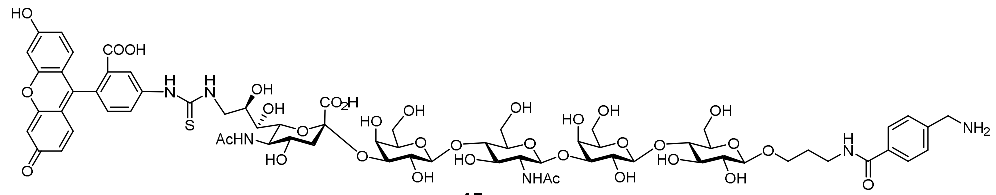

A7

Prepared using General Procedure for "one-pot two-enzyme" approach.

^{1}H NMR (700 MHz, D_2O): δ 8.14 (s, 1H), 7.78 (d, *J* = 7.9 Hz, 2H), 7.69 (s, 1H), 7.53 (d, *J* = 8.0 Hz, 2H), 7.08 (s, 3H), 6.78 (s, 4H), 4.67 (d, *J* = 8.1 Hz, 1H), 4.54 (d, *J* = 7.8 Hz, 1H), 4.45

(d, J = 7.9 Hz, 1H), 4.35 (d, J = 8.0 Hz, 1H), 4.23 (s, 2H), 4.19 – 4.04 (m, 4H), 4.02 – 3.87 (m, 5H), 3.86 – 3.46 (m, 25H), 3.29 (t, J = 8.6 Hz, 1H), 2.79 (s, 1H), 2.03 (s, 3H), 2.02 (s, 3H) 1.95 – 1.92 (m, 2H), 1.84 (t, J = 12.1 Hz, 1H); ESI-MS: m/z calc. for $C_{69}H_{88}N_6O_{34}S$: 1577.5; found:1578.8 $[M + H]^+$.

Compound A8:

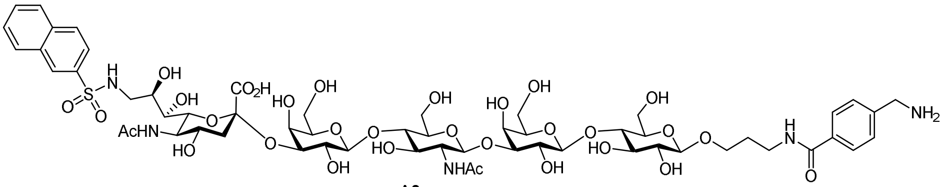

A8

Prepared using General Procedure for "one-pot two-enzyme" approach.

^{1}H NMR (700 MHz, D_2O): δ 8.52 (s, 1H), 8.15 (d, J = 8.7 Hz, 1H), 8.12 (d, J = 8.2 Hz, 1H), 8.06 (d, J = 8.2 Hz, 1H), 7.91 – 7.86 (m, 1H), 7.82 – 7.70 (m, 4H), 7.55 (d, J = 7.9 Hz, 2H), 4.67 (d, J = 8.4 Hz, 1H), 4.49 (d, J = 7.9 Hz, 1H), 4.42 (d, J = 7.8 Hz, 1H), 4.27 (d, J = 7.8 Hz, 1H), 4.25 (s, 2H), 4.14 (d, J = 3.3 Hz, 1H), 4.07 – 3.47 (m, 31H), 3.38 (dd, J = 14.0, 2.8 Hz, 1H), 3.31 (t, J = 8.6 Hz, 1H), 3.07 (dd, J = 13.7, 7.6 Hz, 1H), 2.74 (dd, J = 12.7, 4.6 Hz, 1H), 2.07 – 1.92 (m, 8H), 1.79 (t, J = 12.2 Hz, 1H); ^{13}C NMR (176 MHz, D_2O): δ 175.0, 174.9, 136.6, 131.8, 129.9, 129.4, 129.2, 129.0, 128.2, 128.0, 127.8, 121.6, 102.9, 102.7, 102.6, 102.1, 81.9, 78.4, 78.2, 75.6, 75.1, 74.9, 74.8, 74.5, 74.4, 72.8, 72.0, 70.5, 69.9, 69.24, 69.16, 68.1, 68.0, 67.3, 61.0, 60.9, 60.1, 60.0, 55.1, 51.7, 45.1, 42.6, 39.7, 37.0, 28.4, 22.2, 22.0; ESI-MS: m/z calc. for $C_{58}H_{83}N_5O_{31}S$: 1377.5; found:1378.4 $[M + H]^+$.

Compound A10:

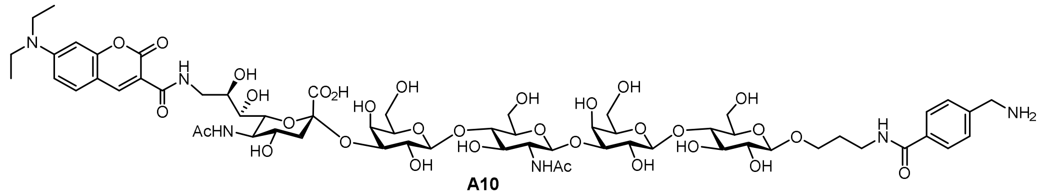

A10

Prepared using General Procedure for "one-pot two-enzyme" approach.

^{1}H NMR (700 MHz, D_2O): δ 8.34 (s, 1H), 7.82 – 7.76 (m, 2H), 7.55 (d, J = 8.1 Hz, 2H), 7.37 (d, J = 9.0 Hz, 1H), 6.71 (dd, J = 9.1, 2.3 Hz, 1H), 6.41 (d, J = 2.3 Hz, 1H), 4.65 (d, J = 8.2 Hz, 1H), 4.50 (d, J = 7.8 Hz, 1H), 4.49 (d, J = 8.0 Hz, 1H), 4.40 (d, J = 7.8 Hz, 1H), 4.25 (s, 2H), 4.15 – 4.10 (m, 1H), 4.08 – 3.99 (m, 3H), 3.99 – 3.82 (m, 5H), 3.81 – 3.40 (m, 29H), 3.30 (t, J = 7.9 Hz, 1H), 2.77 (dd, J = 12.4, 4.6 Hz, 1H), 2.03 (s, 3H), 2.02 (s, 3H), 1.99 – 1.95 (m, 2H), 1.81 (t, J = 12.1 Hz, 1H), 1.22 – 1.15 (m, 6H); ^{13}C NMR (176 MHz, D_2O): δ 174.8, 173.8, 163.7, 153.5, 129.0, 127.8, 111.0, 107.9, 106.7, 102.9, 102.7 102.5, 102.1, 99.8, 82.0 78.4, 75.2, 74.9, 74.8, 74.6, 74.4, 72.8, 72.1, 69.9, 68.3, 68.0, 61.0, 60.1, 55.1, 51.7, 45.0, 42.6, 42.3, 37.0, 28.4, 22.2, 22.1, 11.8; ESI-MS: m/z calc. for $C_{69}H_{88}N_6O_{34}S$: 1430.6; found:1431.8 $[M + H]^+$.

Compound A12

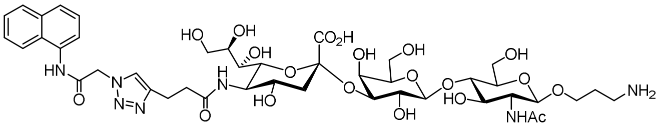

Prepared using General Procedure for "one-pot two-enzyme" approach.

^{1}H NMR (400 MHz, Deuterium Oxide): δ 8.00 (dd, J = 6.8, 2.7 Hz, 1H), 7.95 (m, 3H), 7.66 – 7.59 (m, 2H), 7.59 – 7.51 (m, 2H), 5.58 (s, 2H), 4.52 (d, J = 7.9 Hz, 1H), 4.49 (d, J = 7.8 Hz, 1H), 4.07 (dd, J = 9.9, 3.1 Hz, 1H), 4.00 (m, 2H), 3.92 (d, J = 3.2 Hz, 1H), 3.88 – 3.50 (m, 16H), 3.44 – 3.35 (m, 1H), 3.12 – 3.02 (m, 4H), 2.75 - 2.64 (m, 3H), 2.03 (s, 3H), 1.97 – 1.89 (m, 2H), 1.73 (t, J = 12.1 Hz, 1H); ESI-MS: m/z Calc'd C43H61N7O20 for 995.40, found: 994.63 $[M – H]^-$.

Compound D1:

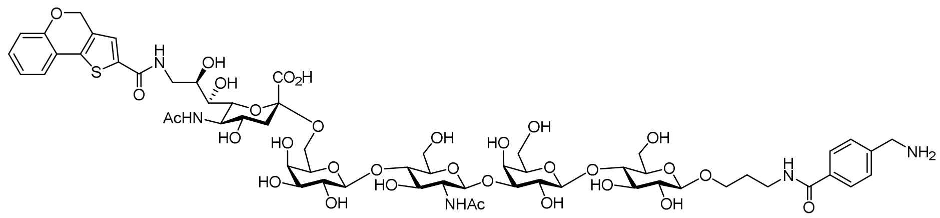

Prepared using General Procedure for "one-pot two-enzyme" approach.

^{1}H NMR (700 MHz, D_2O): δ 7.80 (d, J = 6.8 Hz, 2H), 7.54 (d, J = 7.8 Hz, 2H), 7.31 – 7.29 (m, 2H), 7.24 (t, J = 7.8 Hz, 1H), 7.00 (t, J = 7.4 Hz, 1H), 6.91 (d, J = 8.1 Hz, 1H), 5.16 (s, 2H), 4.61 (d, J = 8.3 Hz, 1H), 4.48 (d, J = 7.8 Hz, 1H), 4.42 – 4.39 (m, 2H), 4.24 (s, 2H), 4.07 –3.45 (m, 34H), 3.31 – 3.29 (m, 1H), 2.68 (d, J = 8.0 Hz, 1H), 2.06 (s, 3H), 2.01 (s, 3H), 1.96 – 1.95 (m, 2H), 1.76 (t, J = 11.2 Hz, 1H); ^{13}C NMR (176 MHz, D_2O): δ 174.9, 174.8, 172.5, 170.2, 163.8, 151.6, 136.3, 135.9, 134.4, 132.1, 129.0, 127.8, 126.5, 123.7, 122.7, 119.0, 116.5, 103.5, 103.0, 102.5, 102.1, 99.7, 82.1, 80.5, 78.5, 74.9, 74.8, 74.4, 72.8, 69.9, 68.0, 65.7, 63.6, 61.0, 60.1, 54.8, 51.8, 42.6, 39.7, 37.0, 28.4, 22.3, 22.1; ESI-MS: m/z calc. for $C_{60}H_{83}N_5O_{31}S$: 1401.5; found:1402.6 $[M + H]^+$.

Compound D3:

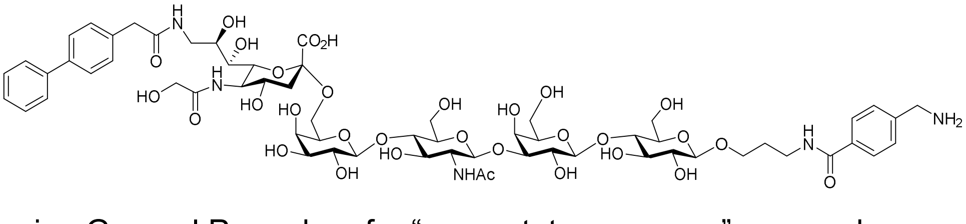

Prepared using General Procedure for "one-pot, two-enzyme" approach.

^{1}H NMR (400 MHz, D_2O): δ 7.80 (d, J = 8.3, 2H), 7.74 – 7.69 (m, 4H), 7.56 – 7.53 (m, 4H), 7.44 – 7.42 (m, 3H), 4.67 (d, J = 8.1 Hz, 1H), 4.48 (d, J = 8.0 Hz, 1H), 4.43 (d, J = 8.0 Hz, 1H), 4.41 (d, J = 8.0 Hz, 1H) , 4.25 (s, 2H), 4.12 (d, J = 3.4 Hz, 1H), 4.07 (d, J = 1.2 Hz, 2H), 4.05 – 3.87 (m, 8H), 3.85 – 3.71 (m, 9H), 3.71 – 3.61 (m, 8H), 3.61 – 3.50 (m, 8H), 3.43 – 3.41 (m, 1H), 3.39 – 3.28 (m, 2H), 2.76 – 2.64 (m, 1H), 2.03 (s, 3H), 2.00 – 1.92 (m, 2H), 1.73 (t, J = 12.2 Hz, 1H); ^{13}C NMR (176 MHz, D_2O): δ 175.6, 139.3, 136.3, 134.5, 129.8, 129.2, 129.0, 127.8, 127.2, 126.8, 103.4, 102.9, 102.6, 102.1, 82.0, 80.4, 78.4, 74.9, 74.7, 74.4, 74.27, 73.6 , 72.8, 72.4, 72.2, 72.1, 70.7, 70.0, 69.93, 69.85, 68.5, 68.3, 68.0, 67.9, 63.6, 61.0, 60.1, 60.0, 54.9, 51.6, 42.6, 42.3, 41.9, 40.1, 37.0, 28.4, 22.3; ESI-MS: m/z calc. for $C_{62}H_{87}N_5O_{31}$: 1397.5; found: 1398.6 $[M + H]^+$.

Compound D4:

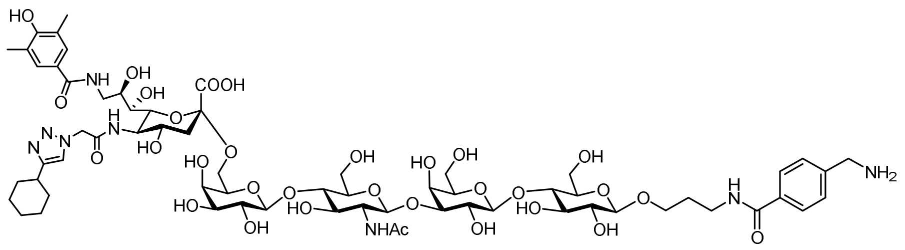

D4

Prepared using General Procedure for "one-pot two-enzyme" approach.

^{1}H NMR (700 MHz, D_2O): δ 7.78 (d, *J* = 7.9, 5.7 Hz, 2H), 7.53 (d, *J* = 7.9 Hz, 2H), 7.48 – 7.43 (m, 3H), 5.17 (d, *J* = 16.6, Hz, 1H), 5.07 (d, *J* = 16.6, Hz, 1H), 4.65 (d, *J* = 8.0 Hz, 1H), 4.46 (d, *J* = 8.1 Hz, 1H), 4.41 (d, *J* = 7.9 Hz, 1H), 4.39 (d, *J* = 8.0 Hz, 1H), 4.22 (s, 2H), 4.11 (d, *J* = 3.3 Hz, 1H), 4.06 – 3.94 (m, 3H), 3.93 – 3.85 (m, 5H), 3.81 – 3.45 (m, 25H), 3.27 (t, *J* = 8.5 Hz, 1H), 2.64 (dd, *J* = 12.5, 4.6 Hz, 1H), 2.59 – 2.51 (m, 1H), 2.21 (s, 6H), 2.02 – 1.93 (m, 4H), 1.88 – 1.78 (m, 2H), 1.73 – 1.59 (m, 5H), 1.37 – 1.13 (m, 5H); ESI-MS: m/z Calc'd for $C_{65}H_{96}N_8O_{31}$: 1484.6; found:1483.5 $[M - H]^-$.

Compound D6:

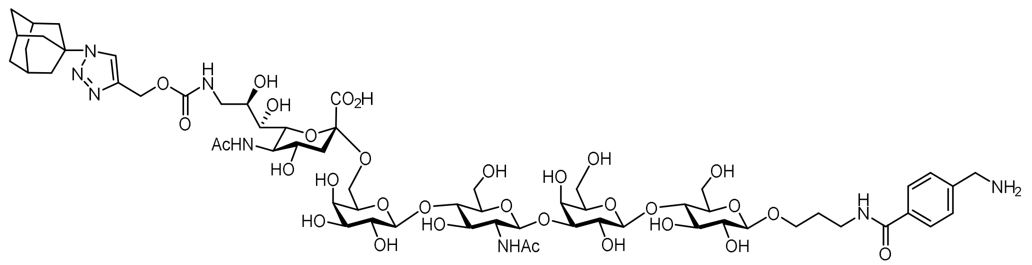

Prepared using General Procedure for "one-pot two-enzyme" approach.

^{1}H NMR (700 MHz, D_2O): δ 8.14 (s, 1H), 7.86 – 7.79 (m, 2H), 7.63 – 7.54 (m, 2H), 5.21 – 5.19 (m, 2H), 4.74 (d, *J* = 7.7 Hz, 1H), 4.50 (d, *J* = 8.0 Hz, 1H), 4.46 – 4.43 (m, 2H), 4.26 (s, 2H), 4.16 (d, *J* = 3.3 Hz, 1H), 4.04 – 4.03 (m, 1H), 3.99 – 3.52 (m, 30H), 3.49 – 3.43 (m, 1H), 3.32 (t, *J* = 8.6 Hz, 1H), 3.27 (dd, *J* = 14.1, 7.2 Hz, 1H), 2.67 (dd, *J* = 12.3, 4.7 Hz, 1H), 2.28 – 2.18 (m, 9H), 2.05 (s, 3H), 2.02 (s, 3H), 2.00 – 1.94 (m, 2H), 1.84 – 1.75 (m, 7H); ^{13}C NMR (176 MHz, D_2O): δ 174.9, 172.6, 170.3, 158.1, 142.4, 136.3, 134.5, 129.0, 127.8, 122.0, 103.5, 103.0, 102.6, 102.1, 99.8, 82.0, 80.5, 78.4, 74.9, 74.8, 74.4, 74.3, 73.7, 72.8, 72.5, 72.4, 72.2, 70.7, 70.0, 69.7, 68.3, 68.0, 67.9, 61.0, 60.9, 60.11, 60.06, 57.8, 54.9, 51.8, 42.6, 42.2, 37.0, 35.2, 29.2, 28.4, 22.3, 22.0; ESI-MS: m/z calc. for $C_{62}H_{94}N_8O_{31}$: 1446.6; found: 1447.7 $[M + H]^+$.

Compound D7:

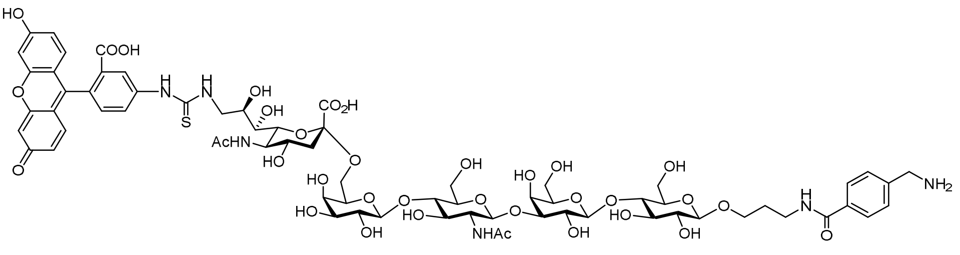

Prepared using General Procedure for "one-pot two-enzyme" approach.

^{1}H NMR (700 MHz, D_2O): δ 8.11 (s, 1H), 7.76 (d, *J* = 7.9 Hz, 2H), 7.67 (s, 1H), 7.52 (d, *J* = 7.9 Hz, 2H), 6.99 (s, 3H), 6.71 (s, 4H), 4.66 (d, *J* = 7.9 Hz 1H), 4.44 – 4.42 (m, 2H), 4.33 (d, *J* = 7.7 Hz, 1H), 4.22 (s, 2H), 4.18 – 3.39 (m, 34H), 3.28 (t, *J* = 8.5 Hz, 1H), 2.68 (d, *J* = 10.1 Hz,

1H), 2.05 (s, 3H), 2.03 (s, 3H), 1.93 – 1.91 (m, 2H), 1.75 (t, *J* = 12.1 Hz, 1H); ESI-MS: m/z calc. for $C_{69}H_{88}N_6O_{34}S$: 1577.5; found:1578.8 [M + H]$^+$.

Compound D8:

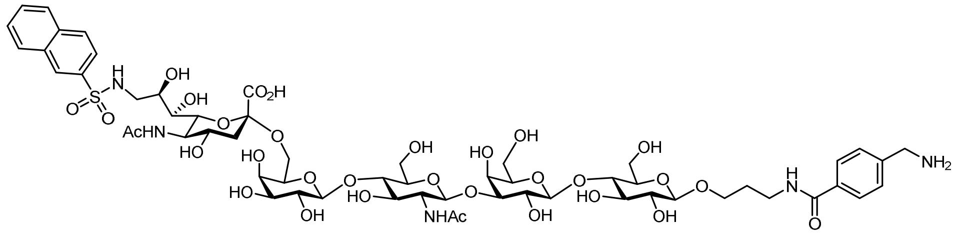

Prepared using General Procedure for “one-pot two-enzyme” approach.

^{1}H NMR (700 MHz, D_2O): δ 8.50 (d, *J* = 1.8 Hz, 1H), 8.13 (d, *J* = 8.8, 1H), 8.1 (d, *J* = 8.2 Hz, 1H), 8.04 (d, *J* = 8.2 Hz, 1H), 7.86 (dd, *J* = 8.6, 1.9 Hz, 1H), 7.80 (d, *J* = 8.1, 2H), 7.76 – 7.74 (m, 1H), 7.72 – 7.70 (m, 1H), 7.55 (d, *J* = 8.1 Hz, 2H), 4.68 (d, *J* = 8.4 Hz, 1H), 4.49 (d, *J* = 7.9 Hz, 1H), 4.42 (d, *J* = 7.3 Hz, 1H), 4.40 (d, *J* = 7.2 Hz, 1H), 4.25 (s, 2H), 4.14 (d, *J* = 3.3 Hz, 1H), 4.03 – 4.01 (m, 1H), 4.00 – 3.45 (m, 30H), 3.36 – 3.27 (m, 2H), 3.05 (dd, *J* = 13.7, 7.4 Hz, 1H), 2.62 (dd, *J* = 12.6, 4.6 Hz, 1H), 2.04 – 1.93 (m, 8H), 1.72 (t, *J* = 12.2 Hz, 1H); ^{13}C NMR (176 MHz, D_2O): δ 174.9, 135.2, 134.5, 131.8, 130.0, 129.3, 129.0, 128.4, 128.0, 127.9, 127.8, 121.6, 103.4, 102.9, 102.5, 102.1, 99.5, 82.0, 80.4, 78.5, 74.9, 74.8, 74.4, 72.8, 72.6, 72.1, 69.9, 68.0, 67.7, 63.3, 61.0, 60.1, 54.9, 51.8, 45.2, 42.6, 39.6, 37.0, 28.4, 22.2, 22.0; ESI-MS: m/z calc. for $C_{58}H_{83}N_5O_{31}S$: 1377.5; found: 1378.4 [M + H]$^+$.

Compound D10:

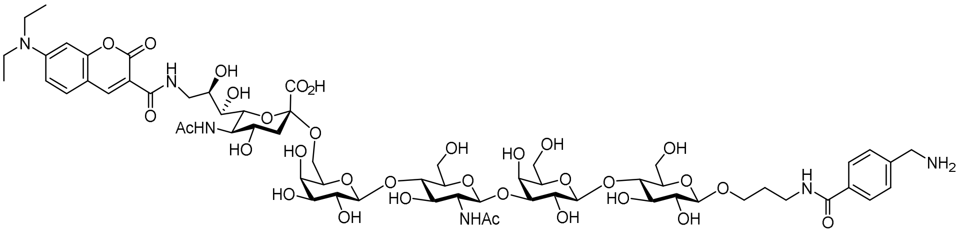

Prepared using General Procedure for “one-pot two-enzyme” approach.

^{1}H NMR (700 MHz, D_2O): δ 8.40 (s, 1H), 7.79 (dd, *J* = 8.2, 2.7 Hz, 2H), 7.55 (dd, *J* = 8.4, 2.6 Hz, 2H), 7.45 (dd, *J* = 9.1, 2.6 Hz, 1H), 6.81 (d, *J* = 9.2 Hz, 1H), 6.54 (s, 1H), 4.73 (d, *J* = 8.0 Hz, 1H), 4.49 (d, *J* = 8.0 Hz, 1H), 4.44 – 4.43 (m, 2H), 4.25 (s, 2H), 4.15 (s, 1H), 4.08 – 3.42 (m, 37H), 3.35 – 3.31 (m, 1H), 2.70 – 2.63 (m, 1H), 2.11 – 2.00 (m, 6H), 1.98 – 1.95 (m, 2H), 1.78 (t, *J* = 7.2 Hz ,1H), 1.20 – 1.18 (m, 6H); ^{13}C NMR (176 MHz, D_2O): δ 170.2, 136.3, 134.4, 129.0, 127.8, 103.5, 103.0, 102.5, 102.1, 99.7, 82.0, 80.6, 78.4, 74.9, 74.8, 74.4, 74.3, 72.8, 69.3, 68.3, 67.9, 61.0, 60.1 , 42.6, 37.0, 28.4, 22.3, 22.1, 11.5; ESI-MS: m/z calc. for $C_{62}H_{90}N_6O_{32}$: 1430.6; found: 1431.7 [M + H]$^+$.

Compound D11:

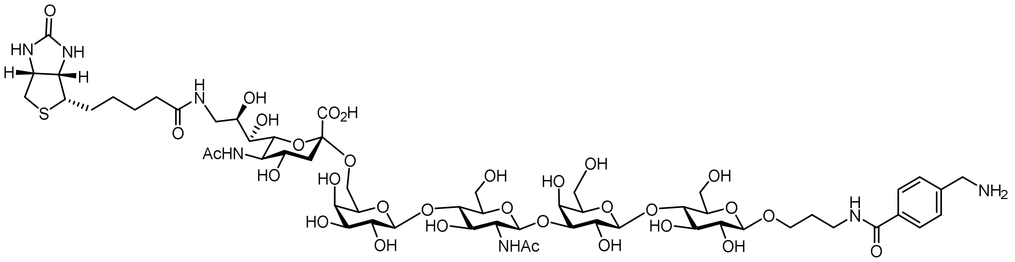

Prepared using General Procedure for “one-pot two-enzyme” approach.

^{1}H NMR (700 MHz, D_2O): δ 7.81 (d, J = 8.2 Hz, 2H), 7.56 (d, J = 8.0 Hz, 2H), 4.73 (d, J = 7.9 Hz, 1H), 4.62 (dd, J = 8.0, 4.9 Hz, 1H), 4.50 (d, J = 7.9 Hz, 1H), 4.46 (d, J = 7.9 Hz, 1H), 4.45 – 4.42 (m, 2H), 4.26 (s, 2H), 4.16 (d, J = 3.4 Hz, 1H), 4.03 – 4.00 (m, 2H), 3.98 – 3.90 (m, 4H), 3.87 – 3.51 (m, 25H), 3.43 (dd, J = 8.9, 1.4 Hz, 1H), 3.38 – 3.29 (m, 3H), 3.01 (dd, J = 13.0, 5.0 Hz, 1H), 2.79 (d, J = 13.1 Hz, 1H), 2.31 (t, J = 7.4 Hz, 2H), 2.06 (s, 3H), 2.04 (s, 3H), 1.98 – 1.96 (m, 2H), 1.78 – 1.72 (m, 1H), 1.70 – 1.57 (m, 3H), 1.45 – 1.42 (m, 2H); ^{13}C NMR (176 MHz, D_2O): δ 177.2, 174.8, 170.3, 165.4, 134.5, 129.0, 127.8, 103.5, 102.9, 102.6, 102.1, 99.4, 78.4, 74.9, 74.8, 74.4, 74.2, 73.6, 72.8, 72.5, 72.4, 72.2, 70.7, 69.9, 69.8, 69.6, 68.3, 68.0, 67.7, 62.1, 61.0, 60.3, 55.3, 54.9, 51.8, 42.6, 39.7, 37.0, 35.4, 28.4, 27.8, 27.6, 25.2, 22.3, 22.1. ESI-MS: m/z calc. for $C_{58}H_{91}N_7O_{31}S$: 1414.4; found: 1415.5 $[M + H]^+$.

Compound D12

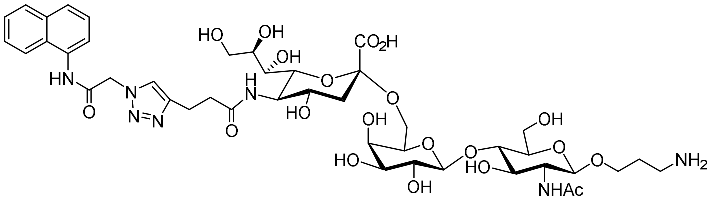

Prepared using General Procedure for “one-pot two-enzyme” approach.

^{1}H NMR (400 MHz, Deuterium Oxide): δ 8.03 – 7.98 (m, 1H), 7.98 – 7.91 (m, 3H), 7.68 – 7.58 (m, 2H), 7.60 – 7.51 (m, 2H), 5.59 (s, 2H), 4.48 (d, J = 8.4 Hz, 1H), 4.42 (d, J = 7.9 Hz, 1H), 4.03 – 3.48 (m, 20H), 3.37 (d, J = 8.9 Hz, 1H), 3.10 – 3.00 (m, 4H), 2.69 (t, J = 7.1 Hz, 2H), 2.63 (dd, J = 12.4, 4.5 Hz, 1H), 2.01 (s, 3H), 1.95 – 1.84 (m, 2H), 1.67 (t, J = 12.2 Hz, 1H); ESI-MS: m/z Calc’d C43H61N7O20 for 995.40, found: 994.60 $[M – H]^-$.

5. NMR and MS Spectra

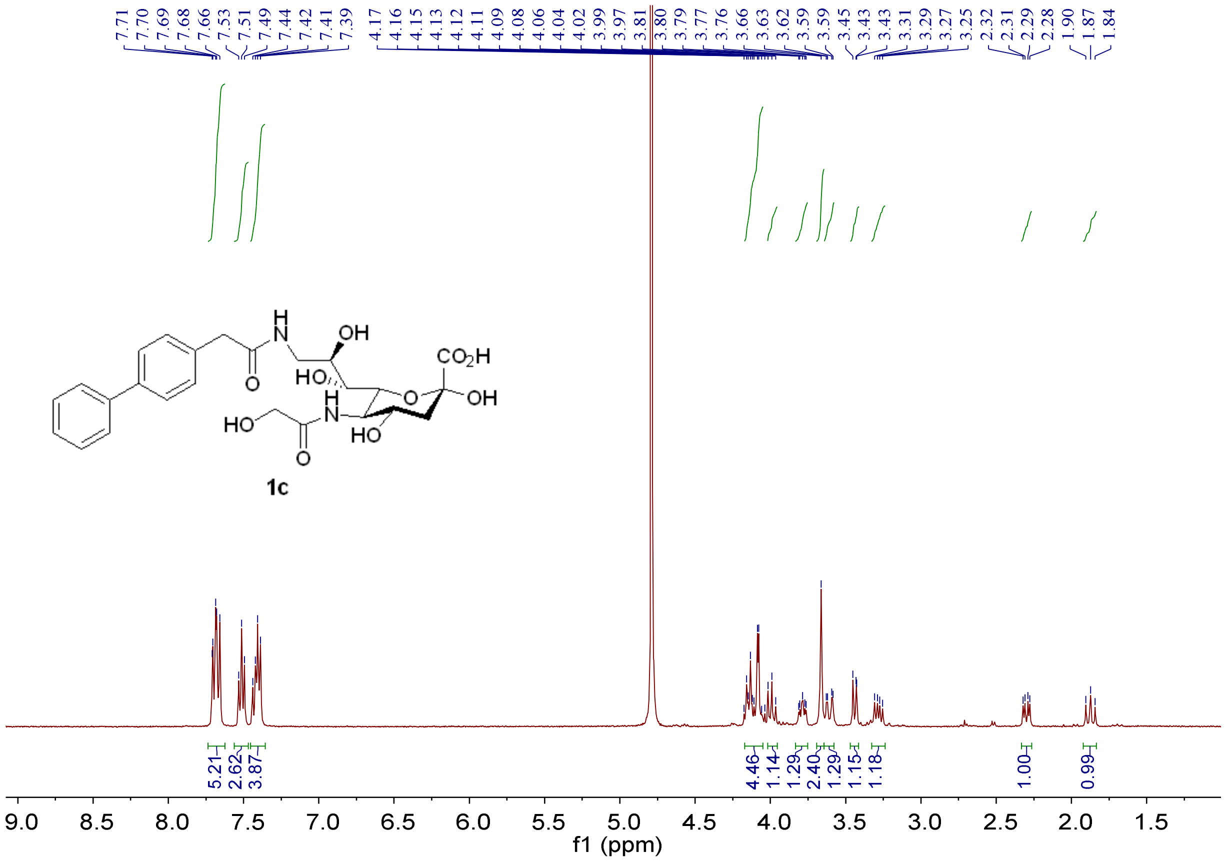

^{1}H NMR spectra of compound **1c**

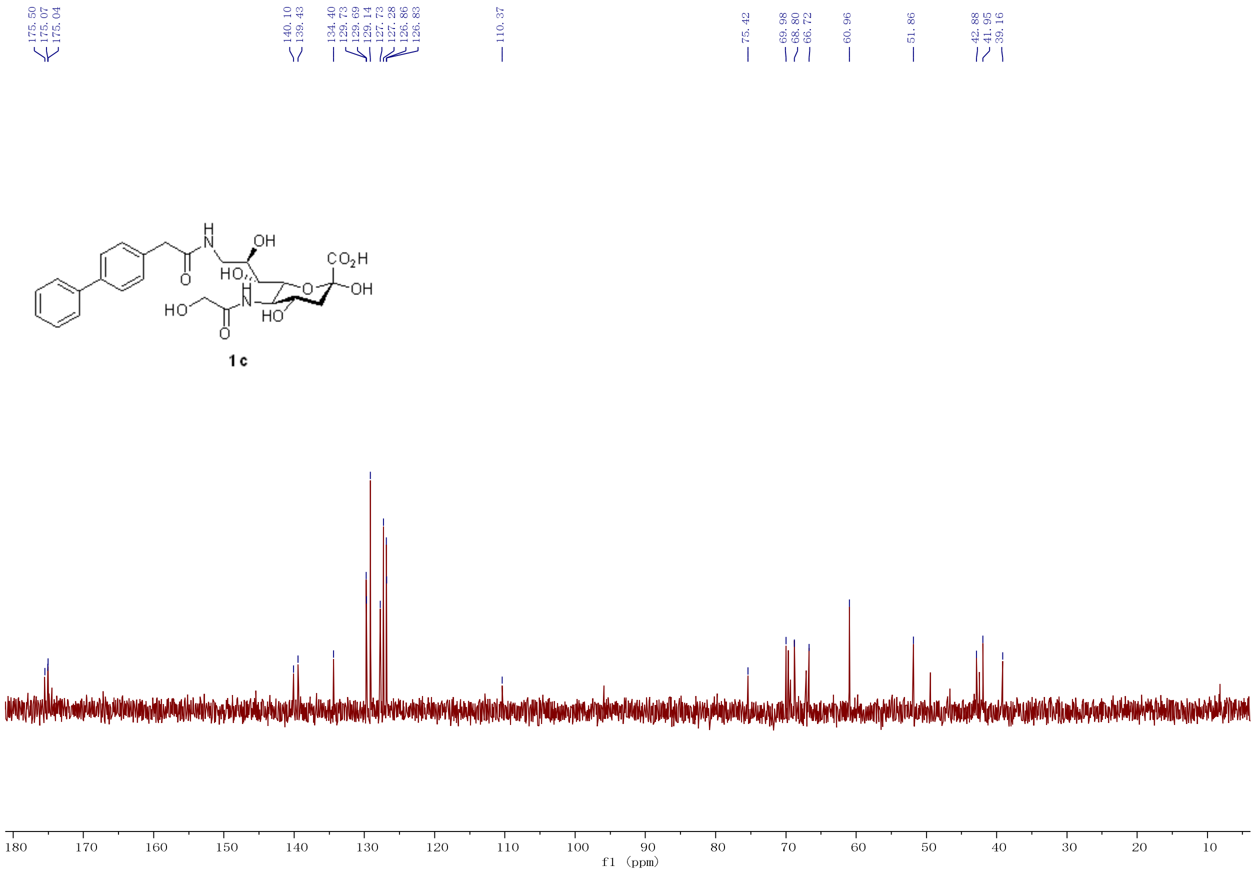

^{13}C NMR spectra of compound **1c**

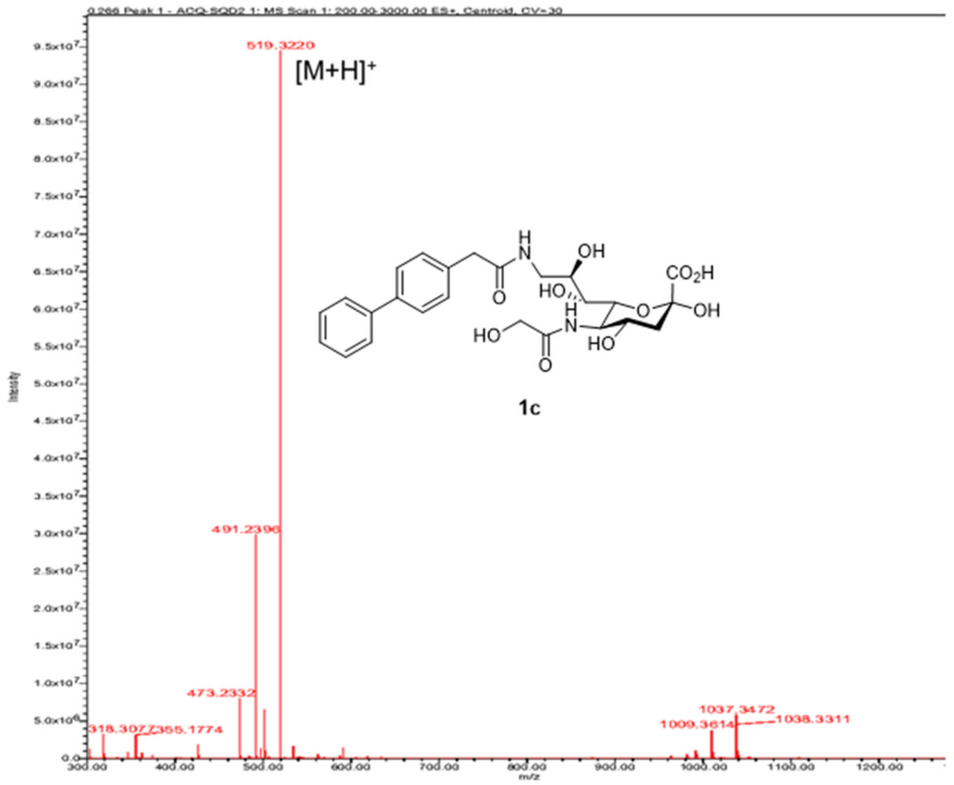

ESI-MS specta of compound **1c**

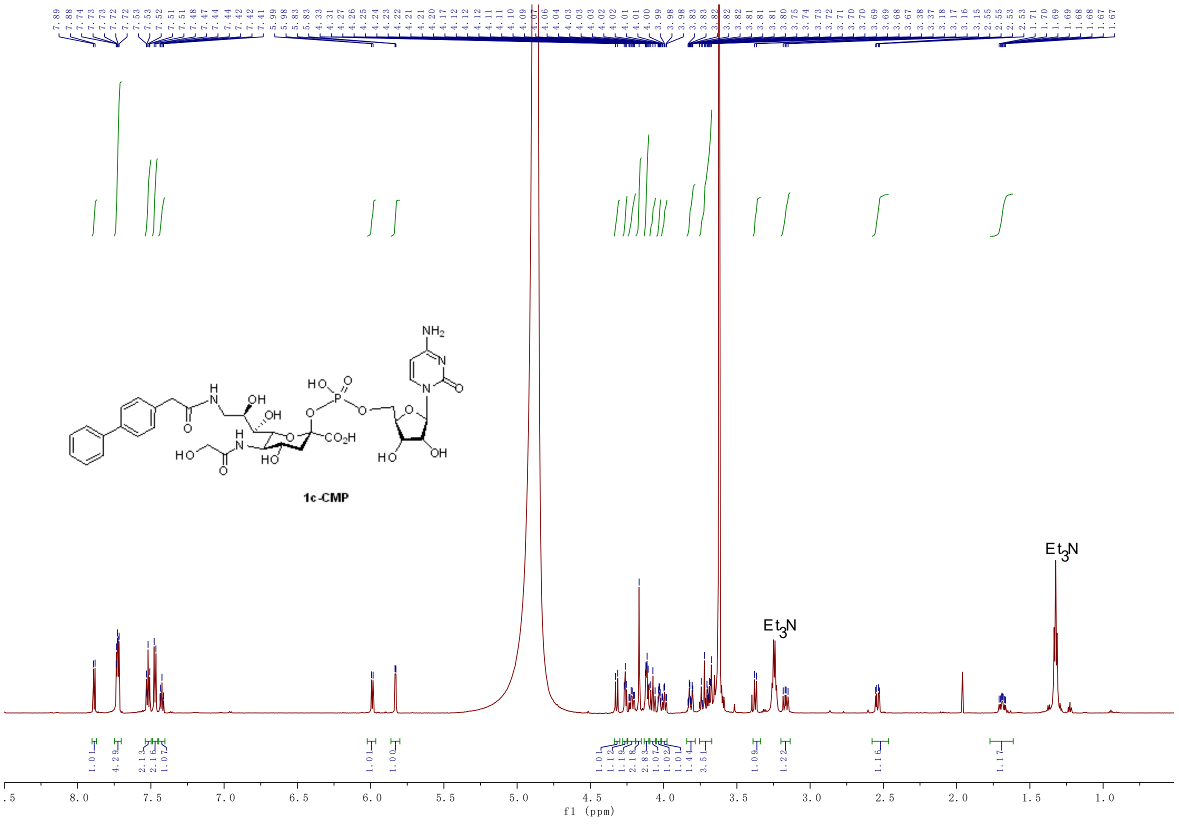

^{1}H NMR spectra of compound **1c-CMP**

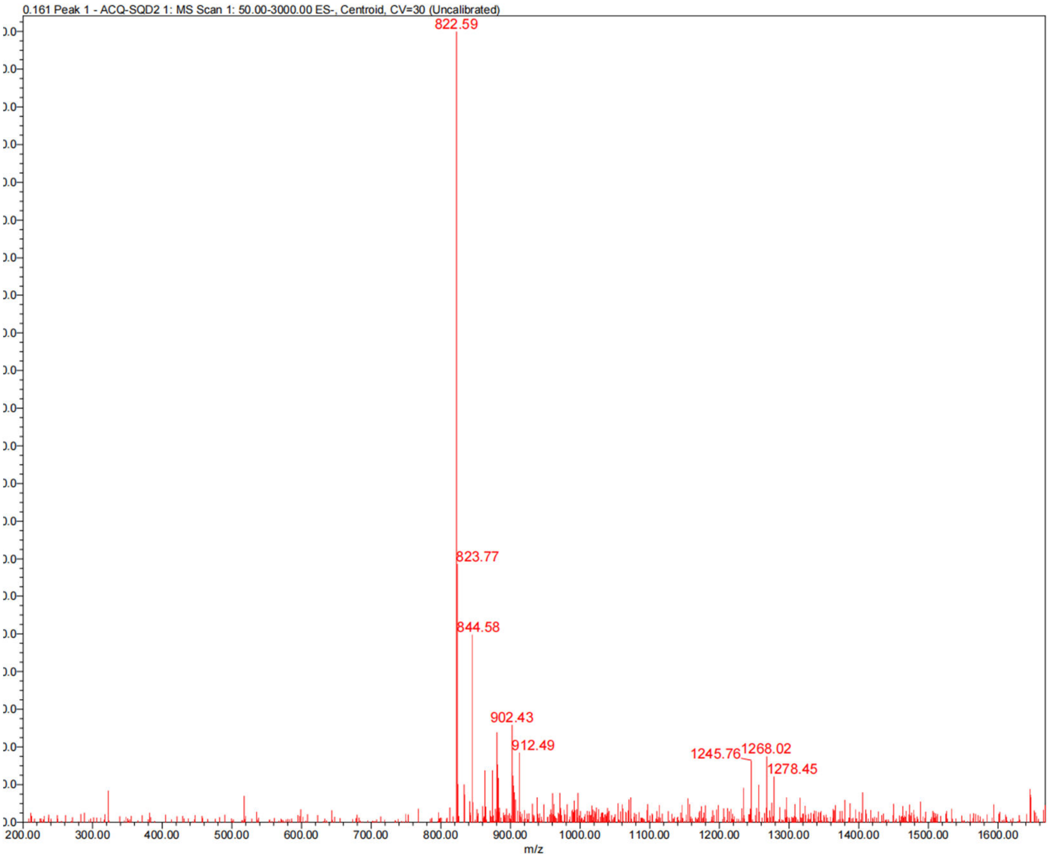

ESI-MS specta of compound **1c-CMP**

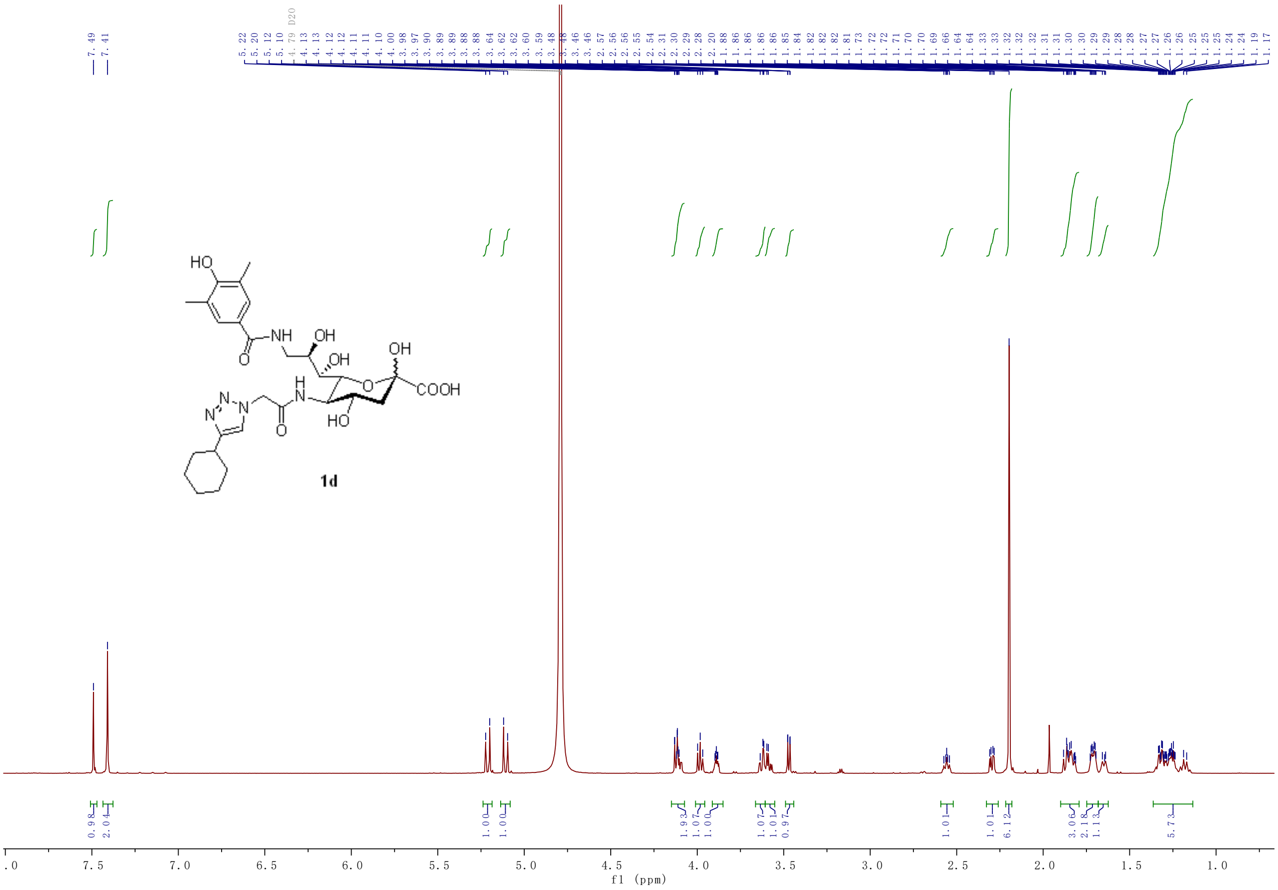

^{1}H NMR spectra of compound **1d**

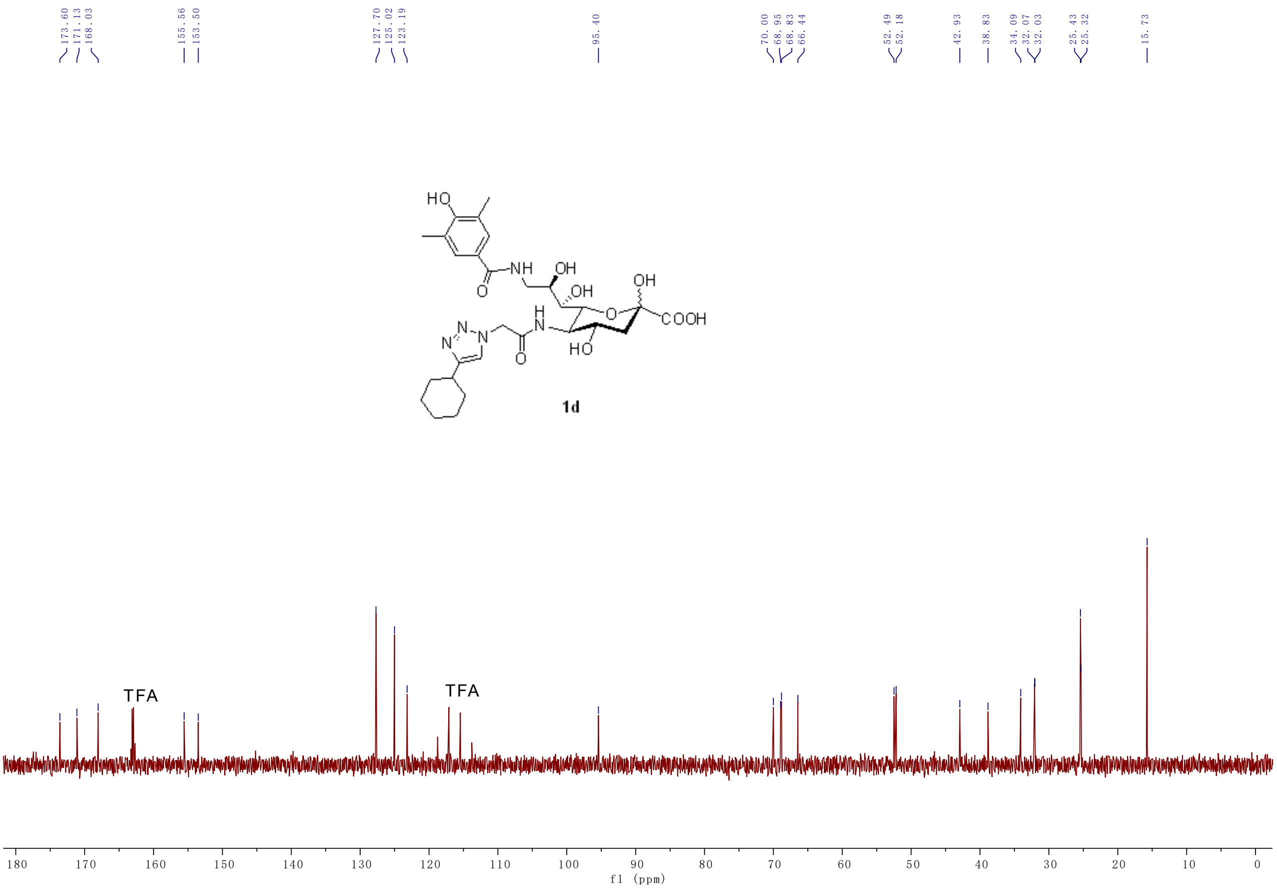

^{13}C NMR spectra of compound **1d**

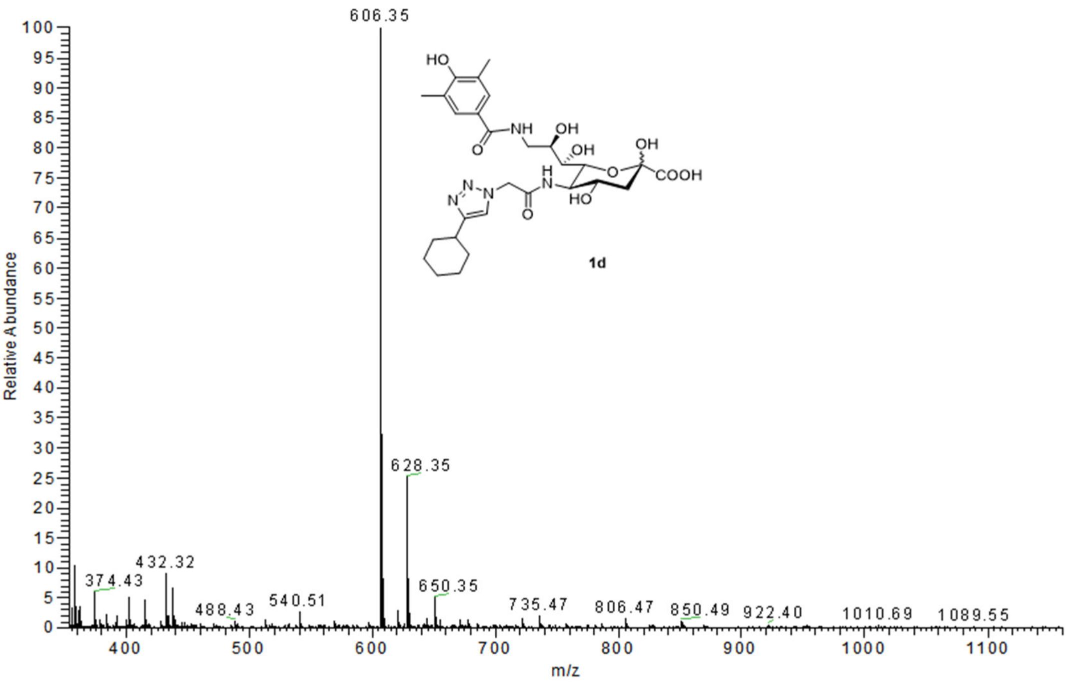

ESI-MS spectra of compound **1d**

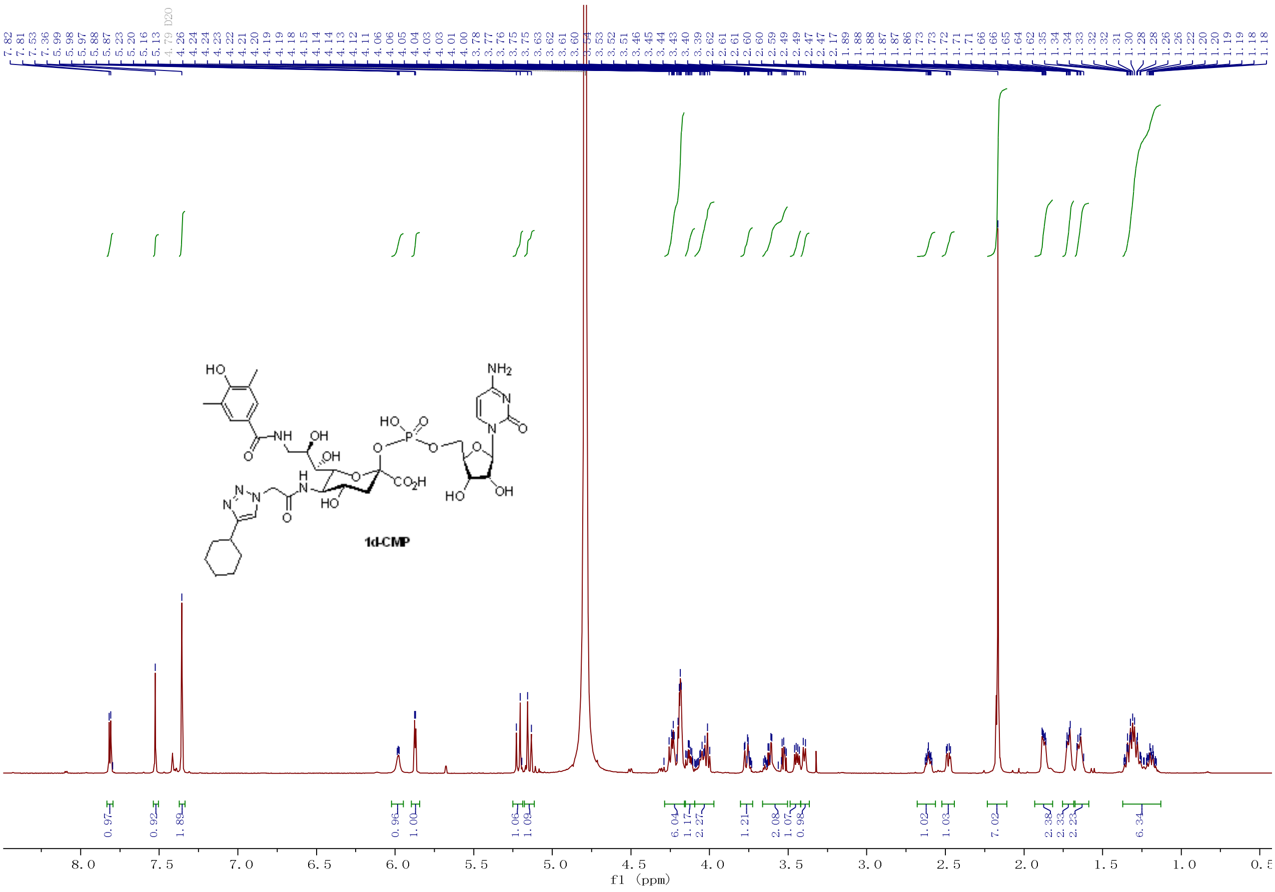

^{1}H NMR spectra of compound **1d-CMP**

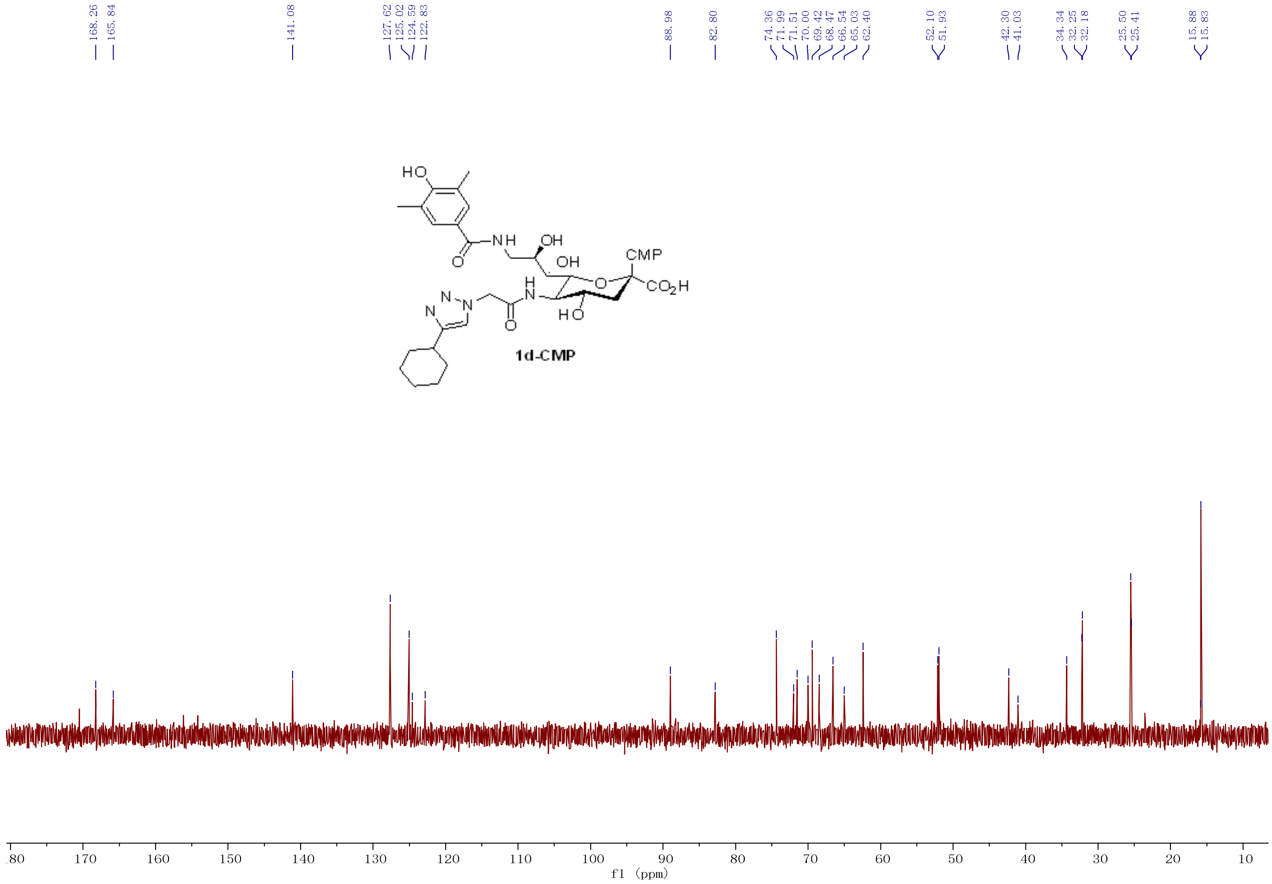

^{13}C NMR spectra of compound **1d-CMP**

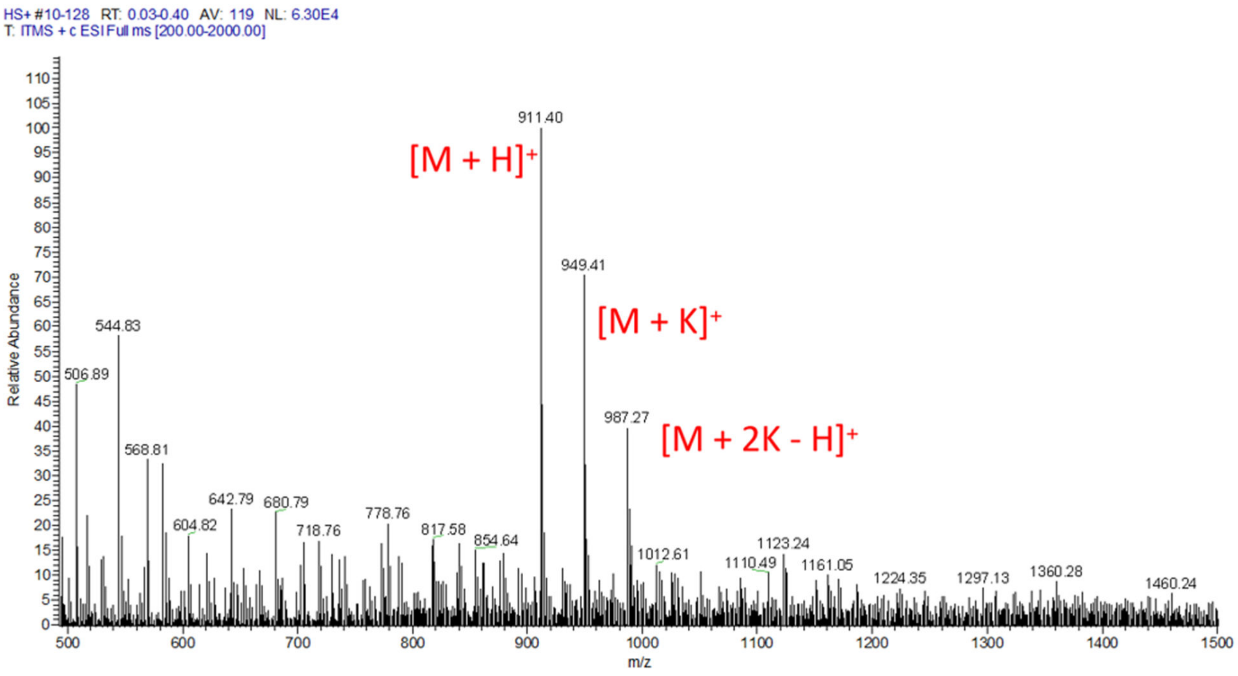

ESI-MS spectra of compound **1d-CMP**

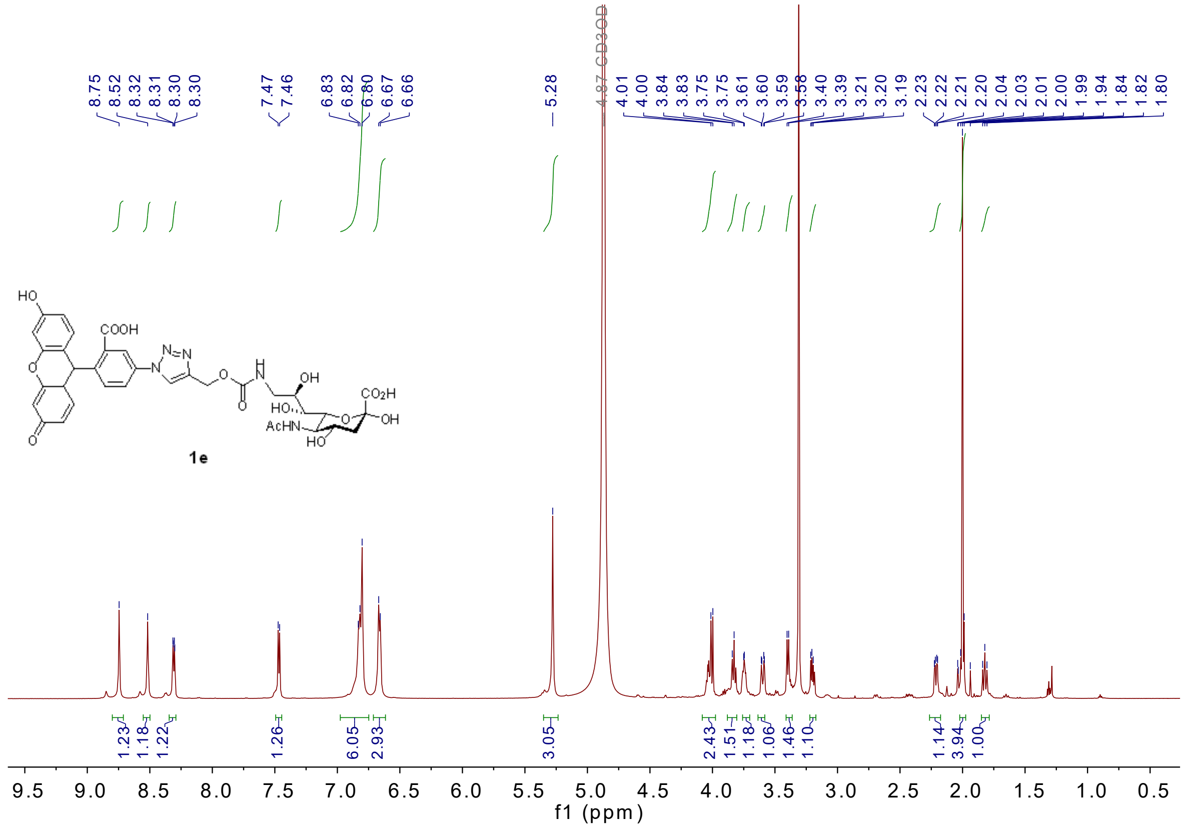

^{1}H NMR spectra of compound **1e**

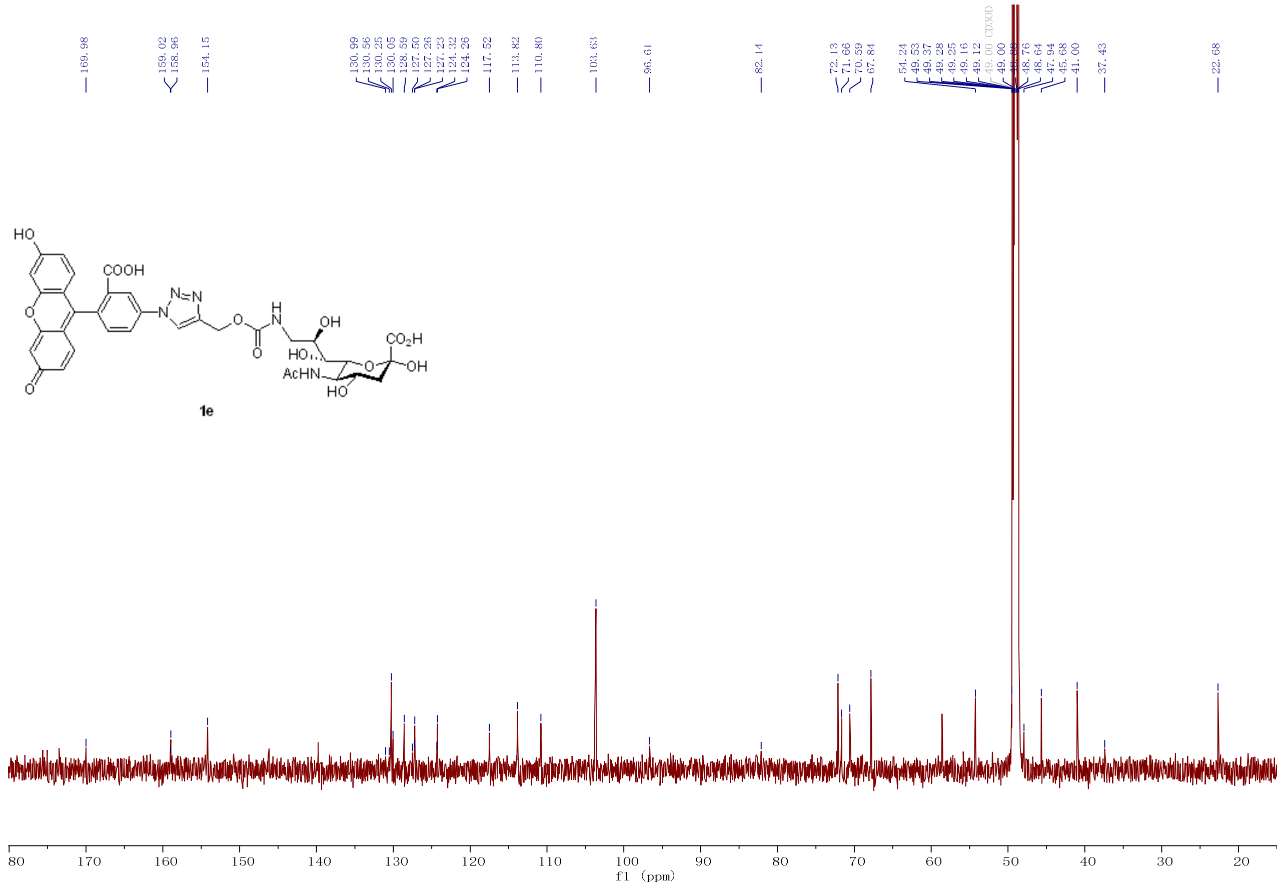

^{13}C NMR spectra of compound **1e**

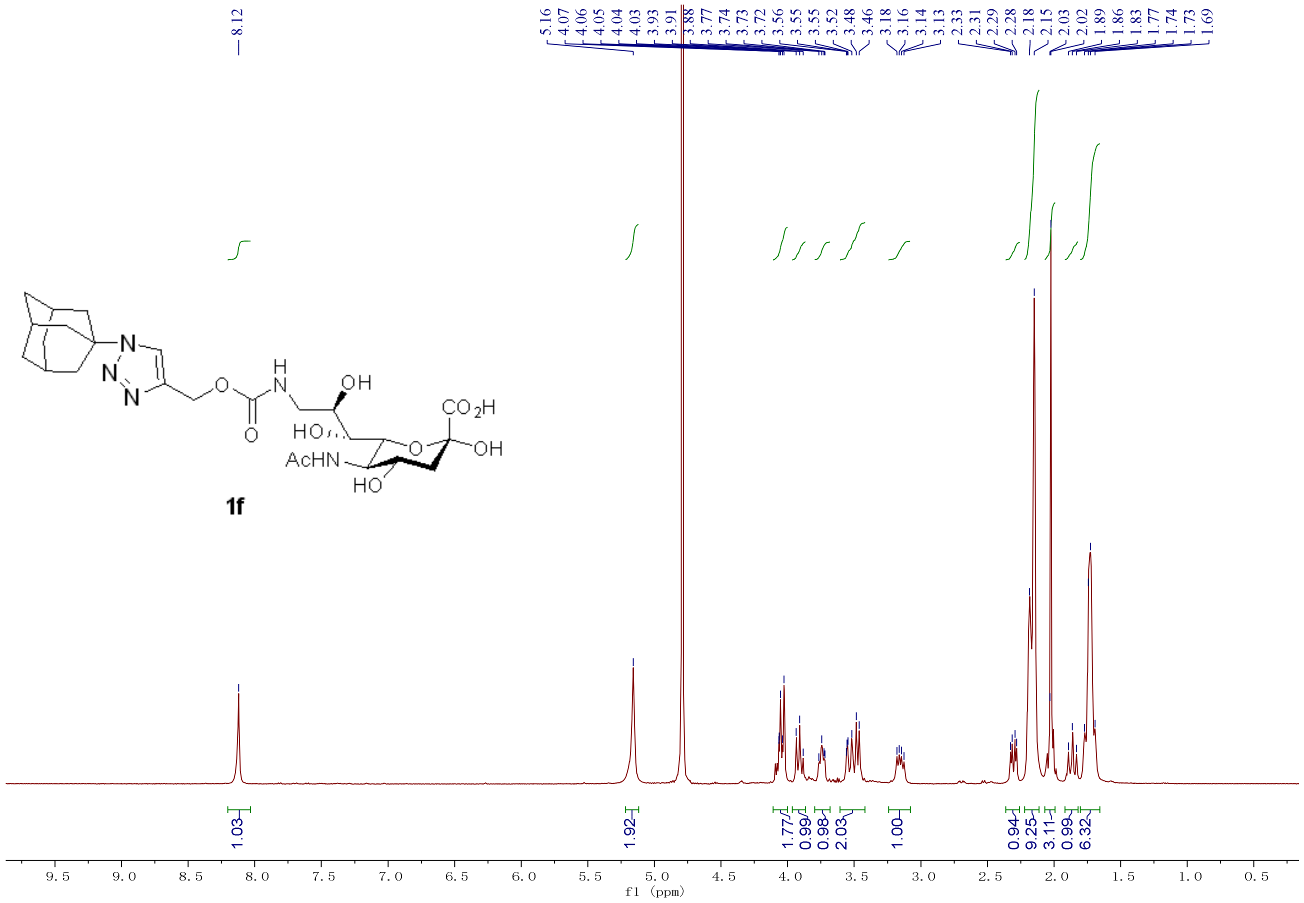

^{1}H NMR spectra of compound **1f**

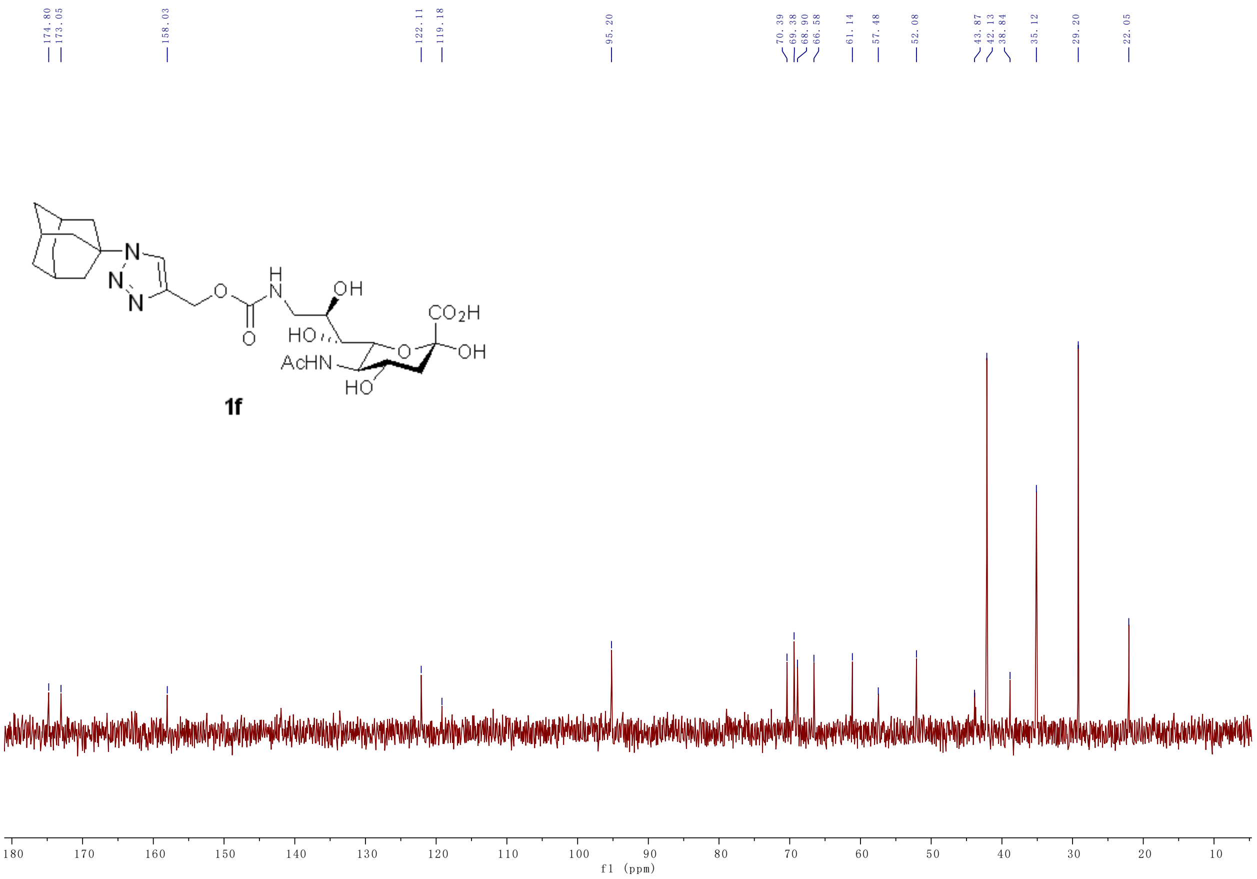

^{13}C NMR spectra of compound **1f**

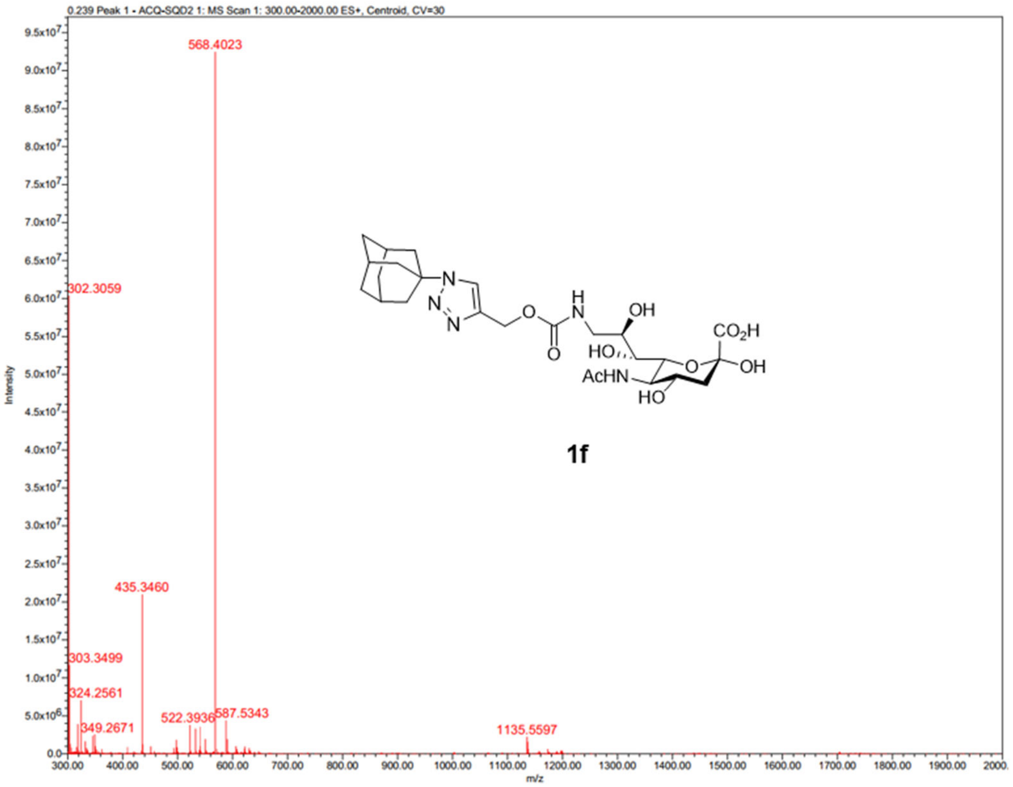

ESI-MS specta of compound **1f**

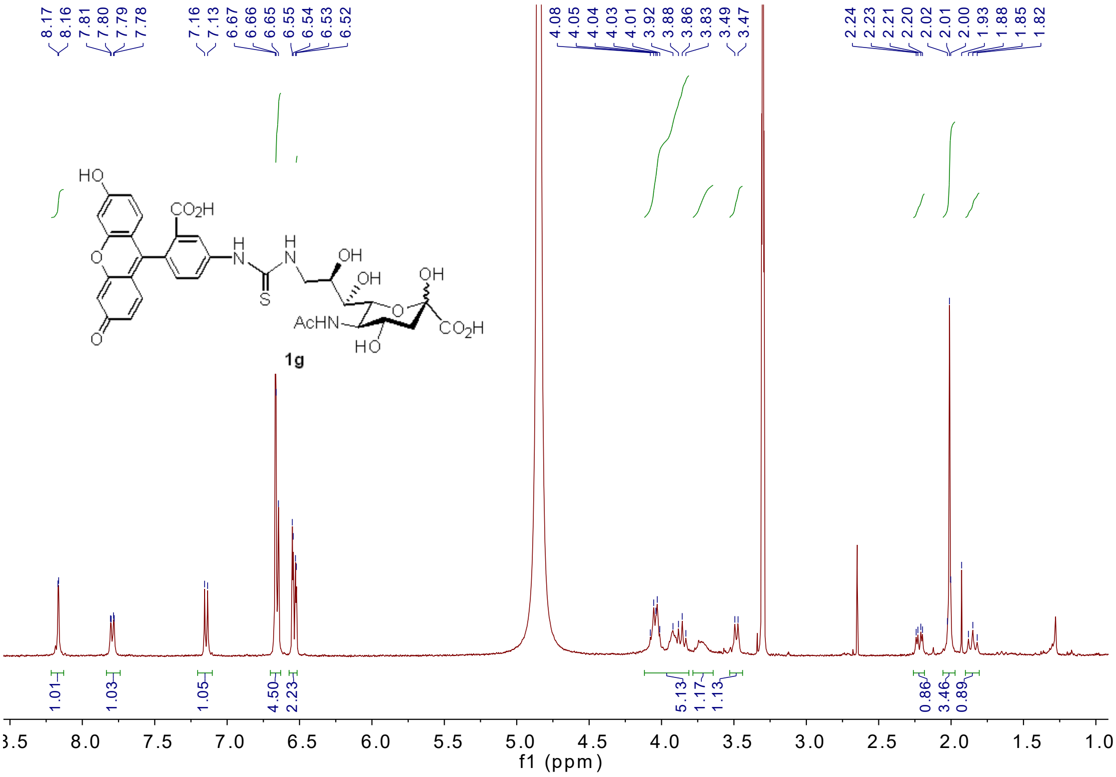

^{1}H NMR spectra of compound **1g**

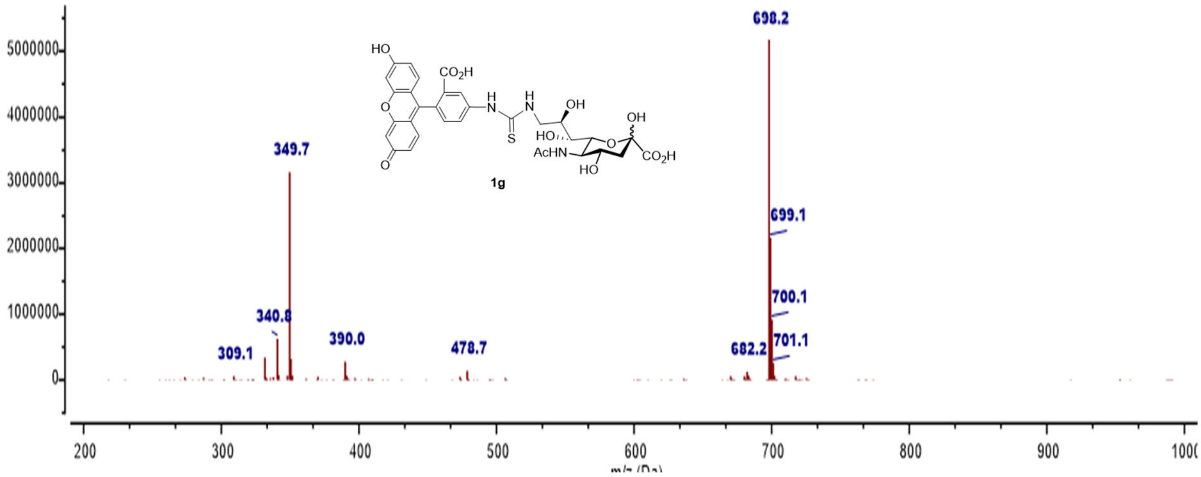

ESI-MS specta of compound **1g**

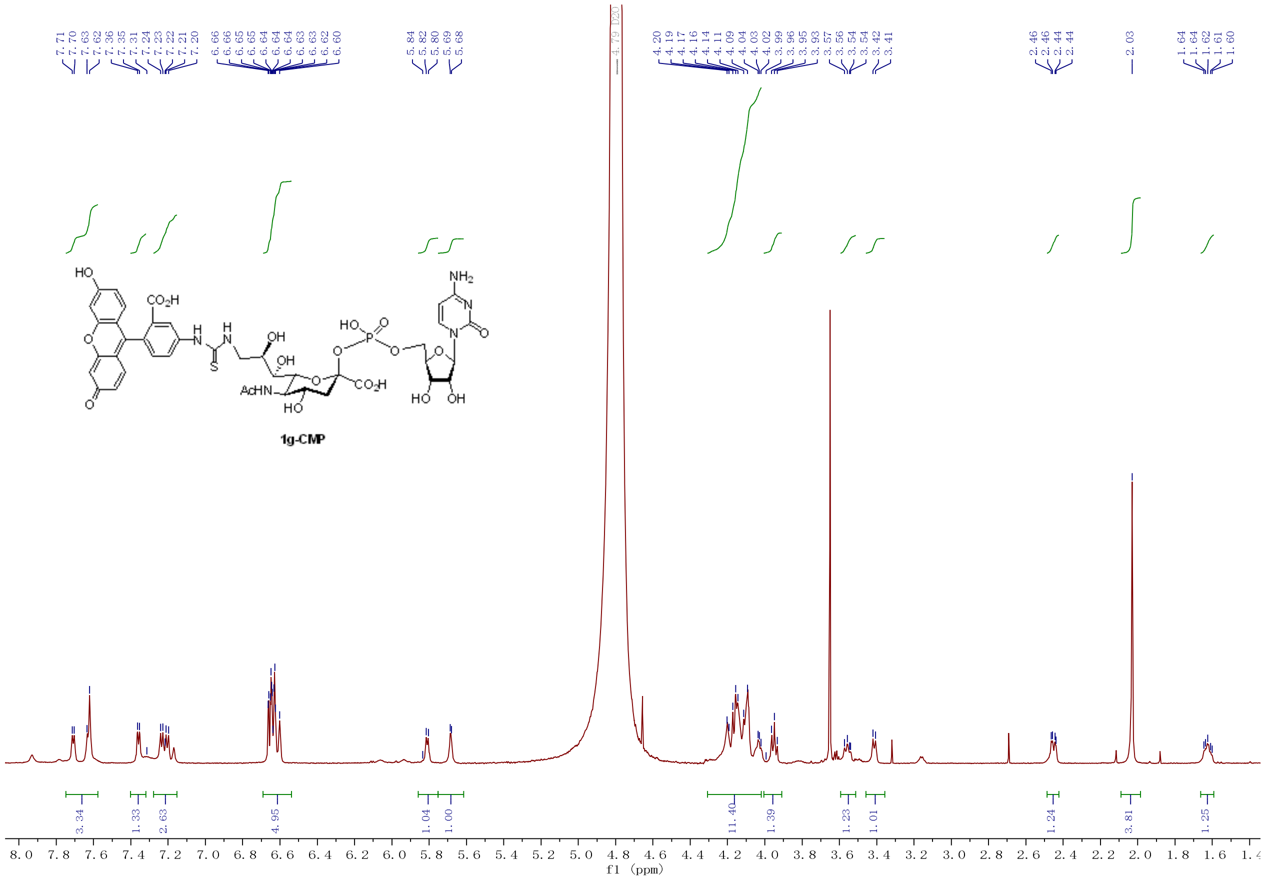

^{1}H NMR spectra of compound **1g-CMP**

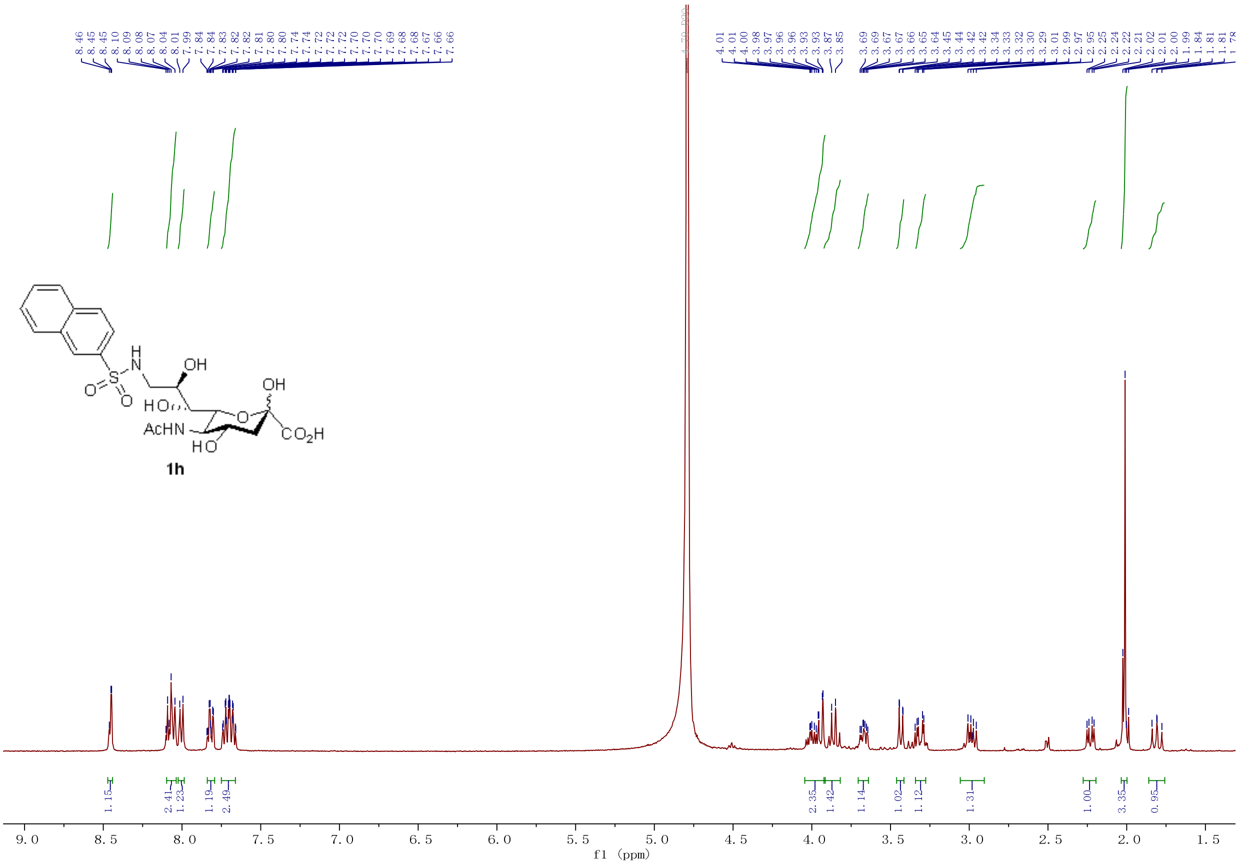

^{1}H NMR spectra of compound **1h**

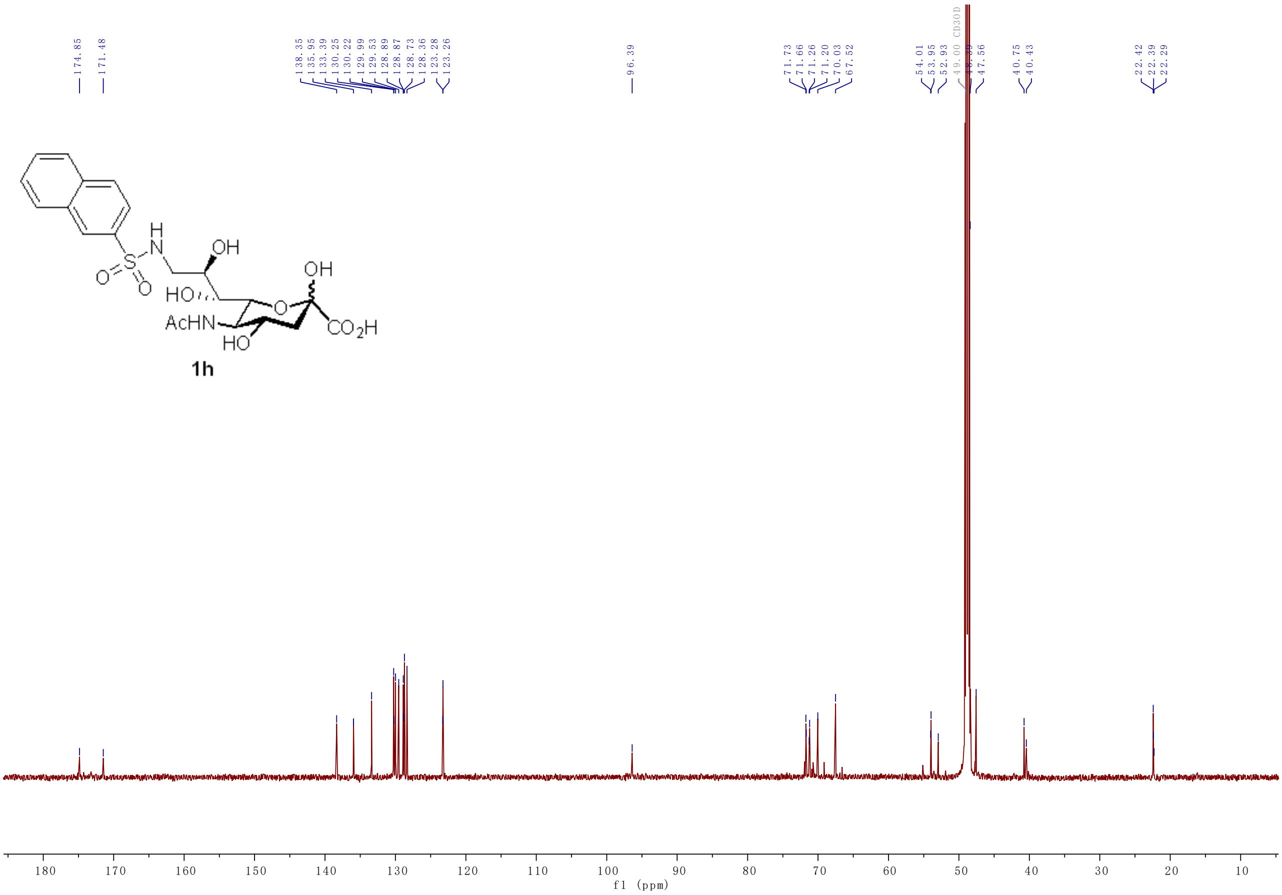

^{13}C NMR spectra of compound **1h**

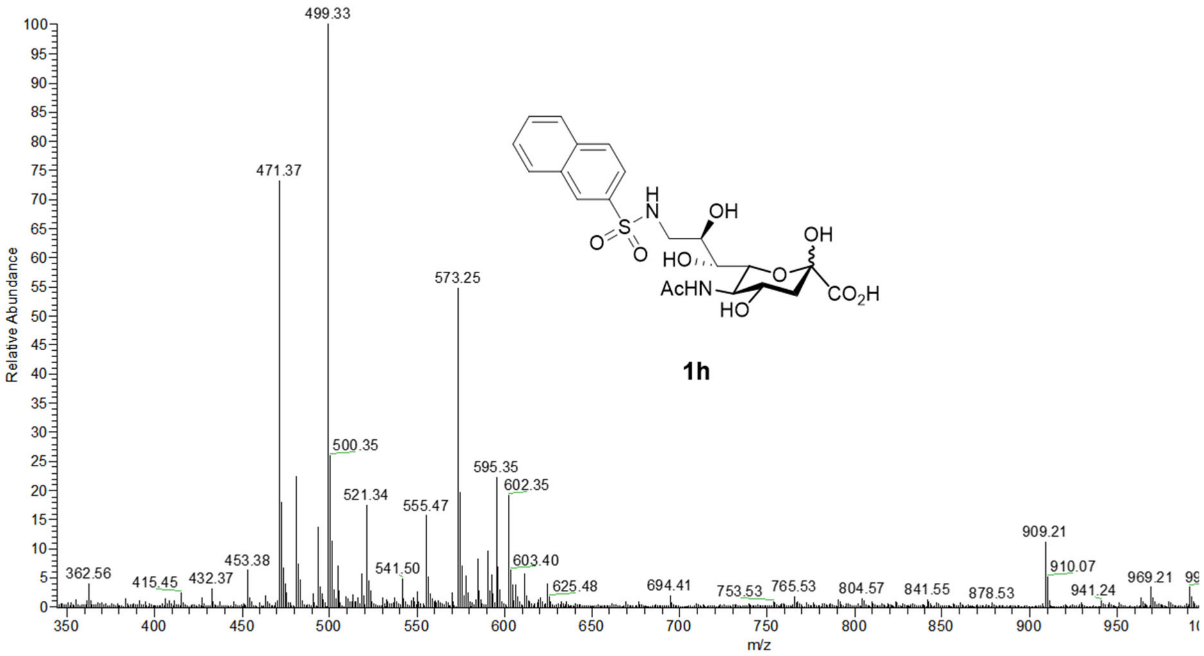

ESI-MS specta of compound **1h**

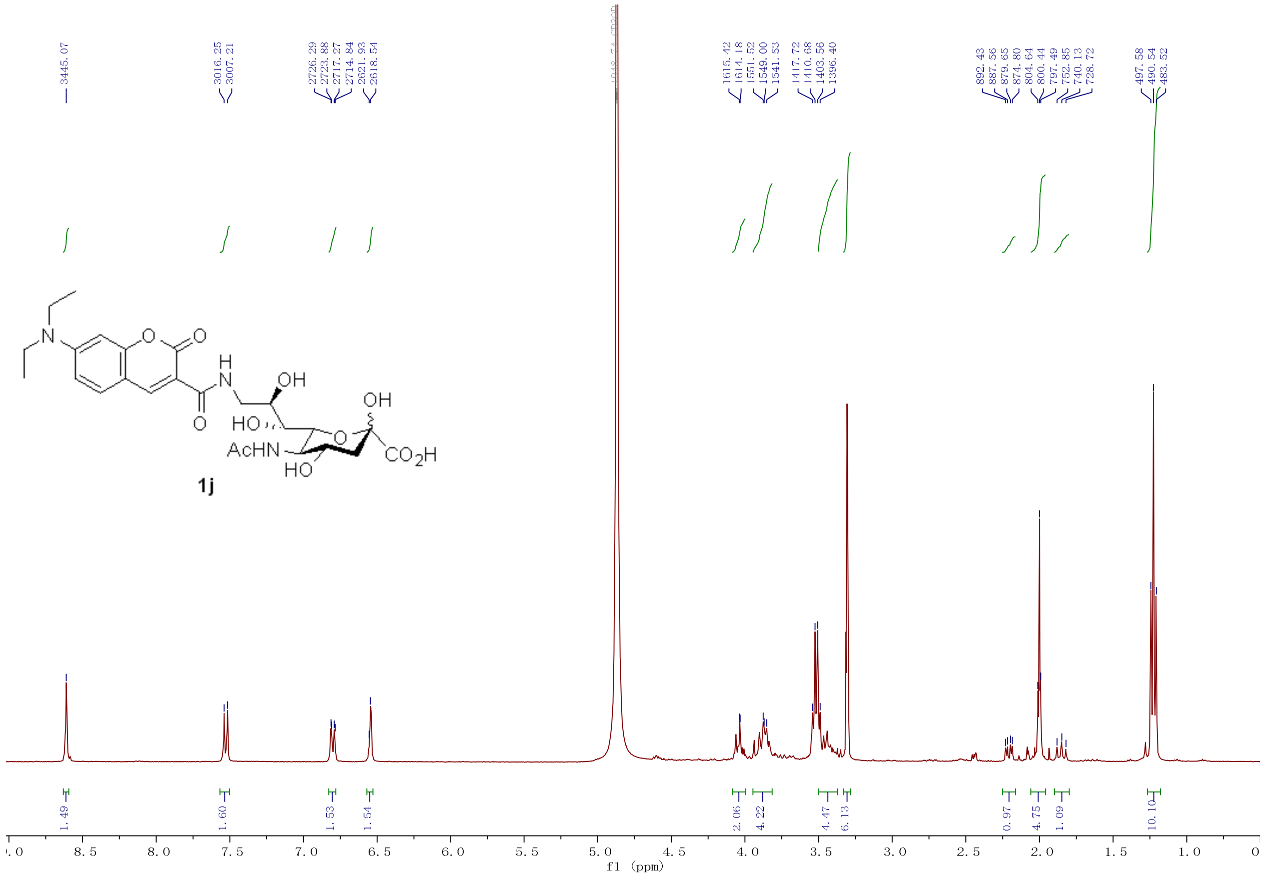

^{1}H NMR spectra of compound **1j**

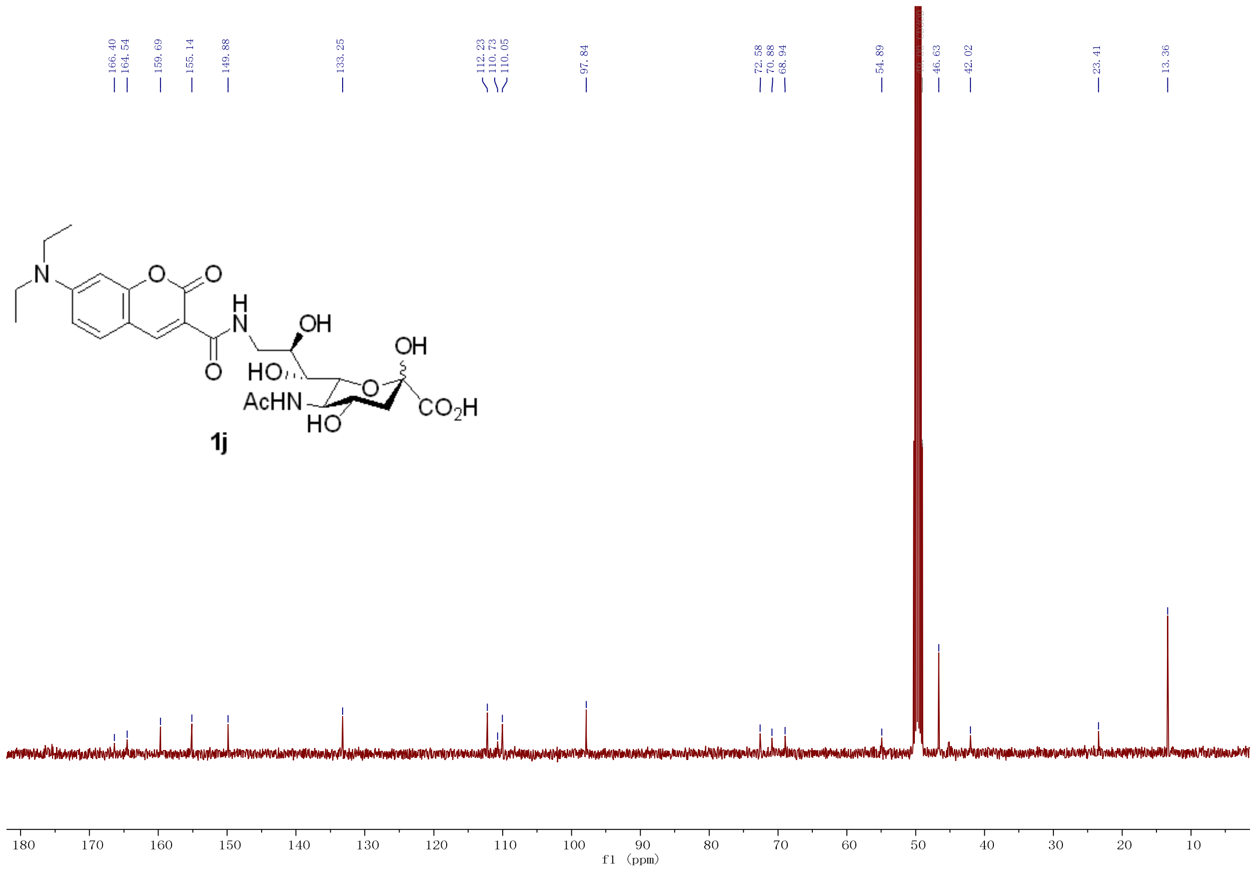

^{13}C NMR spectra of compound **1j**

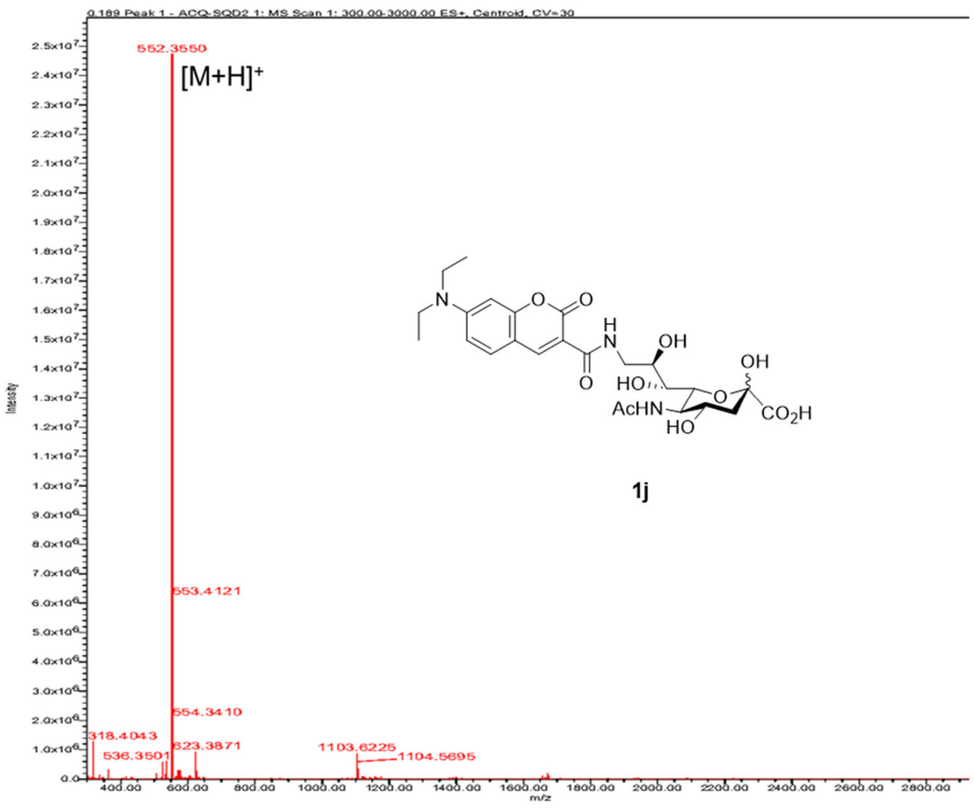

ESI-MS specta of compound **1j**

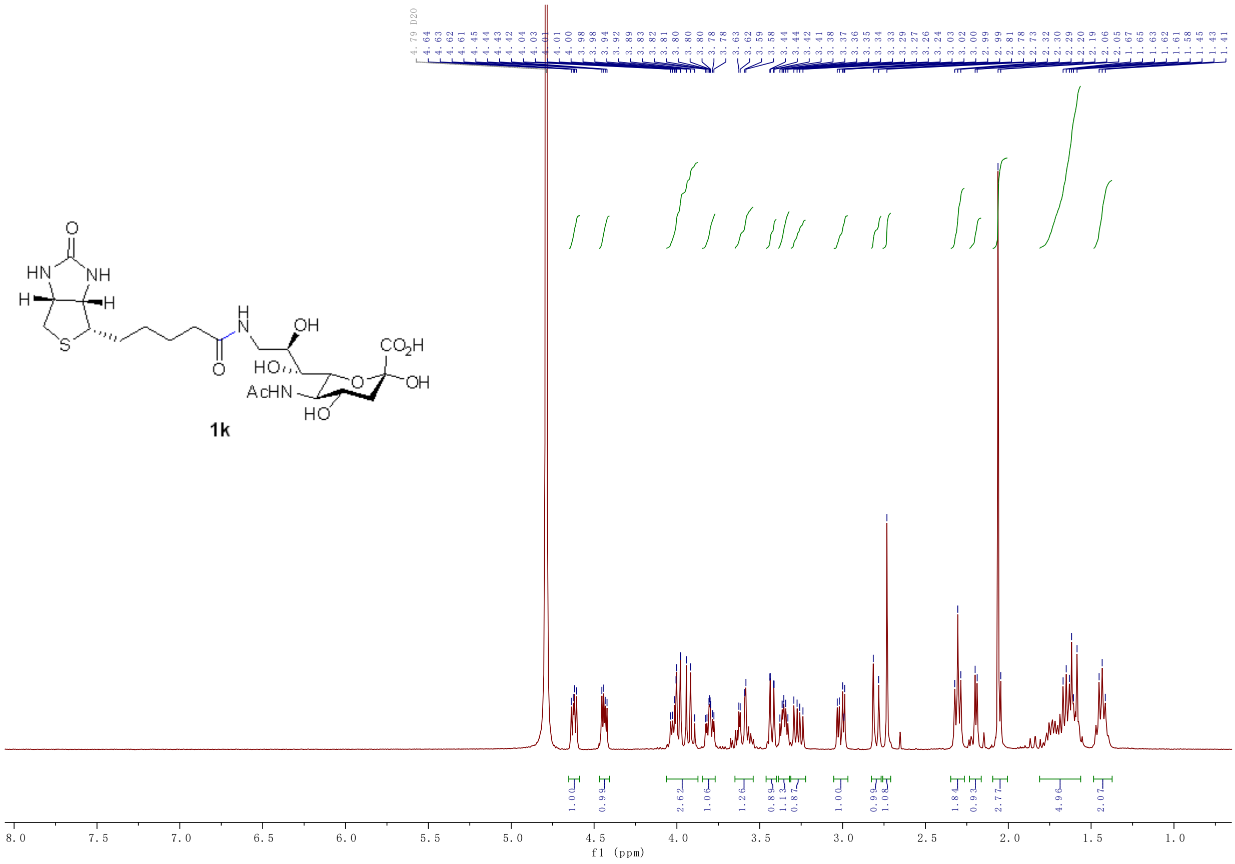

^{1}H NMR spectra of compound **1k**

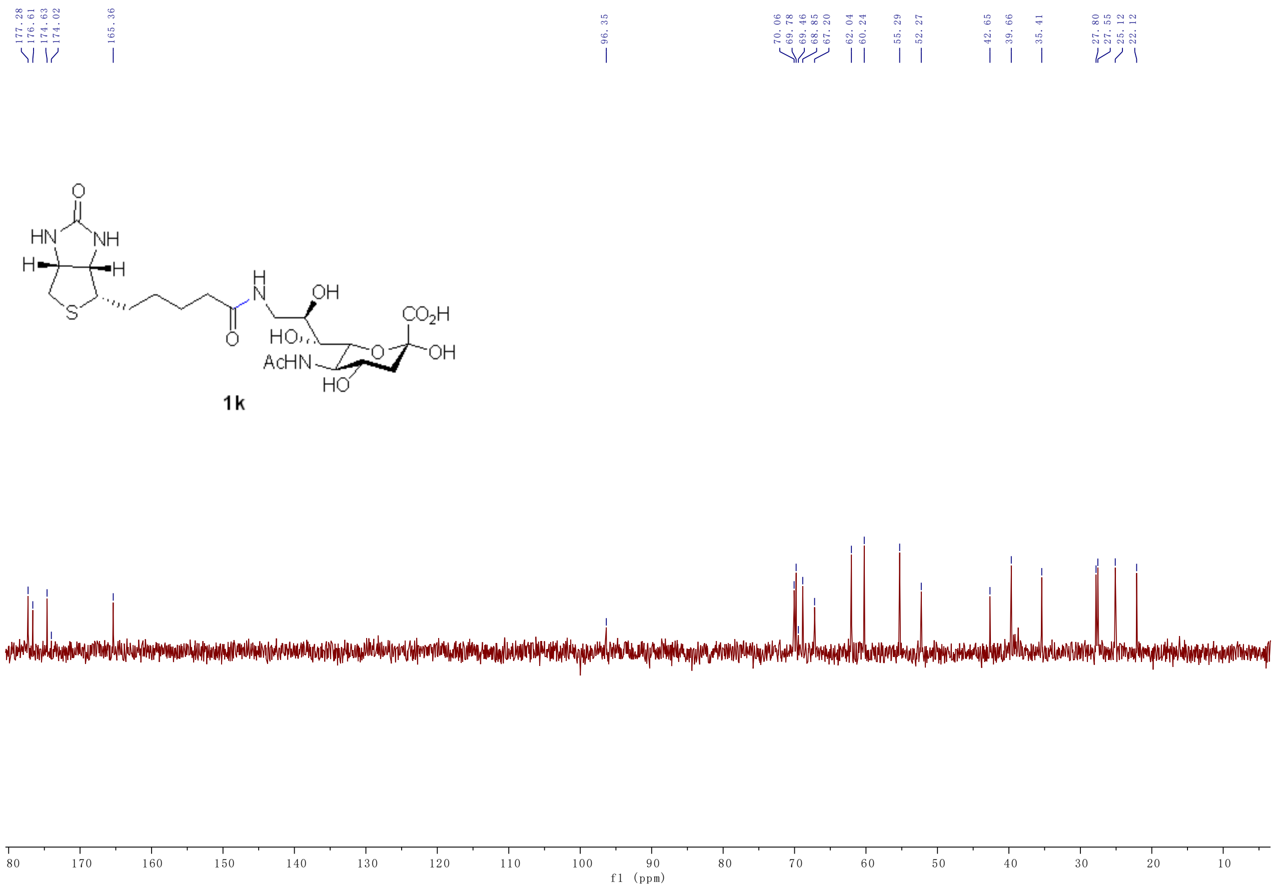

^{13}C NMR spectra of compound **1k**

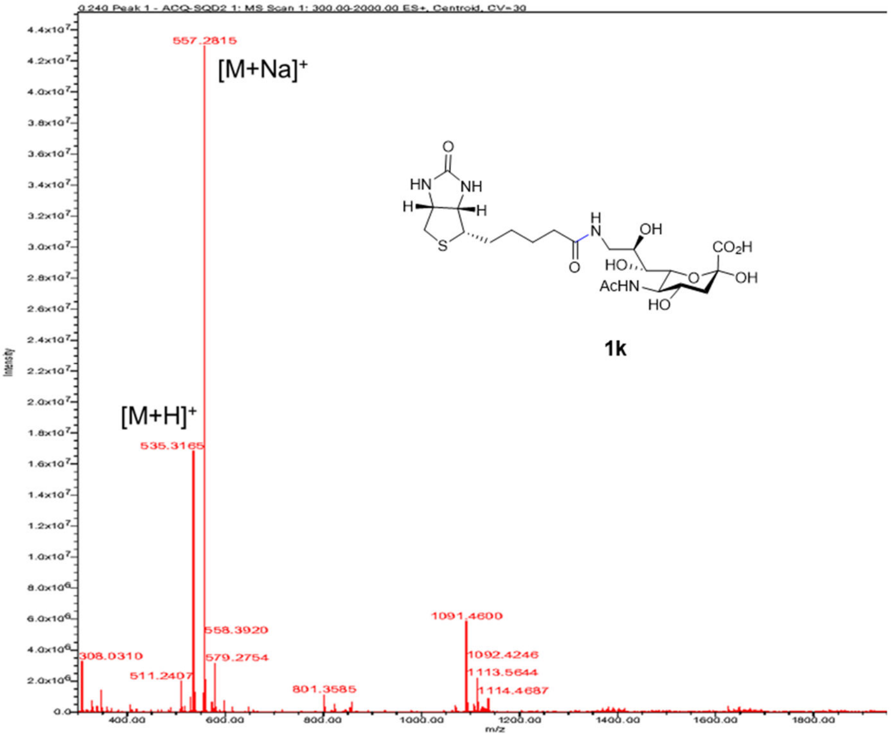

ESI-MS specta of compound **1k**

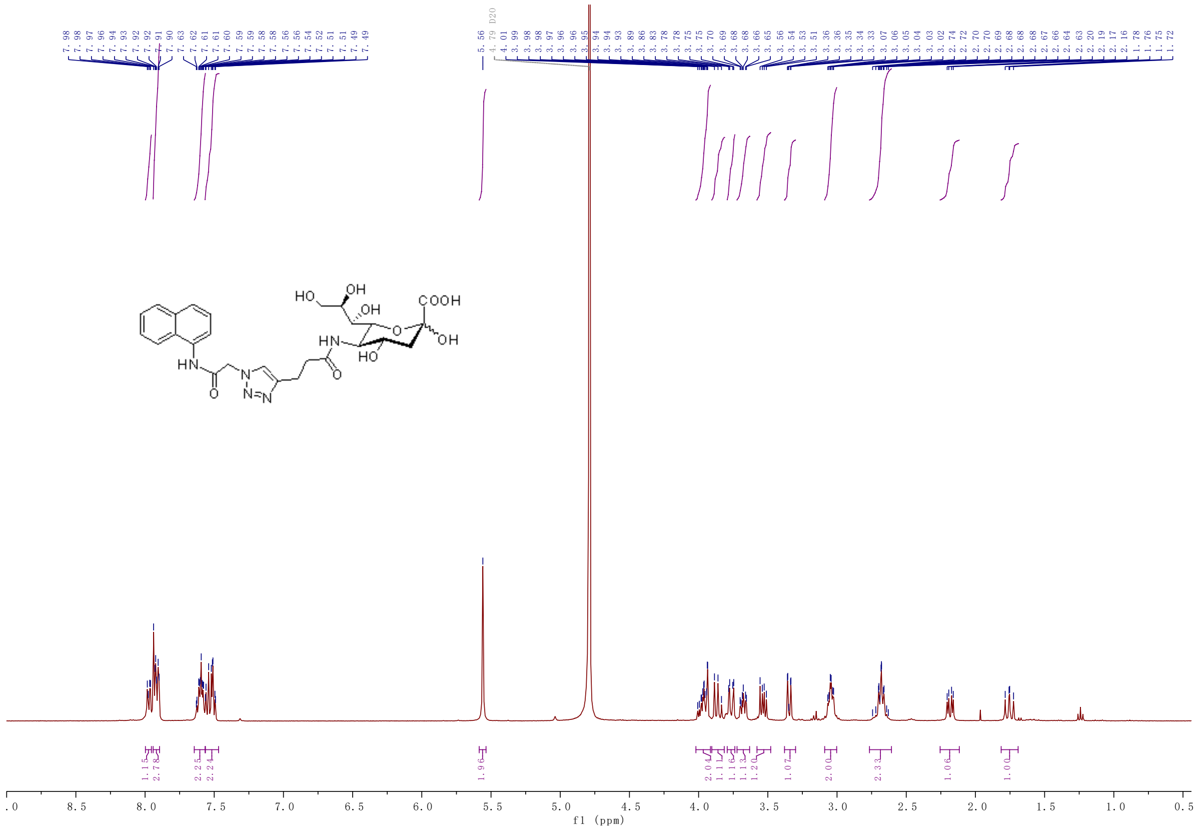

^{1}H NMR spectra of compound **1l**

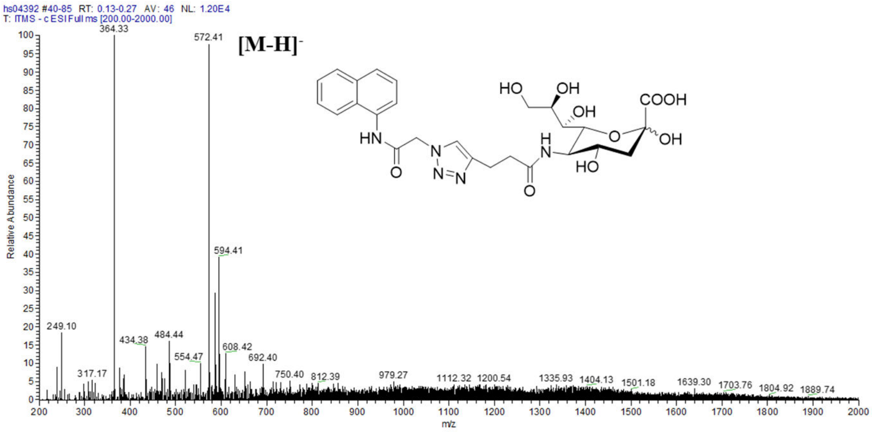

ESI-MS specta of compound **1l**

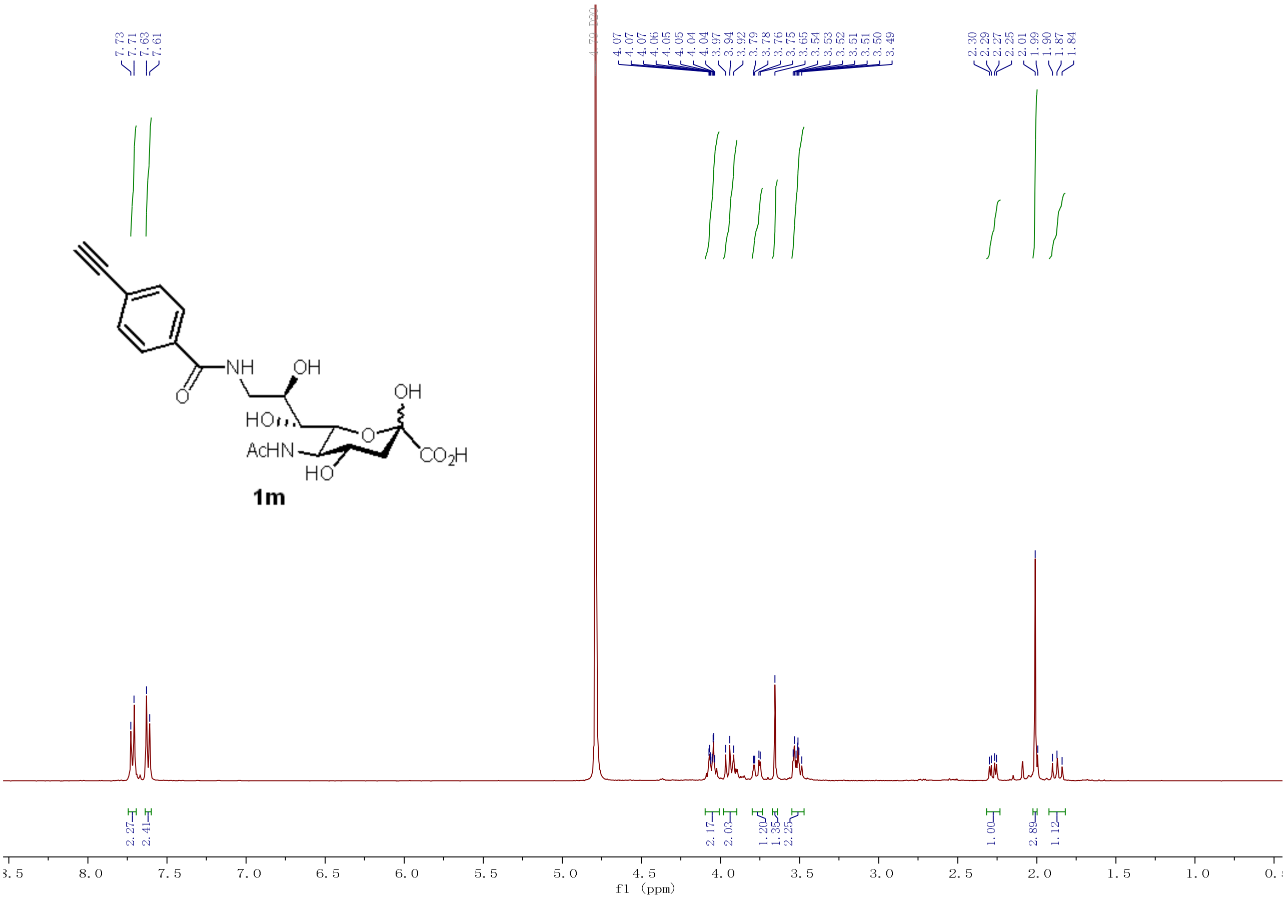

^{1}H NMR spectra of compound **1m**

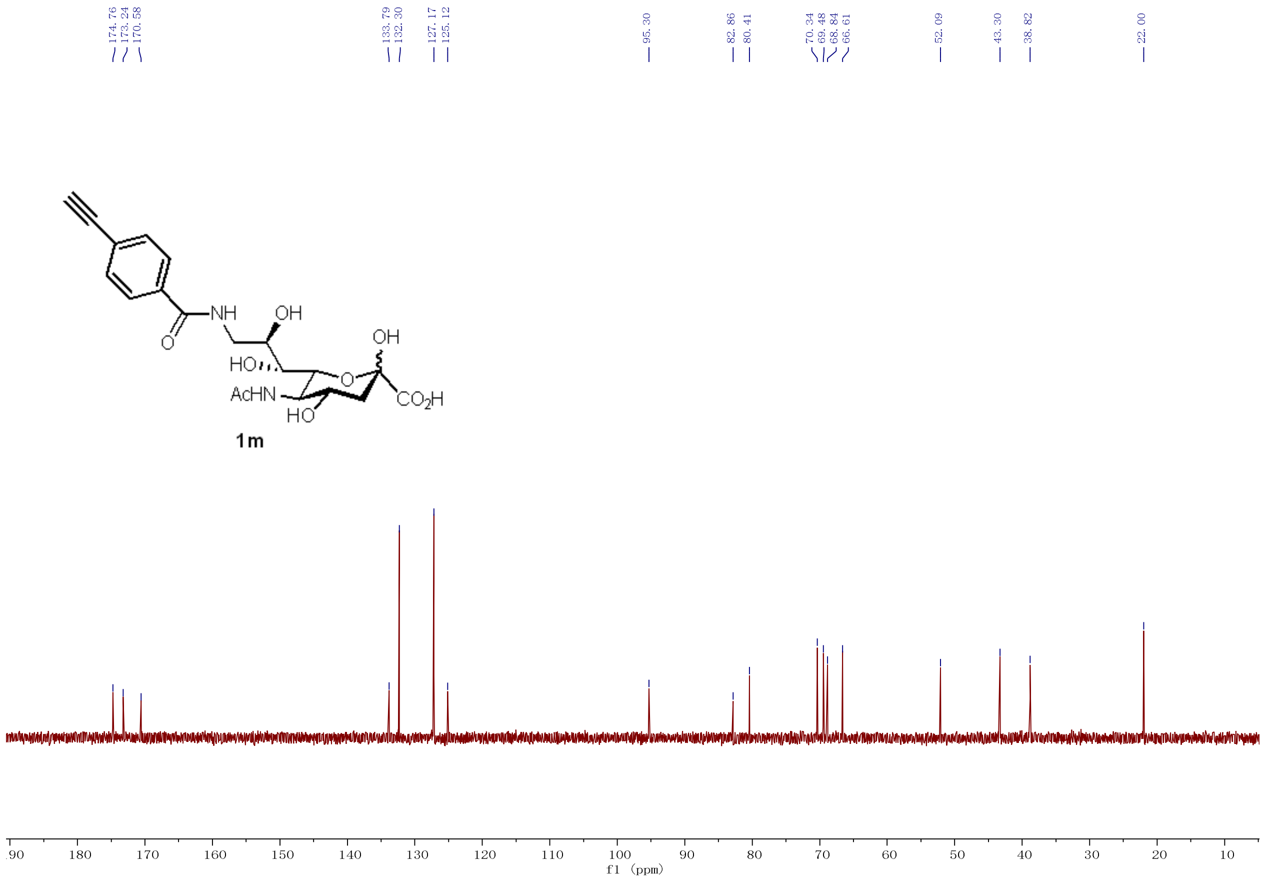

^{13}C NMR spectra of compound **1m**

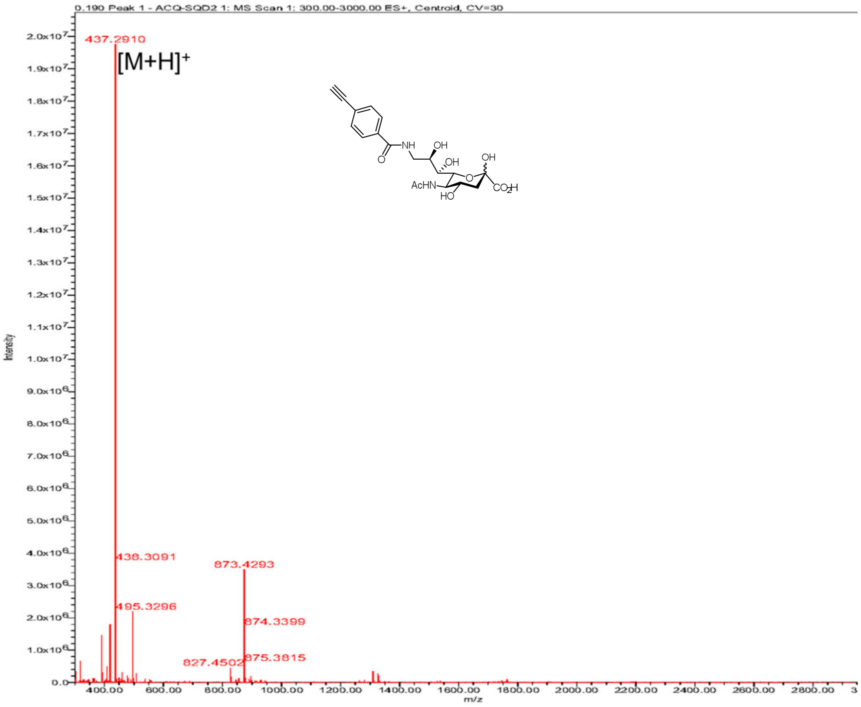

ESI-MS specta of compound **1m**

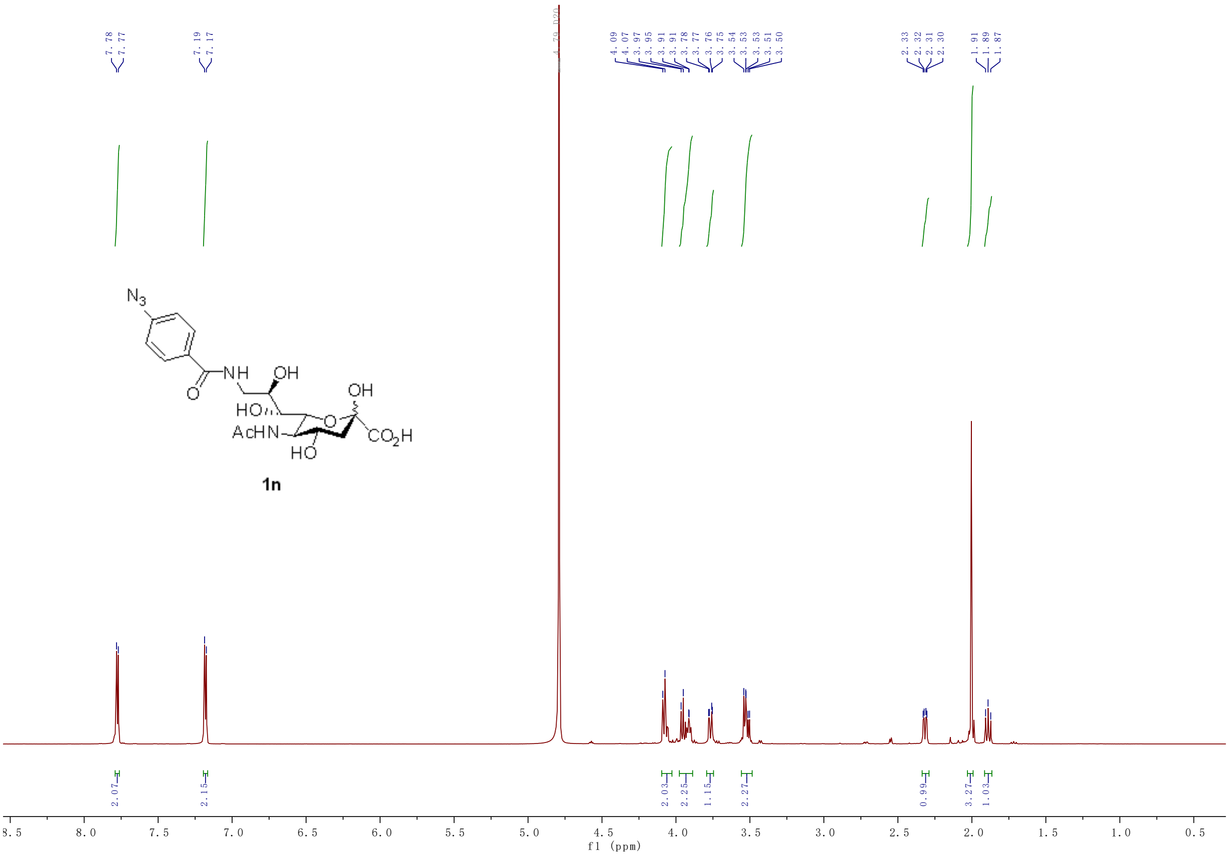

^{1}H NMR spectra of compound **1n**

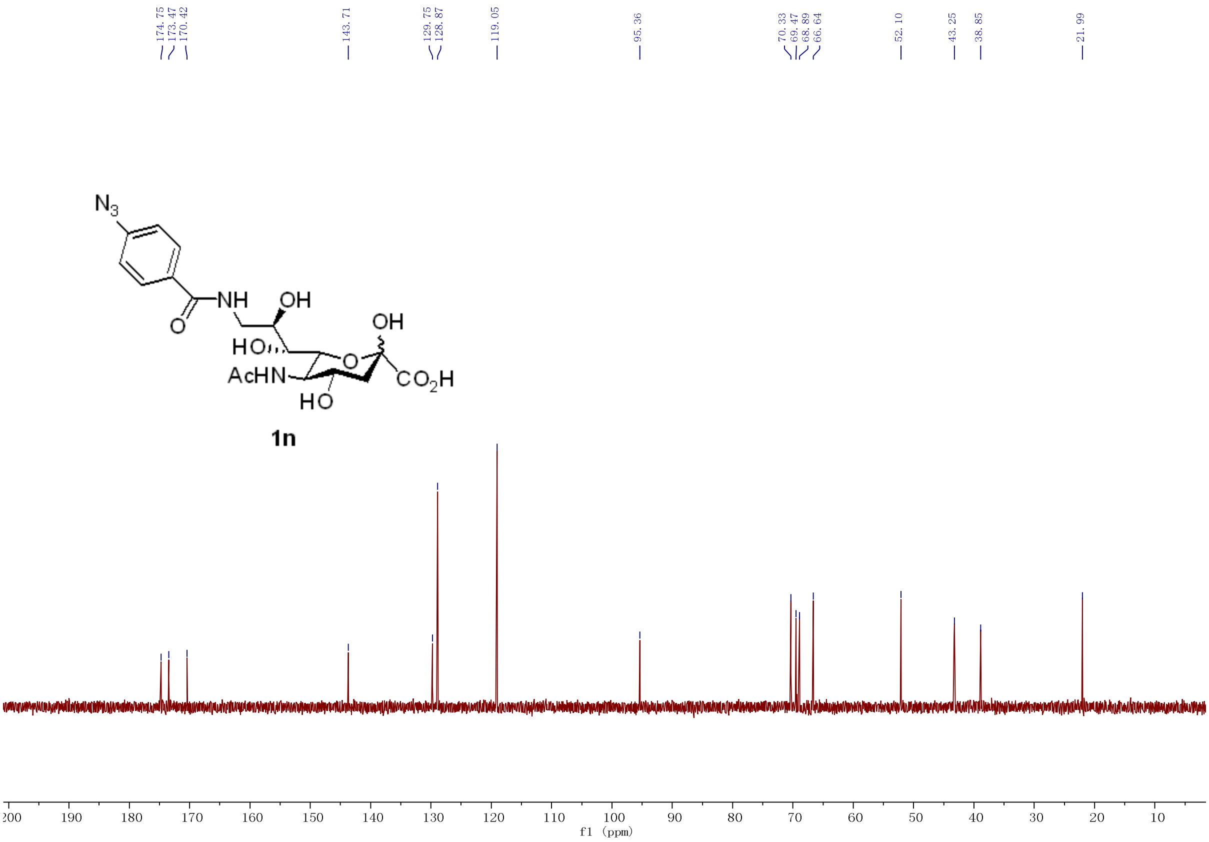

^{13}C NMR spectra of compound **1n**

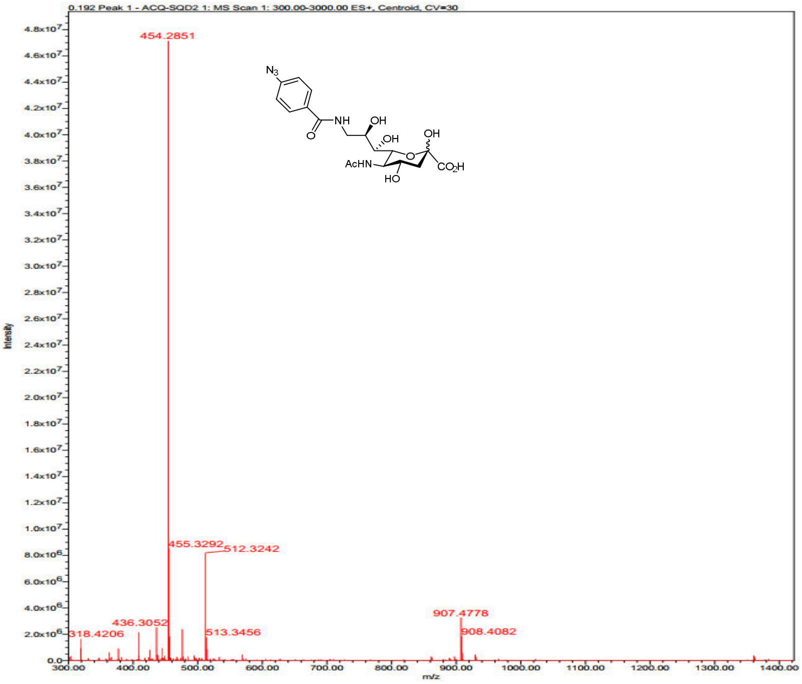

ESI-MS specta of compound **1n**

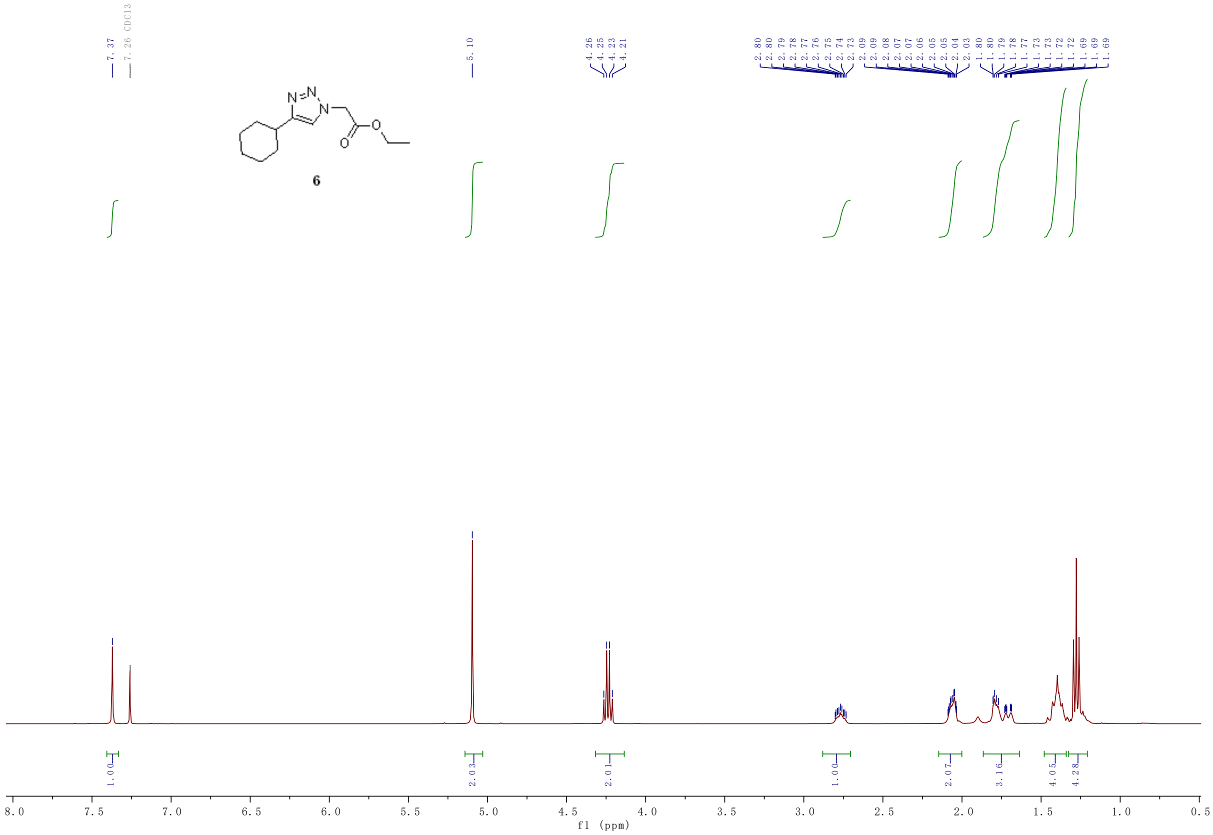

^{1}H NMR spectra of compound **6**

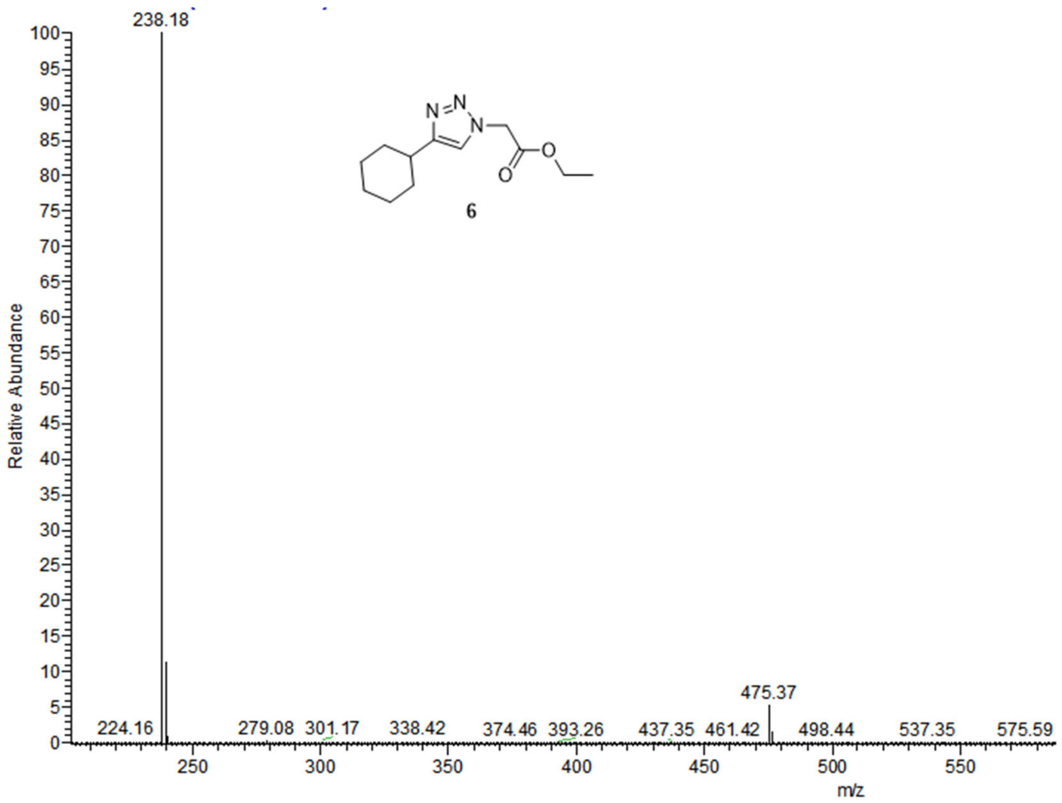

ESI-MS spectra of compound **6**

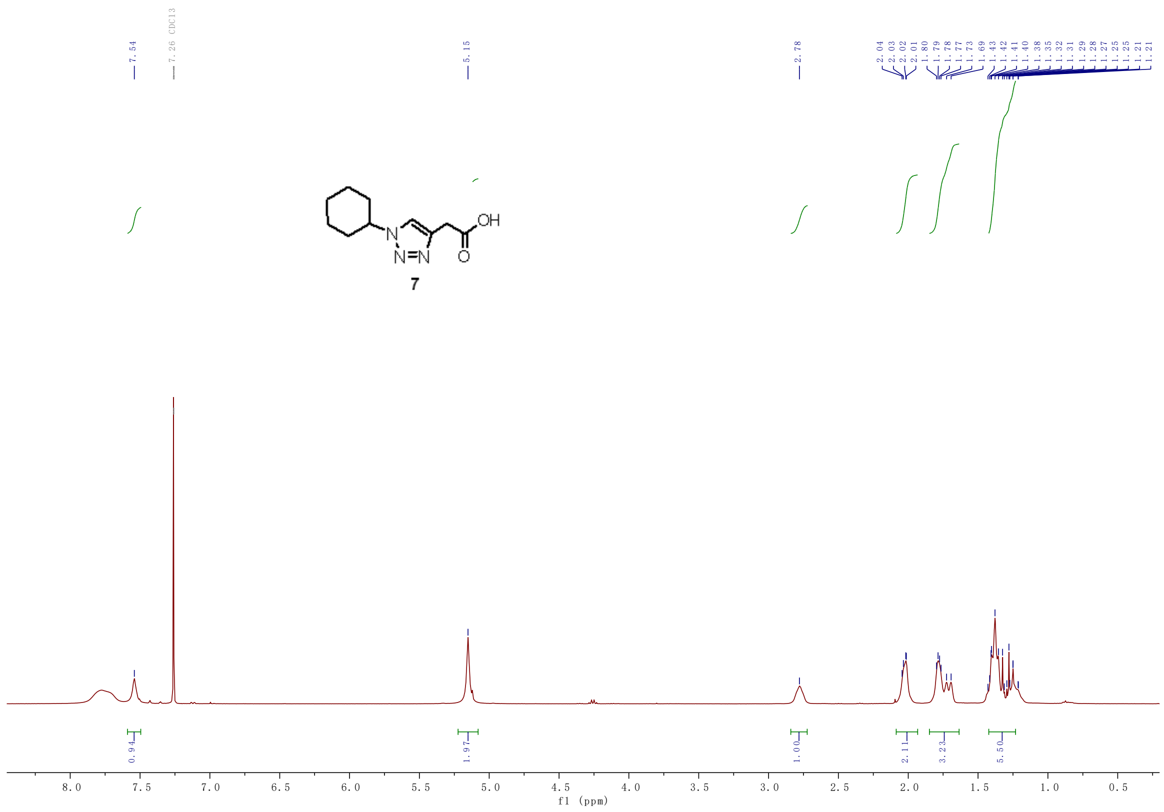

^{1}H NMR spectra of compound **7**

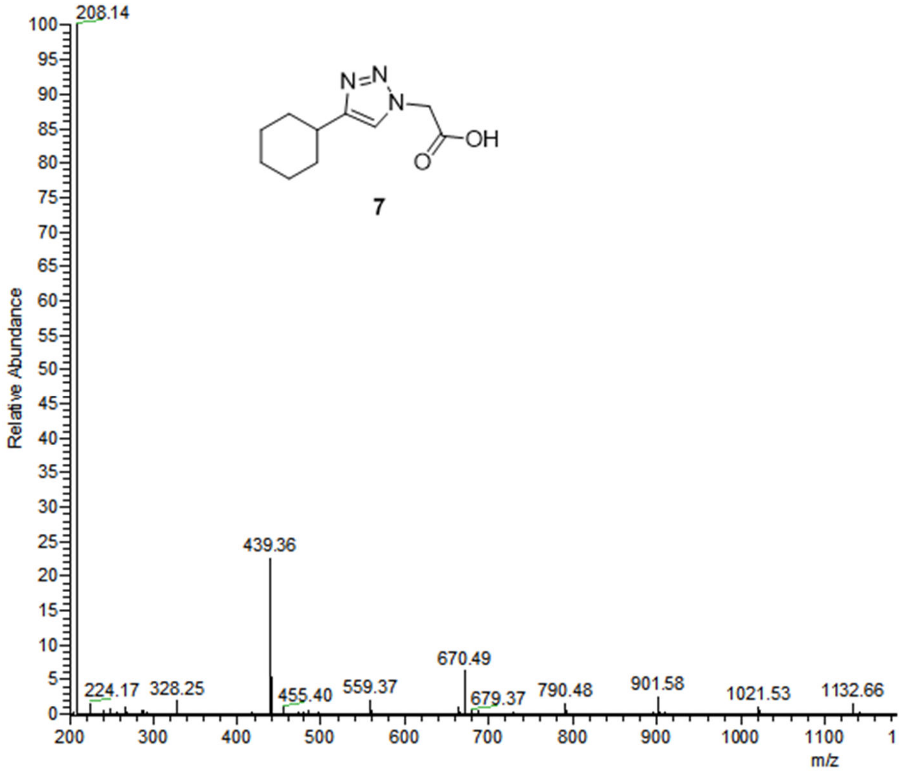

ESI-MS spectra of compound **7**

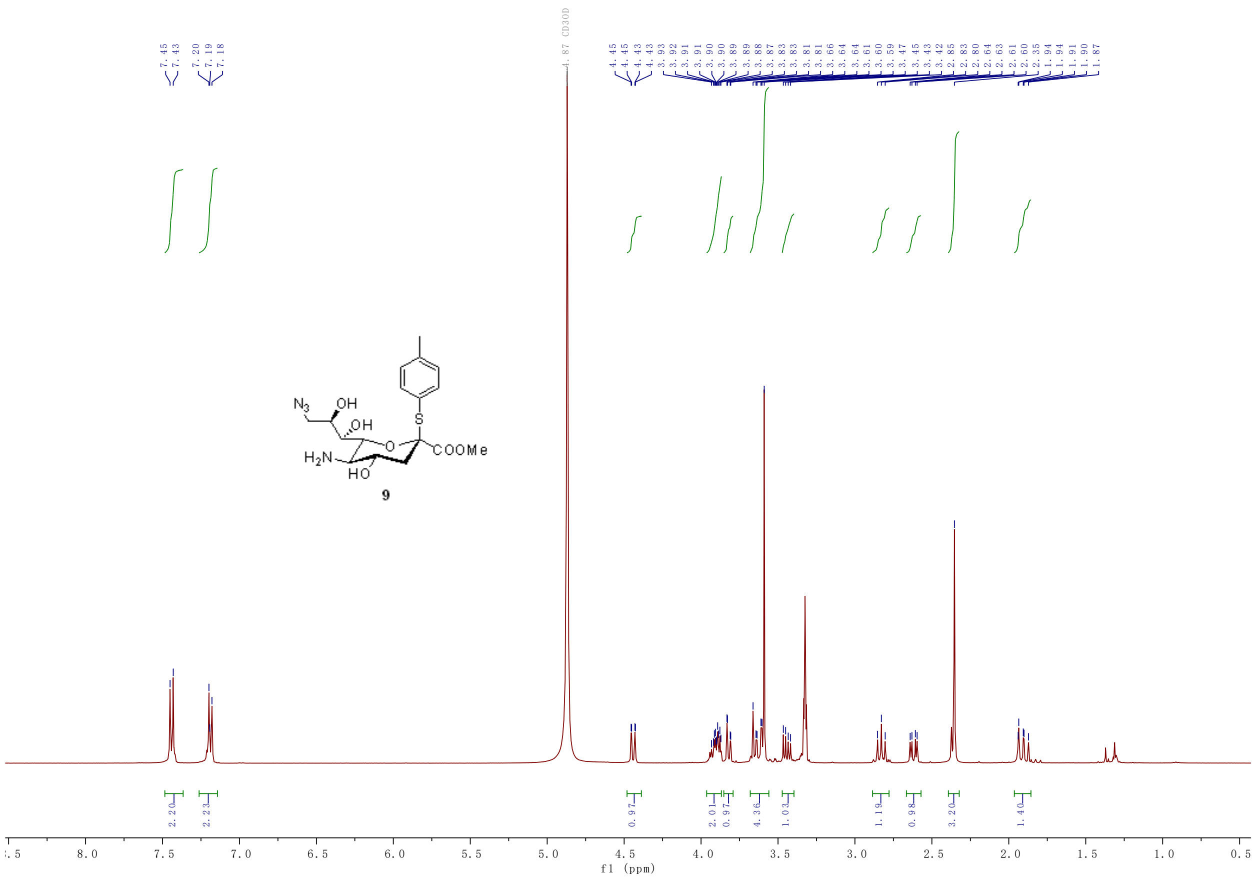

^{1}H NMR spectra of compound **9**

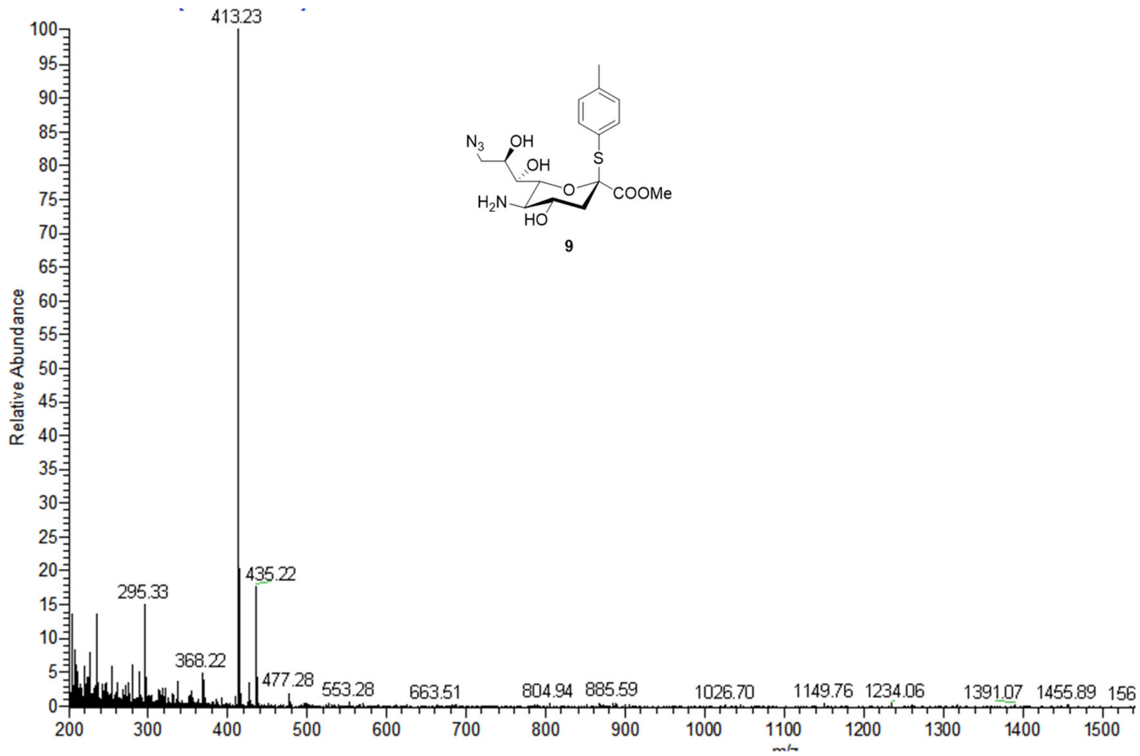

ESI-MS spectra of compound **9**

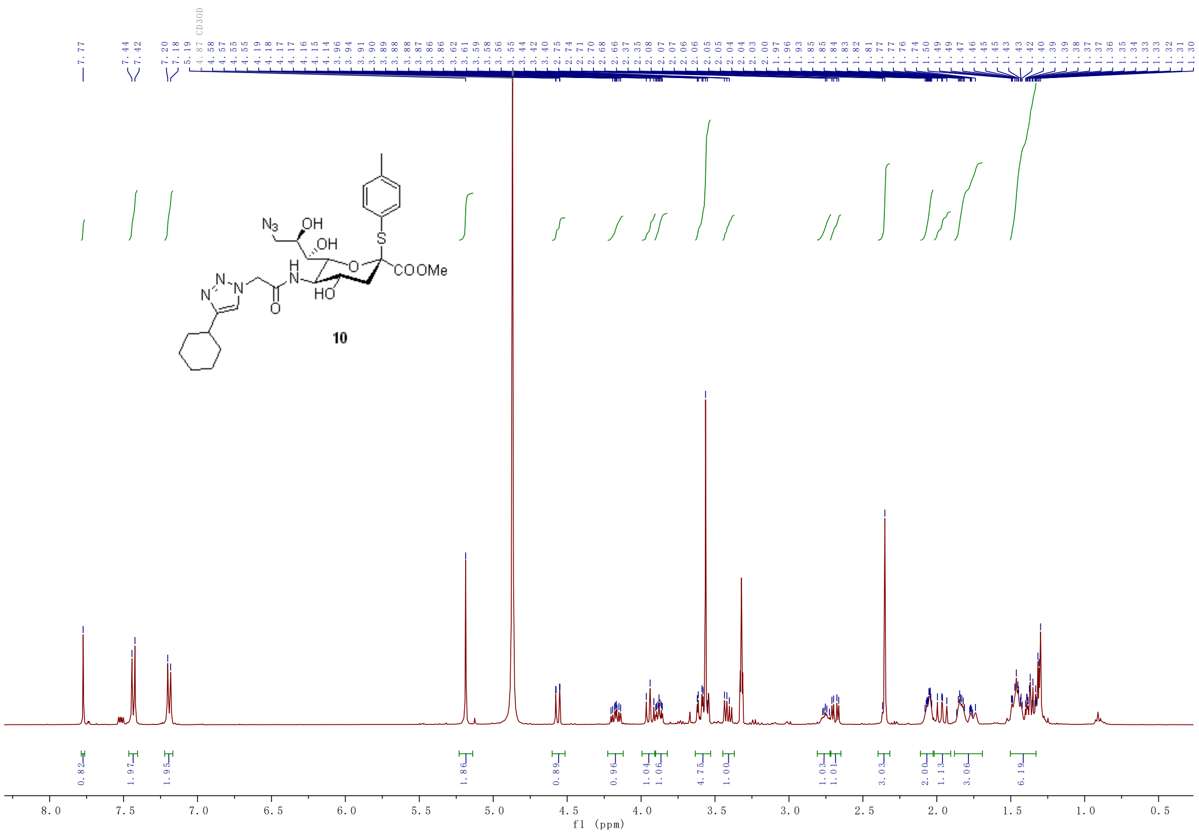

^{1}H NMR spectra of compound **10**

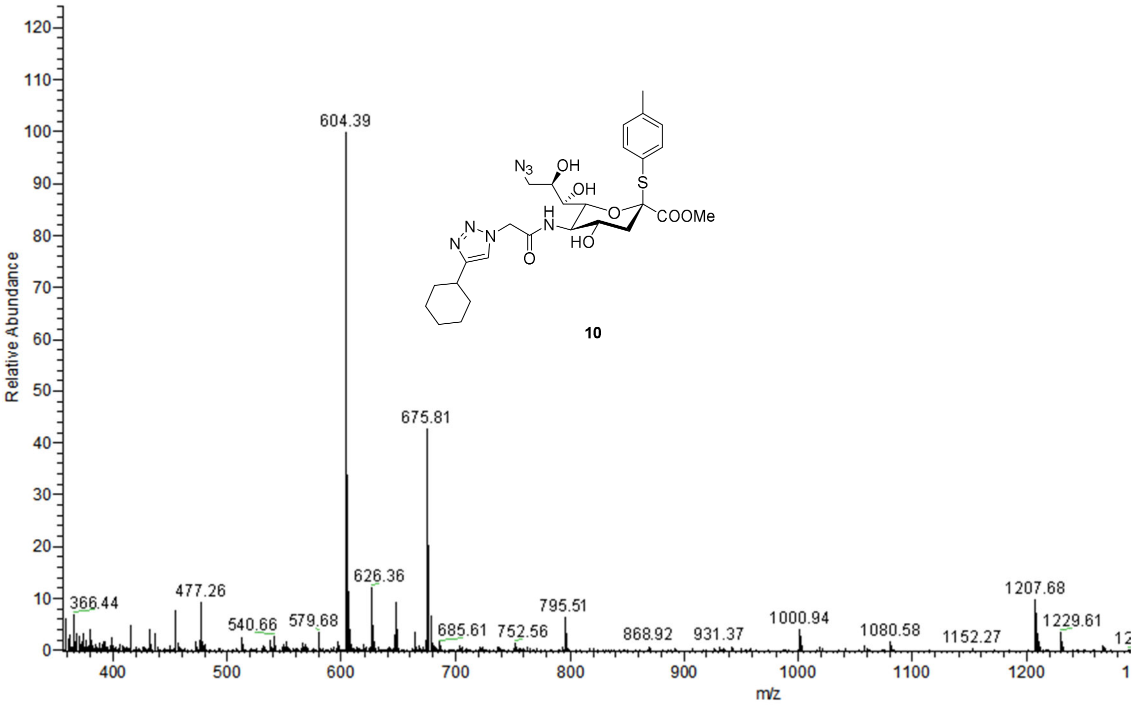

ESI-MS spectra of compound **10**

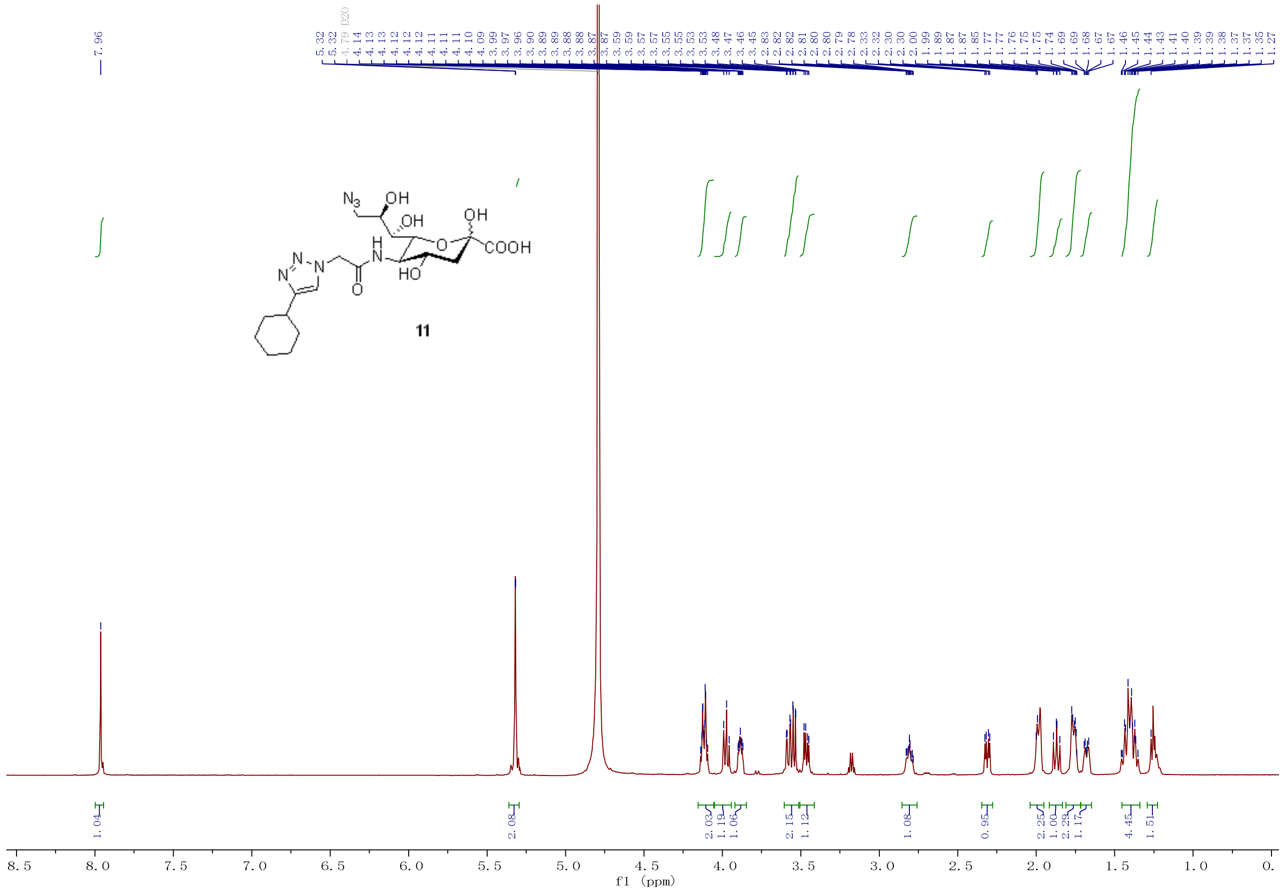

^{1}H NMR spectra of compound **11**

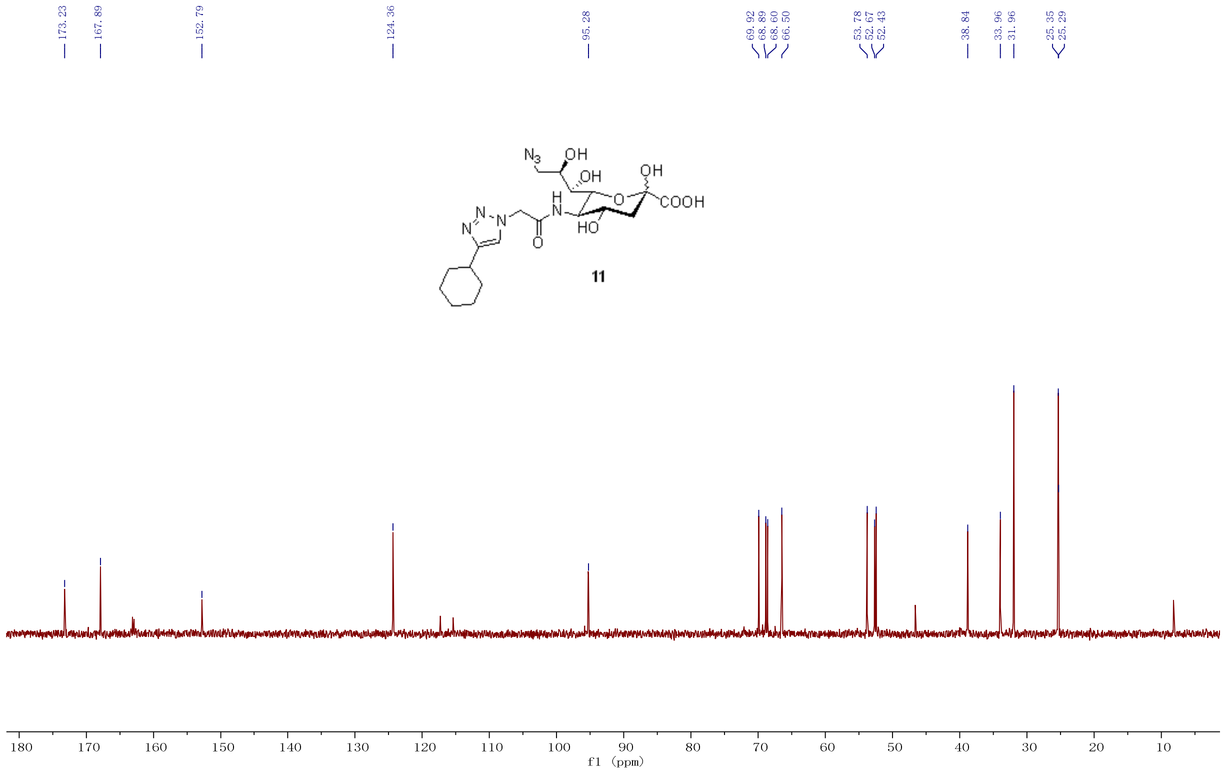

^{13}C NMR spectra of compound **11**

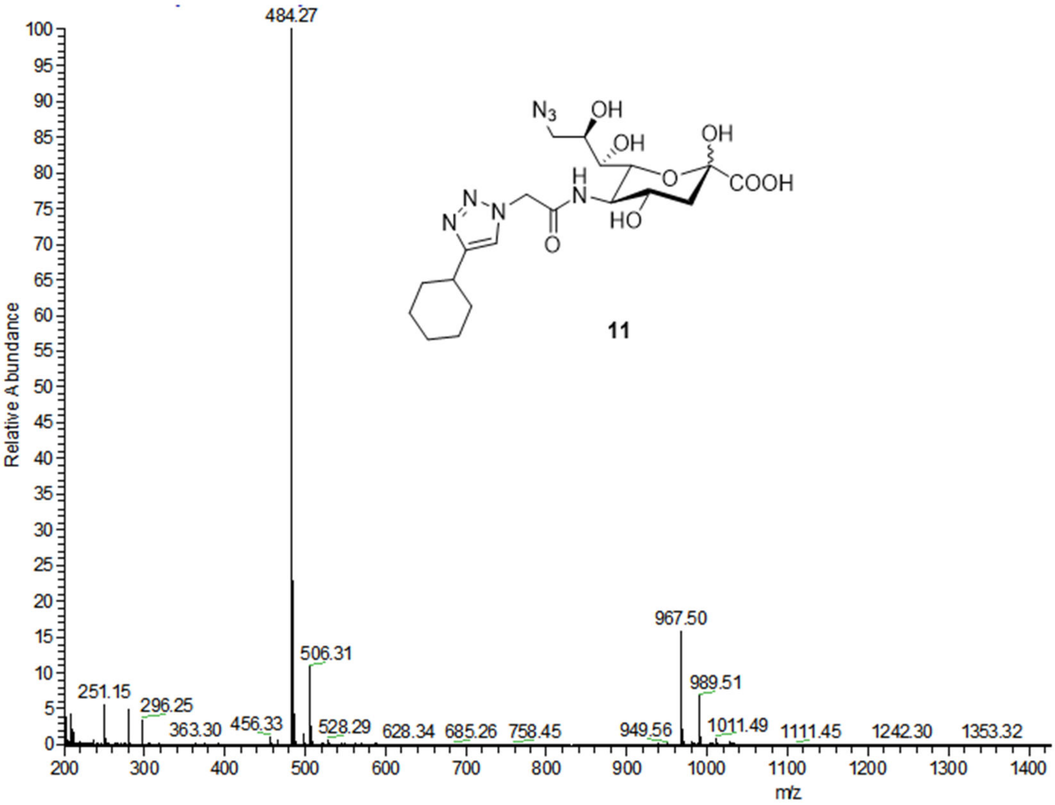

ESI-MS spectra of compound **11**

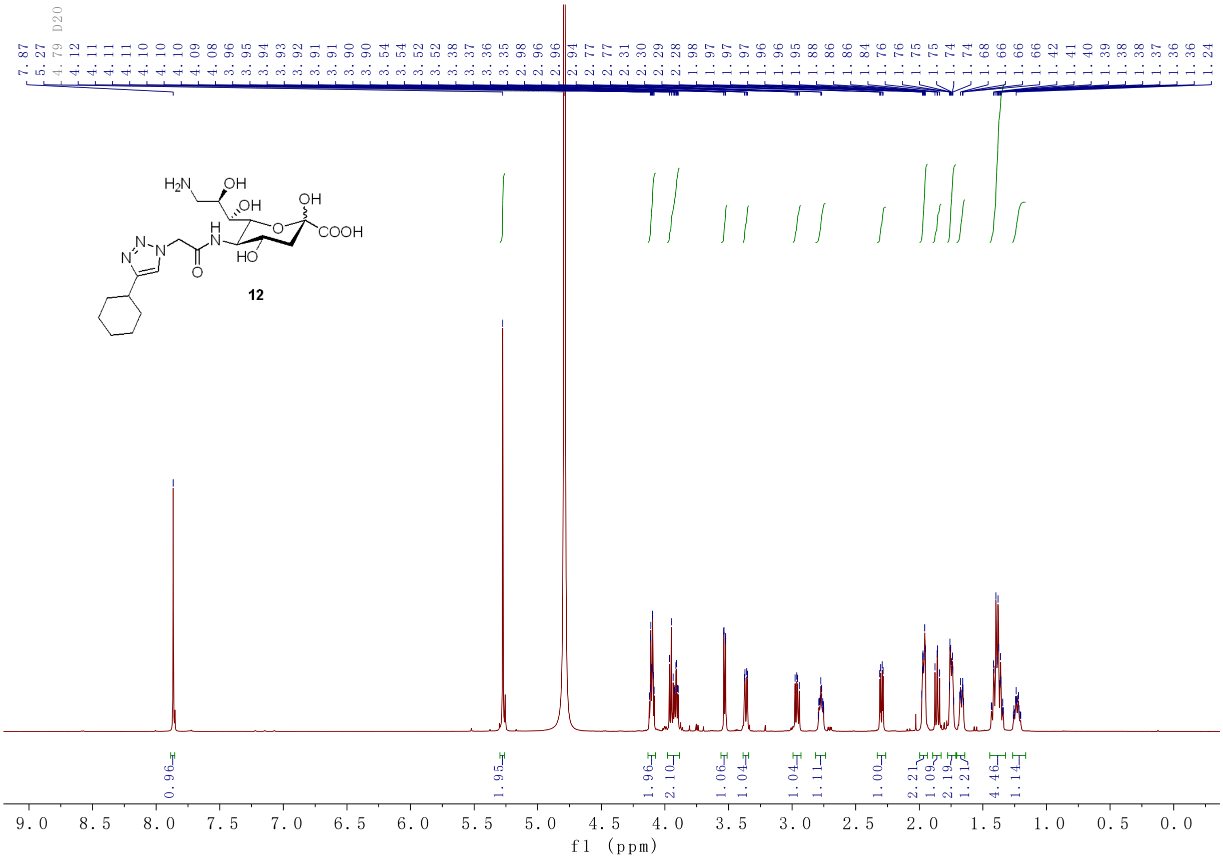

^{1}H NMR spectra of compound **12**

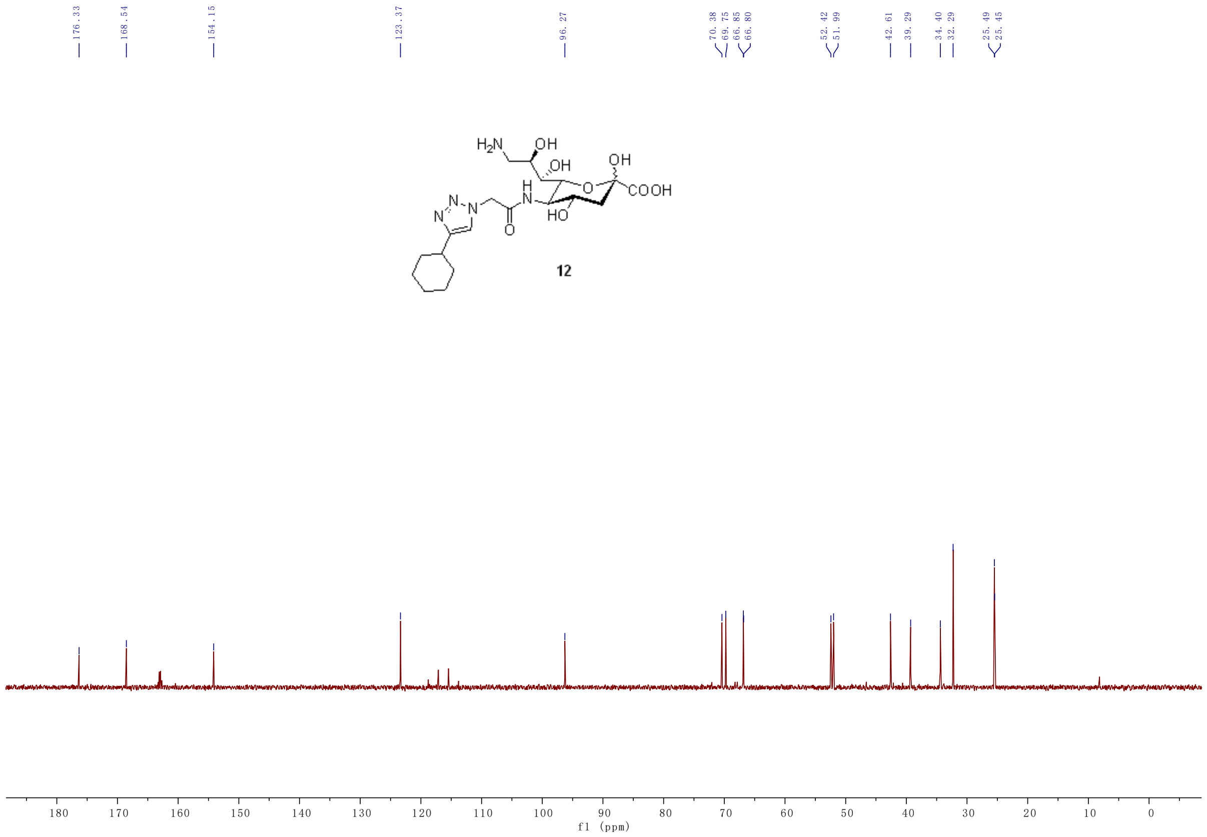

^{13}C NMR spectra of compound **12**

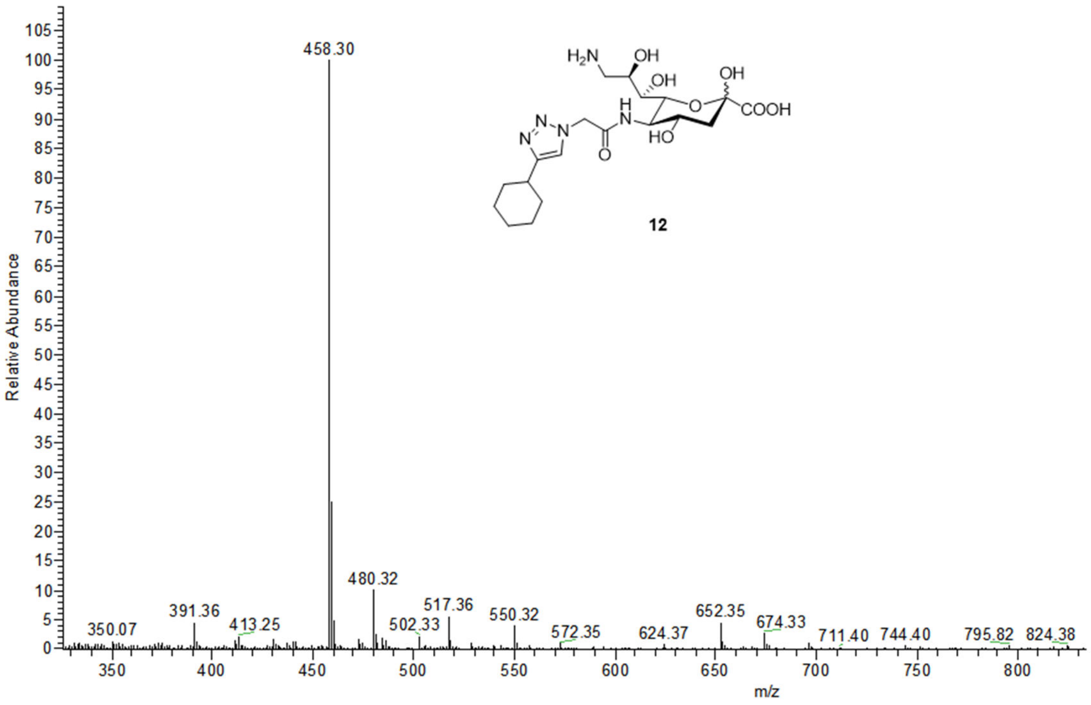

ESI-MS spectra of compound **12**

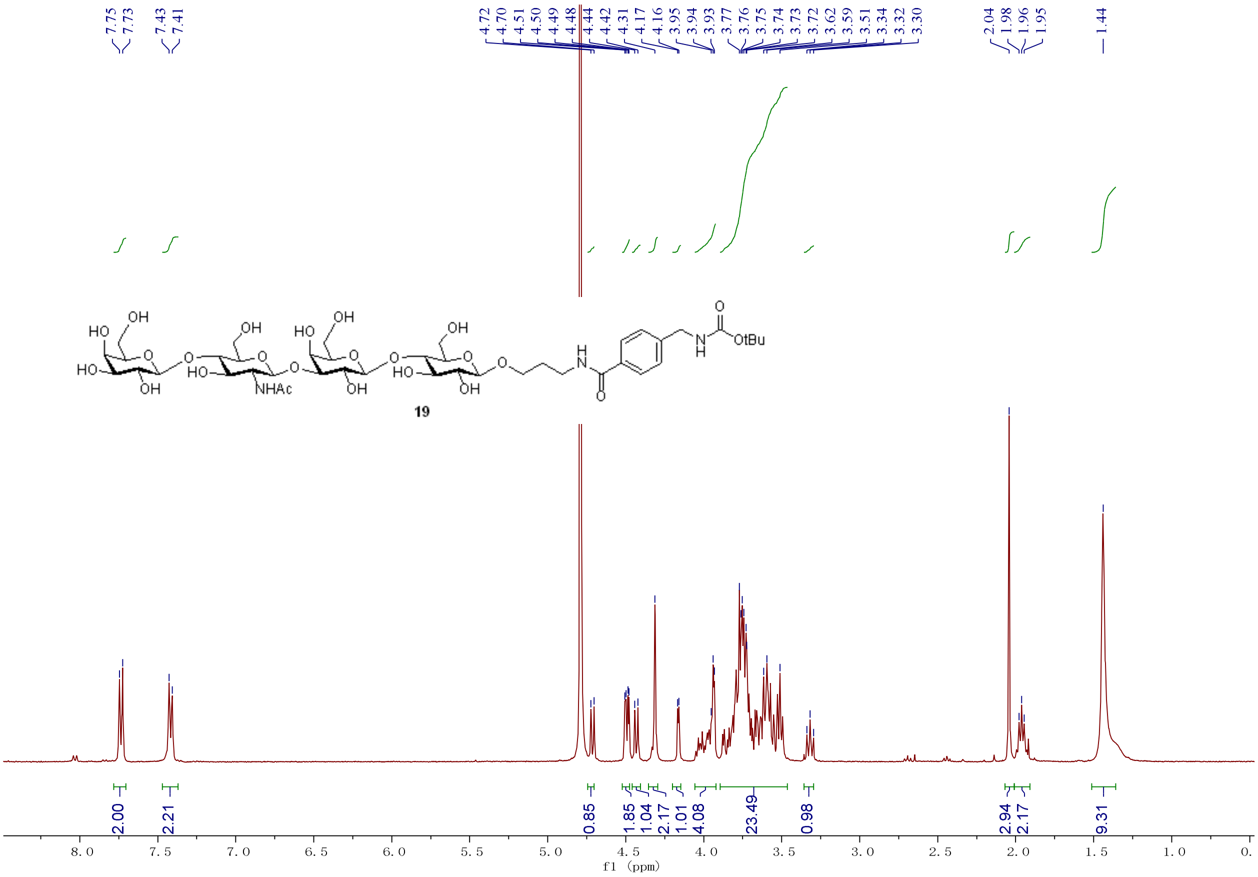

^{1}H NMR spectra of compound **19**

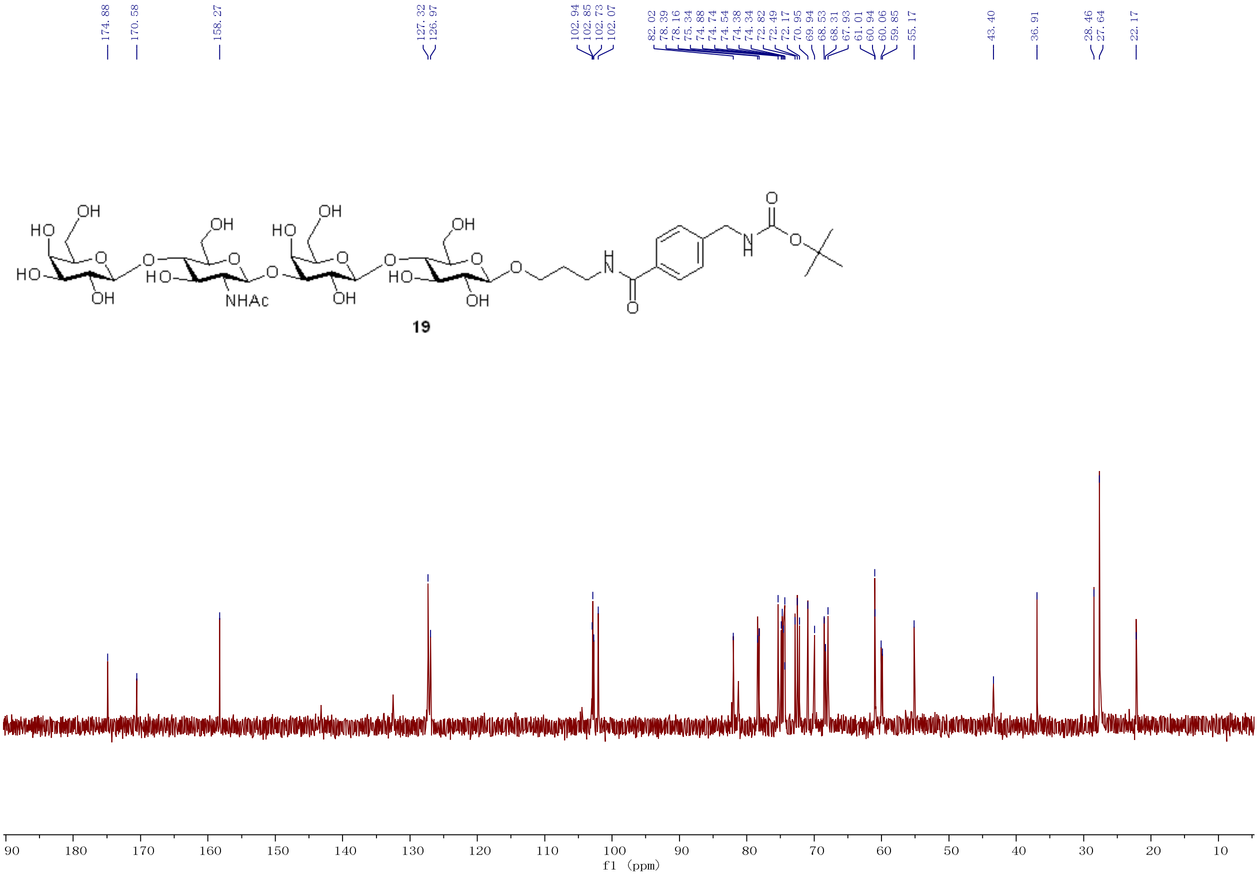

^{13}C NMR spectra of compound **19**

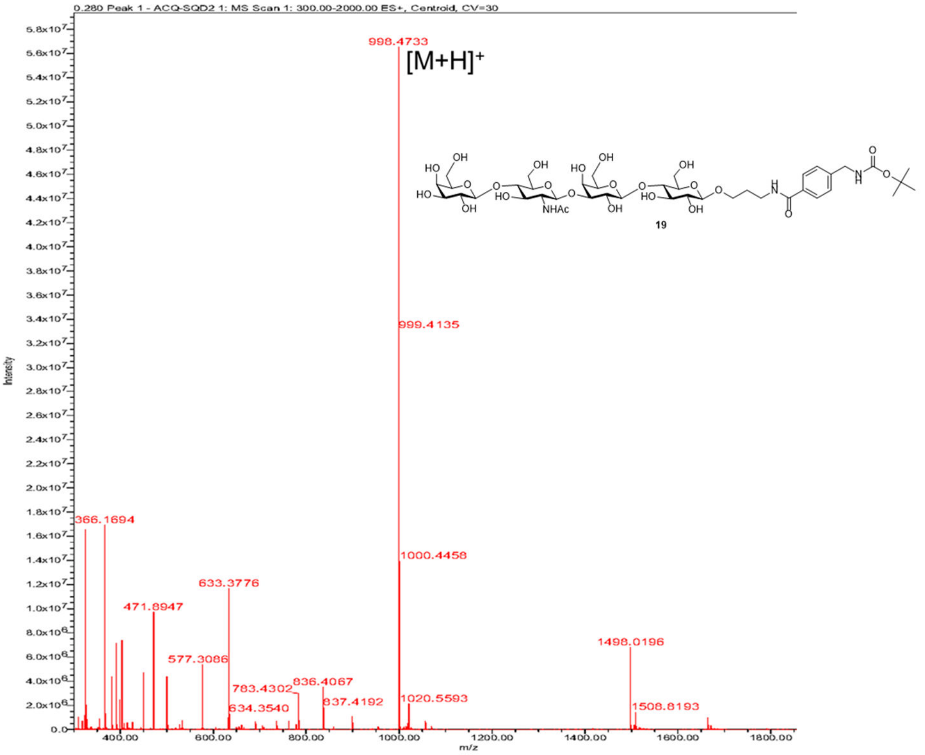

ESI-MS spectra of compound **19**

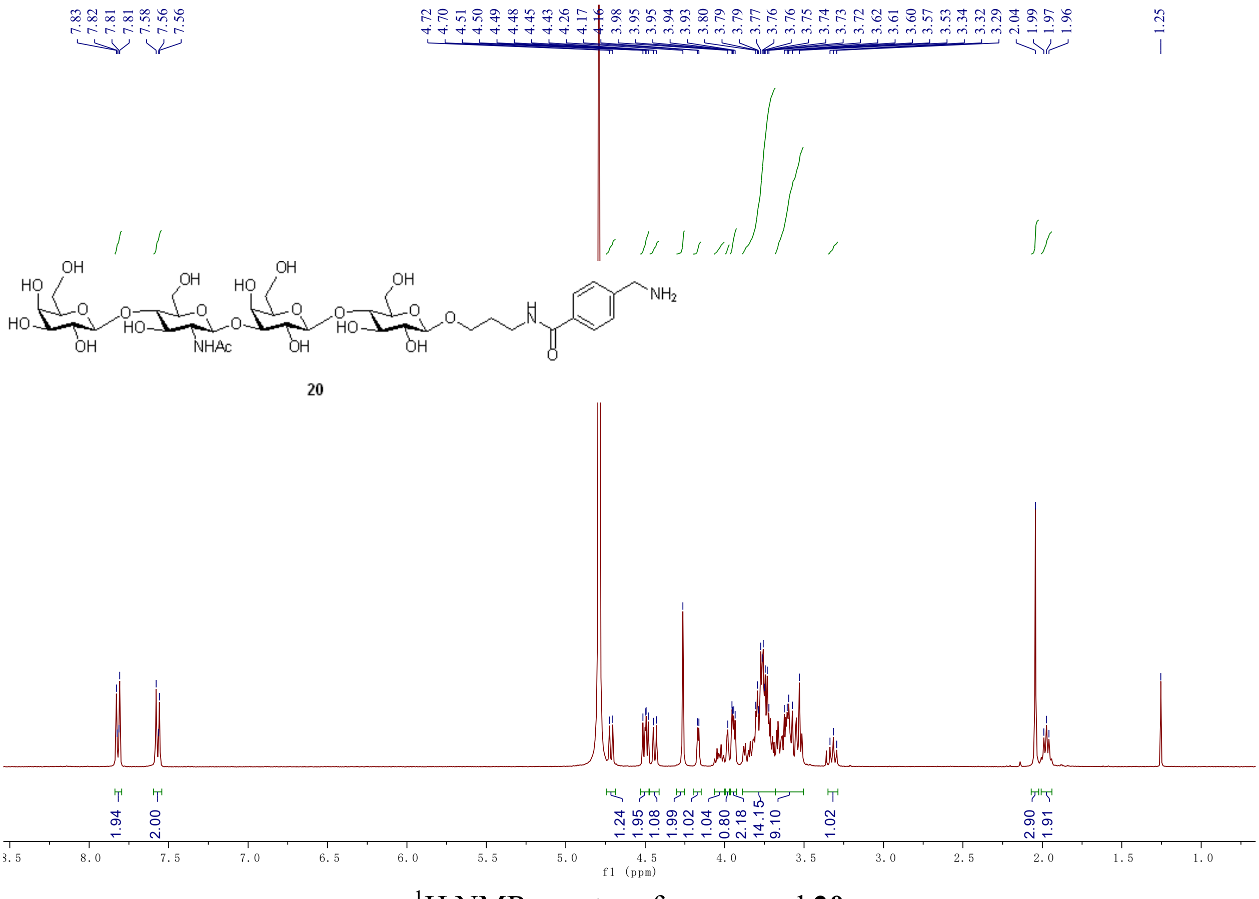

^{1}H NMR spectra of compound **20**

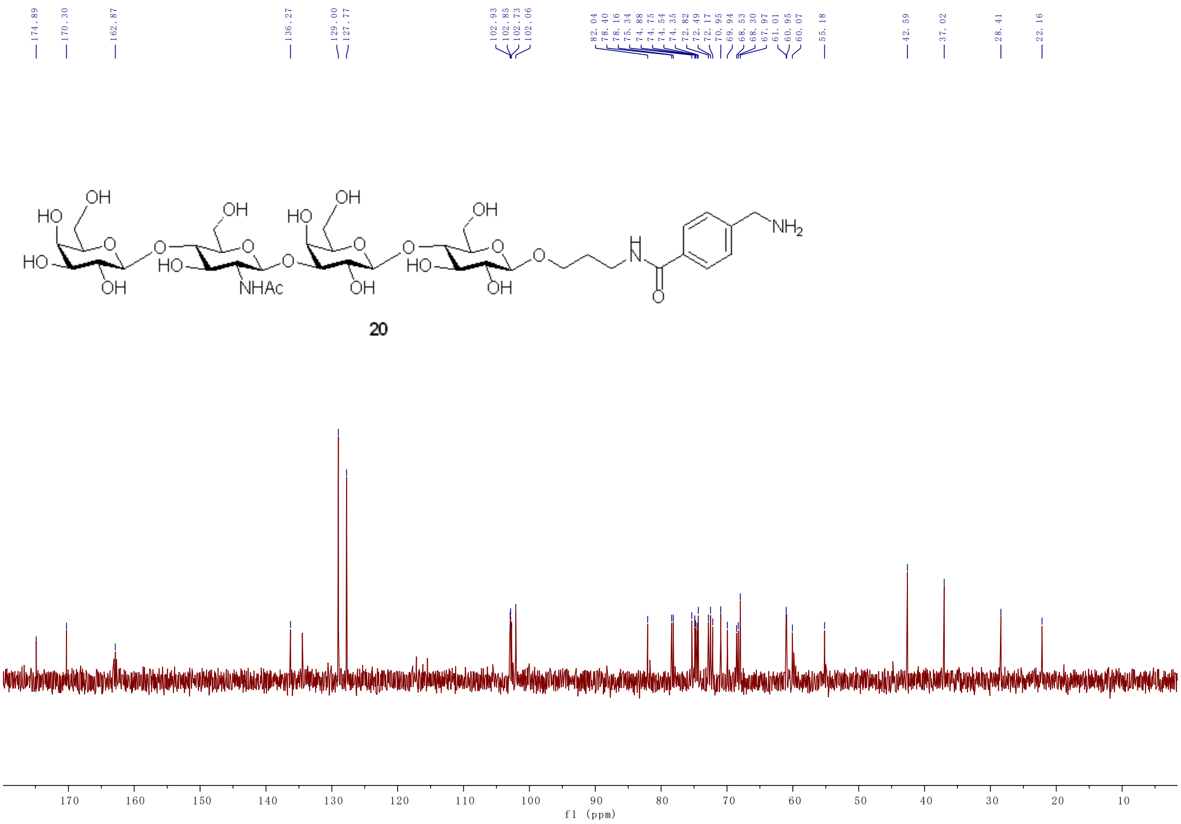

^{13}C NMR spectra of compound **20**

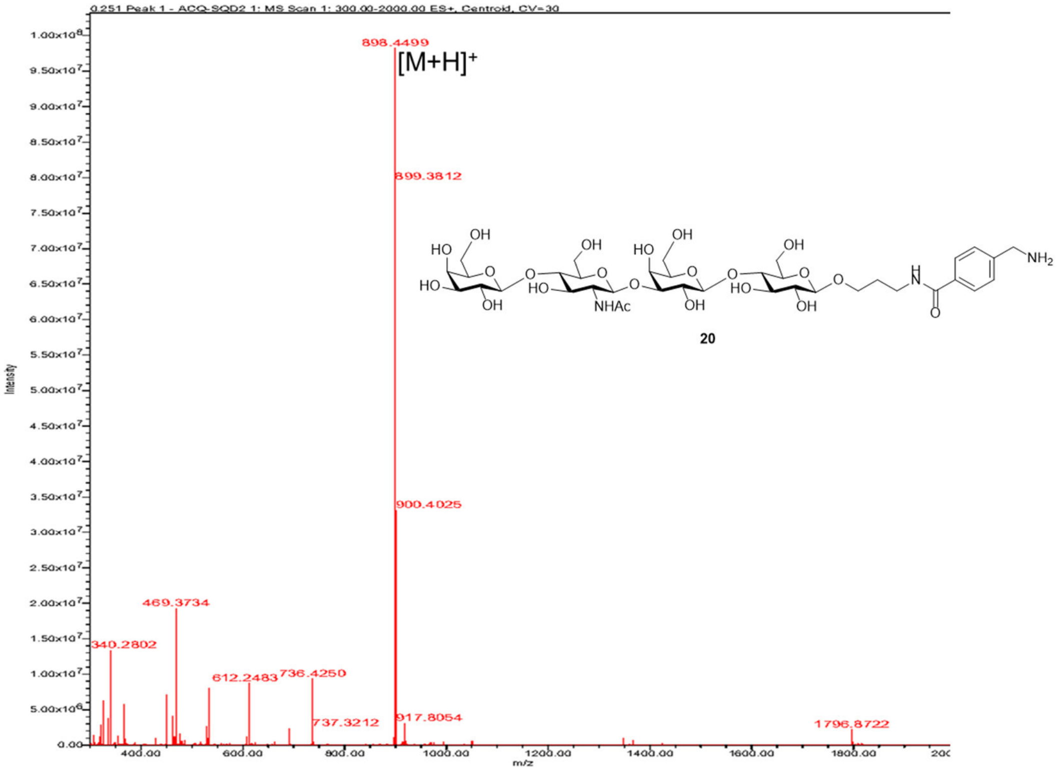

ESI-MS spectra of compound **20**

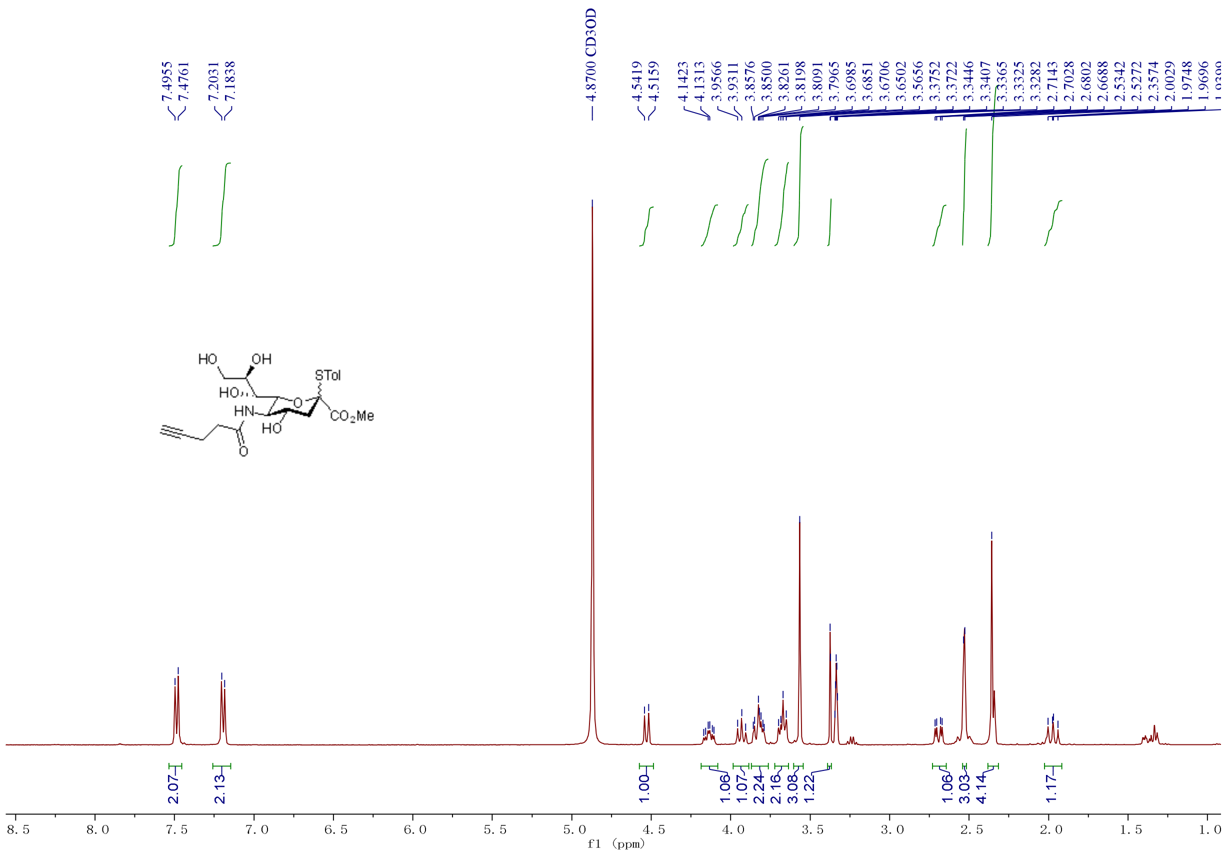

^{1}H NMR spectra of compound **23**

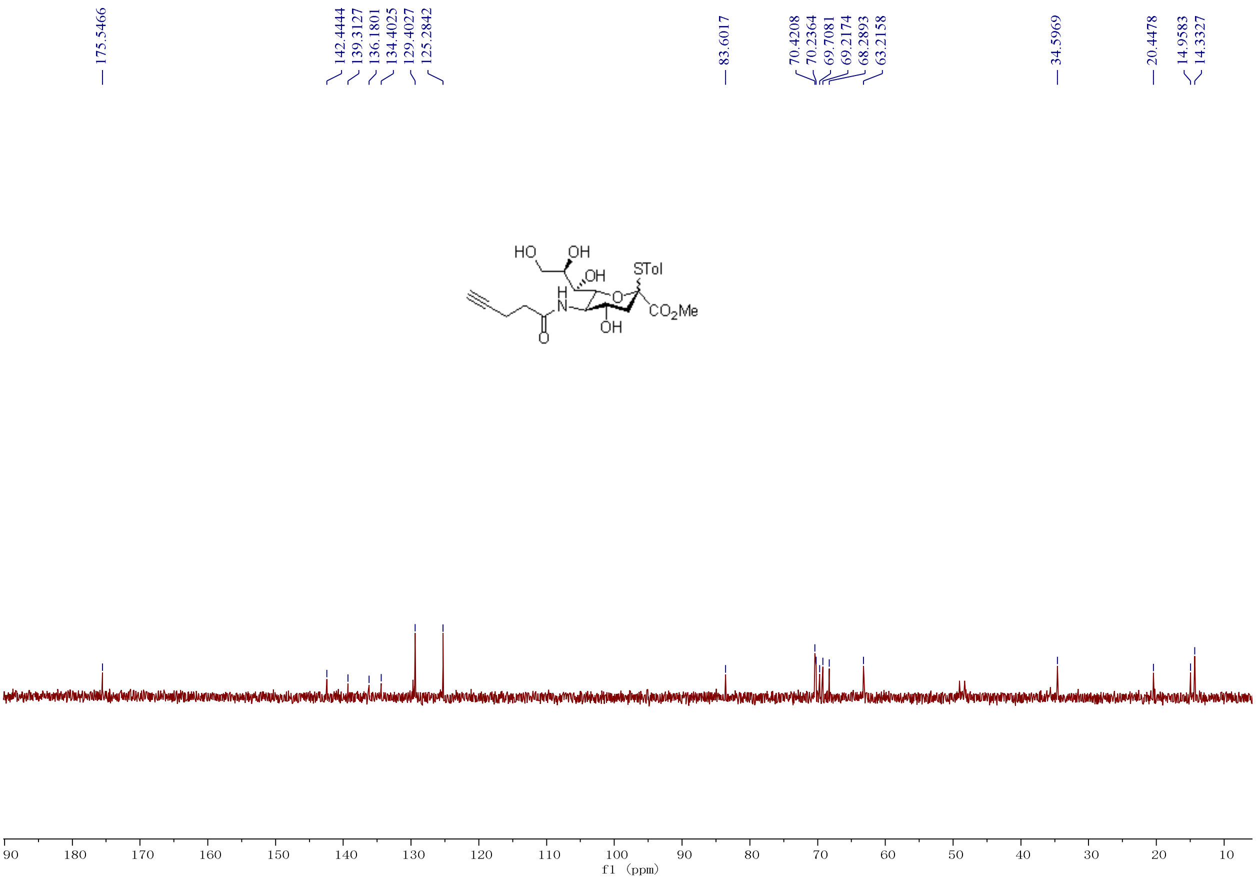

^{13}C NMR spectra of compound **23**

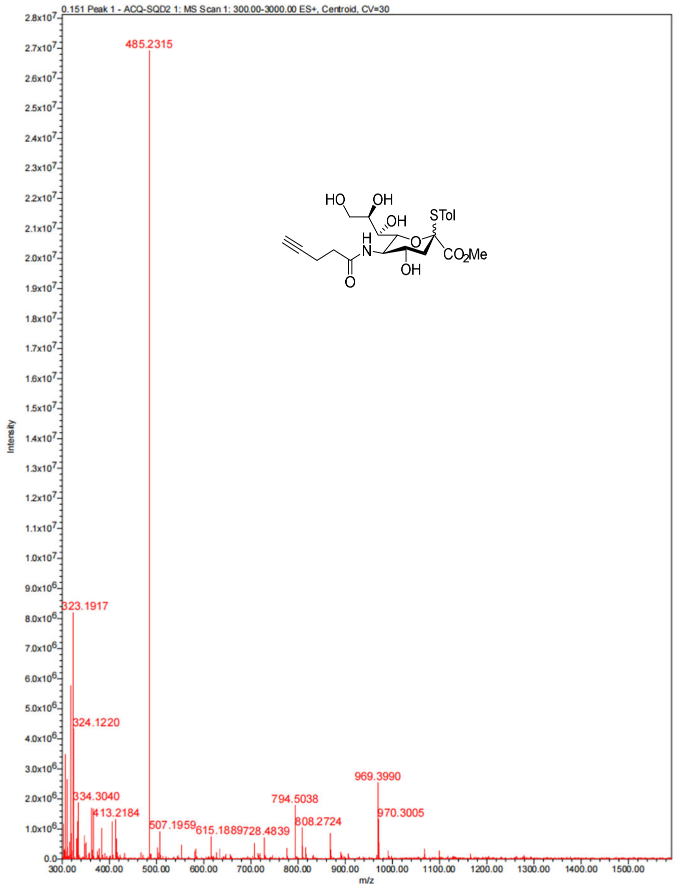

ESI-MS spectra of compound **23**

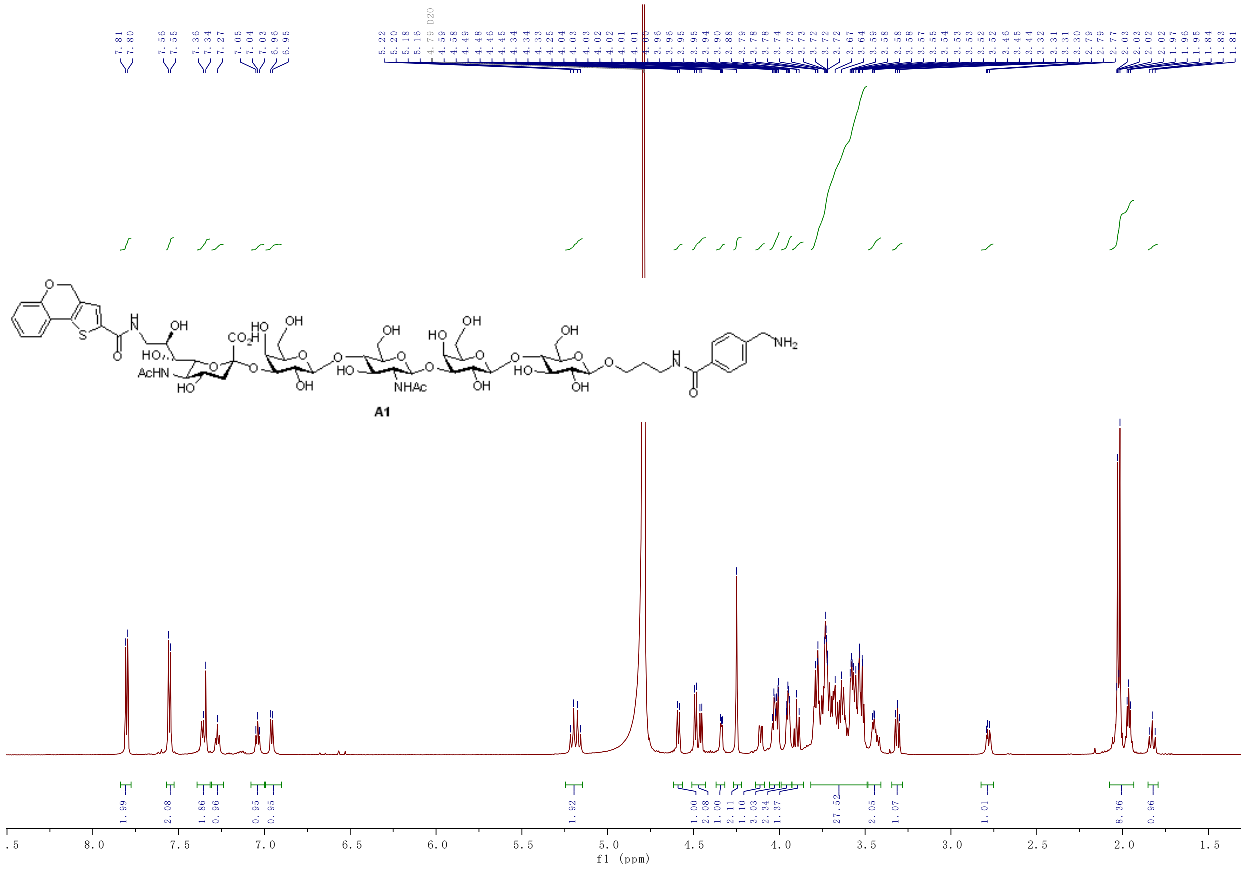

^{1}H NMR spectra of compound **A1**

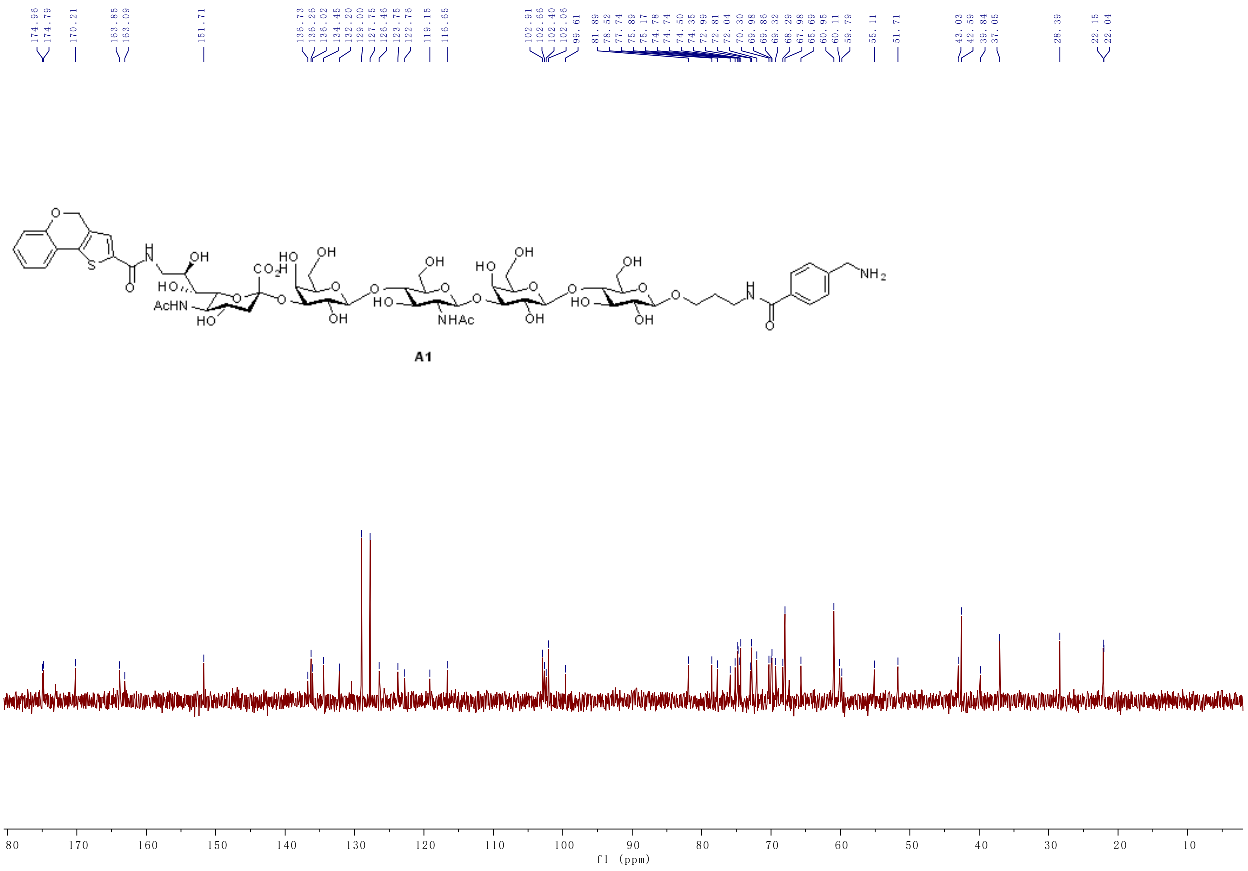

^{13}C NMR spectra of compound **A1**

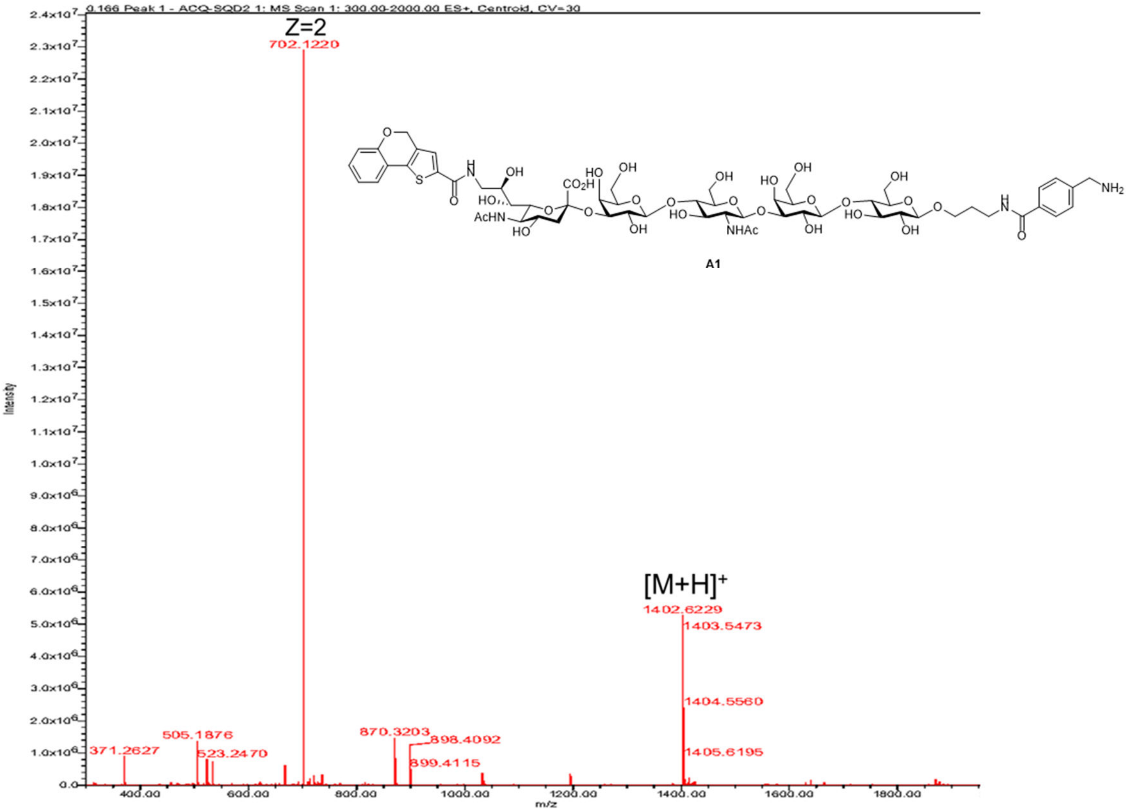

ESI-MS spectra of compound **A1**

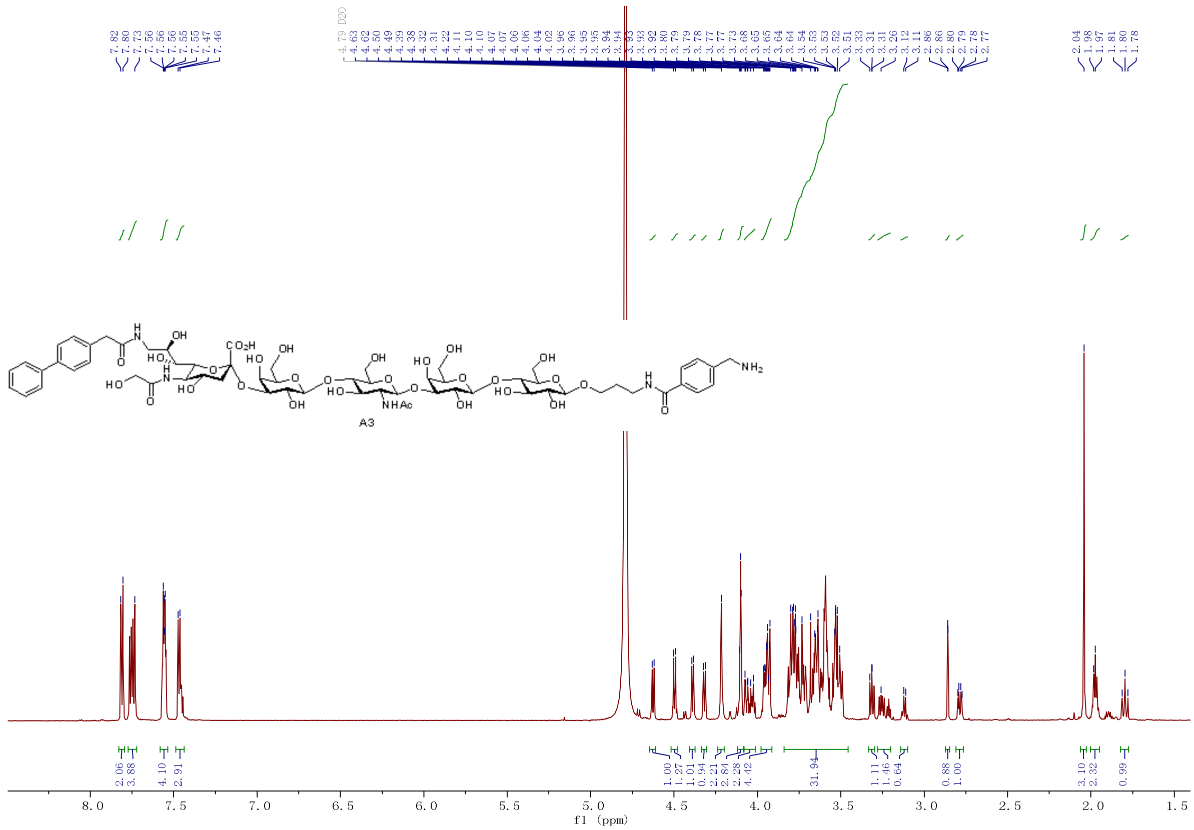

^{1}H NMR spectra of compound **A3**

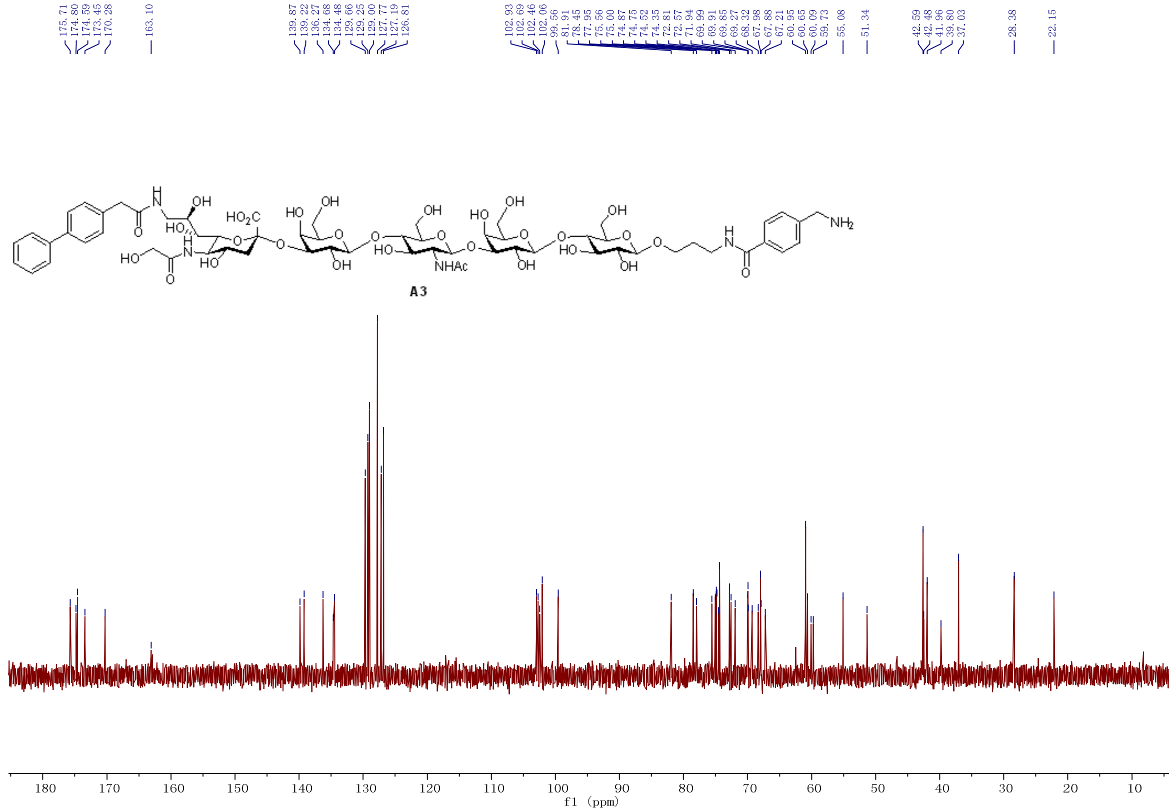

^{13}C NMR spectra of compound **A3**

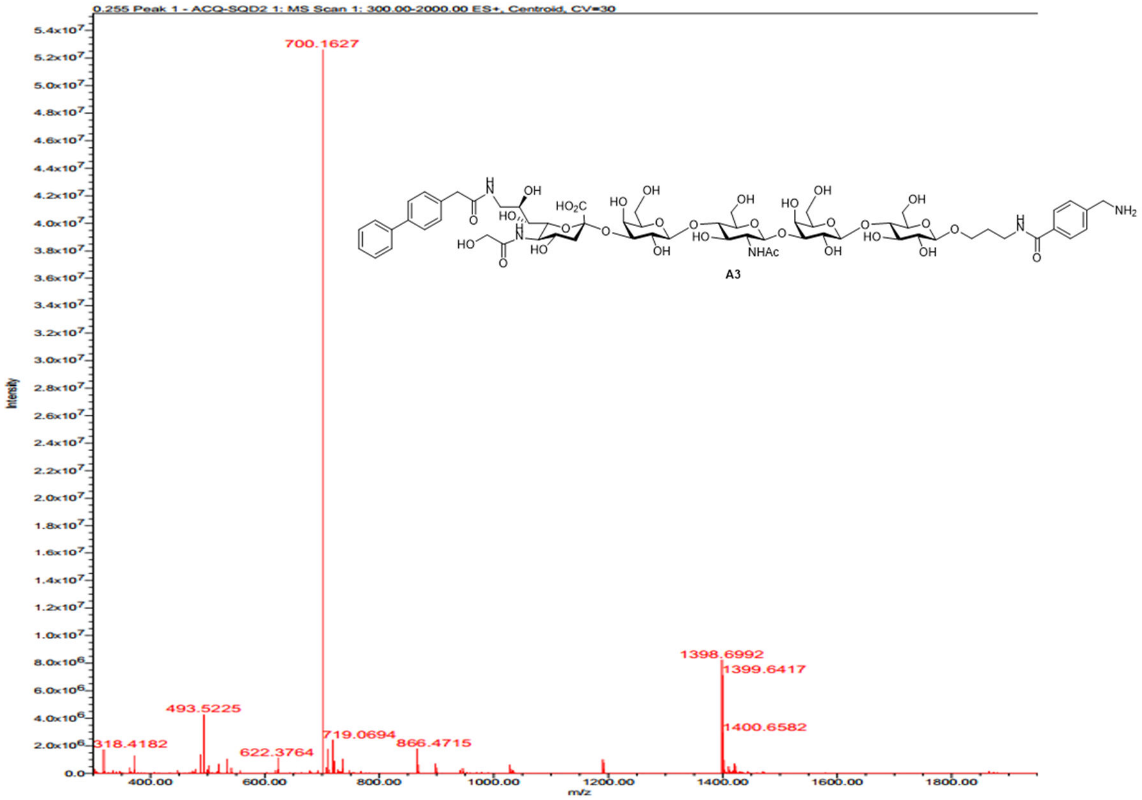

ESI-MS spectra of compound **A3**

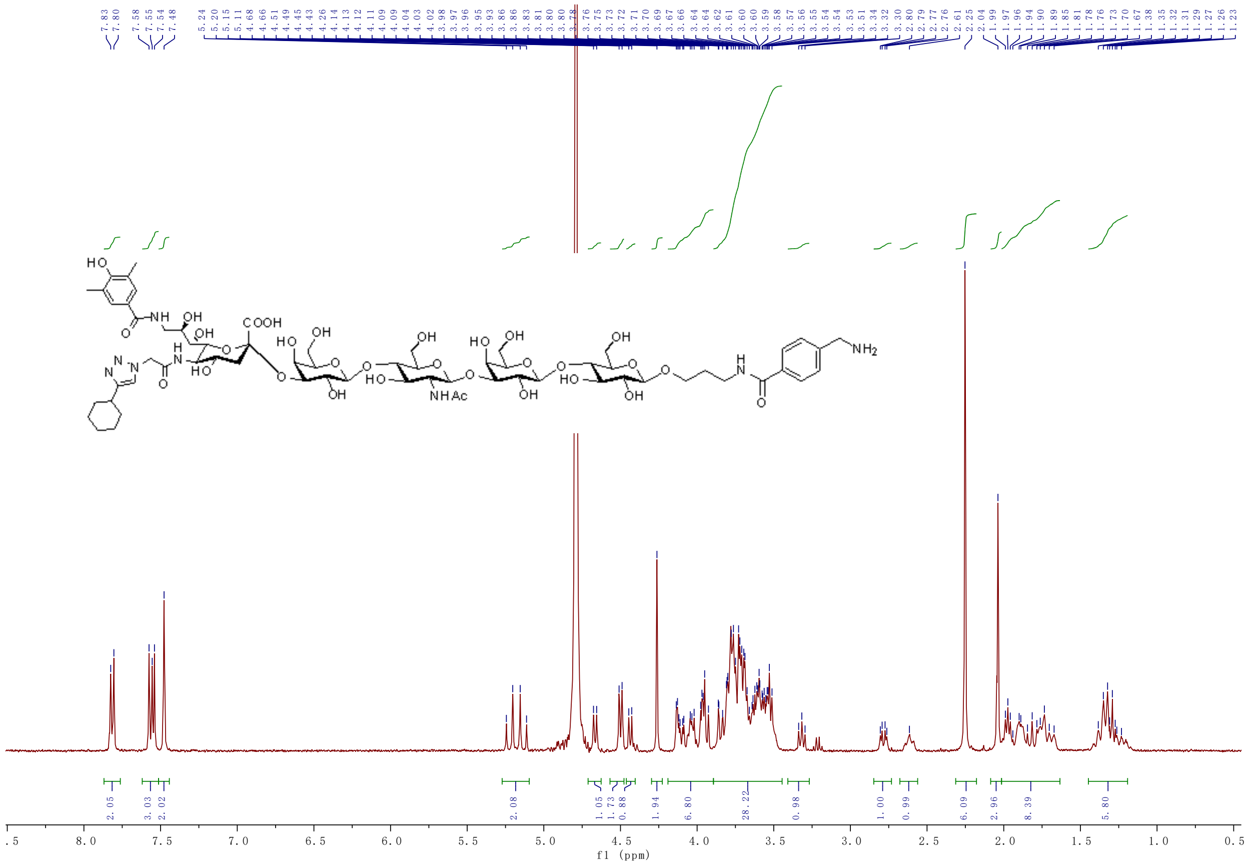

^{1}H NMR spectra of compound **A4**

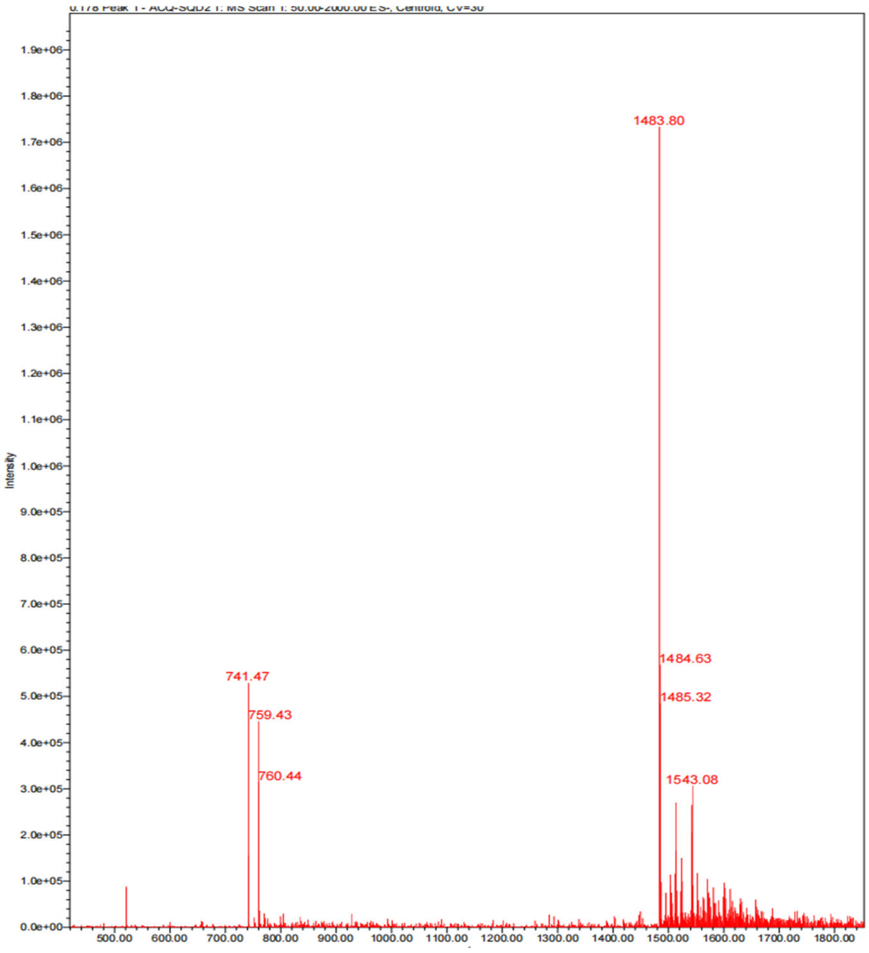

ESI-MS spectra of compound **A4**

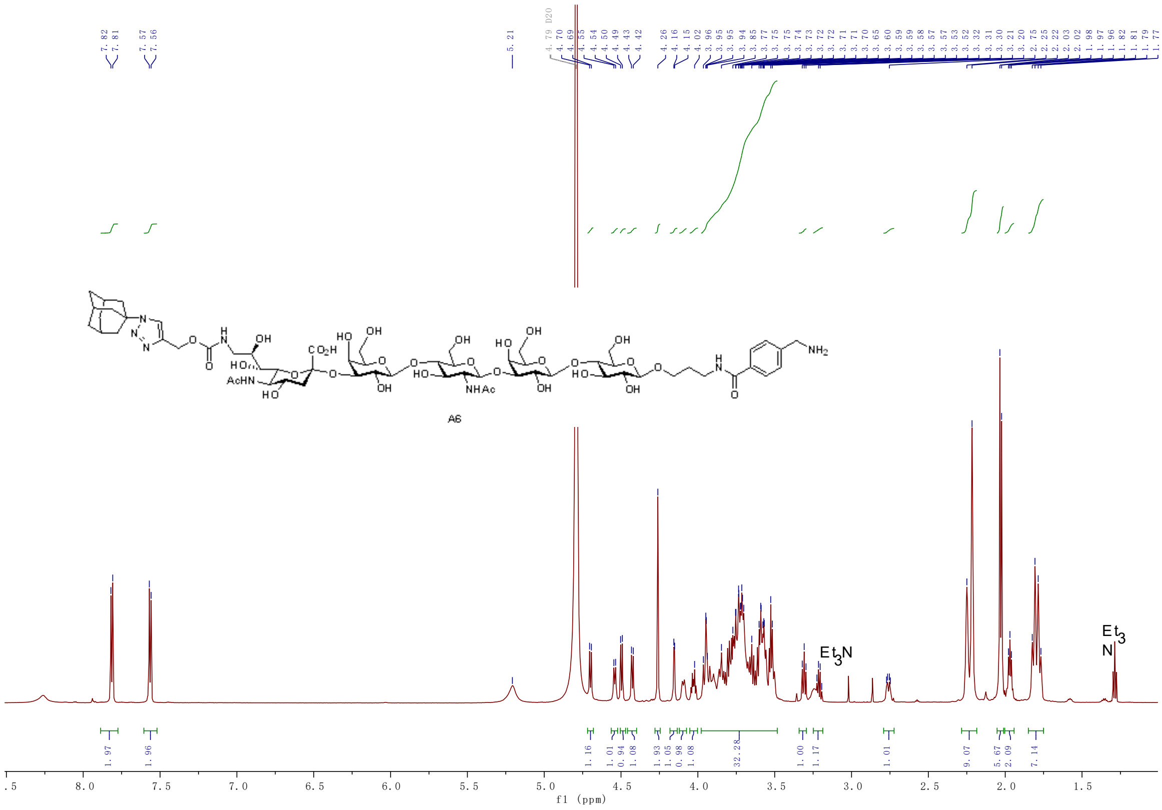

^{1}H NMR spectra of compound **A6**

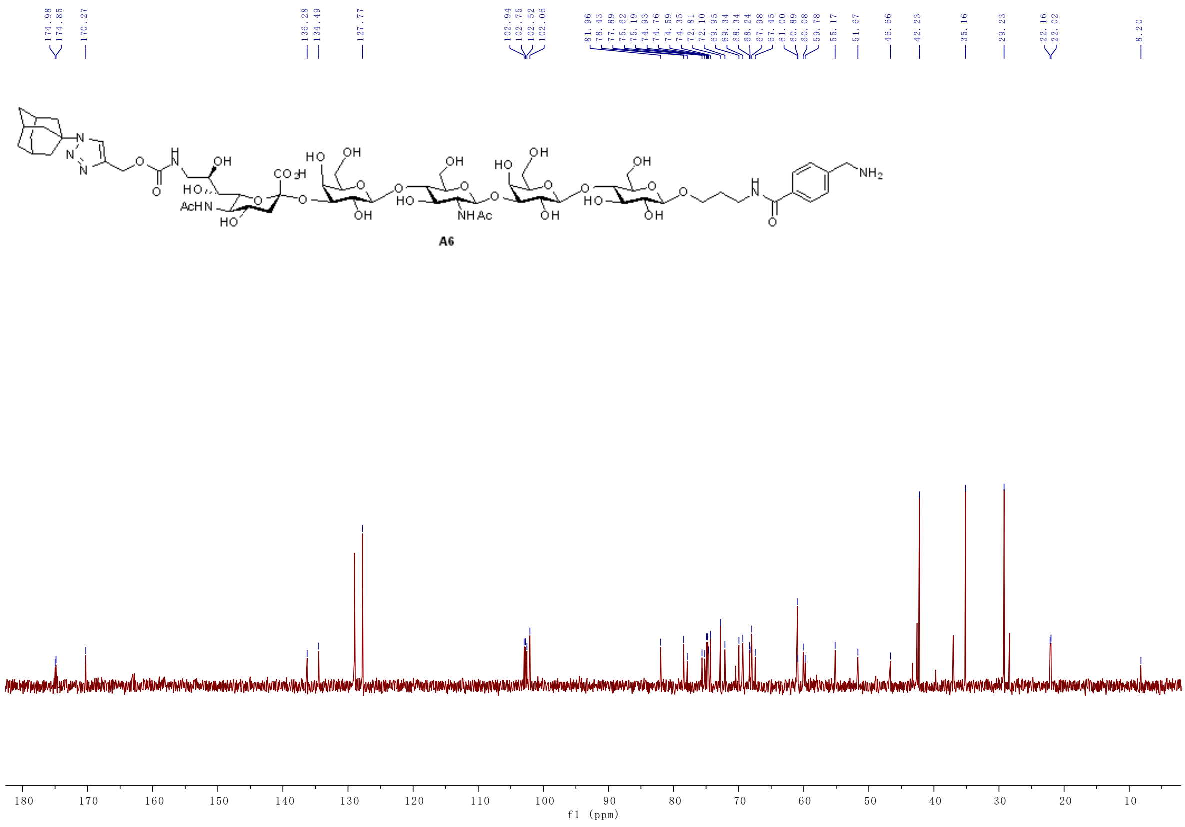

^{13}C NMR spectra of compound **A6**

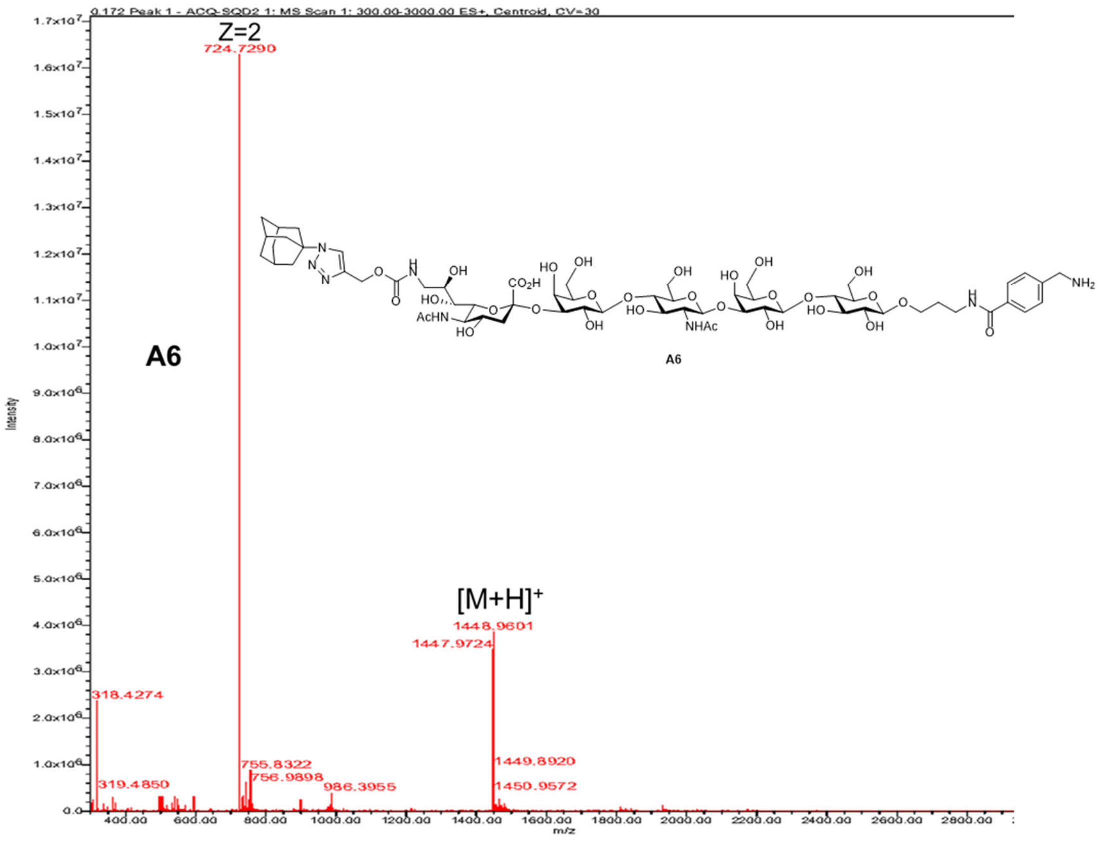

ESI-MS specta of compound **A6**

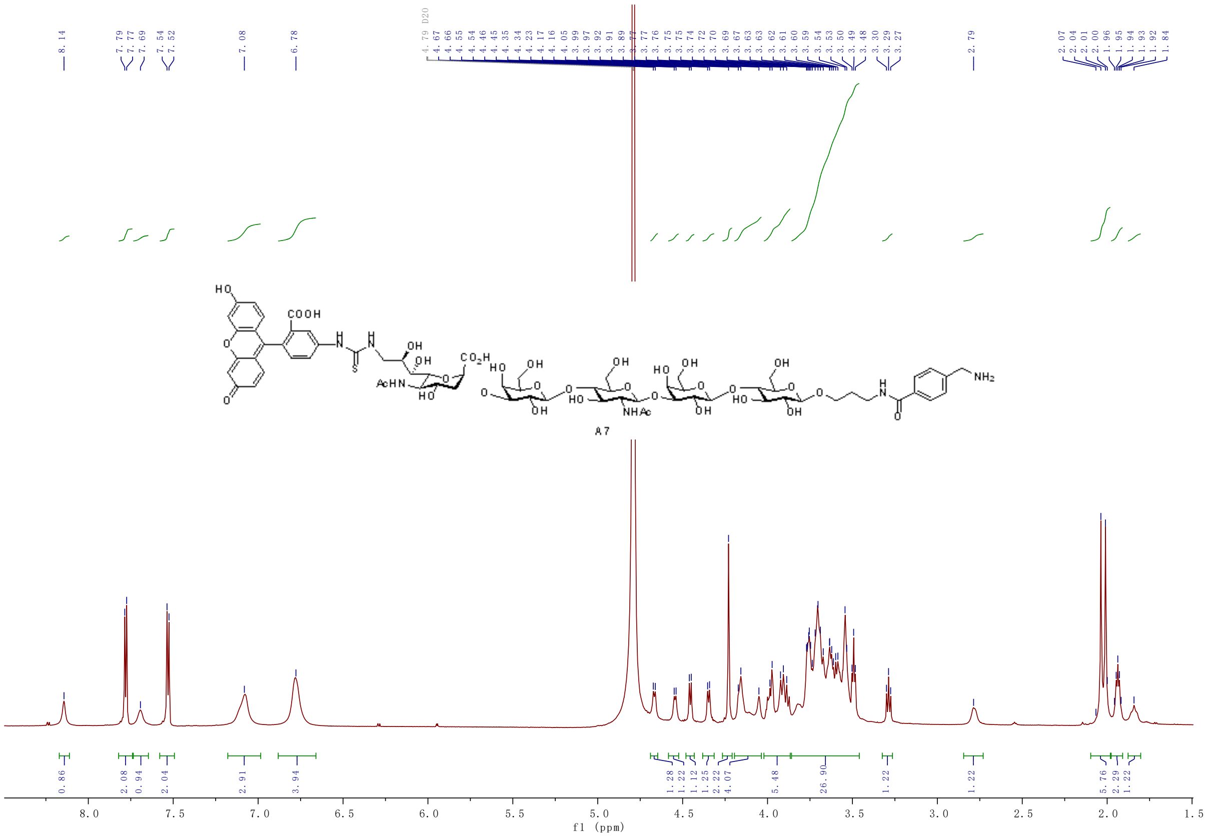

^{1}H NMR spectra of compound **A7**

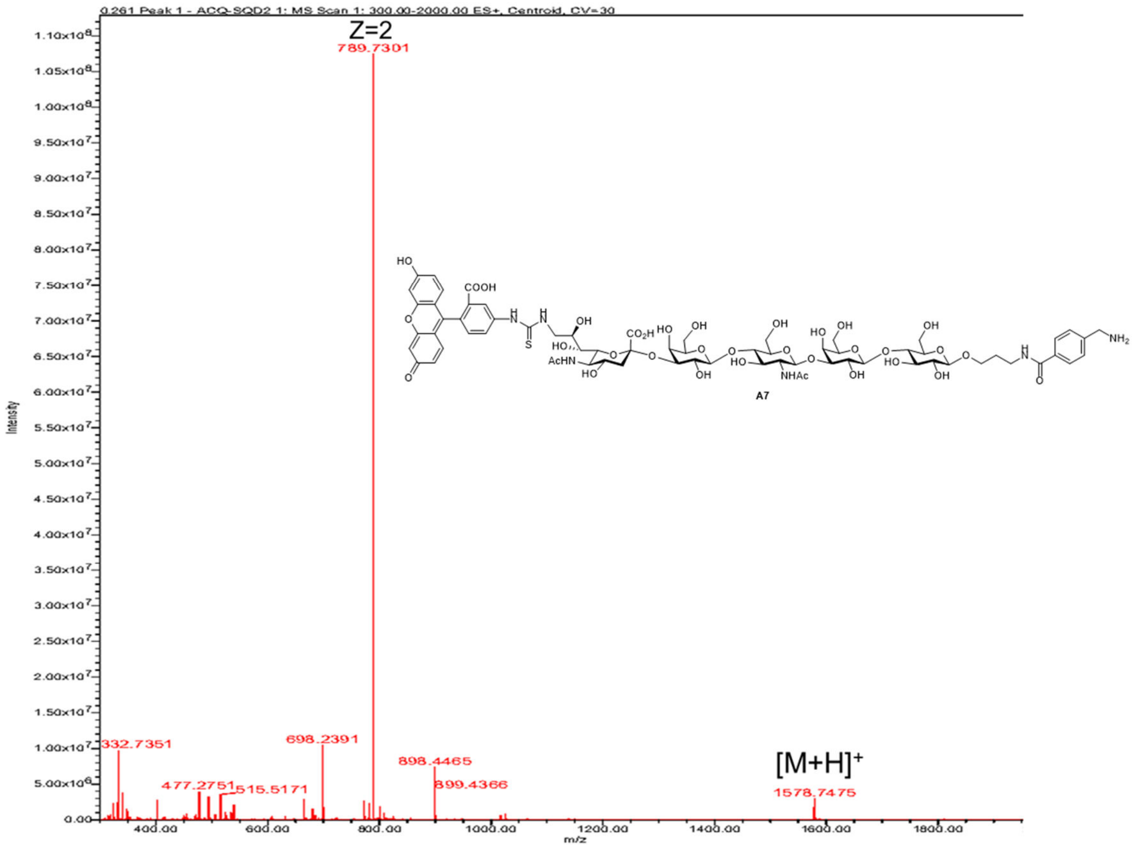

ESI-MS specta of compound **A7**

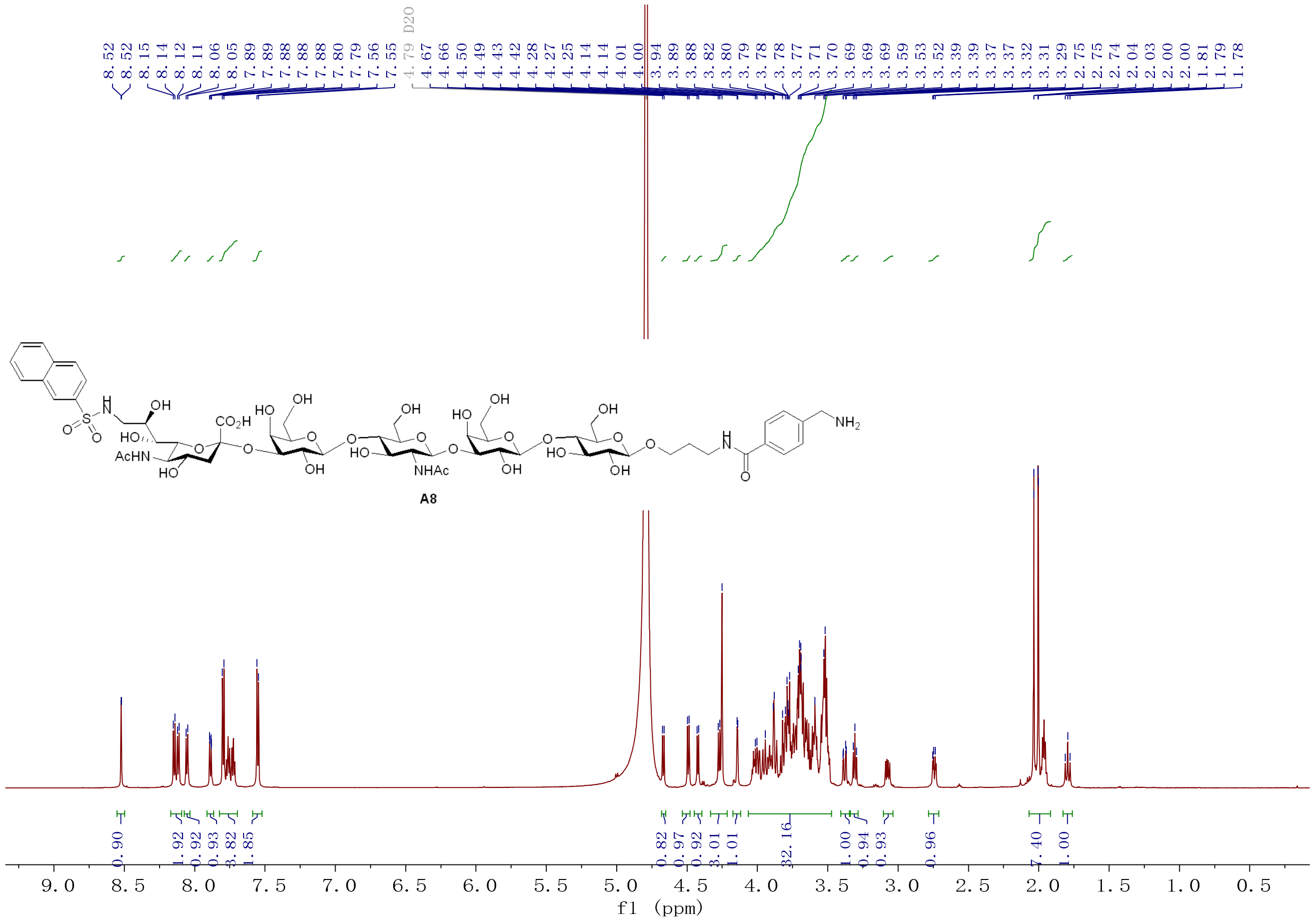

^{1}H NMR spectra of compound **A8**

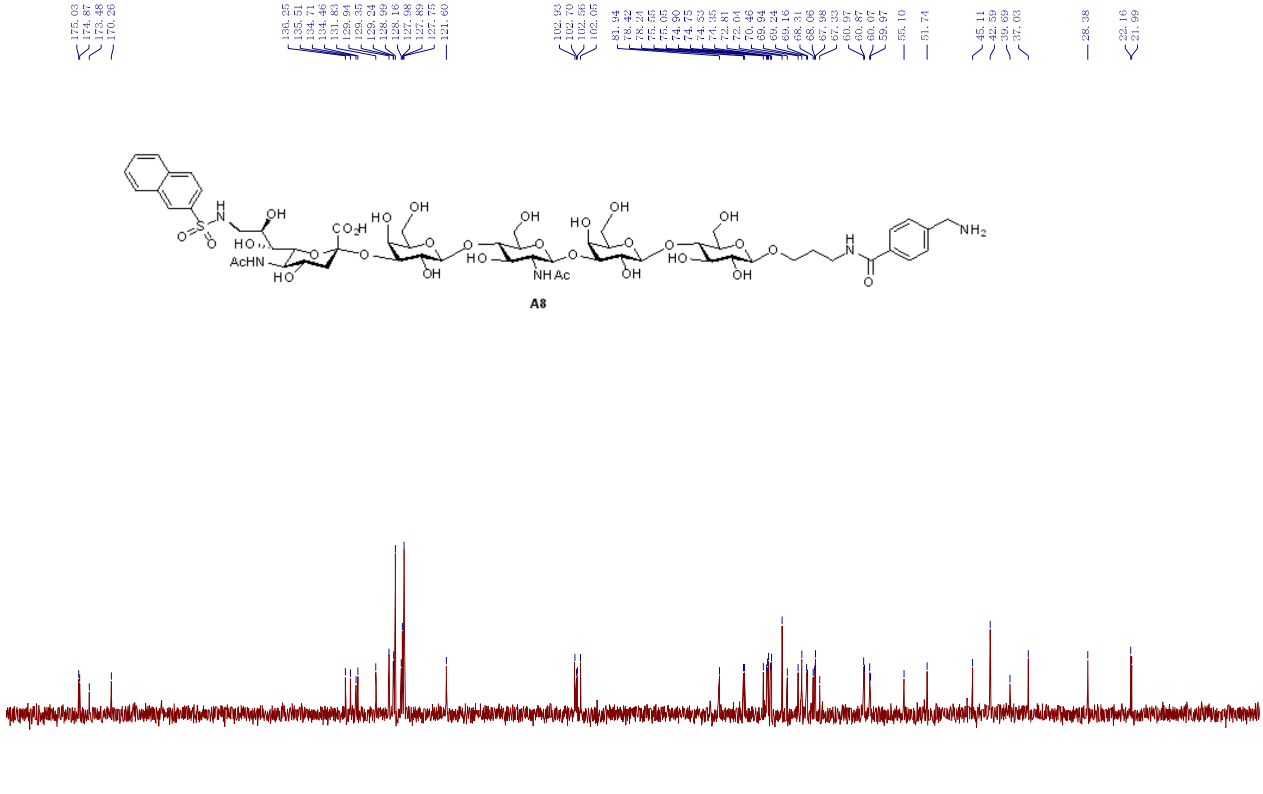

^{13}C NMR spectra of compound **A8**

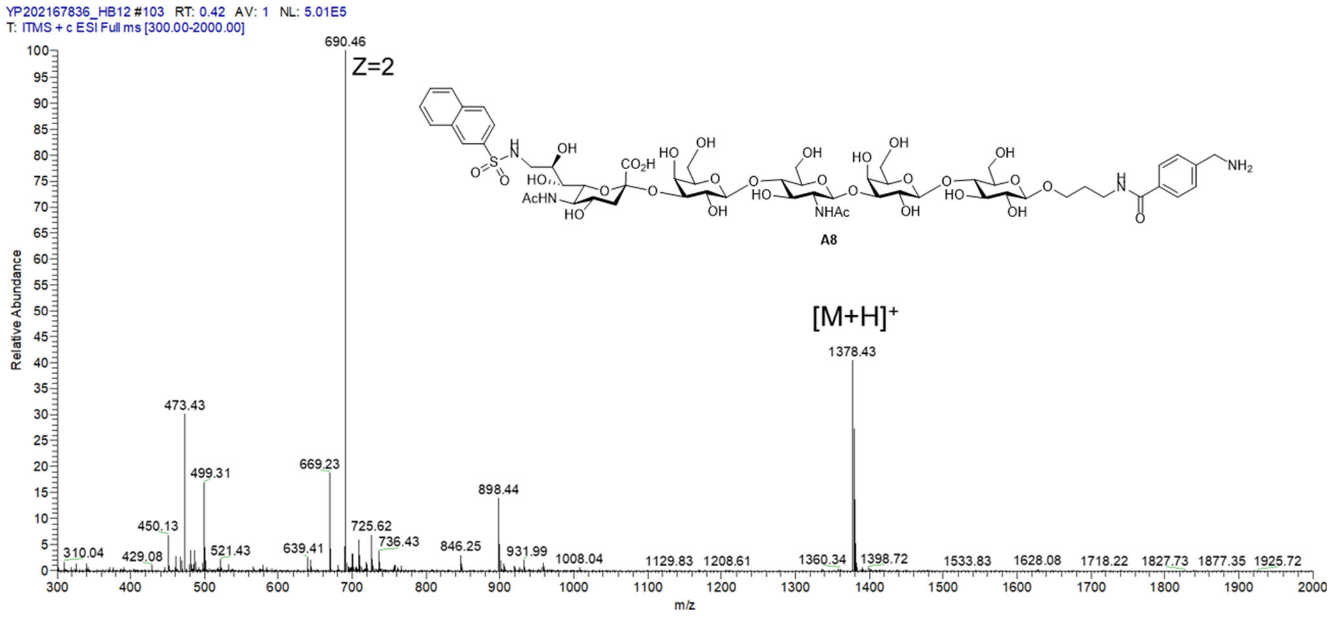

ESI-MS specta of compound **A8**

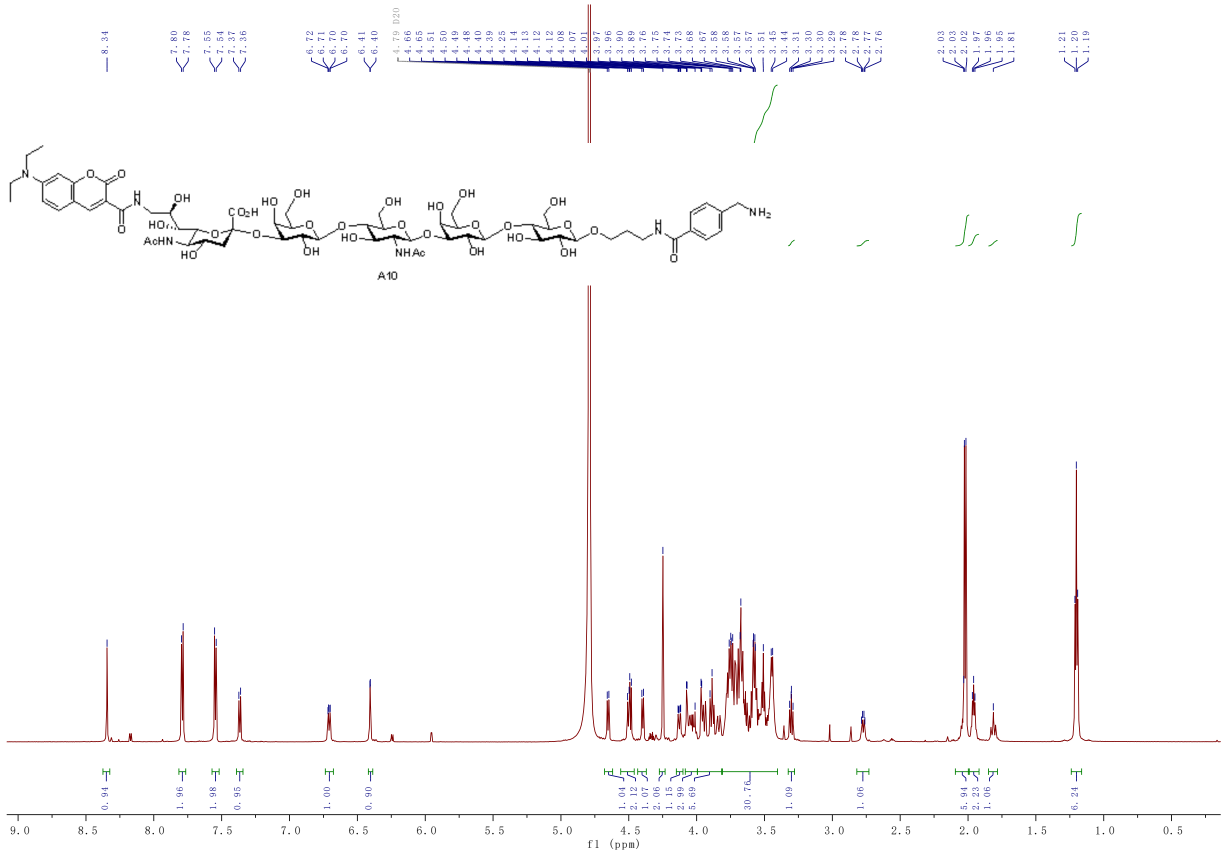

^{1}H NMR spectra of compound **A10**

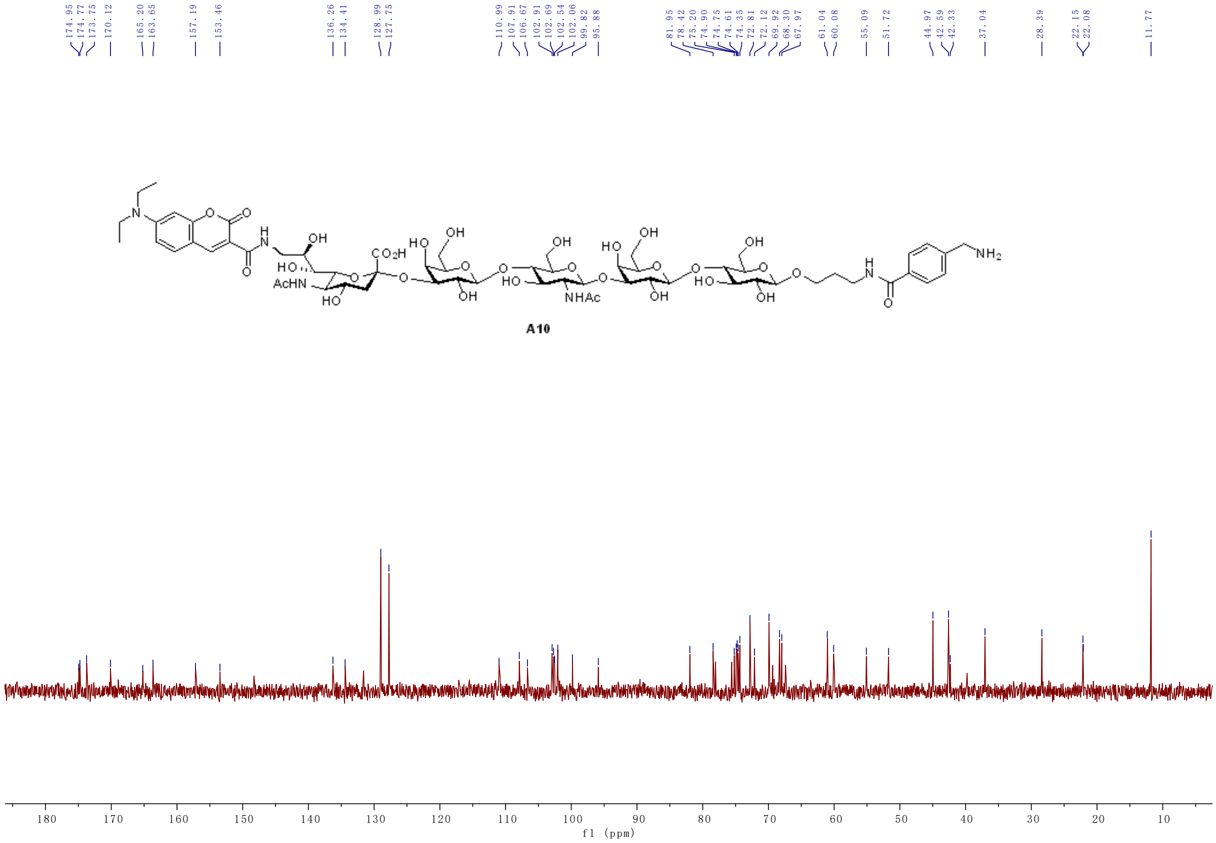

^{13}C NMR spectra of compound **A10**

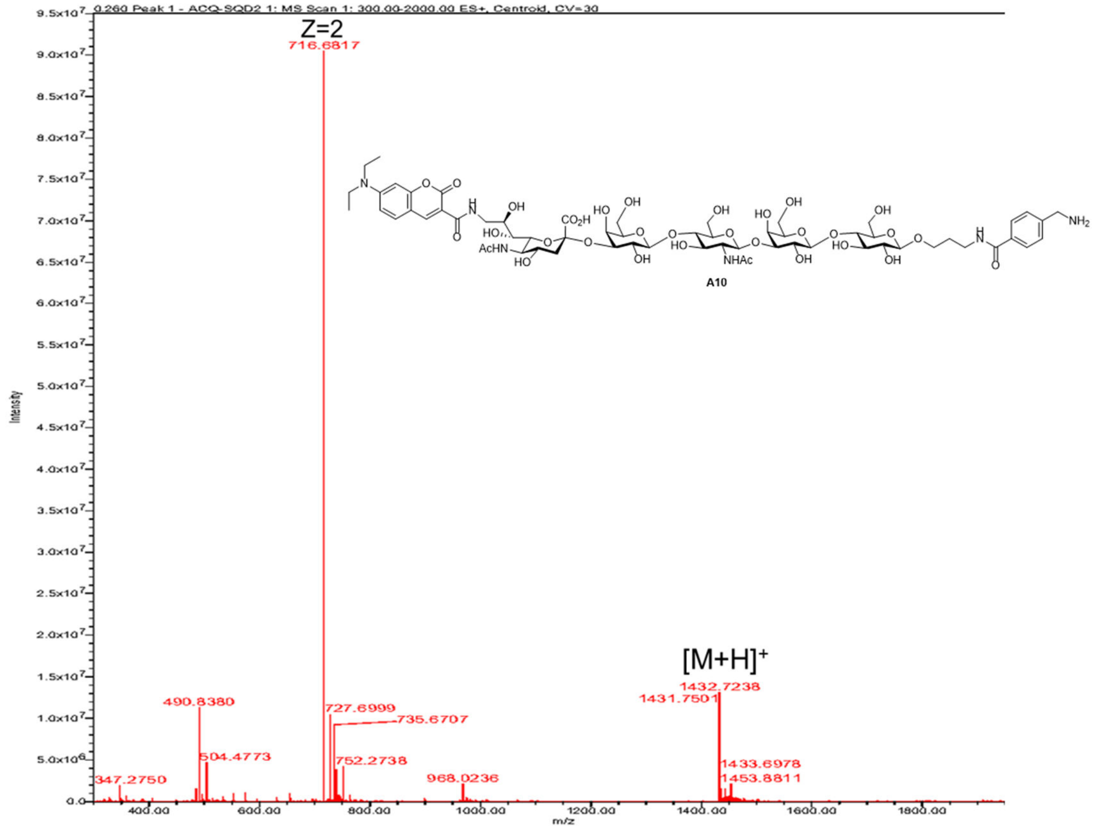

ESI-MS specta of compound **A10**

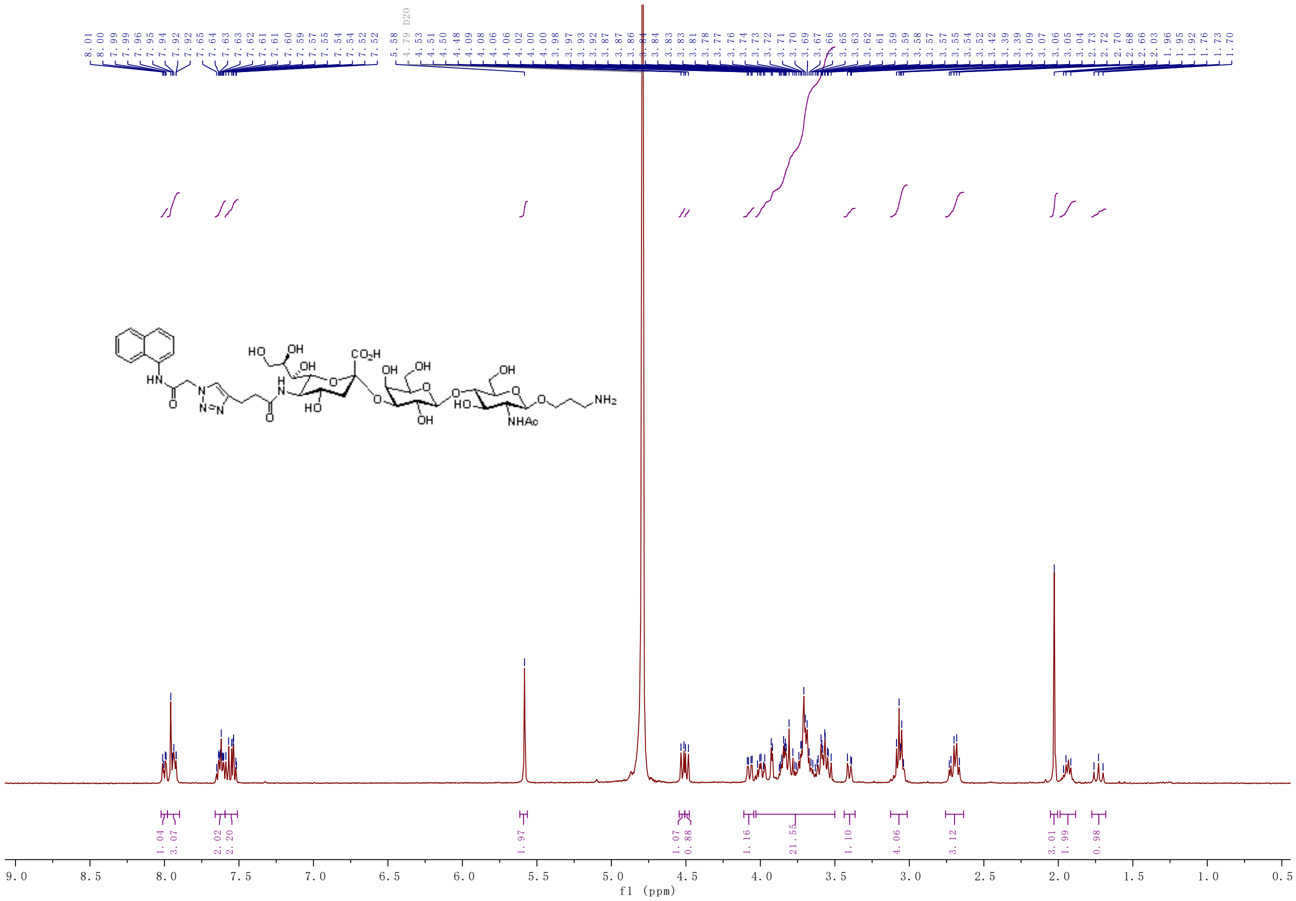

^{1}H NMR spectra of compound **A12**

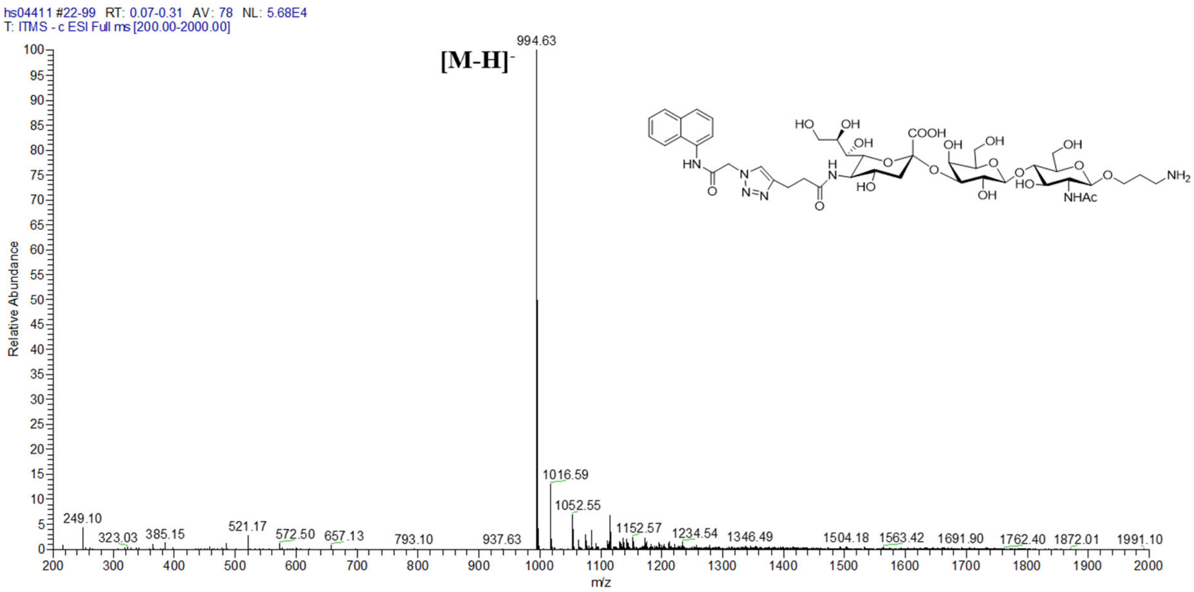

ESI-MS specta of compound **A12**

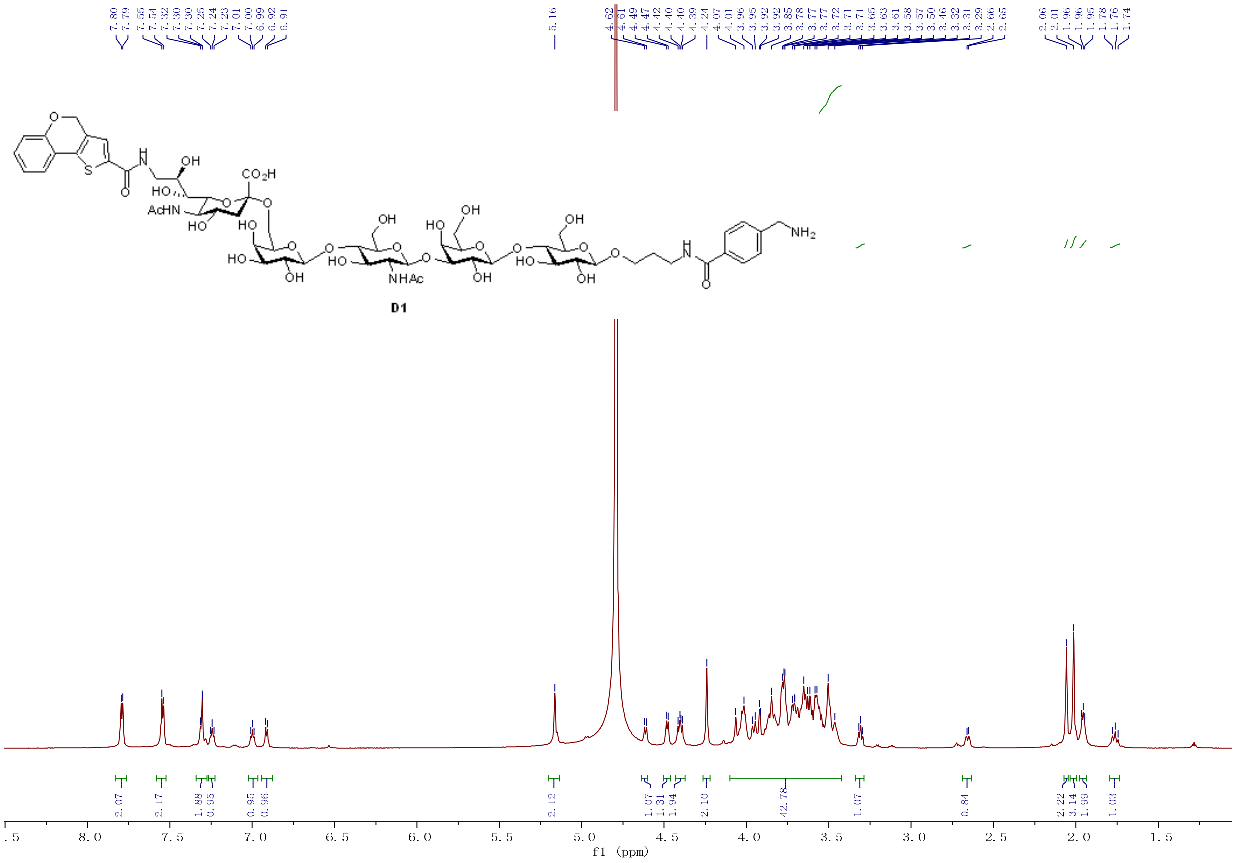

^{1}H NMR spectra of compound **D1**

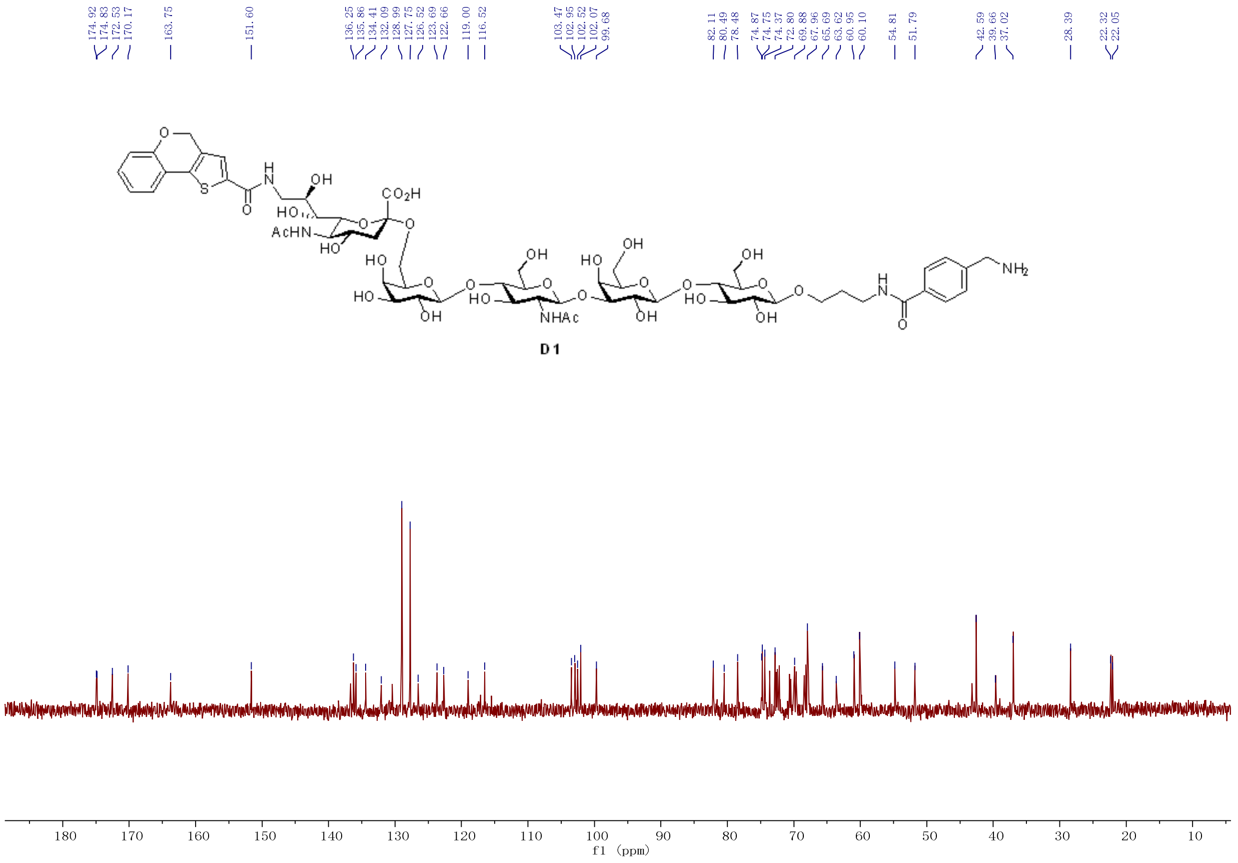

^{13}C NMR spectra of compound **D1**

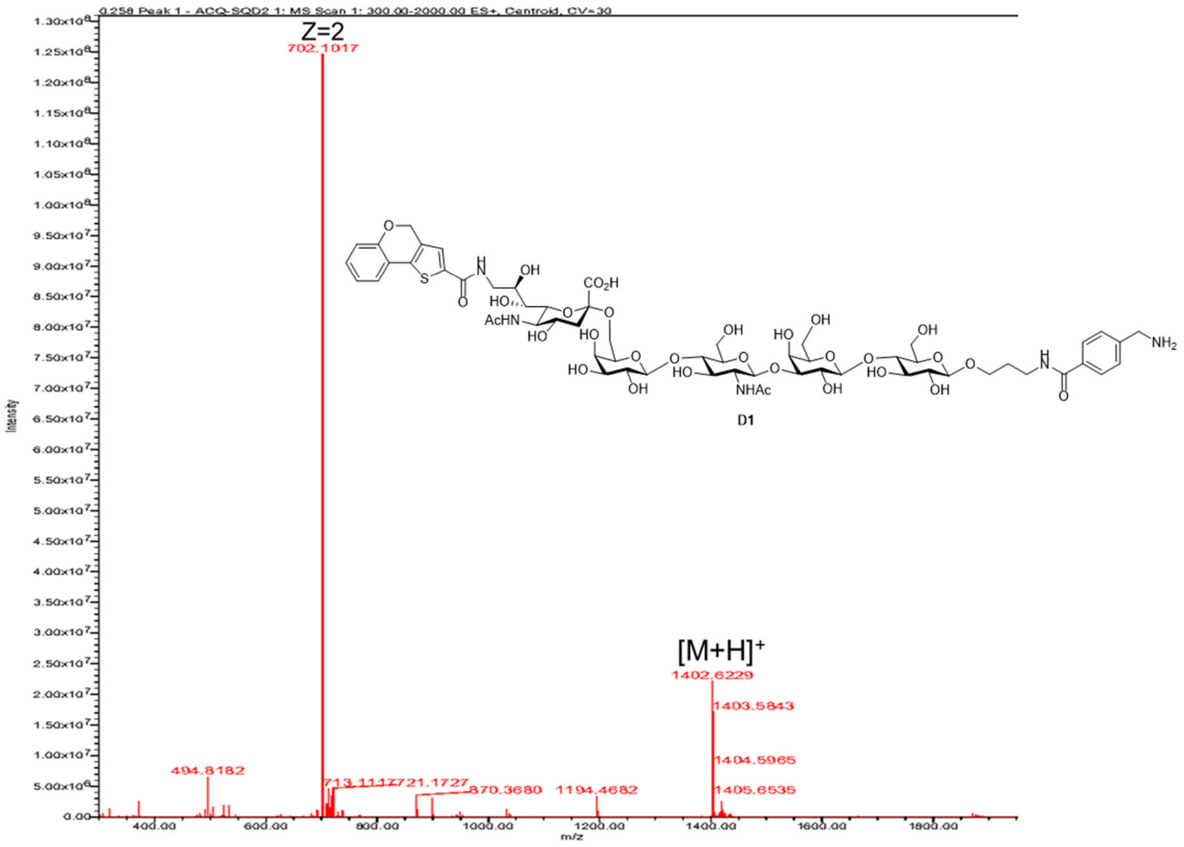

ESI-MS specta of compound **D1**

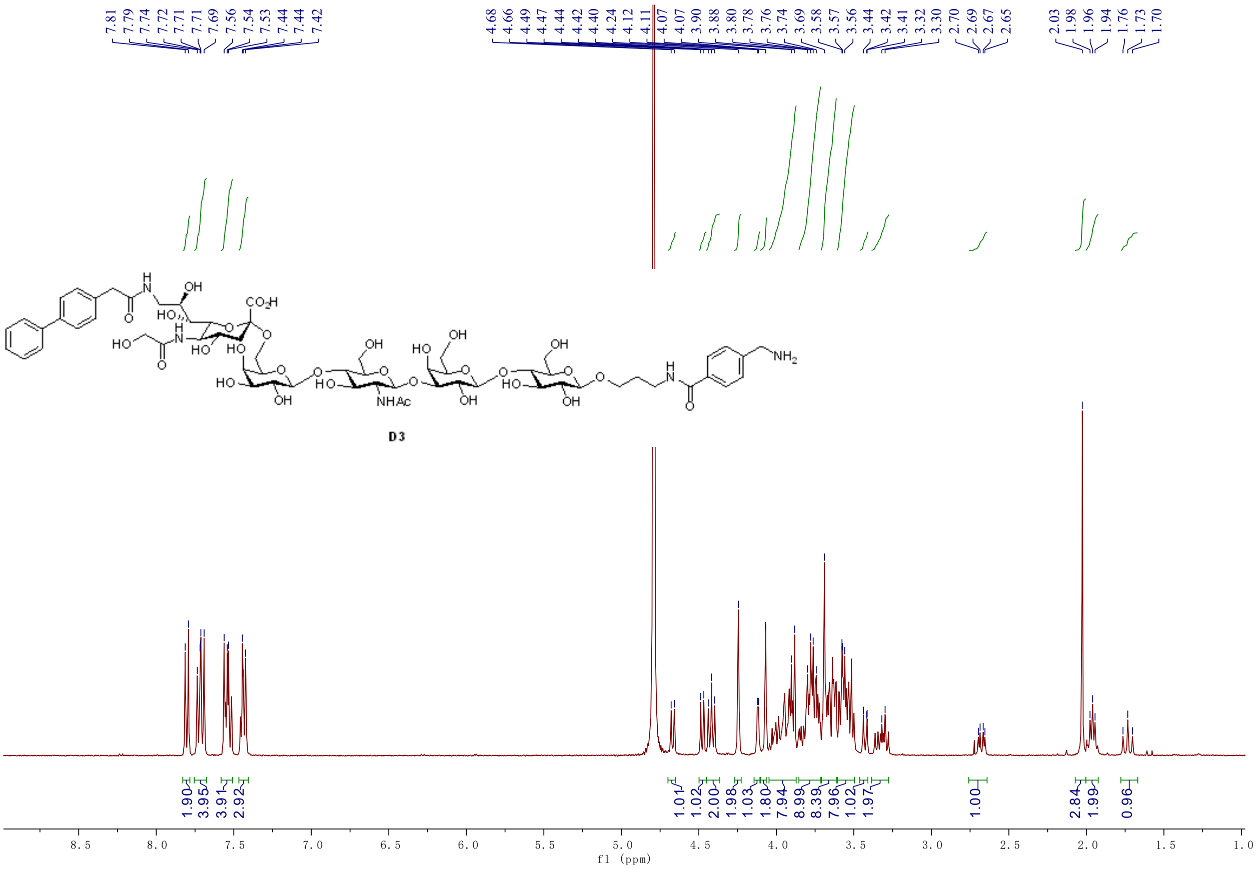

^{1}H NMR spectra of compound **D3**

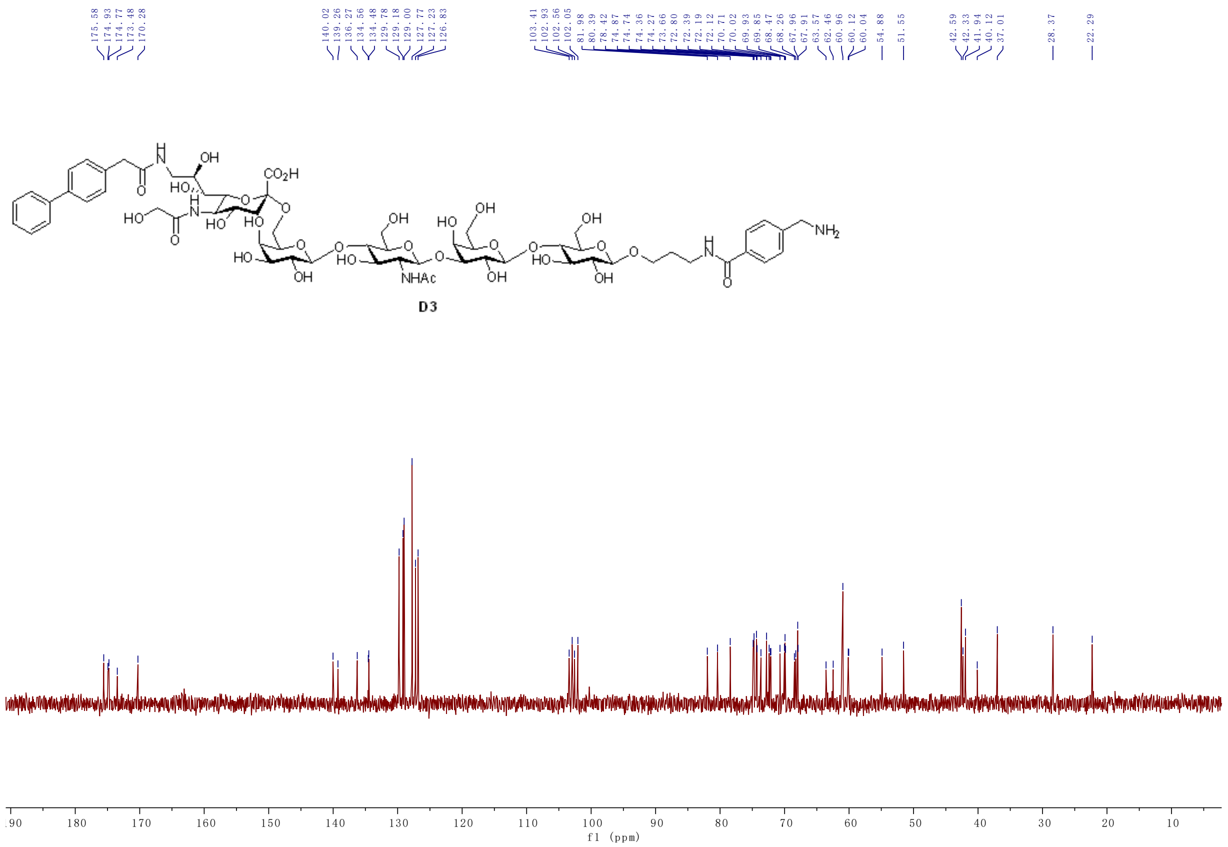

^{13}C NMR spectra of compound **D3**

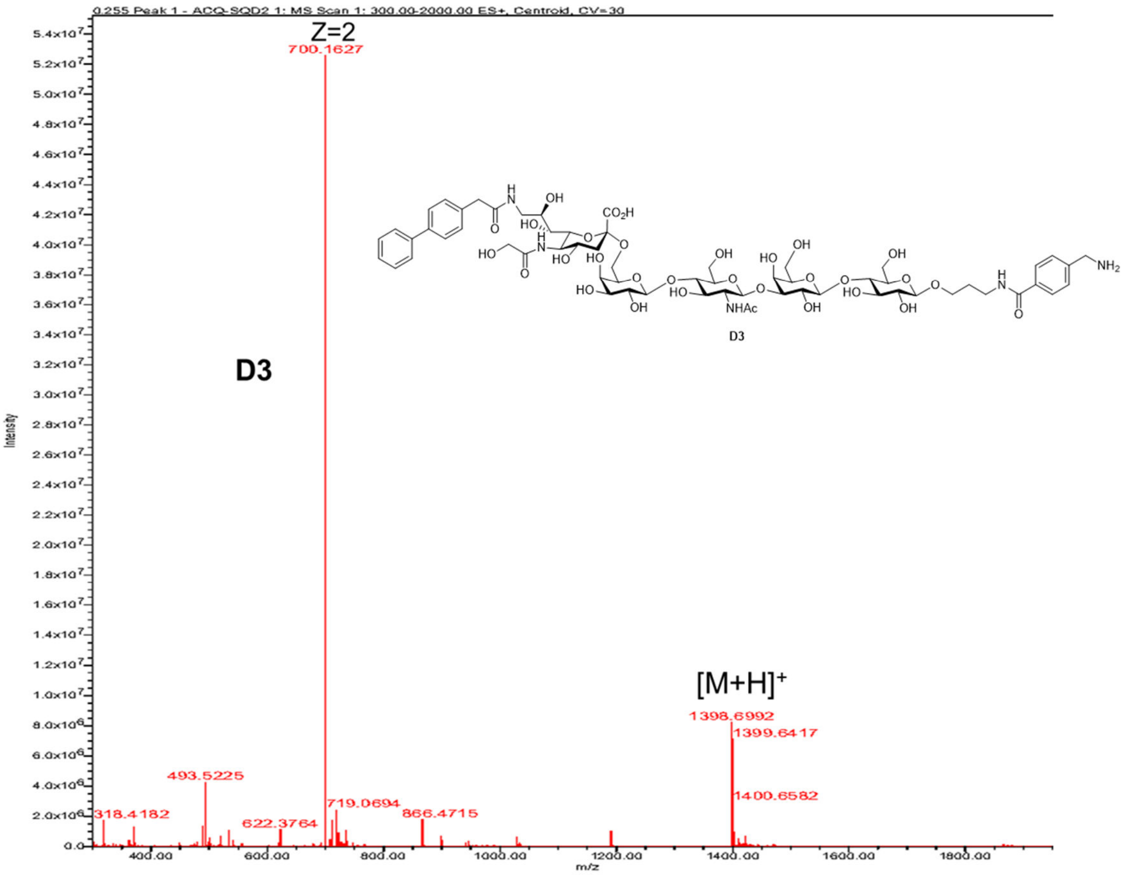

ESI-MS specta of compound **D3**

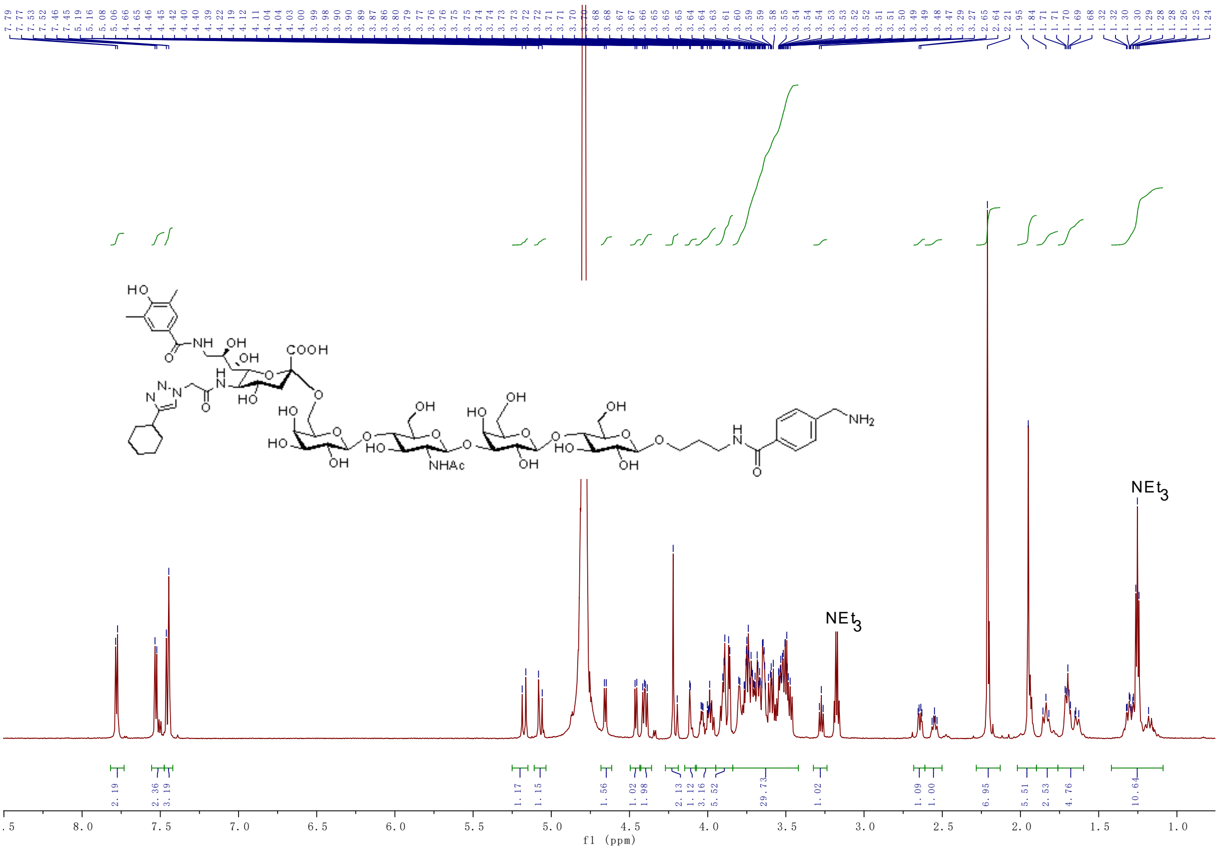

^{1}H NMR spectra of compound **D4**

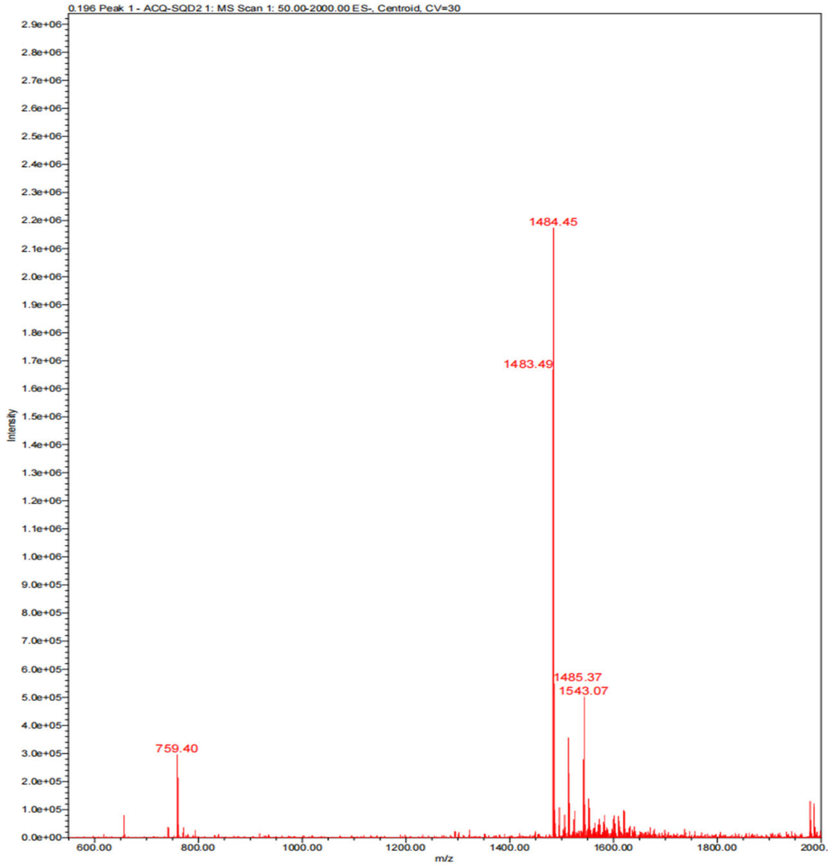

ESI-MS specta of compound **D4**

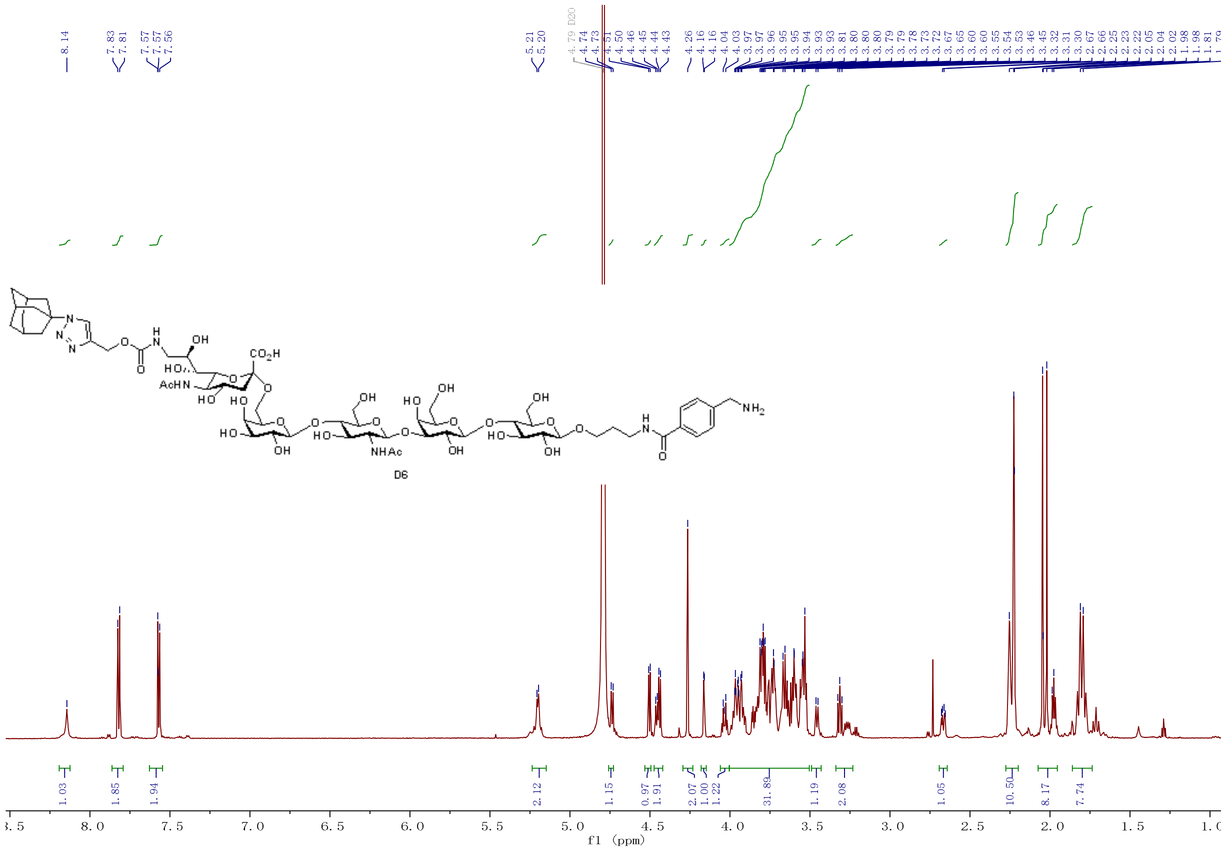

^{1}H NMR spectra of compound **D6**

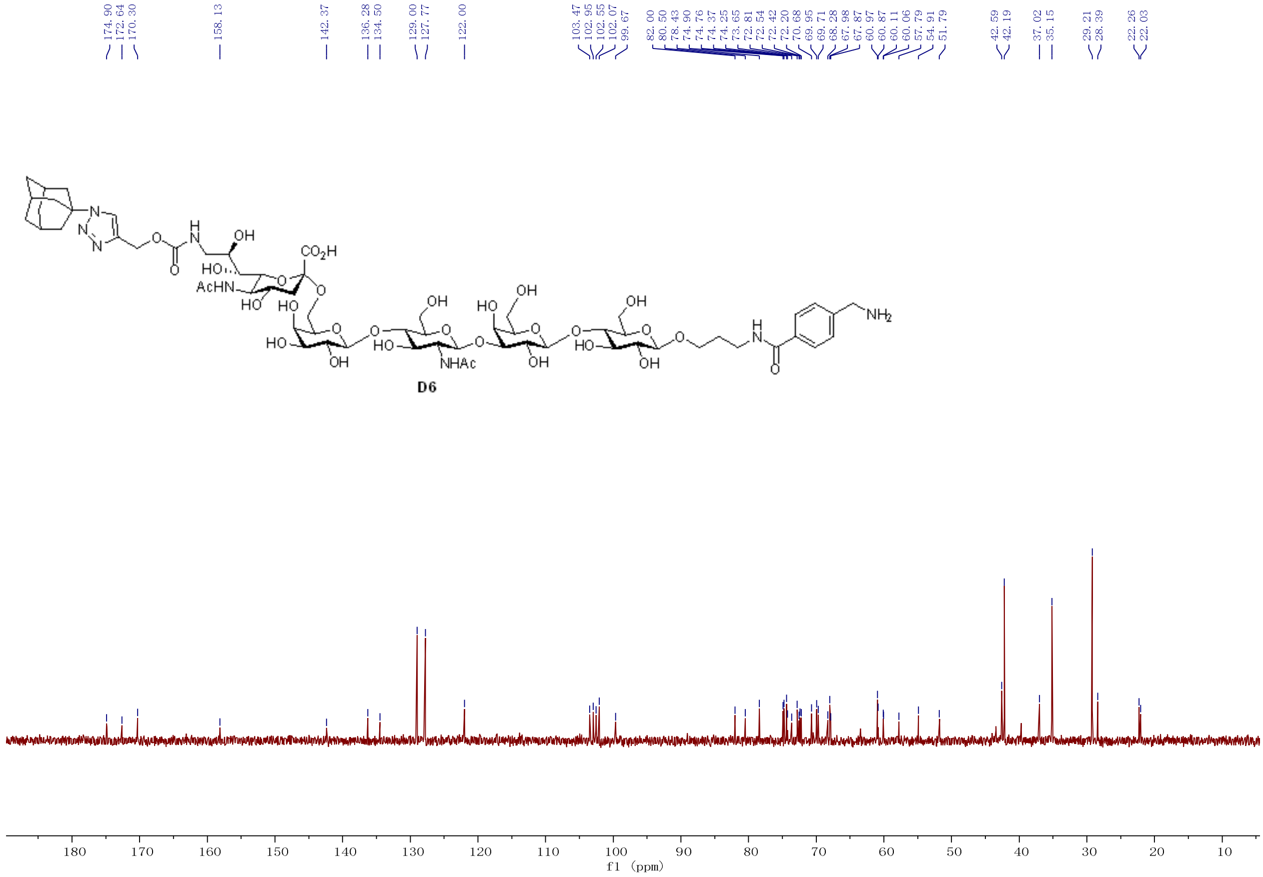

^{13}C NMR spectra of compound **D6**

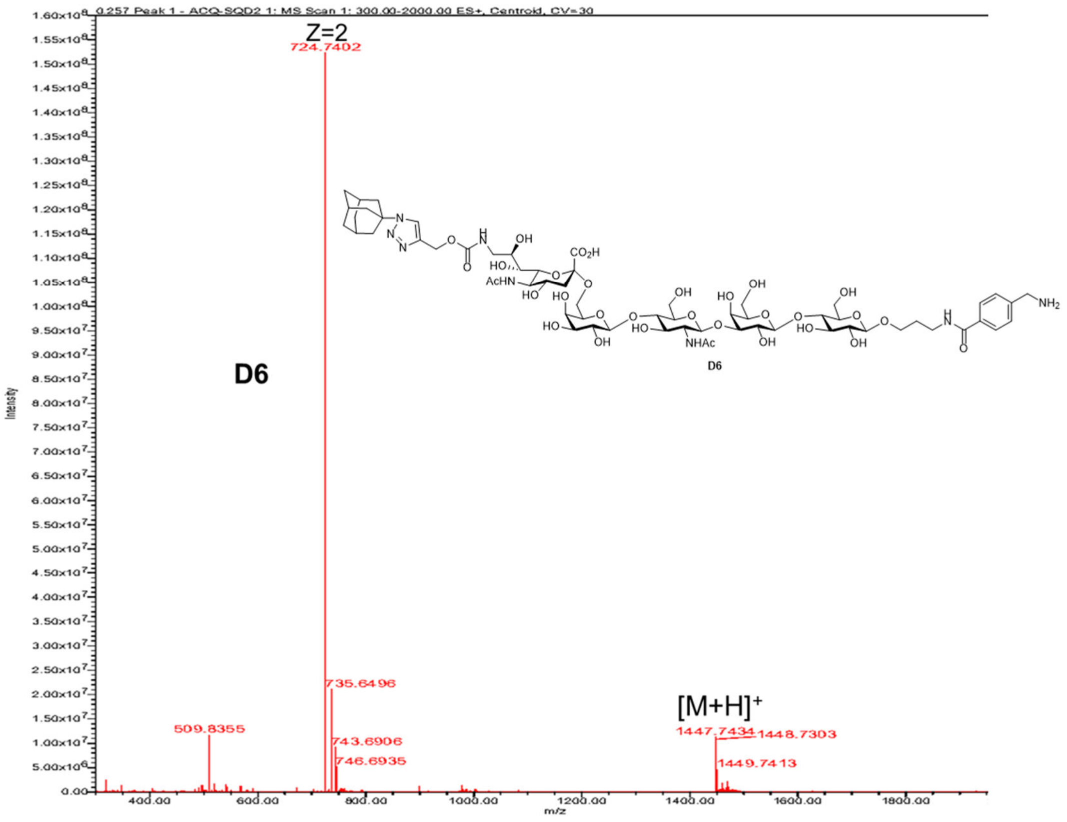

ESI-MS specta of compound **D6**

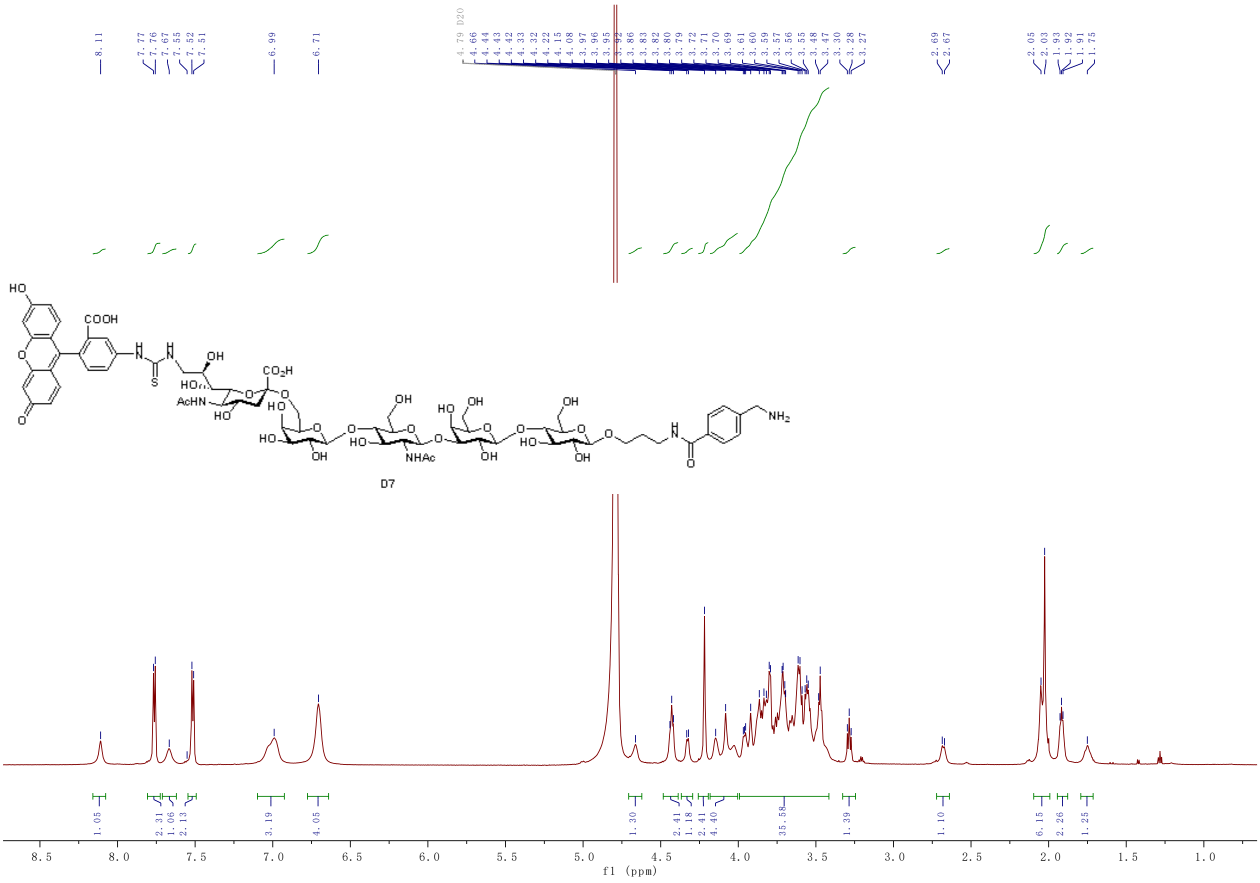

^{1}H NMR spectra of compound **D7**

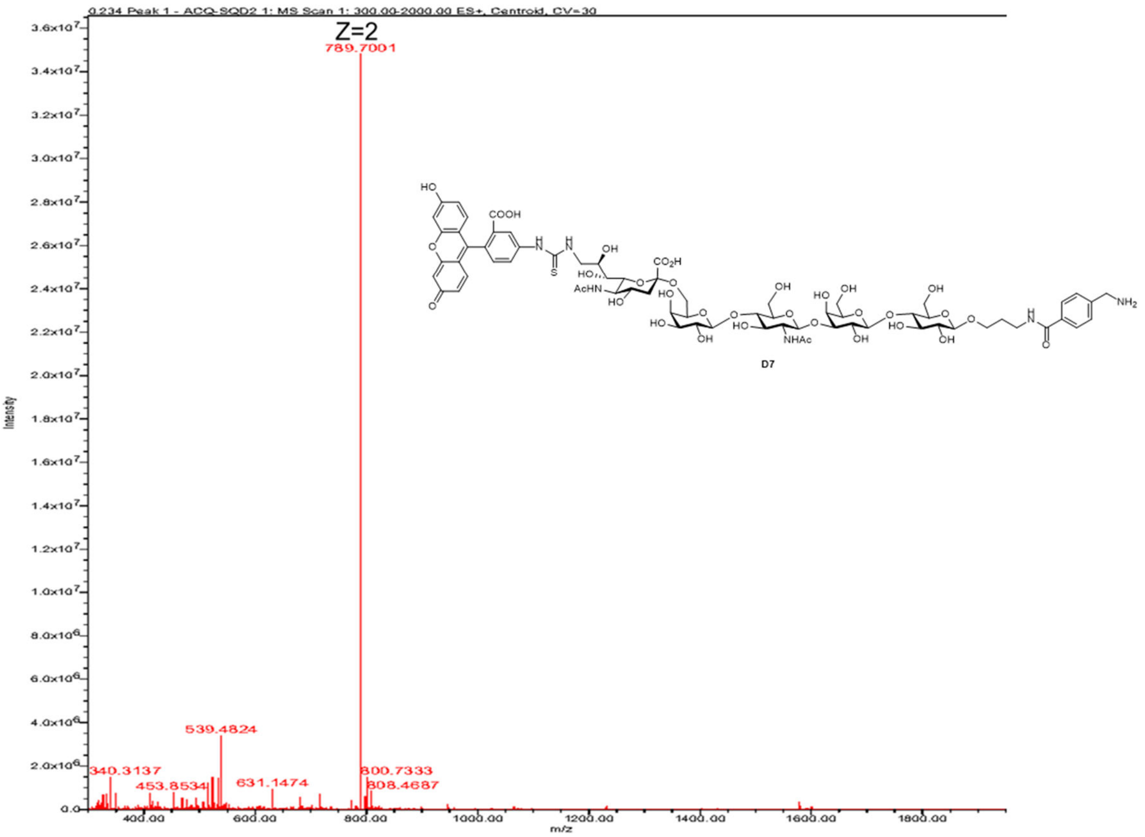

ESI-MS specta of compound **D7**

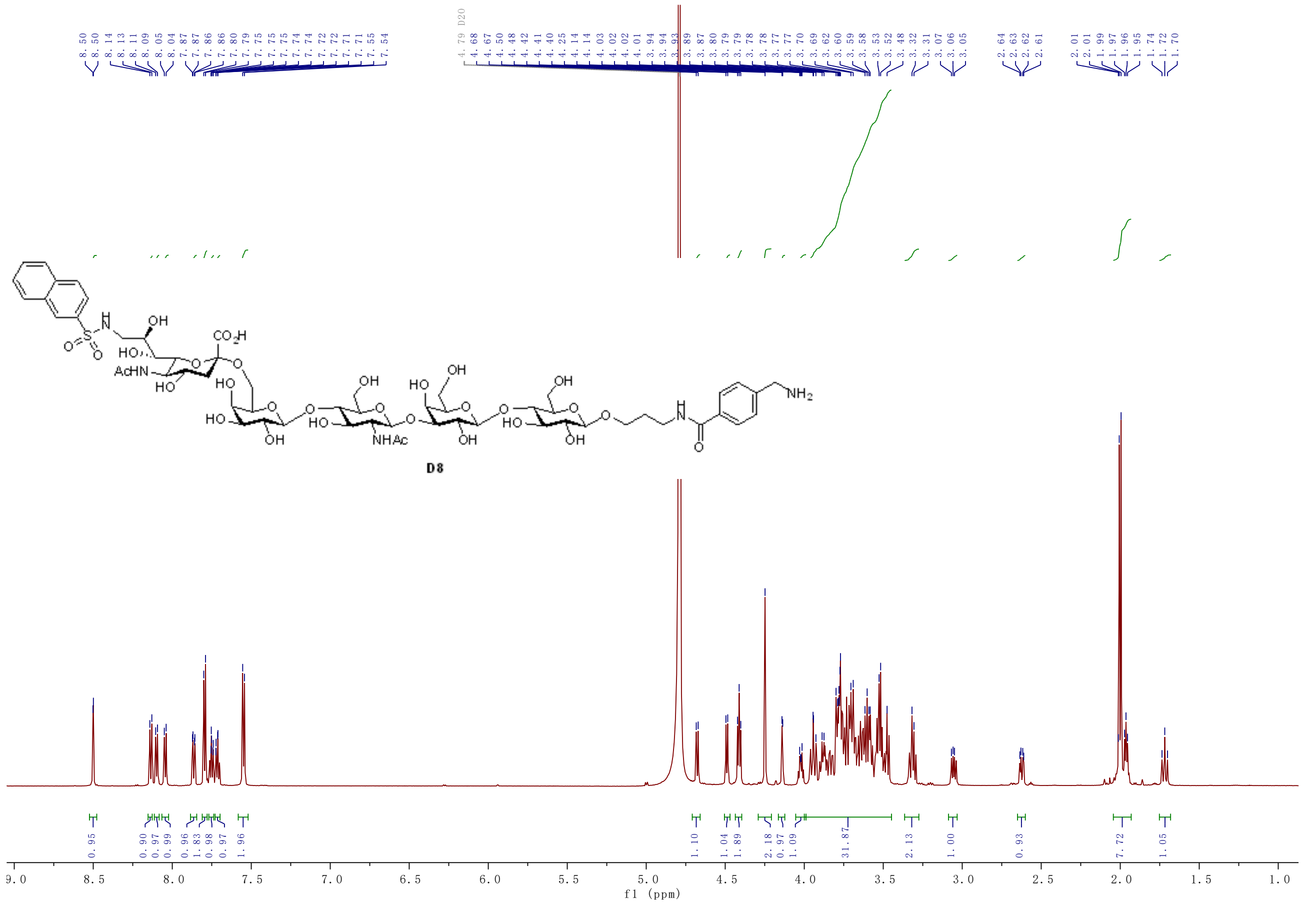

^{1}H NMR spectra of compound **D8**

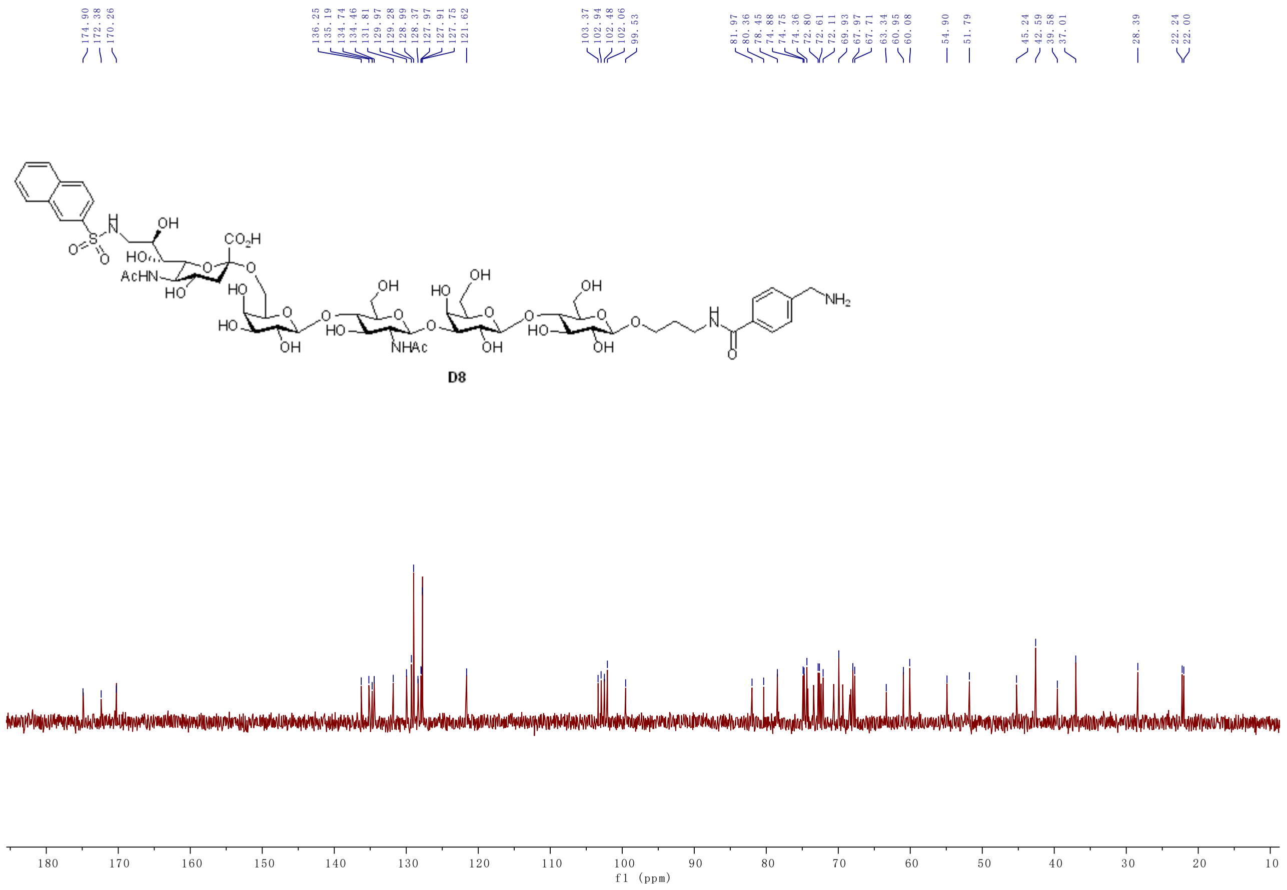

^{13}C NMR spectra of compound **D8**

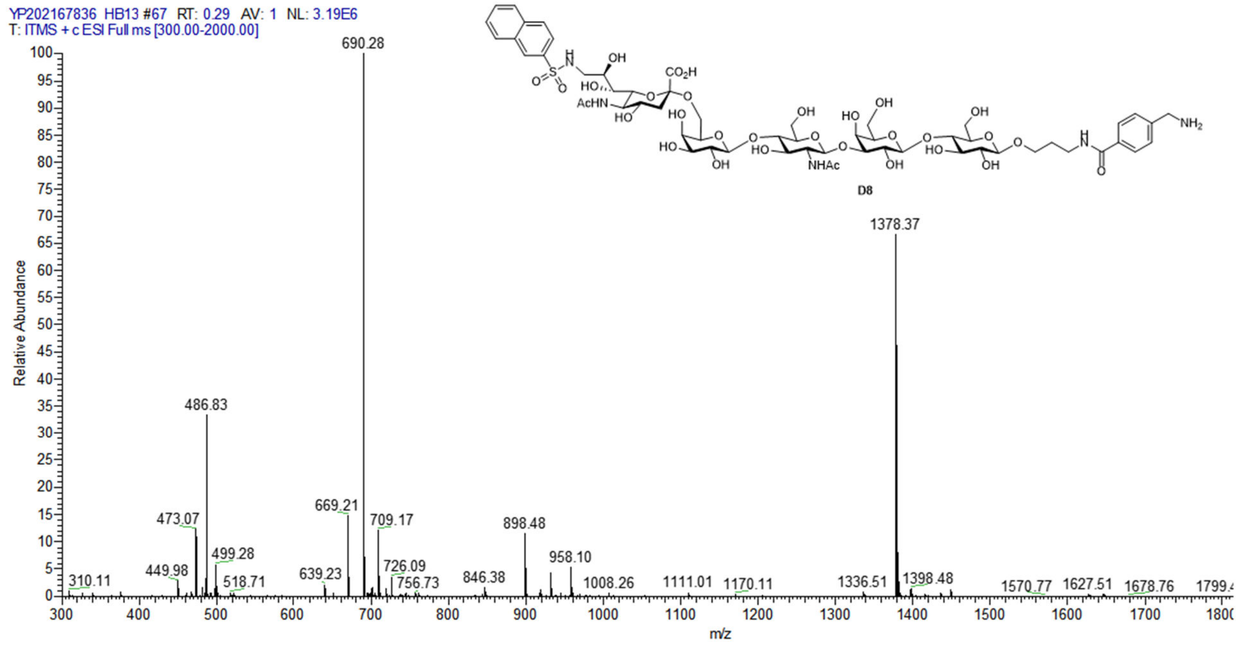

ESI-MS specta of compound **D8**

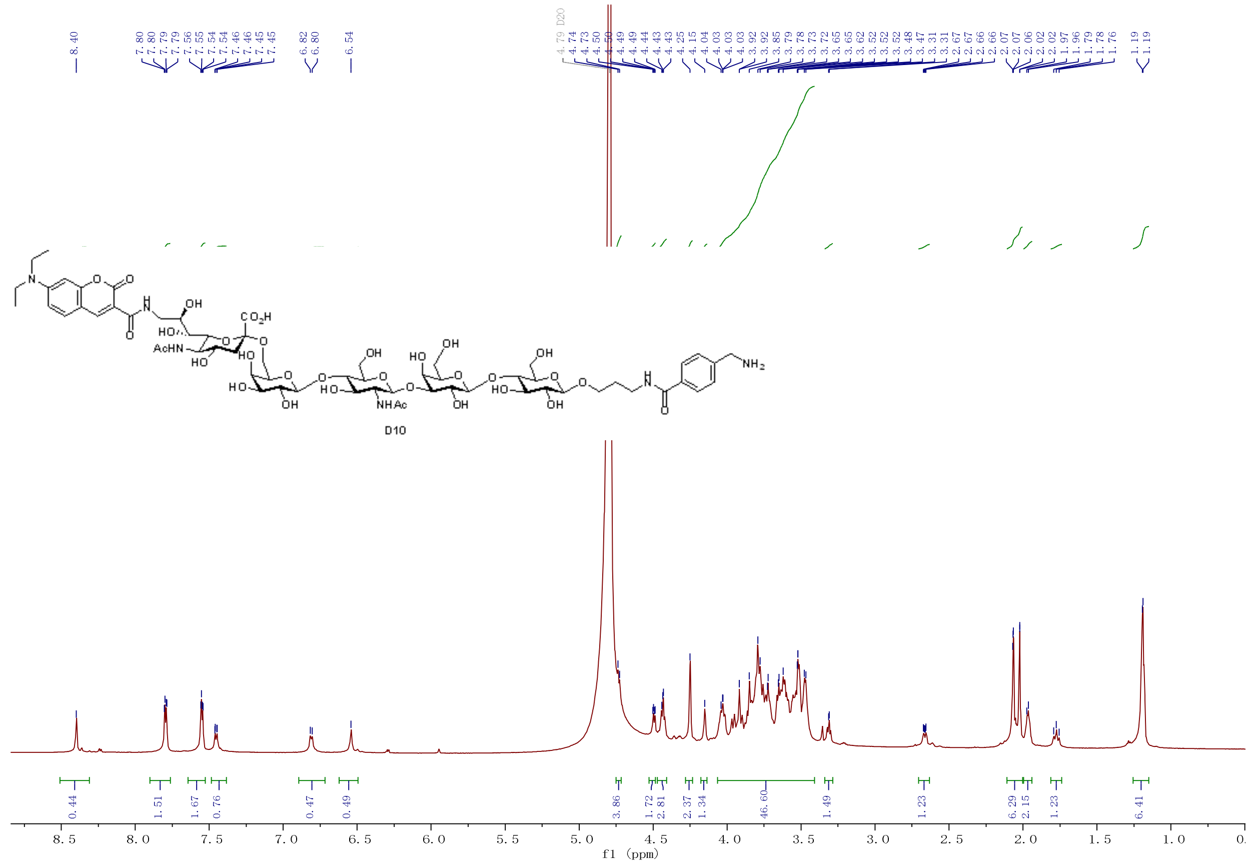

^{1}H NMR spectra of compound **D10**

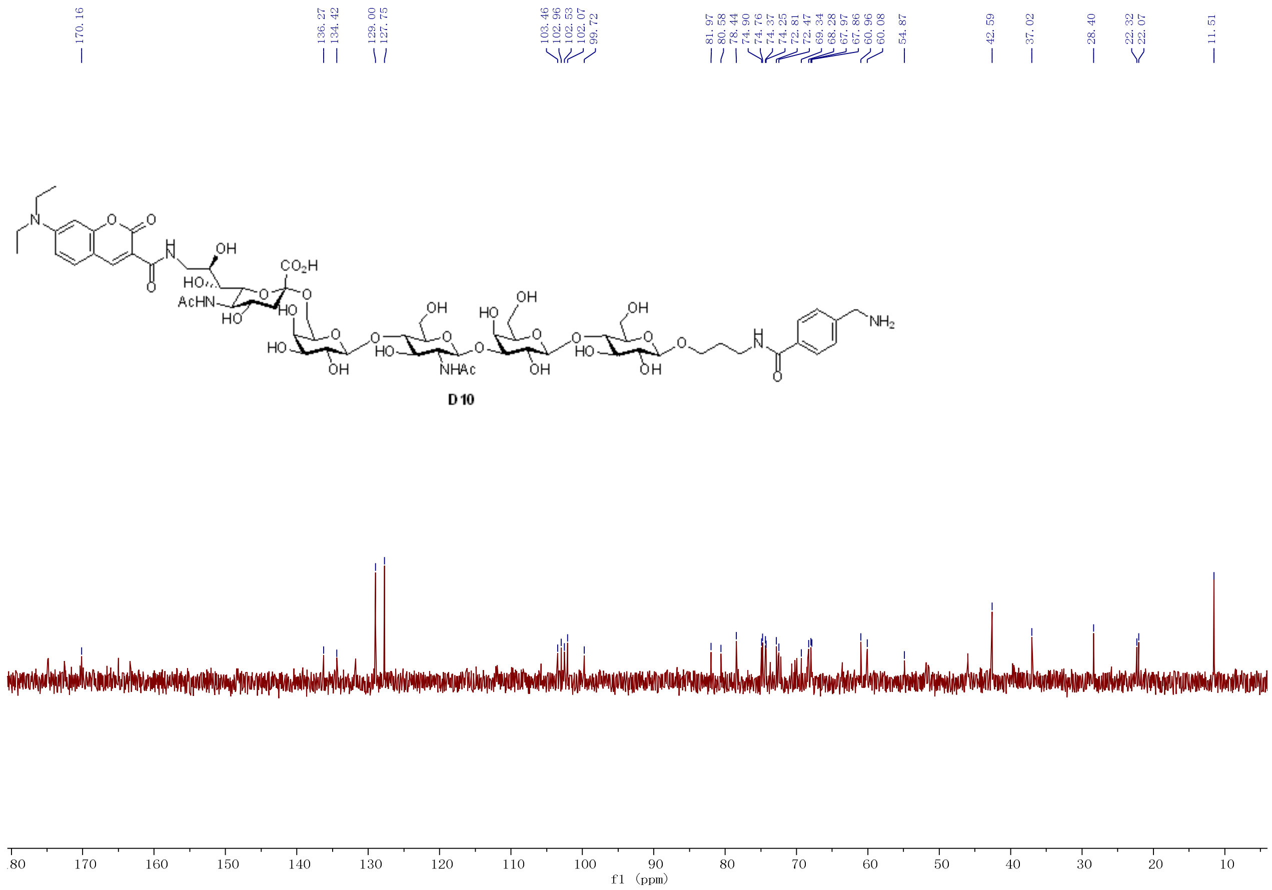

^{13}C NMR spectra of compound **D10**

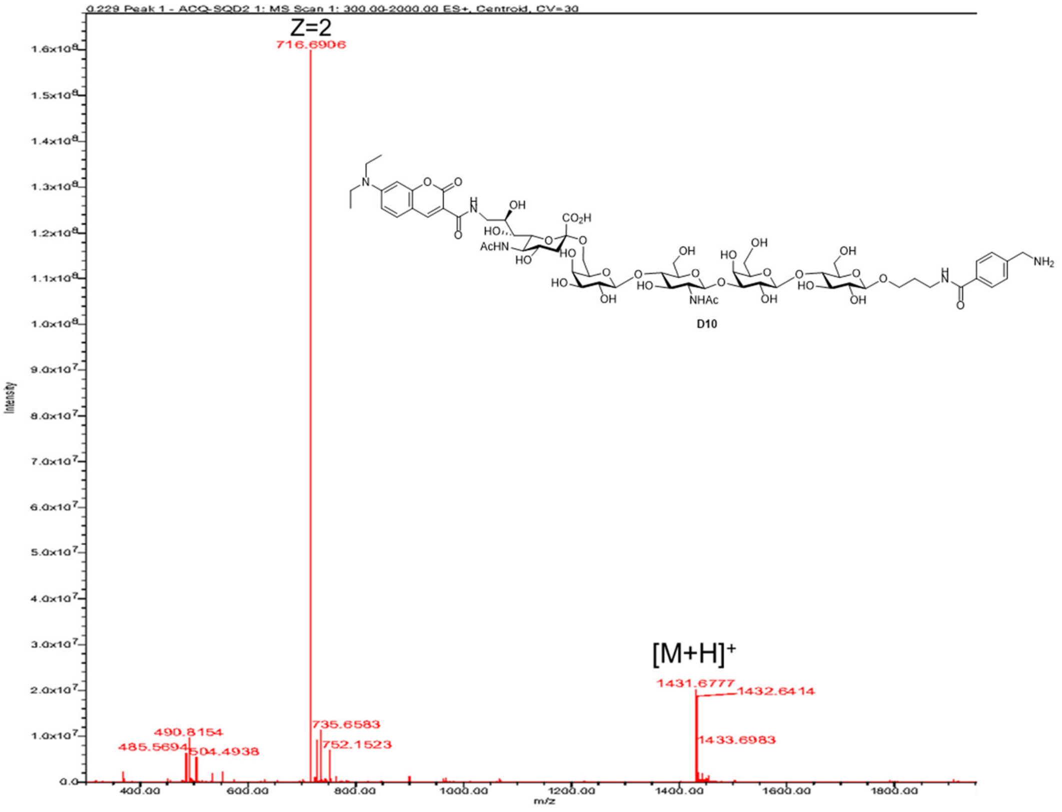

ESI-MS specta of compound **D10**

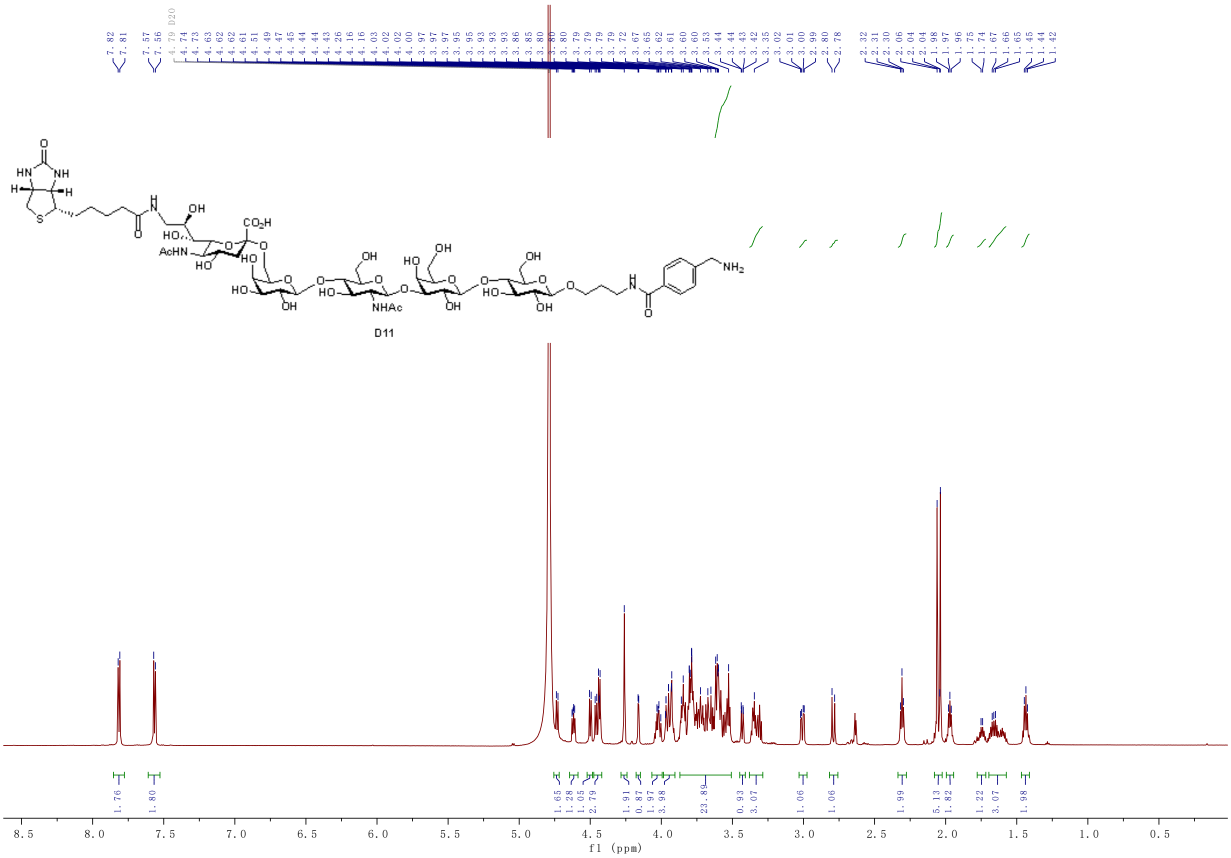

^{1}H NMR spectra of compound **D11**

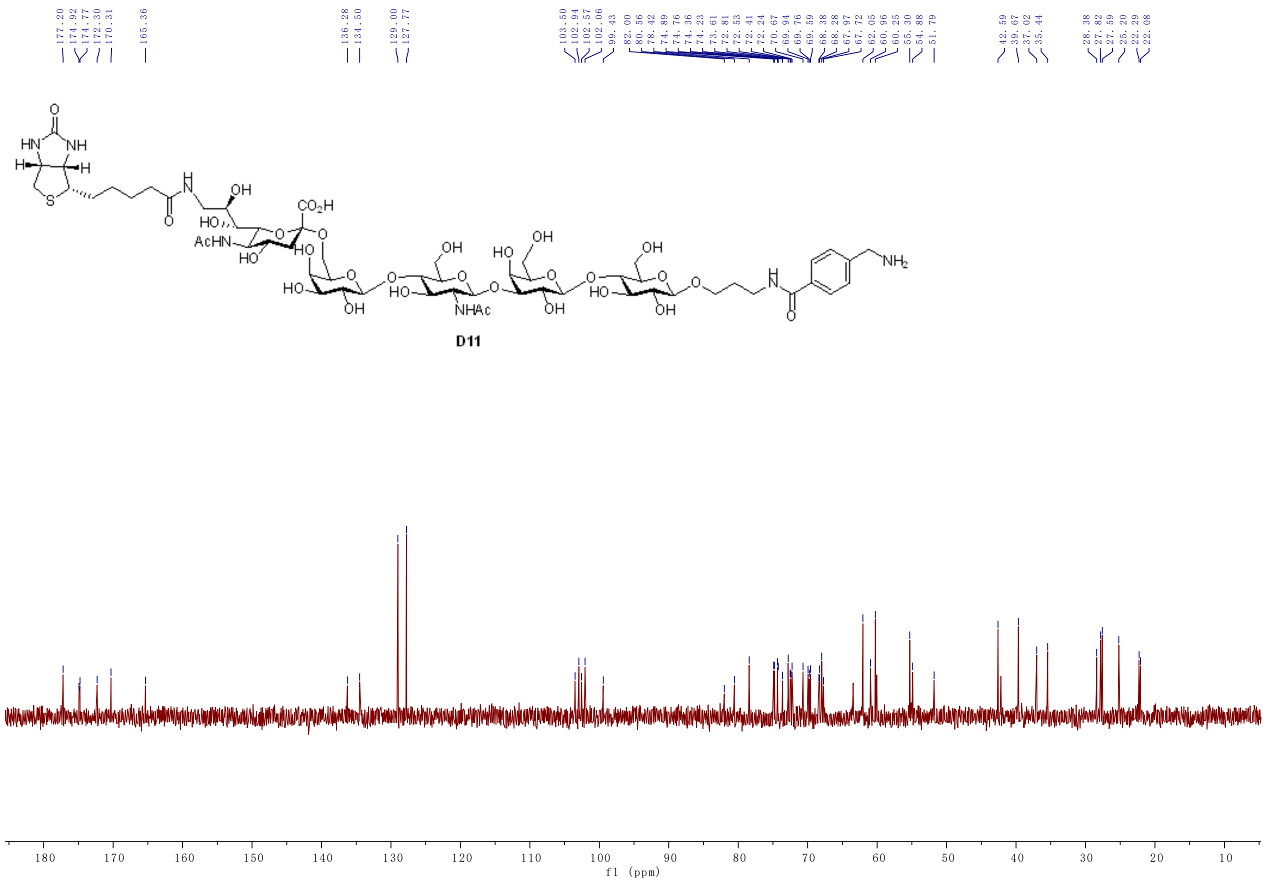

^{13}C NMR spectra of compound **D11**

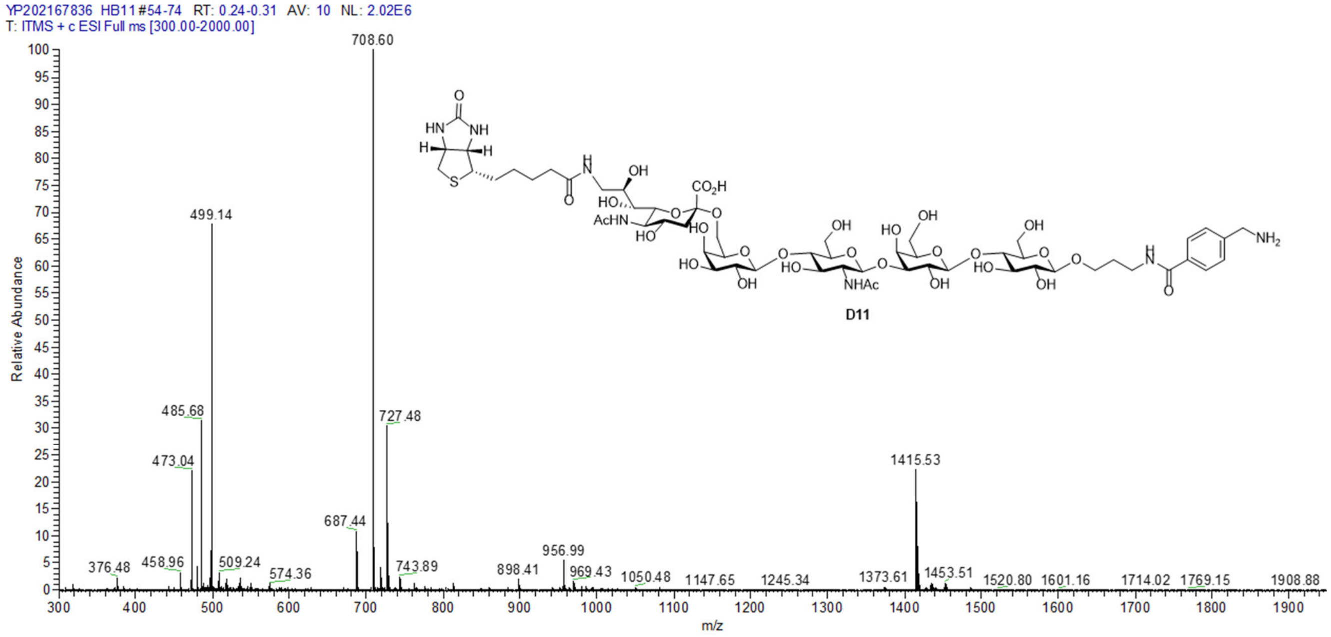

ESI-MS specta of compound **D11**

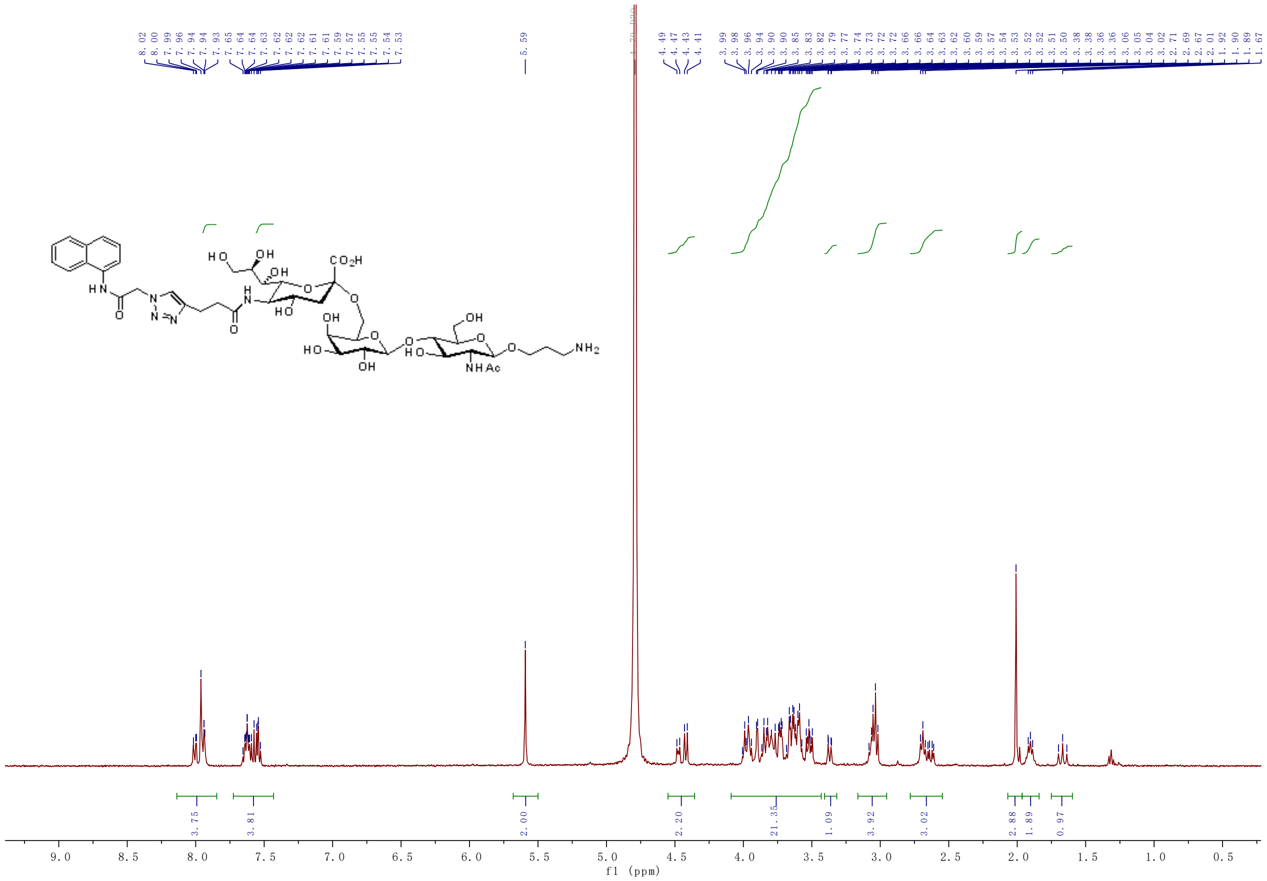

^{1}H NMR spectra of compound **D12**

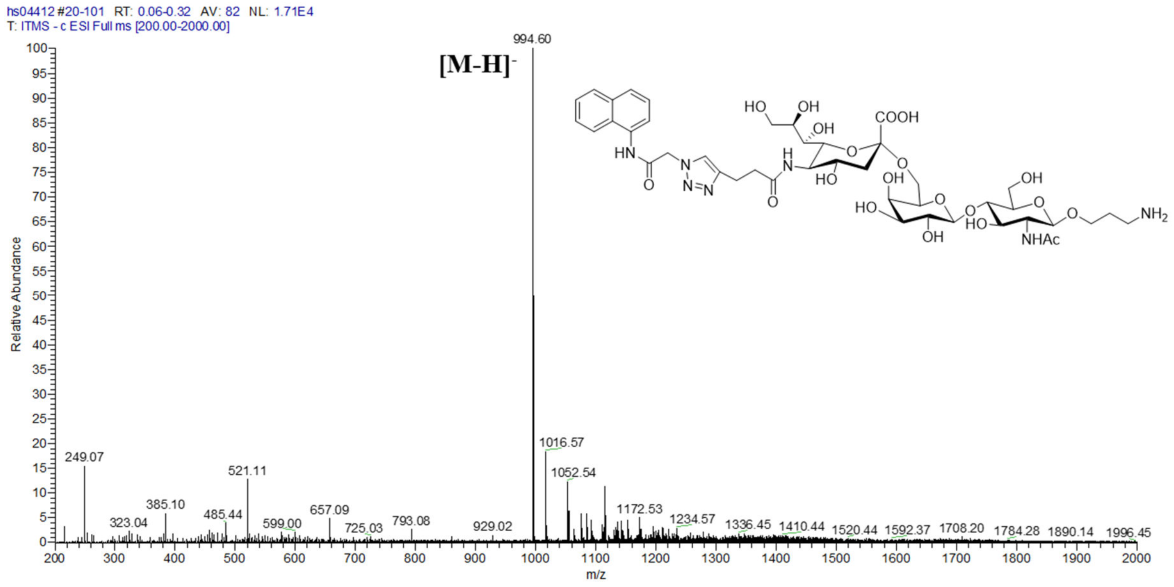

ESI-MS specta of compound **D12**

6. References

[1] O. Blixt, B. E. Collins, I. M. van den Nieuwenhof, P. R. Crocker, J. C. Paulson, "Sialoside specificity of the siglec family assessed using novel multivalent probes: identification of potent inhibitors of myelin-associated glycoprotein" *J. Biol. Chem.* **2003**, *278*, 31007-31019.

[2] W. Peng, J. Pranskevich, C. Nycholat, M. Gilbert, W. Wakarchuk, J. C. Paulson, N. Razi, "Helicobacter pylori beta1,3-N-acetylglucosaminyltransferase for versatile synthesis of type 1 and type 2 poly-LacNAcs on N-linked, O-linked and I-antigen glycans" *Glycobiology* **2012**, *22*, 1453-1464.

[3] O. Blixt, J. Brown, M. J. Schur, W. Wakarchuk, J. C. Paulson, "Efficient preparation of natural and synthetic galactosides with a recombinant beta-1,4-galactosyltransferase-/UDP-4'-gal epimerase fusion protein" *J. Org. Chem.* **2001**, *66*, 2442-2448.

[4] Y. Y. Xu, Y. Y. Fan, J. F. Ye, F. X. Wang, Q. D. Nie, L. Wang, P. G. Wang, H. Z. Cao, J. S. Cheng, "Successfully Engineering a Bacterial Sialyltransferase for Regioselective α2,6-sialylation" *Acs Catalysis* **2018**, *8*, 7222-7227.

[5] M. Gilbert, R. Bayer, A. M. Cunningham, S. DeFrees, Y. Gao, D. C. Watson, N. M. Young, W. W. Wakarchuk, "The synthesis of sialylated oligosaccharides using a CMP-Neu5Ac synthetase/sialyltransferase fusion" *Nat. Biotechnol.* **1998**, *16*, 769-772.

[6] N. Tasnima, H. Yu, Y. Li, A. Santra, X. Chen, "Chemoenzymatic synthesis of para-nitrophenol (pNP)-tagged alpha2-8-sialosides and high-throughput substrate specificity studies of alpha2-8-sialidases" *Org Biomol Chem* **2016**, *15*, 160-167.

[7] X. Zhou, K. Chandarajoti, T. Q. Pham, R. Liu, J. Liu, "Expression of heparan sulfate sulfotransferases in Kluyveromyces lactis and preparation of 3'-phosphoadenosine-5'-phosphosulfate" *Glycobiology* **2011**, *21*, 771-780.

[8] V. Hong, S. I. Presolski, C. Ma, M. G. Finn, "Analysis and optimization of copper-catalyzed azide-alkyne cycloaddition for bioconjugation" *Angew. Chem. Int. Ed. Engl.* **2009**, *48*, 9879-9883.

[9] M. M. Matthews, J. B. Mcarthur, Y. Li, H. Yu, A. J. Fisher, "Catalytic Cycle of Neisseria meningitidis CMP-Sialic Acid Synthetase Illustrated by High-Resolution Protein Crystallography" *Biochemistry* **2019**, *59*, 3157-3168.

[10] S. Krapp, A. K. Münster-Kühnel, J. T. Kaiser, R. Huber, J. Tiralongo, R. Gerardy-Schahn, U. Jacob, "The crystal structure of murine CMP-5-N-acetylneuraminic acid synthetase" *Journal of Molecular Biology* **2003**, *334*, 625-637.

[11] J. A. Maier, C. Martinez, K. Kasavajhala, L. Wickstrom, K. E. Hauser, C. Simmerling, "ff14SB: Improving the Accuracy of Protein Side Chain and Backbone Parameters from ff99SB" *J. Chem. Theory Comput.* **2015**, *11*, 3696–3713.

[12] M. J. Frisch, G. W. Trucks, H. B. Schlegel, G. E. Scuseria, M. A. Robb, J. R. Cheeseman, G. Scalmani, V. Barone, B. Mennucci, G. A. Petersson, "Gaussian 09; Gaussian Inc.: Wallingford CT, 2009".

[13] J. Wang, W. Wang, P. A. Kollman, D. A. Case, "Automatic atom type and bond type perception in molecular mechanical calculations" *J. Mol. Graph Model* **2006**, *25*, 247−260.

[14] J. Wang, R. M. Wolf, J. W. Caldwell, P. A. Kollman, D. A. Case, "Development and testing of a general Amber force field" *J. Comput. Chem.* **2004**, *25*, 1157−1174.

[15] D.A. Case, I.Y. Ben-Shalom, S.R. Brozell, D.S. Cerutti, I. T.E. Cheatham, V.W.D. Cruzeiro, T.A. Darden, R.E. Duke, D. Ghoreishi, M.K. Gilson, H. Gohlke, A.W. Goetz, D. Greene, R Harris, "AMBER 2018, University of California, San Francisco, 2018".

[16] Y. Zhou, C. Lu, Y. Ma, W. Qu, F. Ye, K. Zhang, L. Wang, M. Gui, Q. Gu, "SeedFold: Scaling Biomolecular Structure Prediction" *arXiv* **2025**, arXiv:2512.24354.

[17] E. Jurrus, D. Engel, K. Star, K. Monson, J. Brandi, L. E. Felberg, D. H. Brookes, L. Wilson, J. Chen, K. Liles, M. Chun, P. Li, D. W. Gohara, T. Dolinsky, R. Konecny, D. R. Koes, J. E. Nielsen, T. Head-Gordon, W. Geng, R. Krasny, G. W. Wei, M. J. Holst, J. A. McCammon, N. A. Baker, "Improvements to the APBS biomolecular solvation software suite" *Protein Sci.* **2018**, *27*, 112-128.

[18] L. Pravda, D. Sehnal, D. Tousek, V. Navratilova, V. Bazgier, K. Berka, R. Svobodova Varekova, J. Koca, M. Otyepka, "MOLEonline: a web-based tool for analyzing channels, tunnels and pores (2018 update)" *Nucleic Acids Res.* **2018**, *46*, W368-W373.

[19] Y. Li, H. Yu, H. Cao, S. Muthana, X. Chen, "Pasteurella multocida CMP-sialic acid synthetase and mutants of Neisseria meningitidis CMP-sialic acid synthetase with improved substrate promiscuity" *Applied Microbiology and Biotechnology* **2012**, *93*, 2411-2423.

[20] S. D. Pegan, Y. Tian, V. Sershon, A. D. Mesecar, "A universal, fully automated high throughput screening assay for pyrophosphate and phosphate release from enzymatic reactions" *Comb Chem High Throughput Screen* **2010**, *13*, 27-38.

[21] W. Peng, J. C. Paulson, "CD22 Ligands on a Natural N-Glycan Scaffold Efficiently Deliver Toxins to B-Lymphoma Cells" *J. Am. Chem. Soc.* **2017**, *139*, 12450-12458.

[22] G. W. Jourdian, L. Dean, S. Roseman, "The sialic acids. XI. A periodate-resorcinol method for the quantitative estimation of free sialic acids and their glycosides" *J. Biol. Chem.* **1971**, *246*, 430-435.

[23] Y. Liu, R. McBride, M. Stoll, A. S. Palma, L. Silva, S. Agravat, K. F. Aoki-Kinoshita, M. P. Campbell, C. E. Costello, A. Dell, S. M. Haslam, N. G. Karlsson, K. H. Khoo, D. Kolarich, M. V. Novotny, N. H. Packer, R. Ranzinger, E. Rapp, P. M. Rudd, W. B. Struwe, M. Tiemeyer, L. Wells, W. S. York, J. Zaia, C. Kettner, J. C. Paulson, T. Feizi, D. F. Smith, "The minimum information required for a glycomics experiment (MIRAGE) project: improving the standards for reporting glycan microarray-based data" *Glycobiology* **2017**, *27*, 280-284.

[24] O. Blixt, S. Han, L. Liao, Y. Zeng, J. Hoffmann, S. Futakawa, J. C. Paulson, "Sialoside analogue arrays for rapid Identification of high affinity Siglec ligands" *Journal of the American Chemical Society* **2008**, *130*, 6680-6681.

[25] C. M. Nycholat, C. Rademacher, N. Kawasaki, J. C. Paulson, "In silico-aided design of a glycan ligand of sialoadhesin for in vivo targeting of macrophages" *J. Am. Chem. Soc.* **2012**, *134*, 15696-15699.

[26] C. D. Rillahan, M. S. Macauley, E. Schwartz, Y. He, R. McBride, B. M. Arlian, J. Rangarajan, V. V. Fokin, J. C. Paulson, "Disubstituted Sialic Acid Ligands Targeting Siglecs CD33 and CD22 Associated with Myeloid Leukaemias and B Cell Lymphomas" *Chem Sci* **2014**, *5*, 2398-2406.

[27] C. D. Rillahan, E. Schwartz, R. McBride, V. V. Fokin, J. C. Paulson, "Click and pick: identification of sialoside analogues for Siglec-based cell targeting" *Angewandte Chemie International Edition* **2012**, *51*, 11014-11018.

[28] Z. S. Chinoy, C. Bodineau, C. Favre, K. W. Moremen, R. V. Durán, F. Friscourt, "Selective engineering of linkage-specific α2,6-N-linked sialoproteins using sydnone-modified sialic acid bioorthogonal reporters" *Angewandte Chemie International Edition* **2019**, *58*, 4281-4285.

[29] W. Peng, J. C. Paulson, "CD22 ligands on a natural N-glycan scaffold efficiently deliver toxins to B-lymphoma cells" *Journal of the American Chemical Society* **2017**, *139*, 12450-12458.

[30] D. Tsering, C. Chen, J. Ye, Z. Han, B.-q. Jing, X.-w. Liu, X. Chen, F. Wang, P. Ling, H. Cao, "Enzymatic synthesis of human blood group P1 pentasaccharide antigen" *Carbohydrate Research* **2017**, *438*, 39-43.

[31] S. Jana, S. Adhikari, M. R. Cox, S. Roy, "Regioselective synthesis of 4-fluoro-1,5-disubstituted-1,2,3-triazoles from synthetic surrogates of α-fluoroalkynes" *Chemical Communications* **2020**, *56*, 1871-1874.

[32] T. P. Gustafson, G. A. Metzel, A. G. Kutateladze, "Photochemically amplified detection of molecular recognition events: an ultra-sensitive fluorescence turn-off binding assay" *Organic & Biomolecular Chemistry* **2011**, *9*, 4752-4755.
